# Structural insights into Sld3-Sld7-dependent Cdc45 loading during replication initiation

**DOI:** 10.1038/s41467-026-76309-6

**Published:** 2026-08-14

**Authors:** Yasunori Noguchi, Almutasem Saleh, Sarah Schneider, Marina E. Ivanova, Zhuo Angel Chen, Lepakshi Ranjha, Ricardo Aramayo, Silvia Tognetti, Sarah V. Faull, Juri Rappsilber, Christian Speck

**Affiliations:** 1 DNA Replication Group, Institute of Clinical Sciences, Faculty of Medicine, Imperial College London, London, UK; 2 Technische Universität Berlin, Chair of Bioanalytics, Berlin, Germany; 3 MRC Laboratory of Medical Sciences (LMS), London, United Kingdom; 4 Si-M/‘Der Simulierte Mensch’, Technische Universität Berlin and Charité, Universitätsmedizin Berlin, Berlin, Germany; 5 Present Address: Department of Cellular Biochemistry, Graduate School of Pharmaceutical Sciences, Kyushu University, 3-1-1 Maidashi, Higashi-ku, Fukuoka, Japan; 6 Present Address: Institute of Molecular Biology (IMB), Mainz, Germany

**Keywords:** Replisome, Origin firing, Cryoelectron microscopy

## Abstract

Regulated helicase activation by DDK kinase is central for genome stability. However, how DDK phosphorylation primes the MCM2-7 double hexamer (DH) for Sld3-Sld7 binding and Cdc45 loading remained unclear. We define this mechanism through cryo-EM structures of MCM2-7 DH-Sld3-Sld7 (MS) and MCM2-7 DH-Sld3-Sld7-Cdc45 (MSC). We reveal that the autoinhibitory Mcm4 tail engages not only Mcm4 but also Mcm6. Upon DDK-dependent phosphorylation, both of these sites become accessible. In the context of the MS structure, we identify that two short Sld3 motifs that contact Mcm4 and Mcm6 read out the DH phosphorylation state, while the Sld3 Treslin domain (STD) binds to Mcm2. In the MSC structure, Cdc45 dislodges the Sld3 STD from Mcm2, allowing Sld3 to position Cdc45 at the Mcm2/Mcm5 interface. Mutagenesis of the Sld3 STD-Cdc45 interface disrupts Cdc45 loading, validating this interaction. Together, our data reveal a phosphorylation-encoded mechanism coupling DDK-activated Mcm4/Mcm6 surfaces to distal Cdc45 placement, explaining how firing factors choreograph the DH-to-CMG transition.

## Abstract (web summary)

DNA replication depends on loading Cdc45 onto the MCM2-7 helicase to start DNA unwinding. Here, the authors use cryo-EM to show how phosphorylation enables Sld3-Sld7 to bind MCM2-7 and position Cdc45 for helicase activation.

## Introduction

The accurate and complete duplication of the eukaryotic genome during the S-phase of the cell cycle is a fundamental aspect of cellular biology, ensuring the faithful transmission of genetic information from one generation to the next. During each cell cycle, a completely new replication fork must be assembled from individual components. This highly conserved and tightly regulated process begins with the loading of the replicative helicase onto DNA origins, also termed pre-replicative complex (pre-RC) formation, followed by activation of the DNA replication motor, unwinding of the DNA double helix, and synthesis of new DNA strands.

During pre-RC formation, the origin recognition complex (ORC), Cdc6 and Cdt1 load two copies of the hexameric minichromosome maintenance 2–7 (MCM2-7) complex on double-stranded DNA^1,2^. MCM2-7 is loaded as an inactive helicase in an N-terminal head-to-head configuration called the MCM2-7 double hexamer (DH)^1,3–5^. This inactive assembly persists until the S-phase, where it becomes activated through the recruitment of a series of factors. This results in the assembly of two replication forks, each with the CMG (Cdc45-MCM2-7-GINS) helicase motor complex at its core, driving bidirectional DNA replication^6–9^.

MCM2-7 helicase activation requires two essential S-phase kinases, which act sequentially: Dbf4-dependent kinase (DDK), which consists of Cdc7 and Dbf4, and S-phase cyclin-dependent kinase (S-CDK)^10^. DDK first recognizes and phosphorylates the unstructured N-terminal tails of Mcm2, Mcm4 and Mcm6 on the MCM2-7 DH (Supplementary Fig. 1). The phosphorylation primarily alleviates the autoinhibitory function of the Mcm4 N-terminal tail and provides a regulatory step that allows the stepwise recruitment of Sld3-Sld7 and Cdc45 (Supplementary Fig. 1)^11,12^. S-CDK then phosphorylates Sld2 and Sld3 (Supplementary Fig. 1), promoting the recruitment of Sld2, Dpb11, GINS and DNA polymerase ε, leading to CMG^6,13–17^. Finally, when MCM10 is recruited, the CMG complex becomes active for processive DNA unwinding^14,18–20^.

Excessive activation of replication forks leads to exhaustion of nucleotides, histones, and the single-stranded DNA-binding protein RPA, resulting in severe genomic instability^21–23^. Thus, helicase activation is tightly regulated at multiple steps. Specifically, Dbf4, Sld3, Sld7, and Cdc45 are expressed at very low levels, limiting the number of replication forks that can be activated simultaneously in the cell^12,24^. Moreover, during DNA damage, the Rad53 kinase phosphorylates Sld3 and Dbf4, suppressing late-origin firing and limiting new replication fork assembly^11,25,26^. Thus, the DDK-dependent recruitment of Sld3-Sld7 and Cdc45 represents a key regulatory step that controls replication fork assembly.

Recently, cryo-EM studies have identified how DDK is recruited to the MCM2-7 DH, how it phosphorylates multiple and distal subunits, and that DDK mainly targets the Mcm4 N-terminal tail^27–29^. Biochemical work identified a region in Sld3 (aa510-545) that promotes DDK-dependent binding of Sld3 to Mcm4 and Mcm6^11^. The data also suggested that a second region (aa251-325) may also be necessary for DDK-dependent recruitment of Sld3 to MCM2-7^11^ (Supplementary Fig. 2a). However, how Sld3 can distinguish between the DDK-phosphorylated and the unphosphorylated DH remains unclear. Moreover, although we know that Sld7 is required for regulated origin firing and normal replication efficiency^30^, it is unknown how Sld7 is recruited to the MCM2-7 DH and how it enhances Sld3 function. The current understanding is that Sld3 can bind DDK-phosphorylated Mcm4/Mcm6 N-terminal tails at the DH interface^11^, but how Sld3 can, at the same time, recruit Cdc45 to the distal Mcm2/Mcm5 interface remains elusive^13^. It is also unclear whether Cdc45 binding restructures the MCM2-7 hexamer to support GINS binding, whether GINS binding triggers Sld3 release, or whether another factor is responsible. Now, our structural, proteomic and biochemical work identifies how Sld3 can recognize the DDK-activated MCM2-7 DH and how Sld3 dynamics support MCM2-7 recognition and distal Cdc45 recruitment. Together, our data provide a structural framework for DDK-dependent activation of DNA replication.

## Results & discussion

### DDK releases an Mcm4 autoinhibitory loop that binds to rigid surfaces on Mcm4 and Mcm6

**Fig. 1:**
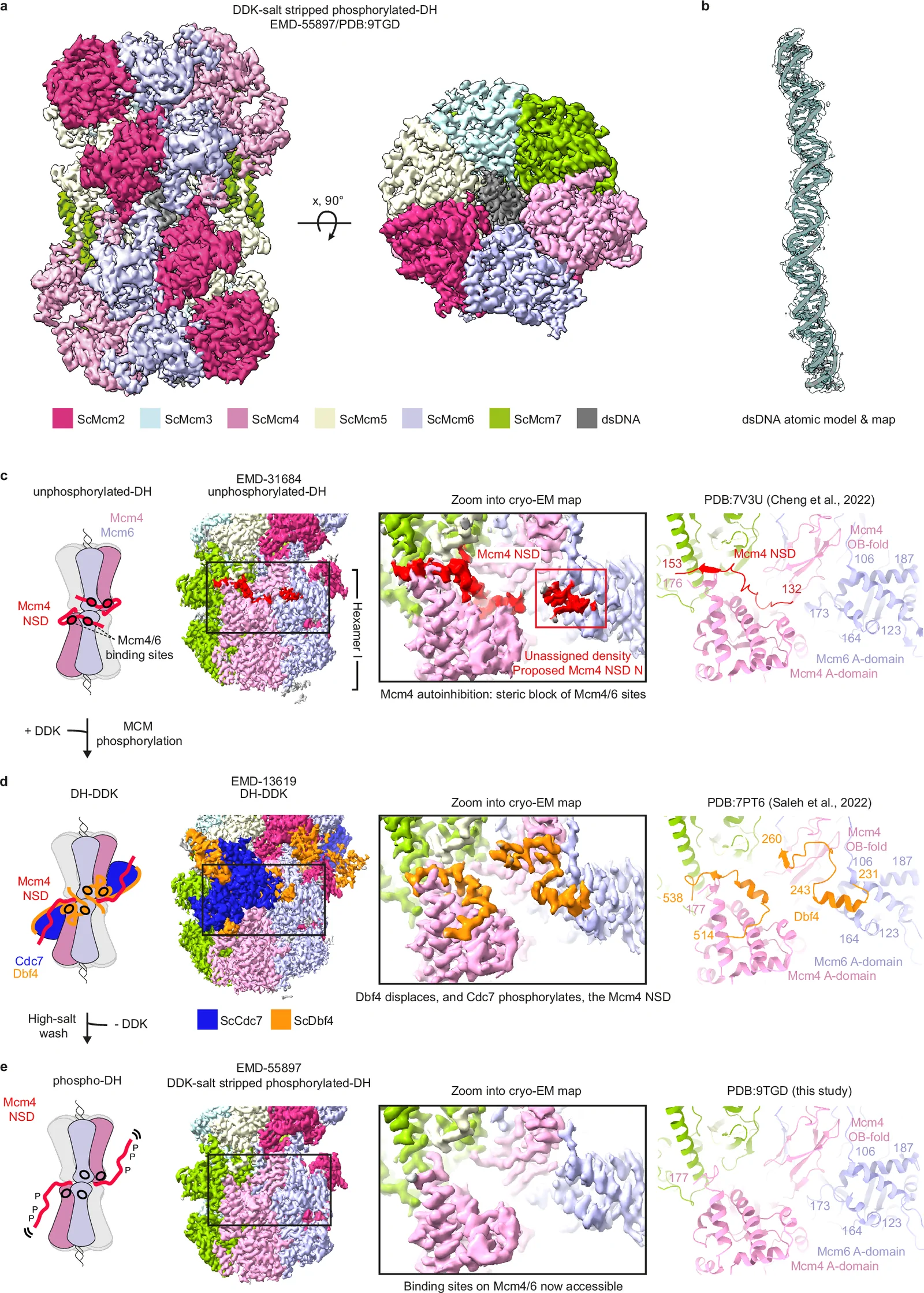
Mcm4 N-terminal serine/threonine-rich domain (NSD) autoinhibition is alleviated within the DDK-salt stripped phosphorylated MCM2-7 double hexamer (DH). **a** Cryo-EM map of the DDK-salt stripped phosphorylated MCM2-7 DH complexed with DNA, resolved at a mean resolution of 3.3 Å (this study; PDB:9TGD/EMD-55897). **b** Surface map clipping of the dsDNA and derived atomic model, illustrating the quality of the map. **c–e**. Cryo-EM maps and corresponding illustrations of distinct, sequential intermediate complexes formed during DH helicase activation and structural changes to the MCM2-7 DH core due to the enzymatic activity of DDK phosphorylation. Zoomed-in views of each map and its associated atomic model are shown. **c** The Mcm4 NSD (N-terminal serine/threonine-rich domain, colored red) has an autoinhibitory function that prevents helicase activation by sterically blocking activation factor binding sites on rigid regions of Mcm4 (pink, NSD in red) and Mcm6 (lilac). The region of Mcm4 NSD spanning aa. 132–153 in the unphosphorylated DH state (EMD-31684) has previously been shown to interact with the Mcm4 terminal domain. However, there is unassigned additional density adjacent to this Mcm4 NSD region, which is most consistent with Mcm4 N-terminal residues preceding the previously modeled NSD segment (132–153) and can be clearly seen bound on the rigid region of Mcm6. **d** DH bound by the kinase DDK (Dbf4/Cdc7): the zoomed view shows only Dbf4 densities (orange) that bind at the same sites as the Mcm4 NSD in the unphosphorylated state. **e** DH core after DDK is salt-stripped. MCM2-7 DH phosphorylation by DDK removes the autoinhibition. This is attributed to the structural rearrangement of the Mcm4 NSD, which is no longer observed in the phosphorylated DH.

The MCM2-7 DH acts as the assembly platform for the eukaryotic replication fork^31^. Initially, it is kept inactive by the autoinhibitory domain in Mcm4^32^. In late G1-/early S-phase, this N-terminal serine/threonine-rich domain (NSD) of Mcm4 becomes phosphorylated by DDK at multiple sites, relieving the autoinhibition^32^. Here we have generated a 3.3 Å resolution structure of the DDK-phosphorylated MCM2-7 DH bound to DNA, with DDK absent (Fig. 1a, b, Supplementary Fig. 3 and Supplementary Tables 1–3), which serves as a reference point for understanding Mcm4 autoinhibition. Cheng et al. (2022) reported the structure of the unphosphorylated MCM2-7 DH (EMDB-31684), which showed that Mcm4 N-terminal NSD (aa132-152) contacts rigid regions of the Mcm4 N-terminal domain within the DH core^29^ (Fig. 1c). Moreover, the map features additional unmodelled density adjacent to the rigid Mcm6 N-terminal domain surface (Fig. 1c), which overlaps with the Dbf4-binding site observed in DDK-DH maps (Fig. 1d) and absent in our DDK-salt stripped phosphorylated MCM2-7 map (Fig. 1e) and all other available DDK-phosphorylated MCM2-7 maps^27–29^. Importantly, while the density adjacent to Mcm4 is retained in the DHΔN140 truncation mutant, the density adjacent to Mcm6 is absent in both the DHΔN140 and DHΔN174 mutants^29^; however, this observation was not mentioned in the corresponding study^29^. The data highlight that the N-terminal tail of Mcm4 contacts not only the rigid Mcm4 N-terminal domain but also the rigid Mcm6 N-terminal domain. Thus, upon DDK phosphorylation, the flexible Mcm4 N-terminal tail is released, and the Mcm4 and Mcm6 N-terminal domain surfaces become available for Dbf4 binding in the DH-DDK complex (Fig. 1d). This indicates that Dbf4 and the unstructured Mcm4 NSD compete for overlapping interaction sites on MCM2-7.

In summary, the data suggest that the Mcm4 N-terminal autoinhibitory domain contacts both Mcm4 and Mcm6, thereby forming a more extensive interaction surface. DDK phosphorylation of Mcm4 induces a conformational change in the Mcm4 tail, leading to dissociation of the Mcm4 N-terminal autoinhibitory domain and rendering the Mcm4 and Mcm6 sites accessible to DDK. It is unclear if other factors involved in helicase activation bind to the same site as Dbf4 and the autoinhibitory NSD of Mcm4.

### Sld3-Sld7 and Sld3-Sld7-Cdc45 form relatively compact dimers

Sld3, Sld7 and Cdc45 have been investigated structurally^33–35^ (Supplementary Figs. 2, 4, 5). Crystal structures of portions of Sld3 and Sld7 have resolved the Sld7 dimerization domain (DD), the Sld3-Sld7 N-terminal dimerization domains (ND) and the Sld3/Treslin domain (STD)^34,35^. Whilst the crystal structure of human Cdc45 has been solved^36^, the yeast Cdc45 structure has only been identified in the context of the CMG complex^37,38^. To better understand the potential oligomerization state of the Sld3-Sld7 complex and Cdc45 (Supplementary Fig. 6a), we performed native (without crosslinker) glycerol gradient ultracentrifugation sedimentation analysis. This showed that Sld3 and Sld7 cofractionated over a wide range (Supplementary Fig. 6b), suggesting the presence of distinct species, whereas Cdc45 appeared more homogeneous, fractionating over a narrower range (Supplementary Fig. 6c). To resolve individual complexes, we performed BS3 crosslinking followed by SDS-PAGE analysis. With Sld3 and Sld7, we observed formation of multiple complexes at 50 μM BS3 (Supplementary Fig. 6d, lane 3), which we tentatively assigned as Sld3-Sld7, Sld3-(Sld7)^2^ and (Sld3-Sld7)^2^ (Supplementary Fig. 6a). In contrast, Cdc45 appeared to be monomeric (Supplementary Fig. 6e). Sld3-Sld7-Cdc45 formed a higher-order complex that was poorly resolved (Supplementary Fig. 6f). Therefore we separated the Sld3-Sld7 complexes in a glycerol gradient with increasing concentrations of glutaraldehyde, resulting in gradient fixation (GraFix)^39^ (Supplementary Fig. 6g). SDS-PAGE analysis of the crosslinked fractions indicated complexes consistent with Sld3-Sld7, Sld3-(Sld7)^2^ and (Sld3-Sld7)^2^ (Supplementary Fig. 6g). In both native and GraFix conditions, Sld3 and Sld7 co-migrated in the same fractions, indicating that the observed complex is not an artifact of crosslinking and that crosslinking did not detectably alter complex formation. These species were further verified by mass photometry (Supplementary Fig. 6h) and are consistent with AlphaFold analysis of the (Sld3-Sld7)^2^ complex (Supplementary Fig. 4, Supplementary Table 4). Electron microscopy (EM) identified an envelope (Supplementary Fig. 7a–c), which can accommodate all the resolved regions of Sld3/7^34,35^ (Supplementary Fig. 2) and suggests a (Sld3-Sld7)^2^ complex arrangement (Supplementary Fig. 6i). The EM envelope also featured a near-C2 symmetric arrangement, but the symmetry was imperfect, most likely due to the flexibility of the unstructured protein sections.

AlphaFold3 predictions were generated for both the monomeric Sld3-Sld7-Cdc45 complex and the dimeric (Sld3-Sld7-Cdc45)₂ complexC (Supplementary Fig. 5; Supplementary Table 4).

Similar analysis of crosslinked fractions of the Sld3-Sld7-Cdc45 complex identified a large complex of ~385 kDa, which is consistent with the AlphaFold prediction of an (Sld3-Sld7-Cdc45)^2^ complex mediated by Sld7 dimerization (Supplementary Figs. 5, 6j–l, Supplementary Table 4). EM analysis revealed an envelope that can accommodate a dimeric complex consisting of the reported structures of Sld3-Sld7^34,35^ and an AlphaFold model of Cdc45-Sld3 (STD) (Supplementary Figs. 5b–d, 6l and 7d-f). Thus, we conclude that under our crosslinked experimental conditions, the Sld3-(Sld7)^2^, (Sld3-Sld7)^2^, and (Sld3-Sld7-Cdc45)^2^ complexes can form in solution. The data suggest that the Sld3-(Sld7)^2^ complex could serve as an intermediate in the formation of the larger (Sld3-Sld7)^2^ complex, suggesting that the Sld3-Sld7 complex initiates dimerization via Sld7.

### The structure of MCM2-7 DH-Sld3-Sld7 (MS) complex

**Fig. 2:**
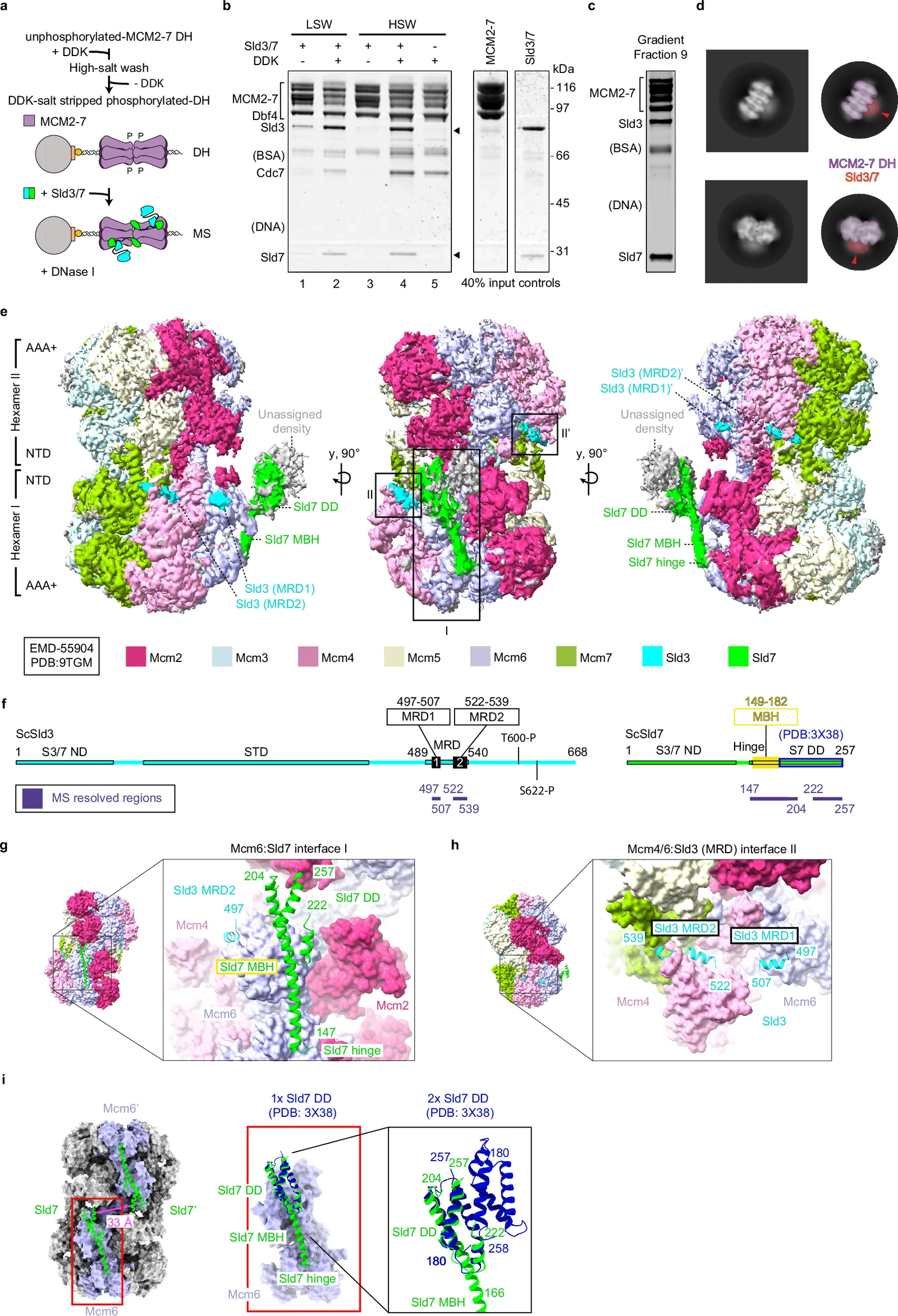
Sld3/7 assembles near the N-terminal head-to-head double-hexamer (DH) interface and can recognize and bind the DDK-phosphorylated DH. **a** Assembly pathway for the MCM2-7 DH-Sld3/7 (MS) complex. For the final step, Sld3/7 were incubated with phospho-DH, DNA, and ATP to facilitate complex assembly. DNase I released DNA-bound complexes from magnetic beads. **b** Silver-stained SDS-PAGE analysis of complex formation in low (LSW) and high-salt wash (HSW) conditions (*n* = 3). **c** Peak fraction of the in vitro assembled and native (no crosslinker) glycerol gradient-purified MS complex. **d** Cryo-EM 2D class averages of the MS complex. At the center N-lobe of the DH (purple), diffuse/flexible density corresponding to Sld3/7 (red) is observed. **e** Composite cryo-EM map of the MS complex at a mean resolution of 3.6 Å. Sld3/7 regions interacting with MCM2-7 are labeled I, II and II’ (middle panel): I) Sld7 (green) MCM binding helix (MBH) forms a long alpha-helix along the surface of Mcm6, II) Sld3 (cyan) MCM recognition domain (MRD) binds, and anchors Sld3, on Mcm4 and Mcm6 and II’) a second Sld3 MRD bound across the second hexamer in a C2 symmetric configuration compared to II). There is unassigned density near the Sld7 dimerization domain (DD, gray). **f** Domain organization/features of Sld3/Sld7. Sld3/7 N-terminal dimerization (S3/7 ND) domain, Sld3-Treslin domain (STD), MCM recognition domain (MRD - which encompasses the MCM6 (MRD1) and Mcm4 (MRD2) recognition domains). S-CDK phosphorylation sites (aa. T600 and S622) are indicated on Sld3. The purple bar denotes regions resolved in the MS atomic model. **g**,** h** Close-up view of the **g** Sld7 binding interface with Mcm6, featuring the Sld7 MBH, and **h** Sld3 binding interface with Mcm4/Mcm6, featuring Sld3 MRD1/2. Sld3/7 is shown as a cartoon ribbon, and MCM2-7 DH as a surface. **i** Sld7 dimerizes via the Sld7 DD. In the MS consensus map before focused processing, the DD interface is separated by 33 Å. Zoomed views show how the second Sld7 subunit forms the dimerization interface. The Sld7 DD crystal structure (PDB: 3X38, blue) is superimposed onto Sld7 and Mcm6 from the MS atomic model. Source data are provided as a Source Data file.

To understand how Sld3-Sld7 become recruited to the DDK-phosphorylated MCM2-7 DH, we reconstituted the MCM2-7 DH-Sld3-Sld7 (MS) complex (Fig. 2a). Our experiments indicated that DDK was required for optimal recruitment of Sld3-Sld7 (Fig. 2b). To purify the sample for cryo-EM, the DDK-treated MCM2-7 DH was high-salt-washed and eluted from DNA-tethered beads with AluI. Subsequently, Sld3-Sld7 was added, and the sample was separated by GraFix^39^. The peak fraction of an uncrosslinked identical control gradient contained near stoichiometric amounts of MCM2-7, Sld3 and Sld7, indicative of a stable complex (Fig. 2c). Analysis of cryo-EM 2D class averages revealed that Sld3-Sld7 binds across the DH interface. The diffuse density indicates that Sld3-Sld7 may adopt multiple conformations (Fig. 2d). We solved the structure of the MS complex at 3.6 Å mean resolution, with a local resolution range of 2.5–8.0 Å (Fig. 2e, Supplementary Figs. 8–10, Supplementary Tables 1–3 and Supplementary Movie 1). The final model was built using a composite map generated from focused refinements of locally resolved regions, which improved the interpretation of peripheral Sld3-Sld7 densities.

The glycerol gradient ultracentrifugation purification method is ideal for purifying a more homogeneous complex for structural studies, but a disadvantage for this particular complex is that the DNA can slide out of the MCM2-7 DH, as previously observed with the MCM2-7 DH purified from yeast^1,5^. Despite this, the loss of DNA from the purified MCM2-7 DH does not substantially alter the overall conformation of the DH, as DNA-bound and DNA-free structures are essentially identical^3,5^, providing confidence in the MS complex structure.

The MS complex identified several fixed Sld3-Sld7 attachment points on the MCM2-7 DH: Sld3 itself makes multiple contacts with the MCM2-7 DH (Fig. 2e, boxes II and II’), including contacts that we describe in the lower-resolution MS consensus map in a later section of the manuscript. Two of the Sld3-MCM2-7 attachment points include a short helix in Sld3, termed the MCM2-7 recognition domain 1 (MRD1, aa497-507), which recognizes Mcm6, and a second helix in Sld3 (MRD2, aa522-539), which binds to Mcm4 (Fig. 2f–h, Supplementary Fig. 11a). As these helices are at the periphery of the complex, we used an integrative approach to model them based on (i) structural information, including local-resolution features, together with AlphaFold predictions (Supplementary Fig. 10), and (ii) biochemical information, such as Sld3-MCM2-7 interaction experiments, mutagenesis, and functional data^11^.

Whilst Sld3 binds to Mcm2 and Mcm4, Sld7 binds to the DH at the interface of Mcm2 and Mcm6 (Fig. 2e). Sld7 mediates this interaction by the newly defined Sld7 MCM Binding Helix (MBH, aa149-182) and also features two Sld7 C-terminal helices (Fig. 2e, box I, and 2f–g, Supplementary Fig. 11b). For the peripheral Sld3 MRD1/MRD2 motifs and the Sld7 MBH/C-terminal helices, the local density supports their overall placement on the MCM2-7 DH and permits tracing of the main backbone features. Because map quality varies across these regions, we interpret them conservatively as backbone level assignments and do not infer uniform side-chain or sequence register certainty. In addition, we noticed additional density adjacent to Sld7, but the resolution was insufficient to assign it to a specific protein (Fig. 2e).

Moreover, we observed that both MCM2-7 hexamers are occupied by Sld3 MRD1/2, but the remaining Sld3 and Sld7 densities were best resolved on only one MCM2-7 hexamer (Fig. 2e). This apparent asymmetry may arise from several factors, including heterogeneous Sld3-Sld7 occupancy during purification, the focused classification strategy used to improve local density or conformational flexibility, and therefore is unlikely to be associated with biological function. Nevertheless, this structural analysis suggests that the Sld3 MRDs serve as a key attachment point on MCM2-7, consistent with biochemical work^11^.

### Sld3-Sld7 dimer splitting across the MCM2-7 double hexamer

Sld7 is known to form dimers via its C-terminal dimerization domain (DD) (Supplementary Fig. 2a, b)^35^. Consistent with this, our biochemical analysis, structural data, and AlphaFold predictions indicate that Sld3-Sld7 forms a (Sld3-Sld7)^2^ complex primarily through dimerization of the Sld7 C-terminal DD (Supplementary Figs. 4, 6). To determine what happens to this dimer upon MCM2-7 DH binding, we examined the low-resolution features of the consensus MS map (Supplementary Fig. 8b), which reveal density consistent with Sld3-Sld7 engaging both hexamers of the DH. In this configuration, which we modeled by generating a C2-symmetric MCM2-7 DH-Sld7 model based on the MS atomic model, we found that the two Sld7 dimerization domains are separated by approximately 33 Å (Fig. 2i), a distance incompatible with the compact Sld7 homodimer interface observed in the crystal structure^35^. This indicates that the (Sld3-Sld7)^2^ dimer splits upon DH engagement, with each Sld3-Sld7 monomer binding one hexamer. The functional relevance of Sld7 dimerization during DDK-dependent helicase activation remains to be explored.

### Sld3-Sld7 recognize the DDK-phosphorylated MCM2-7 DH

**Fig. 3:**
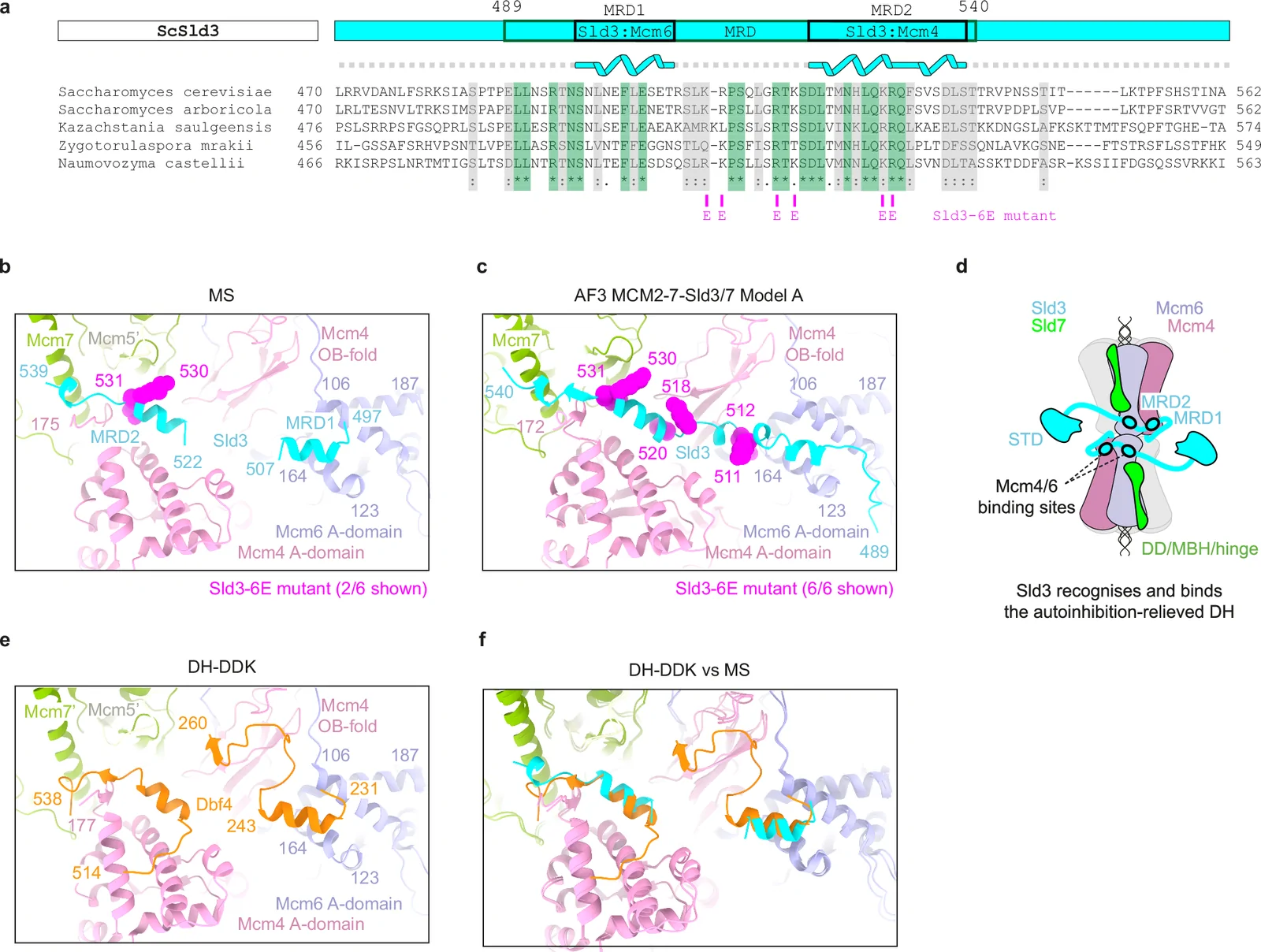
Sld3 MCM recognition domain (MRD) confers DDK-phosphorylated MCM2-7 double hexamer (DH) recognition and anchors Sld3 to the DH. **a** A sequence alignment of Saccharomyces cerevisiae Sld3 and related homologs. Domain boundaries for the Sld3 MRD were defined using both the MCM2-7 DH-Sld3/7 (MS) atomic model and sequence alignment. The Sld3 region, denoted as MRD1, interacts with Mcm6, and MRD2 interacts with Mcm4. The six basic residues substituted with glutamate (E) in the Sld3-6E mutant^11^, which abolish Sld3 binding to the DDK-phosphorylated DH, are indicated. The mutant impairs DDK-dependent binding to Mcm4/6 without affecting Sld3’s ability to bind Cdc45 or Sld7, suggesting that this region plays a role in anchoring Sld3 to the DH. **b** Substituted residues (magenta) in the Sld3-6E mutant within the MS atomic model. Four of the substitutions lie within an intrinsically disordered region between MRD1 and MRD2 (cyan), which could not be modeled and therefore these residues are not shown. **c** AlphaFold3 MS model A (Supplementary Fig. 24) showing the complete MRD domain region (aa. 489–540) prediction and plot of all substitutions within the Sld3-6E mutant (magenta). **d** Illustration of (**b**, **c**) summarizing the proposed molecular mechanism of Sld3 anchoring and DDK-phosphorylated DH recognition. Sld3 recognizes and binds to the DH via its MRD domains (cyan). **e** Zoomed-in view of the DH-DDK complex atomic model (PDB: 7PT6) at the Mcm4/6 interface, featuring sections of Dbf4 that contribute to the displacement of the autoinhibitory Mcm4 NSD on rigid Mcm4/6 sites. **f** A zoomed-in view of the superposition of the DH-DDK and MS atomic models at the Mcm4/6 interface, featuring the Sld3 MRD bound to the same rigid Mcm4/6 sites as Dbf4. The clash of helices and a similar binding manner suggest that Sld3 displaces Dbf4 upon binding at these Mcm4/6 sites.

Previously, the Sld3 MRD1/MRD2 regions, which we have modeled at backbone level confidence (Fig. 2h), were shown to be essential for Sld3 interaction with MCM2-7. Specifically, an Sld3-6E mutant with six amino acids substituted for glutamate disrupted the Sld3-MCM2-7 interaction^11^ (Fig. 3a). By mapping the locations of these mutations on the structure (Fig. 3b), it is clear that one glutamic acid at position Sld3 530 disrupts the interaction. To understand the impact of neighboring substitutions, we modeled the region using AlphaFold (Fig. 3c). This enabled us to understand the potential of the neighboring glutamic acid substitutions to disrupt additional Sld3-Mcm4/Mcm6 interactions. Thus, the analysis highlights that we identified the structure of an essential Sld3-MCM2-7 interaction surface.

As discussed, within the non-phosphorylated MCM2-7 DH, the Sld3 contact surface in Mcm4 and Mcm6 is occluded by the autoinhibitory NSD of Mcm4 (Figs. 1c, 3d). However, DDK-dependent phosphorylation of the Mcm4 NSD releases the autoinhibitory activity of Mcm4 (Figs. 1d, e, 3d) and allows Dbf4 to contact Mcm4 and Mcm6 (Fig. 3e). In our structure, we show that Sld3 also contacts Mcm4 and Mcm6 (Fig. 3b, d) and comparison with the DDK-DH structure reveals a complete overlap between the Dbf4 and Sld3 binding sites (Fig. 3f). Thus, we structurally resolved how Sld3 reads the phosphorylation status of MCM2-7, and we conclude that the autoinhibitory NSD of Mcm4, Dbf4 and Sld3 contact the same binding sites on Mcm4 and Mcm6.

### MCM2-7 ATP status and structural change

**Fig. 4:**
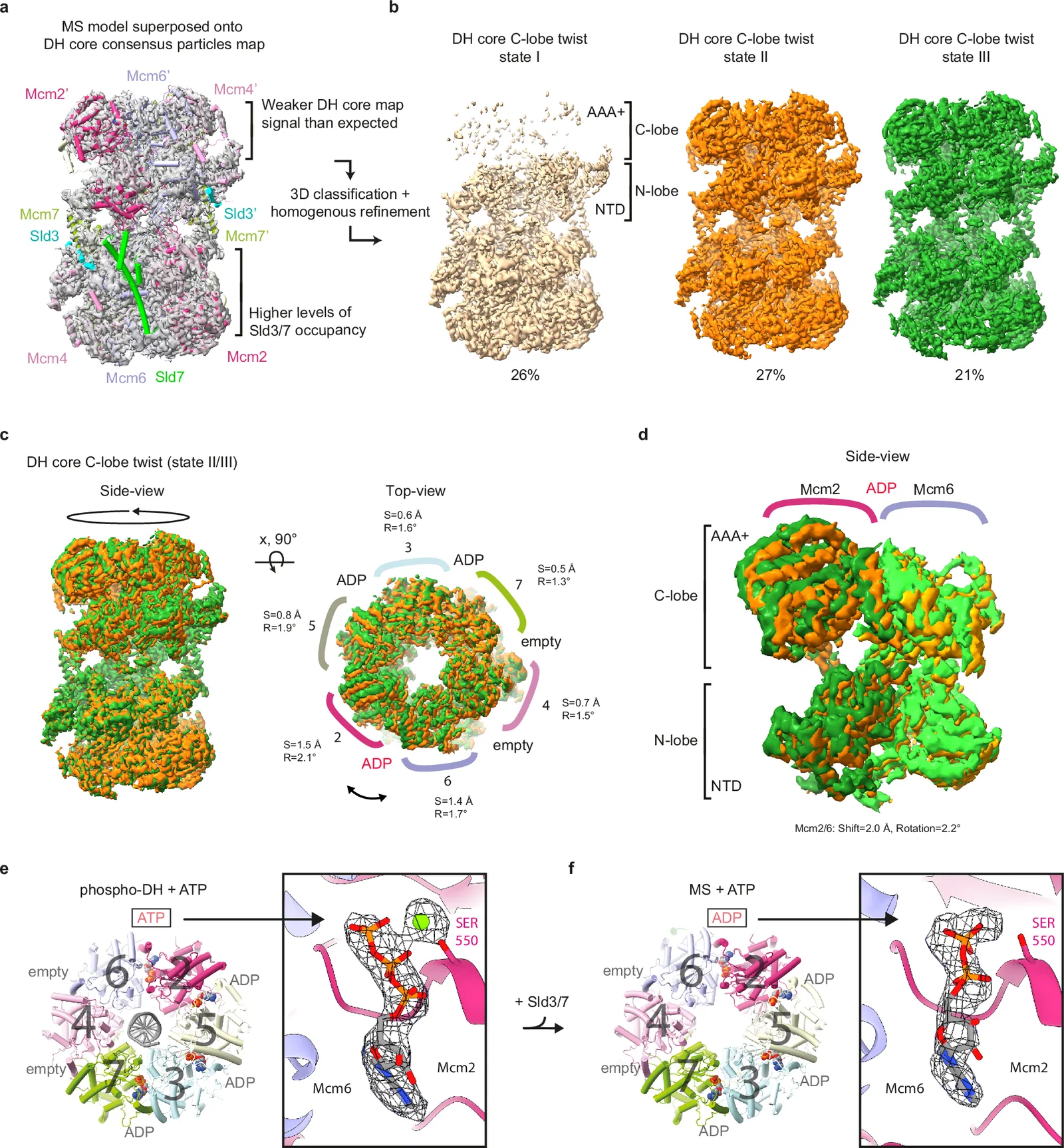
Sld3-Sld7 binding is associated with MCM2-7 DH C-lobe twisting and nucleotide state change relative to the phospho-DH. **a** The MS atomic model was derived from several focused maps combined to form a composite map, one of which was the focused DH core map originating from a consensus particle subset that displayed unexpected conformational heterogeneity. The DH core consensus map showed greater Sld3/7 occupancy on one hexamer than on the opposing hexamer, where the density was weaker, particularly at the C-lobe of MCM2-7. **b** The conformational heterogeneity of the C-lobe region can be attributed to a twisting motion observed from the different maps generated. State I map shows the most diffuse twisting conformation, and state II/III shows more rigid snapshots of this motion. **c** The DH core C-lobe twist state II/III maps superimposed, showing distinct movements in Mcm subunits. Both maps feature ADP at the Mcm2/6 interface, and the movement is mirrored on both hexamers. S represents the shift distance, and R represents the degree of rotation relative to the phospho-DH atomic model. **d** The greatest range difference between subunits occurs between Mcm2/6, with a shift of 2 Å and rotation of 2.2°. **e** The Mcm2/6 interface is occupied by ATP (zoomed in density and coordinating Mg shown in box) in the phosphorylated DH atomic model. **f** The Mcm2/6 interface contains ADP in the MS structure, suggesting Sld3/7 binding promotes ATP hydrolysis or nucleotide exchange at the Mcm2/6 interface.

The MCM2-7 DH core of the MS complex was resolved at a mean resolution of 3.4 Å (Fig. 4a), enabling us to elucidate its organization and structure. Interestingly, after performing 3D classification on the MS DH core consensus map, we observed four classes, including one with a poorly resolved C-lobe, two twisted states (Fig. 4b) and an intermediate twisted state (Supplementary Fig. 8c). Comparing the twisted states shows the movement of the C-lobe in each hexamer (Fig. 4c). Interestingly, the largest twist appears at the Mcm2/6 interface, which is contacted by the Sld3-Sld7 complex (Fig. 4a, d), implying that Sld3/Sld7 binding may impose torsional stress on the DH. Analyzing the nucleotide state of the C-lobe AAA+ domains in all three twisted states also revealed a change in the Mcm2/6 interface during the transition from the phosphorylated MCM2-7 DH to the MS complex. Initially, ATP is in the Mcm2/6 pocket, but upon Sld3-Sld7 binding, we observe ADP (Fig. 4e, f). In summary, we correlate Sld3/Sld7 binding to the Mcm2/6 interface with associated changes in nucleotide occupancy at the Mcm2/6 interface with increased flexibility within the C-lobe. However, whether these changes merely reflect Sld3 binding or have a functional role in helicase activation is currently unknown.

### Flexible links between Sld3 and MCM2-7 position Sld3 at the MCM2-7 DH interface

The 2D classes of the MCM2-7 DH-Sld3-Sld7 complex suggested that the Sld3-Sld7 interaction with the MCM2-7 DH is flexible (Fig. 2d), but localized around the center of the DH. Moreover, multi-body analysis of an independently refined MS particle set revealed the relative motion of the Sld3-Sld7 density on the MCM2-7 DH, including rotational and translational movements (Supplementary Fig. 12, Supplementary Movie 2).

**Fig. 5:**
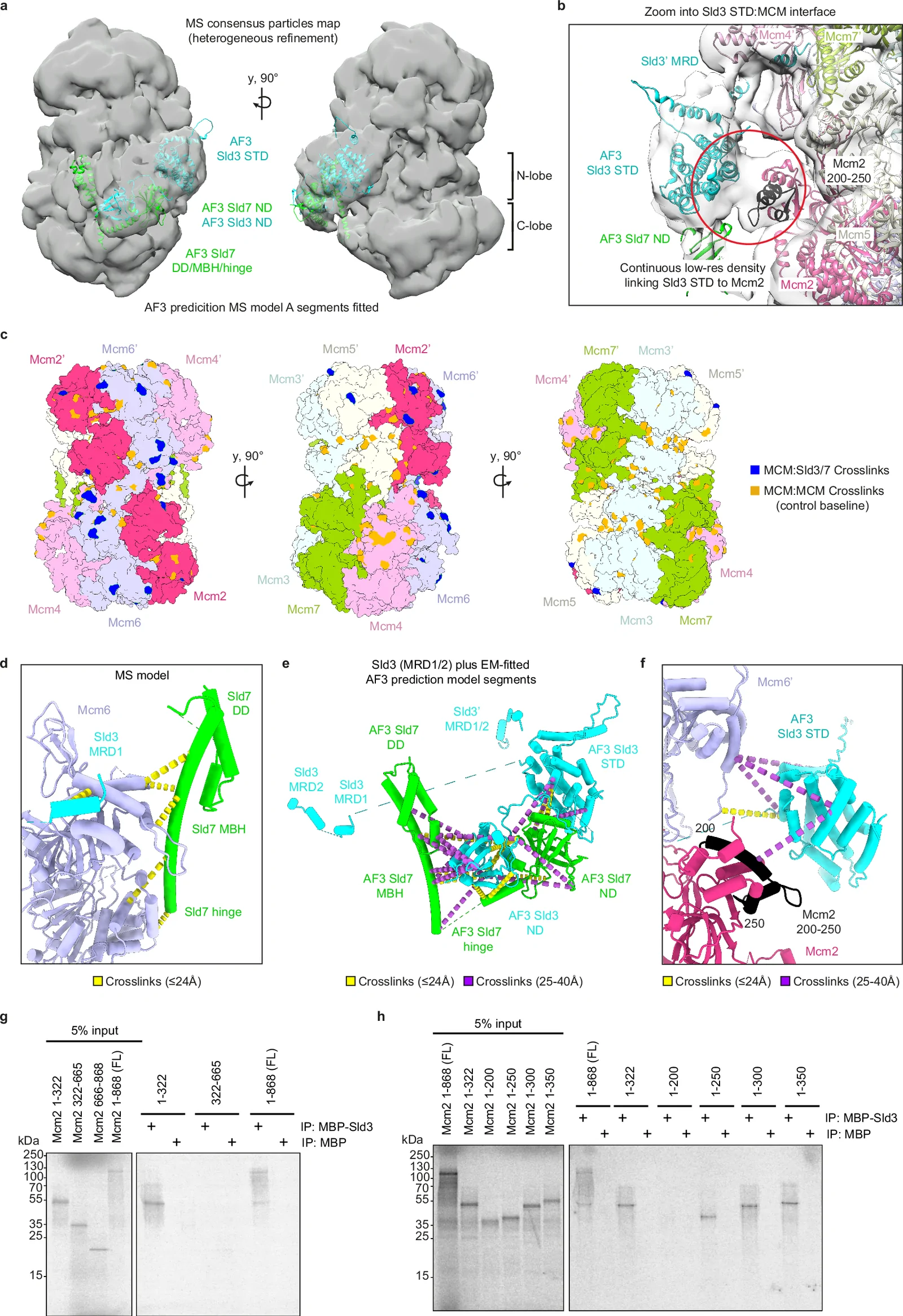
Flexible links between Sld3 and MCM2-7 position Sld3 at the MCM2-7 DH interface. **a** Heterogeneous refined consensus MCM2-7-Sld3/7 (MS) map. The envelope for the Sld3/7 region allows for a fit-in-map of extracted portions of Sld3/7 from an MS AlphaFold3 (AF3) model (Supplementary Fig. 24). **b** Zoom-in map from (**a**) fitted with MCM2-7 DH and Sld3 MRD regions from MS atomic model and AF3 prediction for other Sld3/7 regions. There is a density connecting Sld3 STD to the NTD-A domain of Mcm2. **c** Surface representations of MCM2-7 DH showing all mapped BS3 crosslinks obtained using crosslinking mass spectrometry (XL-MS). MCM:Sld3/7 crosslinks (blue) are predominantly formed between Mcm2/6 and Sld3/7. MCM:MCM crosslinks (orange) mostly occur in structurally resolved regions. **d** A zoomed-in view of the MS atomic model showing crosslinks between Sld7:Mcm6 and Sld3 MRD1:Mcm6 supporting the MS-derived atomic model. **e** XL-MS analysis supports the proposed placement of the Sld3/7 AlphaFold model relative to the DH, based on fitting into the low-resolution EM map shown in (**b**). The stable MCM:Sld3/7 contacts are supported by the data. In contrast, the more flexible Sld3 STD domain is consistent with the data only when a cutoff of ~40 Å is used between Cα atoms, consistent with STD domain flexibility in EM. The orientation of the Sld3 STD face towards the DH agrees with both the EM-fitted models and the crosslinking data. **f** Zoomed view of the Sld3 interaction region with the Mcm2 A-domain. Placement of Sld3/7 N-terminal dimerization domain (ND) was guided by rigid-body fitting into a low-resolution EM map of MS. AF3 predicted placement of the Sld3-Treslin domain (STD). Both are supported by cross-linking mass spectrometry data (**c**). **g** Immunoprecipitation assay mapping the Sld3 interaction region with Mcm2 to the N-terminus (residues 1–322). MBP or MBP-Sld3 were immobilized on beads, used as bait for ^35^S-labeled in vitro transcribed/translated truncations of Mcm2, and analyzed by SDS-PAGE/autoradiography. Data representative of 3 biological repeats. **h** N-terminal truncations help to define the minimal binding region as residues 200–250 (shown in black in (**f**)). Data representative of 3 biological repeats. Source data are provided as a Source Data file.

In order to understand the underlying structure of the MS complex better, we performed heterogeneous refinement of the MS consensus particles, yielding a well-resolved DH core (3.1 Å mean resolution), with the most stably bound Sld3-Sld7 regions visible at higher contour levels. In contrast, additional Sld3-Sld7 density was only apparent when the same consensus map was displayed at lower contour levels, consistent with increased flexibility of these regions (Fig. 5a). At these lower contour levels, we detected densities for the Sld3 MRDs and the Sld7 DD/MBH/hinge domains on both hexamers, as well as an additional diffuse, globular density in the center of the DH. Notably, continuous density was visible connecting this additional density to the N-terminal domain of Mcm2, suggesting a flexible yet directional linkage between Sld3/7 and the MCM2-7 DH (Fig. 5b). Furthermore, we docked AlphaFold model segments corresponding to the Sld7 DD/MBH/hinge, Sld3-Sld7 ND and Sld3 STD domains into the density, generating a good fit between structure and density (Fig. 5a, b, Supplementary Table 4). To verify this model, we performed crosslink mass spectrometry (Fig. 5c, Supplementary Fig. 13). Because we used BS3 as a crosslinker, we observed crosslinks with minimal flexibility (<24 Å), which were also the most frequent (Supplementary Fig. 13e; yellow). Longer-distance crosslinks (25–40 Å, Supplementary Fig. 13e, purple and >40 Å Supplementary Fig. 13e, gray) highlight the flexibility of Sld3 and Sld7, consistent with our EM analysis (Supplementary Fig. 12e). Still, they may also originate from the fact that crosslinking cannot distinguish between Sld3-Sld7 binding to one or the other hexamer. However, we note that the Sld3-Sld7 crosslinks with the MCM2-7 DH occur predominantly on one face of the DH, in proximity to Mcm2/6/4 (Fig. 5c).

To further validate the MS consensus map, we plotted the more stringent crosslinks (<40 Å) onto the MS atomic model and the docked AlphaFold model segments (Fig. 5d, e, Supplementary Fig. 13c–g). This confirmed several interactions resolved by cryo-EM, including the Sld7 interaction with Mcm6 and the Sld3 MRD contacts with Mcm4 and Mcm6. In addition, crosslinks mapped onto the AlphaFold model segments fitted into the lower-resolution Sld3-Sld7 density supported the placement of these flexible regions and highlighted the dynamic nature of the Sld3-Sld7 complex. Although many crosslinks were consistent with the docked model, several crosslinks were longer than 25 Å (Supplementary Fig. 13f, g), corroborating the conformational flexibility inferred from the EM data. The crosslinking analysis suggests that the Sld7 DD/MBH/hinge domains are in proximity to the Sld3 ND and Mcm6. To understand why these interactions are flexible, we generated several AlphaFold models of Sld7 (Supplementary Fig. 4f–i). We observed that AlphaFold predicted the structure of the Sld3 DD and ND domains very well. Interestingly, when looking at the ND and DD-associated helices and their associated linker, the AlphaFold models varied in their predictions of the angle and orientation between the two helices and their associated Sld3 ND and DD domains. The short linker between the helices is flexible, suggesting that this segment forms a hinge. Thus, the data are consistent with Sld7 having a flexible hinge (Supplementary Fig. 4h, i), which could explain some of the underlying complex dynamics.

Moreover, the crosslinking data suggest that the Sld3 STD is in proximity to the Mcm2 A-domain (Fig. 5f). To verify this model, we performed an immunoprecipitation (IP) assay using in vitro-transcribed and translated Mcm2 truncations as prey and Sld3 as bait. We observed a specific interaction between Sld3 and the N-terminus of Mcm2 (residues 1–322, Fig. 5g). Additionally, fine mapping of the interaction revealed that all C-terminal truncations, excluding 1–200 (Fig. 5h), and all N-terminal truncations analyzed can bind MBP-Sld3, indicating that amino acids 200–250 of Mcm2 constitute an important region (Supplementary Fig. 14). Based on our AlphaFold data, we suggest that these amino acids have a structural role in keeping the domain correctly organized. In summary, the Sld3 STD interaction with Mcm2 fits with our overall complex organization and previous observation using yeast two-hybrid assays^40^, but their lower resolution suggests that the interaction is more dynamic. Overall, we suggest that Sld3-Sld7 are tightly tethered to MCM2-7 via the Mcm4/Mcm6 MRD motifs and the long Sld7 helix (MBH) and that the Mcm2 A-domain makes contacts with the Sld3 STD. Finally, the data highlight dynamics between the Sld7 N-terminal and Sld7 dimerization domains, which are flexibly linked within Sld7, allowing them to explore various conformations.

### The structure of MCM2-7 DH-Sld3-Sld7-Cdc45 (MSC) complex

**Fig. 6:**
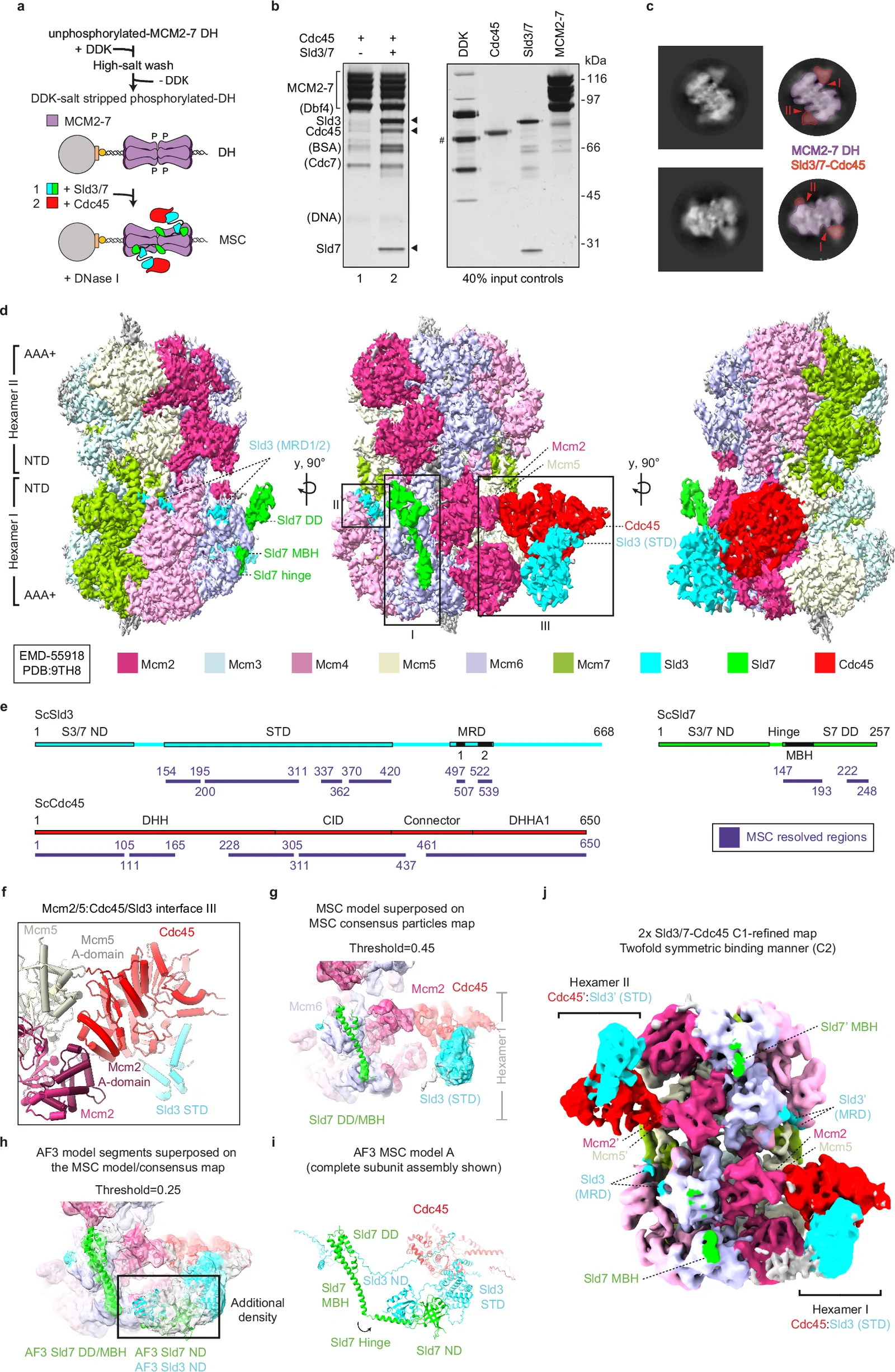
The Sld3 STD relocates to the MCM C-terminal lobe and stabilizes Cdc45-Mcm2/5 A-domain interactions. **a** Assembly of MCM2-7 DH-Sld3/7-Cdc45 complex. **b** Silver-stained SDS-PAGE of Sld3/7-dependent Cdc45 binding to the DDK-phosphorylated MCM2-7 DH. # contaminant (heat shock protein Ssa1) from DDK purification. Data representative of 3 biological repeats. **c** Cryo-EM 2D class averages of the MSC complex. Two molecules of Sld3/7-Cdc45 (I and II, red) bound to the DH, with one density better resolved. **d** A composite map of the MSC complex bound to dsDNA, resolved at a mean resolution of 3.6 Å. The map features one copy of Sld3/7-Cdc45. MCM:Sld3/7-Cdc45 interaction-interfaces I-III: I) Sld7 MCM binding helix (MBH) binds Mcm6, II) Sld3 MCM recognition domain (MRD) binds Mcm4 and Mcm6, and III) the Sld3-Treslin domain, located at the C-lobe of the DH, forms contacts with Cdc45 and allows Cdc45 to contact the NTD-A-domains of Mcm2 and Mcm5. **e** Domain organization and features of Sld3, Sld7 and Cdc45. Cdc45 domains: N-terminal Aspartate-Histidine-Histidine motif (DHH), CMG/C-terminal Interacting Domain (CID), connector/RecJ-like domain, and C-terminal DHH-Associated Domain from subfamily 1 (DHHA1). For Sld3/7: the Sld3/7 N-terminal dimerization (S3/7 ND) domain, Sld3-Treslin domain (STD), MCM recognition domain (MRD), Sld7 MCM binding helix (MBH), and Sld7 dimerization domain (S7 DD). Purple bar indicates regions resolved in the MSC atomic model. **f** Close-up view of the Mcm2/5:Cdc45 interface (interaction interface III in (**d**)). Cdc45 contacts only the NTD-A domains of Mcm2/5 and is tethered via Sld3 STD (cyan). **g** The MSC consensus map, consisting of all particles prior to focused processing and used to generate the composite map in (**d**), reveals unassigned density at the C-lobe of the MCM2-7 DH that extends from the Sld7 MBH/hinge to the Sld3 STD. **h** The unassigned density positions the remaining globular density of the Sld3/7 ND. AlphaFold3 (AF3) predictions of the Sld3/7 ND and Sld7 DD/MBH/hinge were fitted into the EM map. Sld3/7 were extracted from the AF3 MSC model A prediction (Supplementary Fig. 24). **i** AF3 MSC prediction. **j** The C1-refined map, consisting of two molecules of Sld3/7-Cdc45 bound to the DH, exhibits C2 symmetry features. Source data are provided as a Source Data file.

The MS structure has revealed how Sld3-Sld7 reads the DDK activation signal in the MCM2-7 DH. However, it also raises the question of how Sld3 can recruit Cdc45, as the only known Cdc45-binding site within the CMG is far from the main Sld3 density. To address this question, we reconstituted the MCM2-7 DH-Sld3-Sld7-Cdc45 (MSC) complex (Fig. 6a). We found that Cdc45 recruitment was Sld3-Sld7 dependent (Fig. 6b). 2D class analysis of the MSC complex revealed density spanning from the N- toward the C-terminal face of the MCM2-7 ring (Fig. 6c), highlighting a major reorganization, considering that in the MS complex Sld3-Sld7 were found dominantly at the center of the MCM2-7 DH interface (Fig. 2d). Moreover, these densities can be seen at both hexamers, indicating that at least in part, both MCM2-7 hexamers participate in complex assembly. We obtained a 3.6 Å mean resolution map of the MSC complex with a local resolution range of 2.5–8.0 Å (Fig. 6d, e, Supplementary Figs. 15–20, Supplementary Tables 1–3 and Supplementary Movie 1). The MSC model was built using a composite map generated from focused refinements of locally resolved regions, thereby improving the interpretation of peripheral Sld3-Sld7 and Cdc45 densities. In the MSC map, the peripheral Sld3 MRD and Sld7 contact densities were interpreted conservatively as backbone level assignments, guided by local density, AlphaFold3 predictions and prior biochemical constraints. These densities support the overall placement of the motifs, but do not justify high-resolution or sequence register certainty, particularly for the Sld3 MRDs. Cdc45 is seen bridging the Mcm2/Mcm5 interface and is bound by the Sld3 STD. In addition, we observed the Sld7 DD/MBH/hinge domains and the Sld3 MRD in contact with Mcm4 and Mcm6 (Fig. 6d, e). In the MSC complex, the DNA spans the entire channel and is double-stranded throughout (Supplementary Figs. 15e, 17b). Interestingly, Sld3 STD and Cdc45 are attached flexibly to MCM2-7, with only the Mcm2/5 NTD/A-domain interface serving as a direct fixed connection point (Fig. 6f). Accordingly, we can observe conformational dynamics in Sld3 and Cdc45 (Supplementary Fig. 21).

### Cdc45, as part of the MSC, is only partially associated with MCM2-7

The Cdc45 protein was resolved at a local resolution of 4–7 Å, allowing us to build the majority of the protein. The highly conserved Cdc45 CMG Interaction Domain (CID, Supplementary Figs. 22, 23) contacts both Mcm2 and Mcm5 N-terminal A-domains (Fig. 6f). This is markedly different from Cdc45 contacts within the CMG^37^, where Cdc45 also makes extensive contacts with the Mcm2 and Mcm5 CTDs. Comparing the contacts between Cdc45 and Mcm2 and Mcm5 in the MSC, it is clear that Cdc45 makes much more extensive contacts with Mcm5 than Mcm2. Interactions with Mcm2 are mainly backbone-mediated, whereas the Mcm5-Cdc45 interface involves side-chain interactions. This is different from the CMG^37^, where contacts are nearly evenly split between Mcm2 and Mcm5. Thus, we conclude that Cdc45 within the MSC represents a partially associated state, with the majority of its contacts occurring with the NTD of Mcm5. We suggest that subsequent GINS binding stabilizes Cdc45 on MCM2-7 and promotes the more extensive Cdc45-MCM2-7 contacts observed in the CMG.

### Low-resolution model of MSC

Our composite MSC map at a high threshold resolved the Sld7 DD/MBH/hinge and the Sld3 MRD/STD (Fig. 6d). Lowering the display threshold revealed additional, lower-resolution density connecting Sld7 to the Sld3 STD. Prior to focused classification and refinement of individual regions, homogeneous refinement of the MSC consensus particles yielded a more continuous envelope, enabling better visualization of these flexible regions (Fig. 6g, h). An AlphaFold model of the Sld3-Sld7 ND (Supplementary Fig. 24) could be fitted into this additional density (Fig. 6g–i), suggesting the location of the Sld7 hinge, which appears to flexibly link Sld7 to both Sld3 and Cdc45. In addition to this, a separate C1-refined map derived from MSC consensus particles revealed an approximately C2-symmetric assembly, with Sld3-Sld7-Cdc45 densities visible on both MCM2-7 hexamers (Fig. 6j, Supplementary Fig. 20). Although this map was lower in resolution, the two Sld3-Sld7-Cdc45 modules occupied equivalent positions, supporting the idea that the same interaction architecture can form on both sides of the DH. Together, these observations indicate that Sld3-Sld7 can correctly position Cdc45 at the distal Mcm2/Mcm5 interface. This suggests that the less well-resolved domains represent a flexible linkage that enables Sld3 to bridge distal regions of MCM2-7 and Cdc45.

### The Sld3-Cdc45 interface

**Fig. 7:**
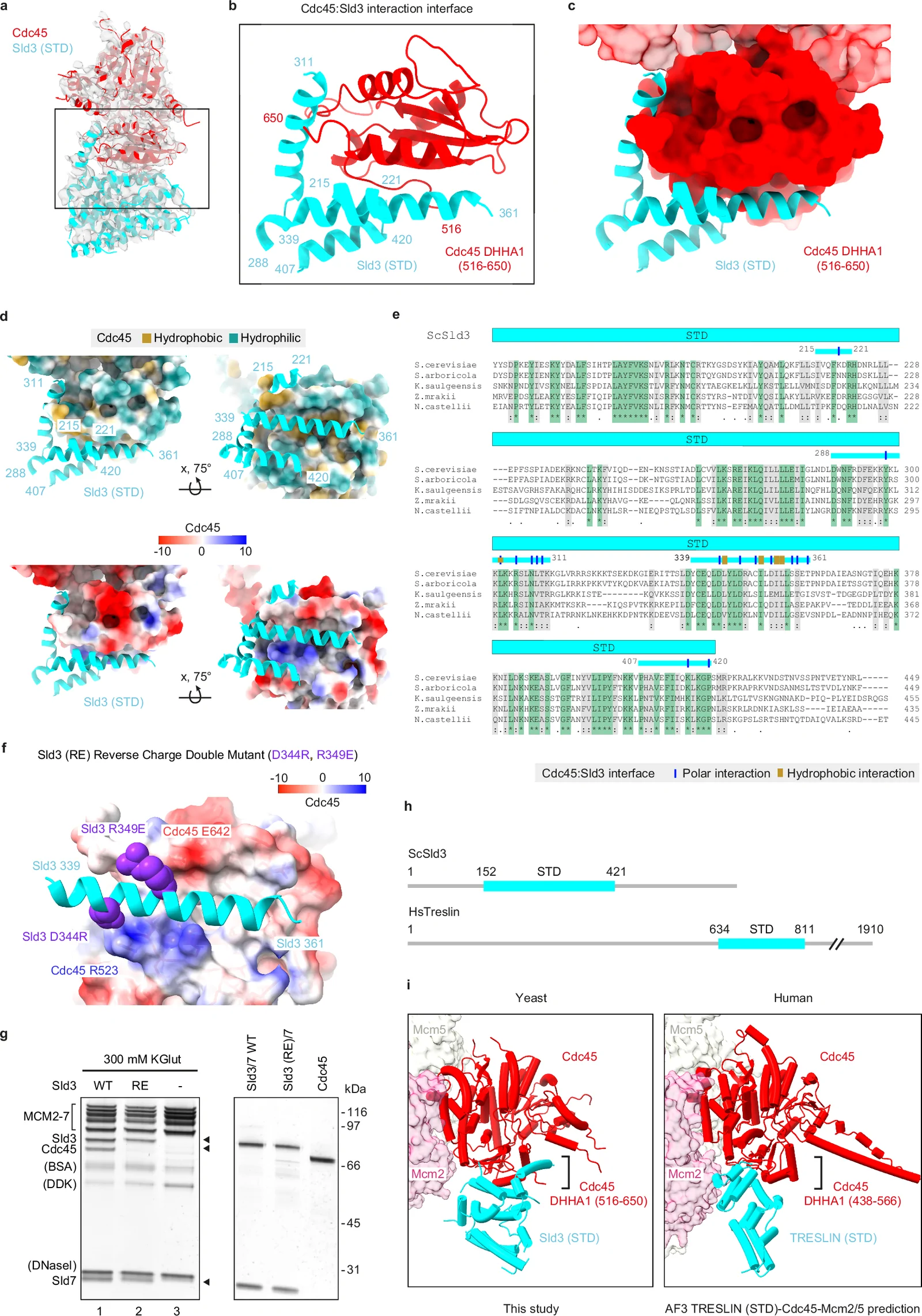
Analysis of the Cdc45:Sld3 STD (Sld3-Treslin domain) interaction interface. **a** A comparison of the composite MSC map (gray) quality against the derived atomic model for Cdc45-Sld3 STD (red and blue). **b** Zoomed-in view of the Cdc45:Sld3 STD interface. The regions of Sld3 involved in binding the C-terminal DHH-Associated Domain from the subfamily 1 (DHHA1) domain of Cdc45 are shown. **c** Surface representation of Cdc45 and cartoon-ribbon representation of Sld3 STD helices involved in interactions. **d** Cdc45 surface colored by hydrophobicity and electrostatic potential. The scale bar indicates the Coulombic electrostatic potential, measured in kcal/(mol·e) at 298 K. The Cdc45:Sld3 interface includes a hydrophobic cleft contacted by an Sld3 helix (residues 339–361) and complemented by multiple polar interactions with Sld3. **e** Multiple sequence alignment of yeast homologs, with Sld3 STD interacting regions at the Cdc45 interface plotted. The Sld3 339-361 region confers most of the contacts with Cdc45. ScSld3 domain boundaries are indicated above the sequence. Highly conserved residues are colored with a vertical green line and indicated by (*), highly similar residues are indicated by (:) and a vertical gray line, and residues with similar properties are indicated by (.). Hydrophobic contacts are indicated by a vertical gold bar and polar contacts by a vertical blue bar. **f** Rationale and mutant design to disrupt Cdc45:Sld3 binding based on the atomic model of MSC. **g** Silver-stained SDS-PAGE analysis to assess the Sld3 reverse charge double mutant (RE) for Cdc45 recruitment to MCM2-7 DH. Data representative of 2 biological repeats. **h** The STD of Sld3 is homologous to the STD of the human TRESLIN/TICRR. The 2D domain organization of Sld3 and TRESLIN is shown, highlighting the position of the STD domain. **i** Comparison of the Sld3 STD-Cdc45 region resolved in this study with an AlphaFold3 (AF3) server prediction of human TRESLIN STD-Cdc45-Mcm2/5. In both models, the STD domain recruits Cdc45 to the A-domains of Mcm2/5 through interactions with the DHHA1 domain of Cdc45, suggesting that the STD domains in yeast and human function similarly to tether Cdc45 to the C-lobe of the MCM2-7 DH. Source data are provided as a Source Data file.

Within the MSC complex, we discovered that the Sld3 STD specifically interacts with Cdc45 (Fig. 7a–c). This interface is formed primarily by three helices of the Sld3 STD and the Cdc45 DHHA1 domain (Supplementary Fig. 1a, Fig. 7b), and is stabilized by shape complementarity alongside hydrophobic, polar, and electrostatic interactions (Fig. 7d). Many of the amino acids that form the interface are conserved within fungi (Fig. 7e, Supplementary Figs. 11a, 22). One helix of Sld3 makes the most extensive contacts, including ionic interactions between Sld3 R349 and Cdc45 E642, and Sld3 D344 and Cdc45 R523 (Fig. 7e, f). To address whether these residues in Sld3 are indeed functionally relevant, we generated the double mutant, which we term Sld3/7 RE (Fig. 7f). We observed that this mutant could be recruited to the MCM2-7 DH, indicating that Sld3-Sld7 recruitment remains functional, but it failed to recruit Cdc45 (Fig. 7g, compare lanes 1 and 2). Moreover, our structure showed that the Sld3 helix 288-311 interacts with the Cdc45 DHHA1 domain (Fig. 7d), and prior mutagenesis data corroborate this interaction. Interestingly, Deegan et al. found that point mutants at Sld3 aa296/297/299/301 (Sld3-4E) and aa303/304/305 (Sld3-3E) disrupted the Sld3-Cdc45 interaction, while the Sld3-3E mutant also affected DNA synthesis^11^, further validating the interface. Although Treslin is the human functional equivalent of Sld3, the two proteins are poorly conserved at the amino acid sequence level. Nevertheless, AlphaFold predicts a similar overall organization when the Treslin STD is modeled in complex with Cdc45 and Mcm2/Mcm5 (Fig. 7h-i; Supplementary Table 4), indicating that the general principle could be conserved in higher eukaryotes, especially as Cdc45 shows high levels of structural similarity between budding yeast and humans (Supplementary Fig. 23).

### Sld3 flexibility couples Mcm4/Mcm6 recognition with Cdc45 recruitment to Mcm2/5

**Fig. 8:**
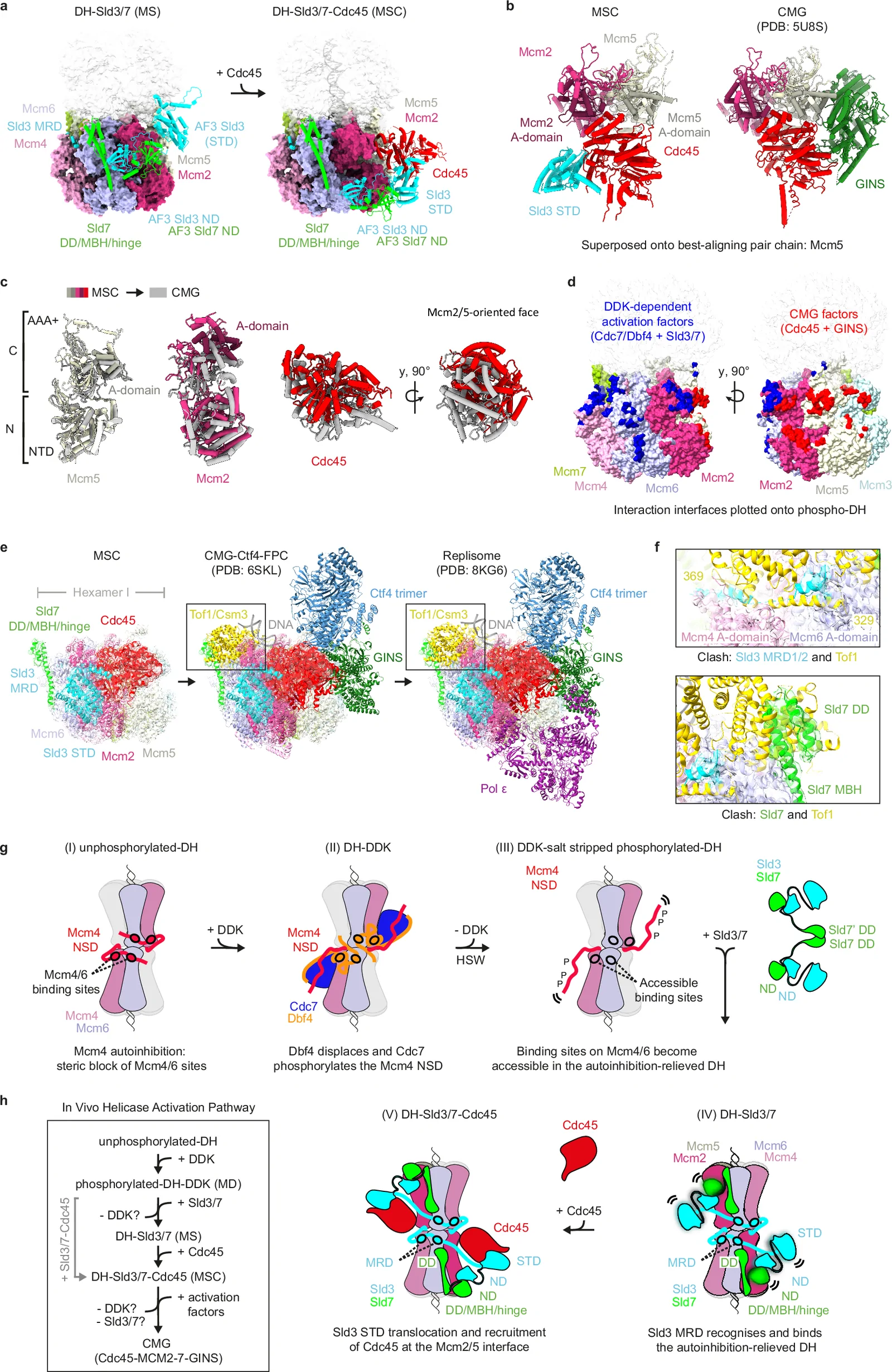
Sld3/7 acts as the phospho-MCM reader on a reused surface of MCM2-7 and recruits Cdc45 to the NTD-A-domains of Mcm2/5 ready for GINS hand-over and CMG assembly. **a** A comparison of the large structural changes that occur from MS to MSC upon the recruitment of Cdc45. Notably, the Sld3 STD domain, localized at the NTD-A of Mcm2, is repositioned from the center of the DH towards the CTD of Mcm2, allowing the recruitment of Cdc45 at the NTD-A-domains of Mcm2/5. Sld3 MRD and Sld7 MBH anchor the Sld3/7 complex, whilst Sld3/7 ND and Sld3 STD display large flexible conformations. **b**,** c** Comparison of the structural changes that occur at Mcm2/5:Cdc45 from MSC to CMG. In both MSC or CMG configurations, Cdc45 is always held in place at the Mcm2/5 interface, either by Sld3 STD or GINS, respectively, suggesting a cooperative hand-over mechanism. The differences between the two structural states are vast; the most significant is a rearrangement of the NTD-A domain of Mcm2 and a larger interaction interface formed between Mcm2/5 and Cdc45. **d** The MCM2-7 DH is engaged by activation factors at two functionally distinct faces, Mcm4/6/2 for DH activation and Mcm2/5/3 for CMG assembly. Interface residues were mapped by projecting all ≤4 Å contacts from superposed complexes containing DDK, Sld3/7, Cdc45, or GINS onto the surface of phosphorylated MCM2-7 DH. **e** An overview of structural snapshots from MSC to the full replisome. The CMG-Ctf4-Fork protection complex (PDB: 6SKL) and the replisome (PDB: 8KG6) both feature multiple components that do not present major structural clashes compared to MSC, except Tof1. **f** Zoomed view of steric clashes involving Tof1 with Sld3 MRD1/2 and Sld7 DD. **g** Schematic illustration summarizing key intermediate steps (I–V) from MCM2-7 DH to MSC involved in the conversion of the inactive helicase into the active CMG during DNA replication initiation. **h** Overview of step-wise helicase activation pathway intermediates that occur in vivo.

Interestingly, within the MS complex, Sld3 engages the N-terminal regions of Mcm2, Mcm4, and Mcm6 (Fig. 8a). This fits with the narrative that Sld3 needs to read-out the DDK phosphorylation status of MCM2-7. However, Cdc45 is located distal to the Sld3-Sld7 binding sites within the MS complex. This raised the question of how Sld3 could recruit Cdc45? A clue was derived from the relatively high mobility of the Sld3 STD, which becomes repositioned in the context of the MSC complex (Fig. 8a, Supplementary Movie 3). In the MS complex, the Sld3 STD surface that contacts Cdc45 also interacts with Mcm2. Thus, the data suggest that binding of Cdc45 breaks the weak Mcm2 A-domain and Sld3 STD interaction and, consequently, allows the establishment of the distal Cdc45-Mcm2-Mcm5 interface, where the Sld3 STD now interacts with Cdc45. Moreover, the Sld7 hinge (Supplementary Fig. 4f–i) indicates that Sld7 helps facilitate the proper positioning of the Sld3 STD domain within the MS and MSC complexes. This may explain why Sld7 supports Sld3 function but is not essential^30^. In addition, by applying 3D Flexible Refinement (3DFlex)^41^ to a focused subset of MSC particles in which Cdc45 is attached at the Mcm2/5 interface, we could visualize a series of conformational snapshots capturing the flexible motions of Sld3 STD and Cdc45 that underpin Cdc45 tethering (Supplementary Fig. 21, Supplementary Movie 4).

### Structural changes upon MSC to CMG transition

Within the MSC, Cdc45 makes only specific contacts with the Mcm2 NTD/A-domain, whereas in the CMG, Cdc45 makes extensive contacts with the Mcm2/Mcm5 NTD/A-domain and the CTD (Fig. 8b). This change originates from GINS pulling Cdc45 closer to its side, together with significant structural changes in Mcm5 (Fig. 8b). Indeed, while an alignment of MSC and CMG Mcm2 shows only minor changes, Mcm5 undergoes significant conformational changes, notably a rotation of its NTD relative to its CTD. Alongside this, Cdc45 also becomes largely restructured, especially along the Mcm2/Mcm5 interface (Fig. 8c). Thus, the data show that during the MSC-to-CMG transition, a major reorganization is occurring in Mcm5 and Cdc45. Together, these changes suggest that ATP-hydrolysis-dependent CMG assembly transitions Cdc45 from a partially associated state in the MSC to a stably integrated component of the replication fork^7^.

Since ATP hydrolysis is important for CMG formation^7^, we compared the MCM2-7 nucleotide states from the MCM2-7 DH to the CMG (Supplementary Fig. 25). During the transition from the MCM2-7 DH to the DDK-salt stripped phospho-DH, changes occur at the Mcm6/2 interface with ADP initially present in the MCM2-7 DH, and ATP only present after DDK action (Supplementary Fig. 25)^28^. In MS and MSC, the Mcm6/2 interface transitions back to ADP, suggesting a potential Sld3-Sld7-dependent ATP hydrolysis event. Moreover, during the MS-to-MSC transition, no significant change in nucleotide occupancy or status occurs, which is consistent with the observation that Cdc45 makes only relatively weak contacts with MCM2-7 (Fig. 6f). Finally, in the CMG, several changes occur: ATP is now present at the Mcm6/2, 2/5 and 5/3 interfaces, while all other Mcm interfaces remain empty^42^ (Supplementary Fig. 25g). Thus, only once GINS joins a larger ATP hydrolysis event occurs^6^, coinciding with domain rotation in Mcm5, which allows establishment of a much stronger Mcm2/Mcm5 interface with Cdc45 (Fig. 8b).

### The MCM2-7 DH has two faces for DDK-dependent helicase activation factors and the CMG

We note that the DDK-dependent steps in helicase activation, which involve Dbf4, Cdc7, Sld3, and Sld7, all contact one face of the MCM2-7 hexamer, namely Mcm4, Mcm6, and Mcm2 (Fig. 8d, left). In comparison, within the active helicase, the CMG complex, Cdc45 and GINS contact adjacent proteins, particularly Mcm2, Mcm5, and Mcm3 (Fig. 8d, right). We suggest that this organization physically separates DDK-dependent helicase activation processes from CDK-dependent GINS binding to avoid competition between these distinct steps.

Having all DDK-dependent steps occur on the same surface results in regulatory benefits. Indeed, comparison of the DDK, MS, and MSC structures indicates that DDK and Sld3-Sld7 use overlapping MCM2-7 surfaces (Fig. 3e, f). In MS, Sld3-Sld7 occupies the Mcm4/Mcm6 sites contacted by Dbf4 motif M, while the Sld3 STD engages the Mcm2 N-terminal A-domain region that contributes to Dbf4 docking via the Helix-BRCA1 C-terminal (HBRCT) domain. This means that it is initially difficult for Sld3 to become recruited. That said, it has been reported that Sld3 can bind directly to DBf4^40^, providing an additional anchor point. Once Sld3 has made firm contact with MCM2-7, the competing interactions suggests that Sld3 promotes DDK release (Fig. 3f and 5d–f). In MSC, the Mcm2 site is released from the Sld3 STD and could become available again or even blocked by Dbf4, but Cdc45 now occupies the Mcm2/Mcm5 interface and would therefore clash with DDK in its DH-bound configuration (Fig. 6d, f). In summary, Sld3-Sld7 and Cdc45 may remodel or displace DDK contacts with the MCM2-7 DH. That said, these models require further experimental validation.

Interestingly, when searching for potential clashes between MSC, CMG as part of the fork protection complex^43^ and the replisome^44^, we observed that Tof1/Csm3 overlaps with Sld3-Sld7 (Fig. 8e). In particular, it clashes with the Sld3 MRD1 and MRD2 and the Sld7 DD (Fig. 8f). Previously, a clash between Tof1 and DDK has been reported as well^29^. Since Tof1 associates with the CMG only after MCM10 fully activates the helicase motor, this may indicate that Tof1 could release Sld3 relatively late in replisome assembly. That said, we acknowledge that we have still only a limited understanding of the overall process, and it remains unknown whether Tof1 or other factors influence Sld3-Sld7 release or helicase activation.

### Summary

Here, we resolved the structures of the MCM2-7 DH in a DDK-phosphorylated state (Fig. 1), MCM2-7 DH-Sld3-Sld7 (MS, Fig. 2), and MCM2-7 DH-Sld3-Sld7-Cdc45 (MSC, Fig. 6). These structures, together with reinterpretation of previously published structures^28,29^, explain how DDK-dependent helicase activation is read out by Sld3 and detail how Sld3-Sld7 promote Cdc45 deposition on the Mcm2/Mcm5 interface.

Within the inactive unphosphorylated MCM2-7 DH, the key Sld3 interaction surface formed by Mcm4 and Mcm6 is occupied by the N-terminal NSD tail of Mcm4 (Fig. 8g, step I). This domain becomes displaced upon DDK-dependent phosphorylation (Fig. 8g, steps II-III). Indeed, the Dbf4 motif M contacts the same sites as the Mcm4 NSD, which could potentially help DDK to release the Mcm4 autoinhibitory tail and phosphorylate it (Fig. 8g, step II). In the DDK salt-stripped phospho-MCM2-7 DH, Mcm4 and Mcm6 become accessible to Sld3, and indeed, we identified two short stretches of Sld3 that contact the initially autoinhibited region (Fig. 8g, step III–IV). Thus, Sld3 can read out the DDK phosphorylation status in this way. Crucially, Deegan et al. showed that mutations in MRD2 disrupt DDK-dependent Sld3-Sld7 recruitment, highlighting that the MRD motif is a central point of recruitment^11^. Deegan et al. also found that a second region (aa251-331) was necessary for the Sld3-Sld7 interaction with MCM2-7^11^. Here, we showed by EM and crosslinking mass spectrometry that this region of Sld3 (aa251-331) is in proximity with Mcm2 (Fig. 5f), and validated the interaction between this region and Mcm2 by IP (Fig. 5h). The Mcm2 interaction with Sld3 is also consistent with prior yeast two-hybrid work and pulldown assays^40,45^. Thus, our data demonstrate that the Sld3 MRD1/2 motifs can read out the DDK phosphorylation status of MCM2-7 and provide strong evidence that the Sld3 STD interacts with Mcm2 in the context of MS. Sld7, on the other hand, contacts the interface between Mcm2 and Mcm6 (Fig. 2g). Since this interaction is not targeted by DDK, its binding is likely phosphorylation-independent. Interestingly, we found that the (Sld3-Sld7)^2^ complex exists in solution (Supplementary Fig. 6) and that, within the MS complex, it is separated upon engagement with the DH (Fig. 2i). We also found that Sld3-Sld7 exhibits strong dynamics in complex with the MCM2-7 DH (Supplementary Movie 2). We suggest that Sld3-Sld7 flexibility supports conformational changes when the Sld3 STD is repositioned from its Mcm2 interaction site to the distal site—where the Sld3 STD interacts with Cdc45 and consequently deposits Cdc45 at its binding site at the Mcm2/Mcm5 interface (Figs. 8a, g, steps IV–V). Thus, the data highlight that Sld3-Sld7 have a flexible structure, which allows Sld3-Sld7 to read out the DDK phosphorylation status at Mcm4/Mcm6 and at the same time deposit Cdc45 at a distal site. A comparative analysis of replication complex structures suggests that interactions between DDK and MCM2-7 become destabilized during MS and MSC formation, while Sld3-Sld7 is likely released during later stages of helicase activation. Tof1 is a potential candidate for Sld3-Sld7 release, as it would clash with these factors (Fig. 8e), but we note that experimental evidence is missing. Finally, our AlphaFold prediction of the Treslin (human Sld3 homolog, Fig. 7h) in complex with Cdc45 and Mcm2/Mcm5 (Fig. 7i) raises the possibility that the Treslin STD could recruit Cdc45 to the MCM2-7 DH in a manner similar to the yeast Sld3 STD. Whether the human Sld7 homolog, MTBP, functions structurally similarly to yeast Sld7 is currently unknown; however, it has been shown that the Sld7 regions involved in Sld3 binding and Sld7 self-interaction exhibit greater sequence conservation^46,47^.

We acknowledge that our stepwise analysis of DDK, Sld3-Sld7, and Cdc45 will not fully reflect the in vivo situation. Specifically, DDK is unlikely to be released before Sld3 addition (Fig. 8h). For biochemical and structural analyses, DDK needed to be removed to limit aggregation. Also, in cells, Sld3-Sld7-Cdc45 could likely form a complex, which would speed up the Cdc45 deposition process (Fig. 8h). Nevertheless, since our results are broadly consistent with biochemical and in vivo data^7,11,12,30^, we feel they provide a valuable reference for the field. Moreover, only by breaking the reaction down into individual steps can we understand the contributions of each factor.

## Methods

### Purification of ORC

ORC was expressed and purified from Hi-5 cells (Invitrogen) as described^48^. Cells were grown adherently and infected for 2 h with baculoviruses gifted by Bruce Stillman (CSHL) (Orc1,6 MOI 20; Orc2,5 MOI 15; Orc3,4, MOI 10) before serum was added to the media. After 48 h, cells were pelleted via centrifugation (10 mins, 4 °C, 270 x* g*). Cold PBS was used to wash pellets before again centrifuging and resuspension in HYPO buffer (20 mM HEPES pH 7.5, 5 mM KCl, 1.5 mM MgCl_2_, 1 mM DTT, 1x complete protease inhibitor (CIP) without EDTA (Roche)). Cells were dounced and nuclei collected by a further centrifugation step (20 min, 4 °C, 25,000 x* g*, no brake). Nuclei were homogenized by douncing in H/0.2 buffer (50 mM HEPES pH 7.5, 0.2 M KCl, 5 mM magnesium acetate, 1 mM EDTA, 1 mM EGTA, 0.02% NP40, 1 mM DTT) with 1x CIP without EDTA. Ammonium sulfate precipitation (12.5%/45%) was used to extract ORC. The 45% pellet was resuspended in H/0.2 buffer with 1x CIP without EDTA, 0.1 mM ATP, 5 mM MgCl_2_, 14.3 mM b-Mercaptoethanol (H.0.2*). The same buffer without KCl (H/0*) was added, and the samples were incubation for 20 mins, with intermittent vortexing. Dissolved pellets were centrifuged (15 mins, 4 °C, 27,000 x* g*) and the resultant supernatant was loaded onto an SP Sepharose column (20 ml HR16/60, GE Healthcare) and gradient eluted (H/0.2 – H/0.6 KCl, with 14.3 mM b-Mercaptoethanol). 0.8 volume of H/0 was used to dilute the protein before centrifugation (15 mins, 4 °C, 27,000 x* g*). Supernatant was loaded onto a Mono Q 5/50 GL column (GE Healthcare) and eluted (H/0.2* – H/0.5*). Fractions were pooled before dilution with H/0* plus 1x CIP without EDTA and bound to SP-Sepharose resin overnight. H/0.6* with 1x CIP without EDTA was used to elute ORC. Protein was then loaded onto a Superdex 200 16/60 column (GE Healthcare) equilibrated with H/0.2 with 14.3 mM b-Mercaptoethanol. Protein was concentrated by binding to SP-Sepharose overnight and eluted with H/0.6 with 14.3 mM b-Mercaptoethanol. Protein was diluted to the required concentration (~1.6 mg/ml) using H/0.2 buffer, and glycerol was added to a final concentration of 10%.

### Purification of Cdt1

Cdt1 (pCS178) was expressed in E. coli BL21 CodonPlus RIL (Agilent) and purified as described^2^, except the final gel filtration step was omitted. Briefly, cultures (0.9 OD600) were induced with 0.5 mM IPTG for 5 h at 16 °C with shaking. Collected pellets were sonicated in buffer (50 mM Tris-HCl, pH 7.2, 150 mM NaCl, 5 mM MgCl_2_, 1% Triton (v/v) X-100, 1 mM DTT and 1x CIP with EDTA) containing lysozyme. Supernatant was incubated with glutathione-agarose (Sigma) at 4 °C for 2 h. The resin was rinsed with sonication buffer, washed twice with high salt buffer (50 mM Tris-HCl, pH 7.2, 300 mM NaCl, 5 mM MgCl_2_, 1 mM DTT, 1% (v/v) Triton X-100 and 2x CIP with EDTA), then twice washed and rinsed with sonication buffer without CIP. The GST tag was cleaved using PreScission Protease (Amersham, 1:50 (v/v)) for 2.5 h at 4 °C. Flowthrough was collected and combined with the eluate of three rinses with wash buffer (25 mM Tris-HCl, pH 7.2, 25 mM HEPES KOH, pH 6.0, 75 mM NaCl, 5 mM MgCl_2_, 1 mM DTT). Protein was diluted with 1 volume of buffer (25 mM HEPES KOH pH 6.0, 5 mM MgCl_2_, 1 mM DTT) prior to incubation with SP-Sepharose beads for 30 mins, 4 °C. Cdt1 was eluted drop-by-drop using 25 mM Tris-HCl pH 7.2, 25 mM HEPES KOH pH 6.0, 250 mM NaCl, 5 mM MgCl_2_, 1 mM DTT, 0.1% (v/v) Triton-X100. Protein was diluted to ~0.5 mg/ml using elution buffer plus 15% (v/v) glycerol.

### Purification of Cdc6

Cdc6 (pCS160) was expressed in E. coli BL21 CodonPlus RIL (Agilent) and purified as described^49^ with minor modifications: Cell lysis was performed in sonication buffer (500 mM potassium phosphate, pH 7.6, 150 mM potassium glutamate (KGlu), 5 mM MgCl_2_, 1 mM ATP, 1 mM DTT, 1% (v/v) Triton X-100) with protease inhibitors without EDTA (Sigma, S8830). After incubation with lysate, GST-beads were rinsed with cleavage buffer (sonication buffer with potassium phosphate reduced to 100 mM). Protein eluted from GST-beads was diluted with an equal volume of dilution buffer (5 mM MgCl_2_, 1 mM ATP, 1 mM DTT) to reduce the concentration of potassium phosphate to 50 mM and KGlu to 75 mM. The final elution from Hydroxyapatite beads was performed in 50 mM potassium phosphate, 400 mM KGlu, 5 mM MgCl_2_, 0.1% (v/v) Triton X-100, 10% (v/v) glycerol, 1 mM DTT.

### Expression of MCM2-7, DDK, Cdc45 and Sld3/7

MCM2-7 (YC119), DDK (YC309), Sld3/7 and Cdc45 were transformed into yeast strain AS499 (*MATa. bar1Δ, leu2-3,-112, ura3-52, his3-Δ200, trp1-Δ-63, ade2-1 lys2-801, pep4* – a kind gift from Luis Aragon, MRC-LMS). Yeast cells were grown overnight in selective medium at 30 °C, and proteins were expressed in the presence of galactose^50^. Cells were lysed using a SPEX freezer-mill.

### Purification of MCM2-7

MCM2-7 (HA-tag on Mcm3) was purified as described^2^ with the modifications: A Superose 6 Increase 10/300 column (Cytiva) was used for the final gel filtration step and the gel filtration buffer was supplemented with 5 mM ATP.

### Purification of DDK

MBP-Prescission-Dbf4 and Cdc7 (DDK, pCS313_pESC-TRP MBP-PS-Dbf4-Cdc7) was purified as described^27^. Briefly, cell powder was resuspended with 1 volume of buffer D (50 mM HEPES-KOH pH 7.5, 1 mM EDTA, 1 mM EGTA, 10% (v/v) glycerol, 0.02% (v/v) NP-40, 1 mM DTT) with 400 mM NaCl plus protease inhibitor without EDTA (Sigma). Clarified lysate was incubated with amylose resin (NEB) for 2 h. The resin was then washed with buffer D / 400 mM NaCl, buffer D / 1 M NaCl, and buffer D / 200 mM NaCl, and incubated with lambda phosphatase (NEB) in buffer D / 400 mM NaCl with 1 mM MnCl_2_ for 1 h at 4 °C. The MBP tag was cleaved with PreScission Protease for 2 h, and the eluate was applied to Hitrap Heparin column (Cytiva) and then Superdex 200 column (Cytiva) pre-equilibrated in buffer D / 400 mM NaCl. The fractions containing DDK were pooled and concentrated with an Amicon centrifugal filter with 100 kDa MWCO (Millipore).

### Purification of Cdc45

Cdc45 was cloned into a pESC-HIS backbone (pCS730) with an internal FLAG tag by Genscript and purified based on the method described^7^. Briefly, cell powder was thawed in buffer H (25 mM HEPES-KOH, pH 7.6, 10% (v/v) glycerol, 1 mM EDTA, 1 mM DTT) + 500 mM KOAc. Anti-FLAG M2 affinity gel (Sigma) and protease inhibitors without EDTA were added to clarified extract and incubated for 4 h at 4 °C. Resin was washed in the same buffer in the presence of protease inhibitors without EDTA, followed by buffer P (150 mM KOAc, 10% (v/v) glycerol) + 20 mM K-phosphate, pH 7.4 + protease inhibitors without EDTA. Cdc45 was eluted by incubation with Buffer P + 20 mM K-phosphate + 0.5 mg/ml 3 x Flag peptide for 1 h at 4 °C. The eluate was applied to a Hydroxyapatite (type II) column (Bio-Rad) for 30 mins. The column was washed with buffer P + 80 mM K-phosphate, followed by buffer P + 300 mM K-phosphate. The eluate was dialyzed overnight against buffer H + 300 mM KOAc before concentration using an Amicon centrifugal filter with 50 kDa MWCO (Millipore).

### Sld3/Sld7 purification

Sld3-FLAG/Sld7 was cloned into a pESC-HIS backbone (pCS734 pESC-HIS Sld3-FLAG Sld7, or pCS1387 for the D344R R349E Sld7 mutant) by Genscript and purified based on methods described^7,45^. Briefly, lysed cells were resuspended in buffer H + NP-40 (25 mM HEPES-KOH pH 7.6, 10% (v/v) glycerol, 0.02% (v/v) NP-40, 1 mM EDTA, 1 mM DTT) + 500 mM KCl and protease inhibitors. Extract was clarified by centrifugation prior to the addition of Anti-Flag M2 affinity resin for 1 h at 4 °C. Collected resin was washed with the same buffer, followed by washing with buffer H + NP-40 + 500 mM KCl and 10 mM MgAc, 1 mM ATP and protease inhibitors without EDTA and then again with buffer H + NP-40 + 500 mM KCl and protease inhibitors. Protein was eluted with buffer H + NP-40 + 500 mM KCl and 0.5 mg/ml FLAG peptide for 1 h at 4 °C and then further purified using a Superdex 200 10/300 column (Cytiva) equilibrated with Buffer H + NP-40 + 500 mM KCl. The fractions containing Sld3/Sld7 were pooled and concentrated with an Amicon centrifugal filter with 50 kDa MWCO (Millipore).

### MBP-Sld3 purification

Sld3 was cloned into a pESC-HIS vector with N-terminal MBP tag for pull-down reactions (pCS245) and purified as described^45^. Briefly, lysed cells were resuspended in buffer AS (50 mM HEPES-NaOH, pH 8.1, 500 mM NaCl, 10% (v/v) glycerol, 0.1% (v/v) NP-40, 1 mM DTT plus complete protease inhibitor cocktail (PIC, Roche). Cleared lysate was incubated for 2 h at 4 °C with amylose resin (NEB). Beads were rinsed with 10 CV buffer AS + PIC, then washed twice with 20 CV for 30 mins. Protein was eluted in buffer AS + 10 mM maltose.

### MCM2-7 DH and Sld3/Sld7 or Sld3/Sld7-Cdc45 interaction assay

80 nM ORC, 160 nM Cdc6, 160 nM Cdt1 and 160 nM MCM2-7 were added to 25 μl of preRC buffer + 300 mM KGlu (50 mM HEPES-KOH pH 7.6, 300 mM KGlu, 10 mM MgAc, 0.1 mM ZnAc, 3 mM ATP, 5 mM DTT, 0.1% (v/v) Triton X-100, 10% (v/v) glycerol, 0.05 mM EDTA) supplemented with 0.1 mg/ml BSA. Reactions were incubated on ice for 20 mins, followed by 20 mins at 24 °C. 600 ng of 10x ARS DNA coupled to Dynabeads MyOne Streptavidin T1 beads (Invitrogen) was added for 60 mins at 24 °C with mixing at 900 RPM in an Eppendorf Thermomixer C. The 10x ARS fragment^3^ was produced using pCS735 (pUC57 containing 10× ARS1 248 bp, each flanked by AluI sites) using primers 1693 (GAAGATCAGTTGGGTGCACG) and 1568 (btn-GCATCTTTTACTTTCACCAGCG). The amplified 5-kb DNA was bound to the beads and subsequently digested using HindIII to release the non-origin DNA fragment and maintain ∼3 kb of 10× ARS1 DNA bound to the beads. Beads were washed 3x with 100 μl high-salt buffer (preRC buffer + 100 mM KGlu + 300 mM NaCl) to leave the salt-stable DH bound to beads. After the third wash, beads were rinsed with preRC buffer + 100 mM KGlu and resuspended in 20 μl preRC buffer + 100 mM KGlu. The DH was phosphorylated by the addition of 400 nM DDK for 30 mins at 24 °C, 900 RPM. Beads were then washed 2x with stripping buffer (preRC buffer + 100 mM KGlu + 500 mM NaCl) to remove DDK, followed by a rinse with preRC buffer + 100 mM KGlu. 25 μl of preRC buffer + 100 mM KGlu containing 0.1 mg/ml BSA for MS and MSC or preRC buffer + 300 mM KGlu containing 0.1 mg/ml BSA for mutant analysis was added to each tube. For MS, 400 nM Sld3/Sld7 was added and for MSC 400 nM Sld3/Sld7 + 400 nM Cdc45, for 10 mins at 24 °C, 900 RPM. Reactions were washed twice with high-salt buffer for MS and preRC buffer + 100 mM KGlu or 300 mM KGlu for MSC before elution using DNase I.

### Sld3 pulldown reactions

In vitro transcription and translation and pull-down reactions were performed as described^45^. ^35^S-Met-labeled proteins were generated by amplifying the coding regions using TNT-T7 Quick for PCR DNA system (Promega) using the primers in Supplementary Table 5. Differential methionine content was compensated for by the incorporation of additional methionines into the antisense primer. 25 μl reactions were assembled using 400 ng of PCR product as template DNA with 20 μl of TNT Quick Master Mix and 20 μCi of 35S-Met (Perkin-Elmer) and incubated for 90 mins at 30 °C with mixing.

### MCM2-7 DH-Sld3/7 (MS) complex assembly

MCM2-7 DH was prepared as described for the interaction assay. Following high-salt washes (preRC buffer + 100 mM KGlu + 300 mM NaCl), the beads were rinsed with preRC buffer + 100 mM KGlu before the addition of 400 nM DDK, and the mixture was then incubated for 30 min at 24 °C. DDK was removed using stripping buffer (preRC buffer + 100 mM KGlu + 500 mM NaCl) and the phosphorylated DH was eluted with AluI in Buffer C with 300 mM KOAc (25 mM HEPES-KOH pH 7.6, 300 mM KOAc, 5 mM MgAc, 3 mM ATP) plus 5% preRC buffer without KGlu. The eluate was incubated with Sld3/Sld7 at a threefold excess over the phosphorylated DH for 5 min at 24 °C. The mixture was then applied to a 15–45% (v/v) glycerol and 0–0.1% (v/v) glutaraldehyde (GraFix^39^) gradient in Buffer C with 300 mM KOAc. The sample was centrifuged at 48,477 × *g* for 16 h at 4 °C, and then 32 μl fractions were manually collected. The fractions containing the MS complex were pooled, and glycerol was removed from the sample using a Microcon DNA Fast Flow centrifugal filter unit (Merck) prior to grid preparation.

### MCM2-7 DH-Sld3/7-Cdc45 (MSC) complex assembly

The MSC sample was prepared in a similar manner to MS, as described above, with a few alterations and without the final gradient ultracentrifugation step that is typically used to remove residual intermediate DH to MSC species. After DDK was stripped using high salt, the phosphorylated DH was incubated with 400 nM Sld3/7 and 400 nM Cdc45 in preRC buffer + 100 mM KGlu + 0.1 mg/ml BSA for 5 min at 24 °C. The MSC complex was eluted with AluI in Buffer C with 100 mM KOAc (25 mM HEPES-KOH pH 7.6, 100 mM KOAc, 5 mM MgAc, 3 mM ATP) plus 5% preRC buffer without KGlu and crosslinked with 0.1% (v/v) glutaraldehyde for 15 min on ice. Crosslinking reactions were quenched by the addition of 1 M Tris-HCl, pH 8.0, to a final concentration of 100 mM, and the complex was then concentrated using a Microcon DNA Fast Flow centrifugal filter unit (Merck) prior to grid preparation.

### Sample preparation and data collection for NS-EM

Negative stain-EM sample preparation was carried out using 300-mesh copper grids with a thin carbon layer (Agar Scientific). Grids were glow-discharged for 30 s at 15 mA using a PELCO easiGlow device (Ted Pella, Inc.). Then, 3.5 µl of gradient-purified crosslinked Sld3/7 or Sld3/7-Cdc45 sample was applied to the grids and incubated for 1 min before blotting the excess sample. The sample was stained with one application of 4 µl 2% (w/v) uranyl acetate solution for 1 min before blotting. Integrated micrographs were collected with a Talos F200i TEM (Thermo Fisher Scientific) operated at 200 kV and equipped with a Falcon 3EC direct electron detector. EPU software was used for automated acquisition of micrographs at a nominal magnification of x92,000 and a physical pixel size of 1.6 Å and using a defocus range from -1.0 to −3.0 μm.

### Negative stain-EM data processing of Sld3/7 and Sld3/7-Cdc45 complexes

Negative-stain EM micrographs were processed using RELION 3.1^51,52^. Particles were picked using the Laplacian-of-Gaussian (LoG) filter to select an initial set of particles. Contrast transfer function (CTF) parameters were estimated using CTFFIND4 (ver4.1.14)^53^. Extracted particles were then subjected to multiple rounds of reference-free 2D classification and 3D classification. The final set of particles and 3D volumes were then extracted and imported into cryoSPARC (ver 4.6.2)^54^ for further processing. Homogeneous refinement was performed yielding the final 3D refined maps. For the crosslinked Sld3/7 sample, a theoretical dimer of Sld3/7 was created using the crystal structures: Sld3 STD (PDB: 3WI3), Sld3/7 ND (PDB: 3 × 37) and Sld7 DD (PDB: 3 × 38), which were manually fit into the derived EM map. For the crosslinked Sld3/7-Cdc45 sample, which featured a larger EM envelope, an atomic model for Sld3 STD-Cdc45 was generated using the Sld3 STD crystal structure and Cdc45 (PDB: 5U8S) aligned to an AF3 prediction of Sld3 STD-Cdc45, and this model was fitted into the EM map.

### Sample preparation and data collection for cryo-EM

MS and MSC complexes were prepared as described above. For grid preparation, 3.5 μl of sample was applied to glow-discharged (15 mA for 30 sec using a PELCO easiGlow device, Ted Pella, Inc.) 300 mesh Quantifoil R 2/2 grids coated with an additional homemade 2–3 nm thin continuous carbon film. The grids were incubated for 30 s at 4 °C and 100% humidity environment before being blotted for 1.5 s (blot force of +2) and quickly plunged-frozen into liquid ethane cooled at liquid nitrogen temperatures using an FEI Vitrobot Mark IV. Grids were stored in liquid nitrogen until imaging.

All cryo-EM data were automatically collected on a Titan Krios transmission electron microscope (Thermo Fisher Scientific) operated at 300 kV, with EPU software. For the MS sample, movies were recorded in super-resolution mode on a Gatan K3 direct electron detector at a nominal magnification of 81,000x, corresponding to a pixel size of 0.530 Å/pixel. A total of 29,592 multi-frame movies were acquired with a total electron dose of 50.4 e^-^/Å^2^ fractionated into 50 frames, and a defocus range of −1.0 to −3.2 μm.

For the crosslinked MSC complex, two datasets were collected on the same microscope using a Falcon 3EC direct electron detector operated in linear mode at a nominal magnification of 81,000x, corresponding to a pixel size of 1.085 Å. Dataset I (DH/MSC data I), which was later used both for MSC reconstructions and for the phosphorylated DH, comprised 10,192 multi-frame movies recorded with a total electron dose of 58.4 e^−^/Å^2^ fractionated into 27 frames, and a defocus range of −1.0 to −3.5 μm. Dataset II (MSC data II) consisted of 6046 multi-frame movies collected with a total electron dose of 62.1 e^−^/Å^2^ fractionated into 27 frames, and a defocus range of −1.5 to −3.0 μm. An overview of all data collection parameters is provided in Supplementary Table 1.

### Cryo-EM data processing of MCM2-7 DH-Sld3/7 (MS)

Movies were motion corrected and summed using MotionCor2 (ver1.2.3)^55^, and CTF estimation was performed using CTFFIND4 (ver4.1.14)^53^. Image processing was initially performed using RELION (ver 4.0)^56^, with further processing using cryoSPARC (ver 4.6.2)^54^. Micrographs displaying Thon rings to beyond 5 Å resolution were retained, and the remainder discarded. After initial manual picking and 2D/3D classification to assess data quality and complex component occupancy, particles were automatically picked using TOPAZ^57^. Particles were then extracted in a 416 × 416 box (pixel size of 1.06 Å) and then down-sampled to 3.445 Å/pixel. The particle stack was divided into 20 equal subsets to facilitate manual curation. Each subset underwent two rounds of reference-free 2D classification (*T* = 2, *K* = 50), and particles belonging to class averages exhibiting high-resolution features and the expected DH views were retained. This resulted in 1.79 million DH-like particles, which were re-extracted at their original pixel size (1.06 Å/pixel) and subjected to 3D refinement in RELION, yielding a 3.8 Å reconstruction that showed diffuse density at the center of the DH near the Mcm2/6 N-terminal domains and a prominent helix along the Mcm6 NTD-CTD interface. A further round of 2D classification (CTF, *T* = 2, *K* = 50) produced a cleaned set of 1,774,309 particles that was exported to cryoSPARC. These particles were subjected to an additional 2D classification, Fourier-cropped to a box size of 376 pixels (1.4434 Å/pixel), and then used for heterogeneous refinement into five classes. One class, containing 884,000 particles, displayed the best-resolved DH core and central density, and was taken forward as the consensus MS map and particle set for subsequent analyses. Because of flexibility and the limited particle number, subsequent processing focused on individual regions of interest to improve local density and assemble a more detailed picture of the interactions. The focused processing strategy was divided into four regions (I-IV): I) core DH; II) Sld3 MRD:Mcm6 interface; III) Sld3 MRD:Mcm4 interface; and IV) Sld7:Mcm6 interface.

For region I) core DH, the MS dataset exhibited increased heterogeneity within the DH core compared with phosphorylated DH and MSC datasets, most prominently an approximately 2° twist of the C-terminal lobe of the hexamers. To resolve discrete conformational states, the consensus MS particle set (882,092 particles) was subjected to homogeneous refinement with a tight mask around the DH core, followed by 3D classification in cryoSPARC into two classes (filter resolution 3.5 Å). The higher-resolution class (511,094 particles) was further homogeneously refined and re-classified into four classes (filter resolution 5 Å), yielding three predominant C-lobe conformations (states I–III) and one intermediate between states II and III. State I showed highly diffuse C-lobe density, whereas states II and III displayed progressively more ordered twisted C-lobe arrangements. Homogeneous refinement of the three main states produced maps at mean resolutions of 3.2 Å (states I and II) and 3.3 Å (state III). To obtain a better DH core, state II, which displayed the most rigid DH core (138,018 particles), was selected for further processing. These particles were subjected to two rounds of 3D classification and non-uniform refinement, followed by a final non-uniform refinement with 66,052 particles and C2 symmetry applied, yielding a map with a rigid DH core at a mean resolution of 3.3 Å.

For region II), Sld3 MRD:Mcm6, the DH core consensus subset (511,094 particles) was subjected to homogeneous refinement (C1, 3.0 Å) and used as input for iterative cycles of masked 3D classification around the Sld3 MRD:Mcm6 interface (filter resolution 4 Å, no alignment) and homogeneous refinement, yielding a final subset of 63,192 particles with ordered MRD density. Local refinement with a tight mask around Sld3 MRD and adjacent Mcm6 interfaces produced a map at 3.4 Å mean resolution.

For region III, Sld3 MRD:Mcm4, an analogous masked classification and refinement strategy, using a mask centered on the Sld3:Mcm4 interface, produced a subset of 166,980 particles and a locally refined map at 3.0 Å resolution mean resolution.

For region IV) Sld7:Mcm6, the consensus MS particle pool (882,092 particles) was subjected to multiple rounds of masked 3D classification around the Sld7:Mcm6 interface and non-uniform refinement to enrich for particles with ordered Sld7 density, resulting in a non-uniform refinement with a 3.2 Å mean resolution and 156,193 particles. Masked 3D classification of this subset (C1, 3 classes, filter resolution 6 Å, no alignment) produced a class of 48,138 particles that was further refined (C1, 3.5 Å). Signal subtraction retaining only the Sld7:Mcm6 region and neighboring density, followed by local refinement with centering (masked), generated a map at 3.7 Å mean resolution in which Sld7 is clearly resolved docked onto the Mcm6 N-terminal and C-terminal domains. Focused maps from regions I-IV were combined using phenix.combine_focused_maps to generate a composite MS map (comprising 292,336 particles, including symmetry-expanded particles, corresponding to 217,502 original particles, of which 149,668 were C2 symmetry-expanded particle images derived from 74,834 original particles in region II), in which the DH core and all Sld3/Sld7 interfaces are represented at their locally optimal resolutions, and this composite map was used for atomic model building.

### Modeling MS conformational heterogeneity

To further understand conformational variability in MS, the consensus particle set (884,000 particles) was subjected to 3D classification in RELION, and the class with the best-resolved central DH density (339,564 particles) was selected for 3D refinement, yielding a 6.6 Å map with weak, diffuse density at the center of the DH, proximal to the Mcm2/6 N-terminal domains. Conformational heterogeneity was then analyzed using multi-body refinement in RELION, with two soft masks (extend 6 pixels, soft edge 6 pixels) defining two rigid bodies: one comprising the DH core and peripheral Sld3/7 density, and a second encompassing only the central Sld3/7 density. The 3D auto-refined particles were subjected to multi-body refinement and subsequent principal component analysis of relative body motions; the first three eigenvectors were visualized as a 10-frame series. For interpretation, the resulting body maps were Gaussian-filtered (sDev 2.5) in UCSF ChimeraX, enhancing visualization of the central MS density and its continuous conformational variability.

### Cryo-EM data processing of MCM2-7 DH-Sld3/7-Cdc45 (MSC)

Movies were motion corrected and summed using MotionCor2 (ver1.2.3)^55^, and CTF estimation was performed using CTFFIND4 (ver4.1.14)^53^. Image processing was initially performed using RELION (ver 4.0)^56^, with further processing using cryoSPARC (ver 4.6.2)^54^. Micrographs that featured Thon rings beyond 5 Å resolution and defocus values above −3.5 μm were retained, and the rest discarded. Initially, particles were manually picked to generate reference 2D classes for assessment of data quality and particle component occupancy. The particles were then automatically picked using TOPAZ^57^, with the default resnet16_u64 general model, for reference-free auto-picking using a maximum particle diameter of 300 Å and then extracted with a box size of 376 pixels and a pixel size of 1.085 Å/pixel. In total, 492,031 particles were auto-picked and subjected to 2D classification without CTF correction (*T* = 4, *K* = 50). Particles belonging to classes with clear DH-like features (200,093 particles) were retained for downstream processing. Duplicate particles within an inter-particle distance of 100 Å were removed, yielding a cleaned dataset of 186,548 particles. The DH-like particle set after 2D classification was subjected to 3D classification (*T* = 4, *K* = 5). One of the five classes (115,857 particles) exhibited a C2 symmetric configuration in which each hexamer was bound to Sld3/7–Cdc45 and showed better-resolved features than the remaining classes. These particles were then subjected to two iterative rounds of masked 3D classification, with a mask encompassing the entire MSC complex featuring one Sld3/7-Cdc45 better resolved than the second, followed by 3D refinement. Due to the low starting particle number, processing focused on resolving and understanding the flexibility of only one Sld3/7-Cdc45 to high-resolution. Any remaining duplicate particles, within a 150 Å inter-particle distance limit, were removed, leaving 100,039 particles. 3D refinement yielded a 4.8 Å map with now stronger density for one Sld3/7-Cdc45. Cdc45 could clearly be observed bound at the Mcm2/5 interface and multiple binding sites for Sld3/7. Further processing of this dataset alone produced maps worse than 5 Å resolution in the Sld3/7-Cdc45 regions, so data from an independent but identical sample preparation collected under identical imaging conditions (MSC sample data I) were combined, resulting in a total of 209,272 MSC-like particles. Focused 3D refinement using an MSC mask on the combined dataset yielded a 4.2 Å map. At higher map thresholds, Cdc45 was rigidly attached at the Mcm2/5 interface, whereas lower thresholds revealed multiple Sld3/7 binding sites at the Mcm4/6 interface. To ensure that all particles contained at least one copy of Sld3/7-Cdc45, particles were subjected to 3D classification without alignment (*T* = 10, *K* = 2) using a loose mask around one Sld3/7–Cdc45 and the Mcm2/5 interface. This separated the data into two distinct classes: one with a well-resolved Cdc45:Mcm2/5 interface (106,950 particles, 78%) and one with poor density in the expected Cdc45 region (30,850 particles, 22%). The better-resolved class was 3D refined to obtain a 4.5 Å map and subsequently re-extracted using a box size of 376 with a pixel size of 1.085 Å/pixel and recentered based on the 3D refinement positions. The 106,950 particles and corresponding 3D map, low-pass filtered to 60 Å, were imported into cryoSPARC (ver 4.6.2) for further processing. The imported particles were first subjected to C1 non-uniform refinement, yielding a 3.3 Å consensus map that contained a clearly defined Sld3/7-Cdc45 on one side of the DH, with weaker density for the second copy visible only at lower thresholds. Because of flexibility and the limited particle number, subsequent processing focused on individual regions of interest to improve local density and assemble a more detailed picture of the interactions. The focused processing strategy was divided into four regions (I-IV): I) core DH and central channel DNA; II) Sld3 MRD:Mcm4/6 interface; III) Sld3 STD/Cdc45:Mcm2/5 interface; and IV) Sld7:Mcm6 interface.

For region I (core DH and central channel DNA), applying C2 symmetry during non-uniform refinement produced a 3.0 Å map that was indistinguishable from the C1 reconstruction, indicating no detectable asymmetry in this region. To improve the density for the DH core and the associated DNA, C2 symmetry heterogeneous refinement was then performed with 3 classes, resulting in one class that displayed better resolved features for the core DH and for the Sld3/7-Cdc45 regions (74,834 particles). Although subsequent C2 non-uniform refinement yielded a map at 3.1 Å with a well-resolved core DH, the map still featured weak density for the DNA. So, to resolve this, 3D classification (using 2 classes and a filter resolution of 5 Å) was performed without alignment and with a loose cylindrical mask encompassing the central DNA channel. The approach enriched for DNA-bound particles, resulting in a clear split between particles bound to and free of DNA. The class with clear DNA density (38,859 particles) was refined using C2 non-uniform refinement to yield a 3.2 Å map with high-resolution features for both the DH core and the central DNA and sharpened with a B-factor of −80.

For region II (Sld3 MRD:Mcm4/6 interface), we used the subset of 74,834 particles that showed well-resolved density for the DH core and Sld3/7-Cdc45 but still weak DNA density. Sld3 MRD density at the Mcm4/6 interface was observed on both sides of each hexamer, although with slightly different local resolution. To improve this region, these particles were subjected to C2 non-uniform refinement, followed by symmetry expansion, yielding 149,668 symmetry-expanded particle images, derived from the 74,834 original particles. These were then subjected to local refinement with a soft mask encompassing a single Sld3 MRD:Mcm4/6 interface. This procedure yielded a locally refined reconstruction at a mean resolution of 3.2 Å with well resolved features at the Sld3 MRD:Mcm4/6 contacts.

For region III (Sld3 STD/Cdc45:Mcm2/5 interface), particles from the 108,950 consensus dataset were used to focus on the Sld3/Cdc45:Mcm2/5 region. Notably, both the Cdc45:Mcm2/5 and Sld3:Cdc45 interfaces displayed high flexibility relative to the DH core, and so focused 3D classification, with a soft loose mask surrounding the Sld3 STD/Cdc45:Mcm2/5 interface and 2 classes, was performed to enrich for particles with ordered density in this region. The particles split with one class having a much more defined density. The particles belonging to this class (52,475 particles) were then subjected to local non-uniform refinement, using the same mask, to improve the Sld3 STD/Cdc45:Mcm2/5 interface. The resulting map, with a mean resolution of 4.7 Å, clearly displayed high-resolution density for Sld3 STD/Cdc45 and Mcm2/5. A manually generated loose mask around the Sld3 STD/Cdc45:Mcm2/5 segment was applied to generate a post-processed map with a resolution of 5.2 Å in RELION, sharpened with a B-factor of −210, which was subsequently combined into the composite MSC map.

For region IV (Sld7:Mcm6 interface), an analogous focused refinement strategy was employed using the same consensus set of particles; processing was continued in RELION. The particles were subjected to 3D classification (*T* = 10, *K* = 5), using a soft, loose mask encompassing the Sld7:Mcm6 subunits. Out of 5 classes, 1 class displayed a defined density for Sld7, the corresponding particles were then subject to 3D refinement followed by another round of focused 3D classification (*T* = 10, *K* = 3) and refinement. A set of 51,334 particles, which features clear density for Sld7, was then signal-subtracted to keep Sld7:Mcm6 and neighboring regions. The signal-subtracted particles were then imported into cryoSPARC for 3D classification (1 class, 5 Å filter) followed by a final local refinement with centering, yielding a map with a mean resolution of 4.0 Å and sharpened with a B-factor of −50. Focused maps from regions I-IV were combined using phenix.combine_focused_maps to generate a composite MSC map in which the DH core and all Sld3/Sld7 interfaces are represented at their locally optimal resolutions, and this composite map was used for atomic model building.

### Modeling MSC conformational heterogeneity

To model continuous conformational heterogeneity and visualize the underlying motions in the cryo-EM dataset, the focused subset of 52,475 particles (encompassing strong density for the Sld3 STD-Cdc45 region) was used as input for 3D Flexible Refinement (3DFlex)^41^ and, in parallel, rigid refinement for comparison using cryoSPARC (ver 4.6.2)^54^. 3DFlex uses a deep learning motion field, parameterized with a graph neural network, to describe non-rigid deformations. The resulting latent space was sampled to obtain three representative trajectories (latent 0–2) that captured DH core conformational changes and rearrangements within Sld3/7-Cdc45 regions. Frames were generated with 3DFlex Generate and assembled into a continuous movie (Supplementary Movie 4).

### Cryo-EM data processing of DDK-salt stripped phosphorylated DH

The phosphorylated DH was derived from a subset of particles without density for Sld3/7, Cdc45 or DDK within the MSC sample preparation. The data was processed as initially described for the MSC sample (MSC data I) above, with a total starting number of 535,412 DH-like particles post 2D classification (Supplementary Fig. 3). The particles were subjected to 3D classification (*T* = 4, *K* = 5), resulting in a clear separate class featuring 172,947 DH-DNA particles (32%) free of Sld3/7, Cdc45 or DDK expected densities. The particles were then re-extracted with a box size of 376 pixels and a pixel size of 1.085 Å/pixel and subjected to three rounds of iterative 3D classification without alignment (*T* = 10, *K* = 3), using a loose mask encompassing the entire DH, followed by 3D refinement yielding a map with a mean resolution of 4.2 Å and with 80,425 particles. For each classification, classes were selected based on the map that exhibited the best-resolved features. The resulting DH-DNA map still featured weak DNA density compared to the rest of the rigid core DH, and so to better resolve the DNA inside the MCM2-7 central channel, 3D classification without alignment (*T* = 4, *K* = 3) was performed with a mask encompassing only the DNA region. This resulted in a set of particles (64,506) that featured strong density for the DNA. 3D refinement with C1 symmetry was then performed, yielding a map at 4.2 Å resolution. The map featured C2 symmetry, as both hexamers were indistinguishable, and C2 symmetry refinement was therefore applied, yielding a post-processed reconstruction at a mean resolution of 3.3 Å (B-factor of −106 applied). For atomic model building, the map was further auto-sharpened in Phenix, applying an overall B-factor of −176 to a high-resolution cutoff of 3.3 Å.

All 3D refinements were performed using the gold-standard method, meaning all split data half-sets were completely independent, and resolutions reported are based on the Fourier shell correlation (FSC) cut-off criterion of 0.143.

### Predictive structural modeling of Sld3/7, Cdc45 and MS and MSC

For predicted structures of Sld3/7, Sld3/7-Cdc45, MCM2-7 DH-Sld3/7 (MS) and MCM2-7 DH-Sld3/7-Cdc45 (MSC) complexes (with some flexible regions omitted to allow for the computational prediction of the entire complex) LocalColabFold^58^ (https://github.com/YoshitakaMo/localcolabfold) was initially used, featuring AlphaFold2-multimer with versioned AF2-multimer weights. The AlphaFold3^59^ server was then used, which, having been trained on a more recent PDB release (cutoff September 30, 2021), surpassed ColabFold in model prediction accuracy by aligning more closely with deposited structural data and with our newly acquired EM data. For the MS and MSC complexes, two AlphaFold3 configurations were generated: model A included Mcm2 residues 1–160 (MS model A, ModelArchive: ma-x5w4s; MSC model A, ModelArchive: ma-1zsqn), whereas model B excluded Mcm2 residues 1–160 (MS model B, ModelArchive: ma-3pbi9; MSC model B, ModelArchive: ma-3dk7p) (Supplementary Table 4).

All predicted models shown were generated using the AlphaFold3 server, configured to run with 10 iterative recycles and 5 independent output models. The output models, ranked based on several metrics, were manually inspected, and the most reliable structure, based on predicted local distance difference test (pLDDT) scores, predicted aligned error (PAE), and similarity to existing structural data, was selected for subsequent analyses or as a template for atomic modeling.

### Atomic model building

Atomic model building was performed in Coot^60^ using existing structures as starting templates. For the DH core, MCM2-7 double hexamer models (PDB: 7PT6, 7P30, and 5BK4) were rigid-body fitted and adjusted to the local density. Sld3 and Sld7 were built using the structures of the Sld3 STD domain (PDB: 3WI3) and the Sld7 dimerization domain (PDB: 3 × 38) as initial templates, and Cdc45 was modeled starting from PDB: 6U0M. Missing loops, helices and interface regions were built de novo, guided by the local density, for Sld3 MRD2 and Sld7 regions. AlphaFold predictions were used to guide initial placement. The models were generated by iterative cycles of manual rebuilding and refinement. All models (phosphorylated-DH, MS, and MSC) were refined in Phenix real_space_refine^61^ with secondary structure, Ramachandran and rotamer restraints applied against their corresponding maps. Mg²⁺ ions were retained only in the phosphorylated-DH model, placed at canonical nucleotide-associated metal sites supported by local density and refined with bond-distance and angle restraints to the Walker A serine and nucleotide phosphate oxygens using phenix.ready_set. Mg²⁺ ions were omitted from the MS and MSC models, where weaker local density precluded unambiguous assignment at all nucleotide-bound sites (Supplementary Fig. 25d–f). Zn²⁺ ions were assigned at canonical coordination sites across all models, where supported by local density, and similarly refined with bond-distance and angle restraints using phenix.ready_set. Refinement and validation statistics for all three models are provided in Supplementary Table 3.

### Crosslinking mass spectrometry

To support our structural models and interrogate the 3D organization of flexible Sld3/7 regions, we crosslinked the MCM2-7 DH-Sld3/7 complex with bis(sulfosuccinimidyl) suberate (BS3) and identified residue pairs by crosslinking mass spectrometry (XL-MS). Phosphorylated DH and Sld3/7 were mixed at a 1:5 molar ratio in Buffer C with 300 mM KOAc + 0.1% (v/v) Triton X-100, incubated for 5 min at 24 °C, and then crosslinked with 5 mM BS3 on ice for 1.5 h and quenched with 100 mM Tris-HCl, pH 8.0. Crosslinked material was purified by 15–45% (v/v) glycerol gradient ultracentrifugation as above; fractions 9–11 were pooled and assessed by SDS-PAGE (Supplementary Fig. 13b). For mass spectrometry analysis, a single biological preparation (*n* = 1) of the crosslinked MCM2-7 DH-Sld3/7 sample (total of 42 µg protein) was precipitated with acetone. The protein pellet was resolubilized in digestion buffer (8 M urea in 100 mM ammonium bicarbonate) to an estimated protein concentration of 1 mg/ml. The disulfide bonds were reduced by the addition of 1 M DTT to a final concentration of 5 mM. The reaction was incubated at room temperature for 30 min. The free sulfhydryl groups in the sample were then alkylated by adding 500 mM iodoacetamide (final concentration of 15 mM) and incubation at room temperature for 20 min in the dark. After alkylation, an additional 1 M DTT was added (total concentration 10 mM) to quench the excess of iodoacetamide. Next, protein samples were digested with LysC (at a 50:1 (m/m) protein to protease ratio) at 25 °C for four hours. The sample was then diluted with 100 mM ammonium bicarbonate to reach a urea concentration of 1.5 M. Trypsin was added at a 50:1 (m/m) protein to protease ratio to further digest proteins overnight (~15 h) at 25 °C. Resulting peptides were desalted using C18 StageTips^62^. The resulting peptides were fractionated using size exclusion chromatography in order to enrich for crosslinked peptides^63^. Peptides were separated using a Superdex 30 Increase 3.2/300 column (GE Healthcare) at a flow rate of 10 μl/min. The mobile phase consisted of 30% (v/v) acetonitrile and 0.1% trifluoroacetic acid. The earliest six peptide-containing fractions (50 μl each) were collected. Solvent was removed using a vacuum concentrator. The fractions were then analyzed by LC-MS/MS. LC-MS/MS analysis was performed using an Orbitrap Fusion Lumos Tribrid mass spectrometer (Thermo Fisher Scientific), connected to an Ultimate 3000 RSLCnano system (Dionex, Thermo Fisher Scientific). Each size exclusion chromatography (SEC) fraction was resuspended in 1.6% v/v acetonitrile, 0.1% v/v formic acid and analyzed with LC-MS/MS acquisitions. For four of the total six SEC fractions that have adequate material, a duplicated LC-MS/MS acquisition was carried out. Peptides were injected onto a 50-centimeter EASY-Spray C18 LC column (Thermo Scientific) that is operated at 50 °C column temperature. Mobile phase A consists of water, 0.1% (v/v) formic acid, and mobile phase B consists of 80% (v/v) acetonitrile and 0.1% (v/v) formic acid. Peptides were loaded and separated at a flowrate of 0.3 μl/min. Peptides were separated by applying a gradient ranging from 2% to 55% B over 102.5 min. The slopes of the gradients were optimized for each SEC fraction. Following the separating gradient, the content of B was ramped to 55% and 95% within 2.5 min each. Eluted peptides were ionized by an EASY-Spray source (Thermo Scientific) and introduced directly into the mass spectrometer. The MS data was acquired in the data-dependent mode with the top-speed option. For each 2.5-s acquisition cycle, the full scan mass spectrum was recorded in the Orbitrap with a resolution of 120,000. The ions with a charge state from 3+ to 7+ were isolated and fragmented using higher-energy collisional dissociation (HCD). For each isolated precursor, stepped collision energy (26%, 28% and 30%) was applied. The fragmentation spectra were then recorded in the Orbitrap with a resolution of 60,000. Dynamic exclusion was enabled with single repeat count and 60 second exclusion duration. MS2 peak lists were generated from the raw mass spectrometric data files using the MSConvert module in ProteoWizard (version 3.0.11729). Precursor and fragment m/z values were recalibrated. Identification of crosslinked peptides was carried out using xiSEARCH software (https://www.rappsilberlab.org/software/xisearch) (version 1.7.6.4)^64^. The peak lists were searched against the sequences and the reversed sequences of subunits of the MCM2-7 DH-Sld3/7 complex. The following parameters were applied for the search: MS accuracy, 3 ppm; MS2 accuracy, 5 ppm; enzyme, trypsin (with full tryptic specificity); allowed number of missed cleavages, 3; missing monoisotopic peak, 2;cross-linker, BS3 (the reaction specificity was assumed to be for lysine, serine, threonine, tyrosine and protein N termini); fixed modifications, carbamidomethylation on cysteine; variable modifications, oxidation on methionine, hydrolyzed BS3, Tris reacted BS3, acetylation on lysine and deamidation on asparagine; maximum variable modification per peptide, 2. Identified crosslinked peptide candidates that have at least three matched fragment ions in each crosslinked peptide and at least two fragments containing residues of both peptides were filtered using xiFDR (version 2.1.5.6)^65^. The minimum peptide length was set to 5 amino acids. A false discovery rate of 1% on residue-pair level was applied with the boost between option selected. Results were inspected in xiVIEW^66^, and inter-residue connections were exported as pseudobonds (.pb) for structural mapping and visualized in UCSF ChimeraX using the XMAS plugin. To account for the two-fold symmetry of the MCM2-7 DH and Sld3 MRD regions, a simple python post-processing step mirrored equivalent links across corresponding subunits. For distance assessment we used a baseline BS3 cutoff of 24 Å (Cα-Cα distance) for allowed distances^67^ and distances in the 25–40 Å range were considered compatible with local flexibility and domain mobility, as evident in the MS cryo-EM data, whereas larger apparent violations were interpreted in light of conformational heterogeneity and potential alternative Sld3/7 stoichiometries, as suggested by in-solution mass photometry and EM analysis of Sld3/7 complexes alone.

### Mass photometry

Samples were prepared by incubating 400 nM Sld3/Sld7 or 400 nM Sld3/Sld7 + 400 nM Cdc45 in 25 mM HEPES-KOH pH 7.6, 5 mM MgAc, 300 mM KOAc, 1 mM DTT for 10 mins at 24 °C. Reactions were applied to 10–30% (v/v) glycerol gradients (same buffer without DTT) containing no crosslinker (native) or 0–0.2% (v/v) glutaraldehyde (GraFix). All gradients were centrifuged at 101924.6 x *g* for 16 h at 4 °C. 32 μl fractions were collected, and native fractions were analyzed by SDS-PAGE and silver staining to determine the density gradient sedimentation profiles of Sld3/7 and Cdc45. Fractions identified by SDS-PAGE were then collected from the crosslinked sample. 1 μL of each selected fraction was diluted into 19 μl of measurement buffer (25 mM HEPES-KOH pH 7.6, 5 mM MgAc, and 300 mM KOAc) in a six-well silicone gasket (Grace Bio-Labs) on a pre-cleaned glass coverslip (Refeyn). Measurements were performed using a Refeyn TwoMP Mass Photometer at room temperature. Movies, capturing landing events, were recorded for 60 s using AcquireMP software (version 2023 R2, Refeyn Ltd) and analyzed with DiscoverMP software (version 2023 R2, Refeyn Ltd). Data was output in the form of graphs that were automatically generated, each signal peak fit by a Gaussian curve, using the Refeyn DiscoverMP software. Prior to measurements, the instrument was calibrated using bovine serum albumin (66 kDa monomer, 132 kDa homodimer) and thyroglobulin (330 kDa monomer, 670 kDa homodimer) standards to ensure mass accuracy falls within the 5% +/- expected instrument error limit.

### Visualization

Protein sequence alignments were generated with Clustal Omega^68^ and manually annotated and colored. Structural figures were prepared with UCSF Chimera^69^ and ChimeraX^70^. Supplementary movies were produced in ChimeraX. Where indicated, EM density maps were enhanced with CryoSAMU^71^ to improve interpretability and generate smoothed maps for figure preparation. Supplementary Movie 3 presents a morph between the MCM2-7 DH-Sld3/7 and MCM2-7 DH-Sld3/7-Cdc45 structures; flexible regions absent from the high-resolution composite EM maps were modeled with AF3 and rigid-body fitted to low-resolution EM densities of corresponding size and shape derived from the same consensus particle set. The morph is illustrative and should not be interpreted as evidence for discrete intermediates.

### Reporting summary

Further information on research design is available in the Nature Portfolio Reporting Summary linked to this article.

## Supplementary information

Supplementary Information

Description of Additional Supplementary Files

Supplementary Movie 1

Supplementary Movie 2

Supplementary Movie 3

Supplementary Movie 4

Reporting Summary

Transparent Peer Review file

## Source data

Source Data

## Data Availability

The cryo-EM data in this study have been deposited (summarized in Supplementary Table 6) in the Electron Microscopy Data Bank (EMDB) under accession code EMD-55897 (DDK-salt stripped phosphorylated DH), EMD-55904 MCM2-7 DH-Sld3/7 (MS), EMD-55918 MCM2-7 DH-Sld3/7-Cdc45 (MSC), EMD-56011 negative-stain EM envelope of crosslinked Sld3/7, EMD-56012 negative-stain EM envelope of crosslinked Sld3/7-Cdc45, EMD-55972 MS consensus map and associated low resolution MS map with associated maps: (EMD-55973 [https://www.ebi.ac.uk/emdb/EMD-55973] C2 DH core, EMD-55974 Sld3 MRD1:Mcm6, EMD-55975 Sld3 MRD2:Mcm4, EMD-55976 Sld7:Mcm6), EMD-55980 MS DH core C-lobe twist consensus map with associated maps: (EMD-55981 [https://www.ebi.ac.uk/emdb/EMD-55981] C-lobe twist State I, EMD-55982 C-lobe twist State II, EMD-55983 C-lobe twist State III), EMD-55985 MSC consensus map with associated focused maps: (EMD-55986 [https://www.ebi.ac.uk/emdb/EMD-55986] DH core, EMD-55987 Sld3 MRD1/2:Mcm4/6, EMD-55988 Sld7:Mcm6, EMD-55989 Sld3 STD/Cdc45:Mcm2/5), MD-55990 3DFlex MSC, EMD-56009 C1 2x Sld3/7-Cdc45 bound MSC, EMD-56010 C2 2x Sld3/7-Cdc45 bound MSC. The atomic coordinates have been deposited in the RCSB Protein Data Bank under accession codes 9TGD (DDK-salt stripped phosphorylated DH), 9TGM MCM2-7 DH-Sld3/7 (MS) and 9TH8 MCM2-7 DH-Sld3/7-Cdc45 (MSC). The crosslinking mass spectrometry data have been deposited in the ProteomeXchange Consortium via the PRIDE partner repository under the accession code PXD070193. Previously published structural data used for comparison, rigid-body fitting and model building are available from the EMDB and RCSB Protein Data Bank under accession codes EMD-31684 (unphosphorylated MCM2-7 DH), 3WI3 (Sld3 STD), 3 × 37 [https://www.rcsb.org/structure/3X37] (Sld3/7 ND homolog), PDB:3 × 38 [https://www.rcsb.org/structure/3X38] (Sld7 DD), 5U8S (Cdc45), 6U0M (budding yeast Cdc45), 5DGO (human Cdc45), 5BK4 (DNA-bound MCM2-7 DH), 7P5Z (phospho-DH-DDK), 7P30 (DDK-salt-stripped phospho-DH), 7PT6 (DH-DDK complex), 5U8T (CMG), 6SKL (CMG-Ctf4-fork protection complex), and PDB:8KG6 [https://www.rcsb.org/structure/8KG6] (replisome). The AlphaFold3 models generated in this study have been deposited (summarized in Supplementary Table 4) in ModelArchive under accession codes ma-3dk7p [https://modelarchive.org/doi/10.5452/ma-3dk7p] (yeast MCM2–7–Sld3–Sld7–Cdc45 complex, MSC model B, excluding Mcm2 residues 1–160), ma-1zsqn [https://modelarchive.org/doi/10.5452/ma-1zsqn] (yeast MCM2–7–Sld3–Sld7–Cdc45 complex, MSC model A, including Mcm2 residues 1–160), ma-3pbi9 [https://modelarchive.org/doi/10.5452/ma-3pbi9] (yeast MCM2–7–Sld3–Sld7 complex, MS model B, excluding Mcm2 residues 1–160), ma-x5w4s [https://modelarchive.org/doi/10.5452/ma-x5w4s] (yeast MCM2–7–Sld3–Sld7 complex, MS model A, including Mcm2 residues 1–160), ma-7fp39 [https://modelarchive.org/doi/10.5452/ma-7fp39] (yeast Sld3 STD–Cdc45 complex), ma-38qbs [https://modelarchive.org/doi/10.5452/ma-38qbs] (yeast Sld3–Sld7–Cdc45 dimer, or (Sld3–Sld7–Cdc45)₂ complex), ma-r8lak [https://modelarchive.org/doi/10.5452/ma-r8lak] (yeast Sld3–Sld7–Cdc45 complex), ma-ykqf6 [https://modelarchive.org/doi/10.5452/ma-ykqf6] (yeast Sld3–Sld7 heterotetramer, or (Sld3–Sld7)₂ complex), ma-t59e5 [https://modelarchive.org/doi/10.5452/ma-t59e5] (human TRESLIN STD–Cdc45–Mcm2/Mcm5 complex), ma-ymqbw [https://modelarchive.org/doi/10.5452/ma-ymqbw] (yeast Sld7), and ma-algfi [https://modelarchive.org/doi/10.5452/ma-algfi] (yeast Sld3). The models’ requests for resources and reagents should be directed to the corresponding authors and will be fulfilled by them. Source data are provided with this paper.

## References

[1] Remus, D. et al. Concerted loading of Mcm2-7 double hexamers around DNA during DNA replication origin licensing. *Cell* **139**, 719–30 (2009). 19896182 10.1016/j.cell.2009.10.015 PMC2804858

[2] Evrin, C. et al. A double-hexameric MCM2-7 complex is loaded onto origin DNA during licensing of eukaryotic DNA replication. *Proc. Natl. Acad. Sci. USA* **106**, 20240–5 (2009). 19910535 10.1073/pnas.0911500106 PMC2787165

[3] Noguchi, Y. et al. Cryo-EM structure of Mcm2-7 double hexamer on DNA suggests a lagging-strand DNA extrusion model. *Proc. Natl. Acad. Sci. USA* **114**, E9529–E9538 (2017). 29078375 10.1073/pnas.1712537114 PMC5692578

[4] Abid Ali, F. et al. Cryo-EM structure of a licensed DNA replication origin. *Nat. Commun.* **8**, 2241 (2017). 29269875 10.1038/s41467-017-02389-0 PMC5740162

[5] Li, N. et al. Structure of the eukaryotic MCM complex at 3.8 A. *Nature* **524**, 186–91 (2015). 26222030 10.1038/nature14685

[6] Douglas, M. E., Ali, F. A., Costa, A. & Diffley, J. F. X. The mechanism of eukaryotic CMG helicase activation. *Nature* **555**, 265–268 (2018). 29489749 10.1038/nature25787 PMC6847044

[7] Yeeles, J. T., Deegan, T. D., Janska, A., Early, A. & Diffley, J. F. Regulated eukaryotic DNA replication origin firing with purified proteins. *Nature* **519**, 431–5 (2015). 25739503 10.1038/nature14285 PMC4874468

[8] Bai, L. et al. Architecture of the saccharomyces cerevisiae replisome. *Adv. Exp. Med Biol.* **1042**, 207–228 (2017). 29357060 10.1007/978-981-10-6955-0_10 PMC5800748

[9] Yardimci, H. & Walter, J. C. Prereplication-complex formation: a molecular double take? *Nat. Struct. Mol. Biol.* **21**, 20–5 (2014). 24389553 10.1038/nsmb.2738

[10] Bell, S. P. & Dutta, A. DNA replication in eukaryotic cells. *Annu Rev. Biochem* **71**, 333–74 (2002). 12045100 10.1146/annurev.biochem.71.110601.135425

[11] Deegan, T. D., Yeeles, J. T. & Diffley, J. F. Phosphopeptide binding by Sld3 links Dbf4-dependent kinase to MCM replicative helicase activation. *EMBO J.* **35**, 961–73 (2016). 26912723 10.15252/embj.201593552 PMC4864760

[12] Tanaka, S., Nakato, R., Katou, Y., Shirahige, K. & Araki, H. Origin association of Sld3, Sld7, and Cdc45 proteins is a key step for determination of origin-firing timing. *Curr. Biol.* **21**, 2055–63 (2011). 22169533 10.1016/j.cub.2011.11.038

[13] Costa, A. et al. The structural basis for MCM2-7 helicase activation by GINS and Cdc45. *Nat. Struct. Mol. Biol.* **18**, 471–7 (2011). 21378962 10.1038/nsmb.2004 PMC4184033

[14] Ilves, I., Petojevic, T., Pesavento, J. J. & Botchan, M. R. Activation of the MCM2-7 helicase by association with Cdc45 and GINS proteins. *Mol. Cell* **37**, 247–58 (2010). 20122406 10.1016/j.molcel.2009.12.030 PMC6396293

[15] Moyer, S. E., Lewis, P. W. & Botchan, M. R. Isolation of the Cdc45/Mcm2-7/GINS (CMG) complex, a candidate for the eukaryotic DNA replication fork helicase. *Proc. Natl. Acad. Sci. USA* **103**, 10236–10241 (2006). 16798881 10.1073/pnas.0602400103 PMC1482467

[16] Tanaka, S. et al. CDK-dependent phosphorylation of Sld2 and Sld3 initiates DNA replication in budding yeast. *Nature* **445**, 328–32 (2007). 17167415 10.1038/nature05465

[17] Zegerman, P. & Diffley, J. F. Phosphorylation of Sld2 and Sld3 by cyclin-dependent kinases promotes DNA replication in budding yeast. *Nature* **445**, 281–5 (2007). 17167417 10.1038/nature05432

[18] Watase, G., Takisawa, H. & Kanemaki, M. T. Mcm10 plays a role in the functioning of the eukaryotic replicative DNA helicase, Cdc45-Mcm-GINS. *Curr. Biol.* **22**, 343–9 (2012). 22285032 10.1016/j.cub.2012.01.023

[19] van Deursen, F., Sengupta, S., De Piccoli, G., Sanchez-Diaz, A. & Labib, K. Mcm10 associates with the loaded DNA helicase at replication origins and defines a novel step in its activation. *EMBO J.* **31**, 2195–206 (2012). 22433841 10.1038/emboj.2012.69 PMC3343467

[20] Kanke, M., Kodama, Y., Takahashi, T. S., Nakagawa, T. & Masukata, H. McM10 plays an essential role in origin DNA unwinding after loading of the CMG components. *EMBO J.* **31**, 2182–94 (2012). 22433840 10.1038/emboj.2012.68 PMC3343466

[21] Donley, N. & Thayer, M. J. DNA replication timing, genome stability and cancer: late and/or delayed DNA replication timing is associated with increased genomic instability. *Semin Cancer Biol.* **23**, 80–9 (2013). 23327985 10.1016/j.semcancer.2013.01.001 PMC3615080

[22] Groth, A. et al. Human Asf1 regulates the flow of S-phase histones during replicational stress. *Mol. Cell* **17**, 301–11 (2005). 15664198 10.1016/j.molcel.2004.12.018

[23] Lin, Y. C. & Prasanth, S. G. Replication initiation: Implications in genome integrity. *DNA Repair* **103**, 103131 (2021). 33992866 10.1016/j.dnarep.2021.103131 PMC8296962

[24] Mantiero, D., Mackenzie, A., Donaldson, A. & Zegerman, P. Limiting replication initiation factors execute the temporal program of origin firing in budding yeast. *EMBO J.* **30**, 4805–14 (2011). 22081107 10.1038/emboj.2011.404 PMC3243606

[25] Boos, D. et al. Regulation of DNA replication through Sld3-Dpb11 interaction is conserved from yeast to humans. *Curr. Biol.* **21**, 1152–7 (2011). 21700459 10.1016/j.cub.2011.05.057

[26] Zegerman, P. & Diffley, J. F. Checkpoint-dependent inhibition of DNA replication initiation by Sld3 and Dbf4 phosphorylation. *Nature* **467**, 474–8 (2010). 20835227 10.1038/nature09373 PMC2948544

[27] Saleh, A. et al. The structural basis of Cdc7-Dbf4 kinase-dependent targeting and phosphorylation of the MCM2-7 double hexamer. *Nat. Commun.* **13**, 2915 (2022). 35614055 10.1038/s41467-022-30576-1 PMC9133112

[28] Greiwe, J. F. et al. Structural mechanism for the selective phosphorylation of DNA-loaded MCM double hexamers by the Dbf4-dependent kinase. *Nat. Struct. Mol. Biol.* **29**, 10–20 (2022). 34963704 10.1038/s41594-021-00698-z PMC8770131

[29] Cheng, J. et al. Structural Insight into the MCM double hexamer activation by Dbf4-Cdc7 kinase. *Nat. Commun.* **13**, 1396 (2022). 35296675 10.1038/s41467-022-29070-5 PMC8927117

[30] Tanaka, T. et al. Sld7, an Sld3-associated protein required for efficient chromosomal DNA replication in budding yeast. *EMBO J.* **30**, 2019–30 (2011). 21487389 10.1038/emboj.2011.115 PMC3098486

[31] Costa, A. & Diffley, J. F. X. The initiation of eukaryotic DNA replication. *Annu Rev. Biochem* **91**, 107–131 (2022). 35320688 10.1146/annurev-biochem-072321-110228

[32] Sheu, Y. J. & Stillman, B. The Dbf4-Cdc7 kinase promotes S phase by alleviating an inhibitory activity in Mcm4. *Nature* **463**, 113–7 (2010). 20054399 10.1038/nature08647 PMC2805463

[33] Li, H. et al. Sld3CBD-Cdc45 structural insights into Cdc45 recruitment for CMG complex formation. (eLife Sciences Publications, Ltd, 2025). 10.7554/eLife.101717 PMC12416888 40920075

[34] Itou, H., Muramatsu, S., Shirakihara, Y. & Araki, H. Crystal structure of the homology domain of the eukaryotic DNA replication proteins Sld3/Treslin. *Structure* **22**, 1341–1347 (2014). 25126958 10.1016/j.str.2014.07.001

[35] Itou, H., Shirakihara, Y. & Araki, H. The quaternary structure of the eukaryotic DNA replication proteins Sld7 and Sld3. *Acta Crystallogr. D. Biol. Crystallogr.* **71**, 1649–56 (2015). 26249346 10.1107/S1399004715010457

[36] Simon, A. C., Sannino, V., Costanzo, V. & Pellegrini, L. Structure of human Cdc45 and implications for CMG helicase function. *Nat. Commun.* **7**, 11638 (2016). 27189187 10.1038/ncomms11638 PMC4873980

[37] Yuan, Z. et al. Structure of the eukaryotic replicative CMG helicase suggests a pumpjack motion for translocation. *Nat. Struct. Mol. Biol.* **23**, 217–24 (2016). 26854665 10.1038/nsmb.3170 PMC4812828

[38] Abid Ali, F. et al. Cryo-EM structures of the eukaryotic replicative helicase bound to a translocation substrate. *Nat. Commun.* **7**, 10708 (2016). 26888060 10.1038/ncomms10708 PMC4759635

[39] Kastner, B. et al. GraFix: sample preparation for single-particle electron cryomicroscopy. *Nat. Methods* **5**, 53–5 (2008). 18157137 10.1038/nmeth1139

[40] Fang, D., Cao, Q. & Lou, H. Sld3-MCM interaction facilitated by Dbf4-dependent kinase defines an essential step in eukaryotic DNA replication initiation. *Front Microbiol* **7**, 885 (2016). 27375603 10.3389/fmicb.2016.00885 PMC4901202

[41] Punjani, A. & Fleet, D. J. 3DFlex: determining structure and motion of flexible proteins from cryo-EM. *Nat. Methods* **20**, 860–870 (2023). 37169929 10.1038/s41592-023-01853-8 PMC10250194

[42] Georgescu, R. et al. Structure of eukaryotic CMG helicase at a replication fork and implications to replisome architecture and origin initiation. *Proc. Natl. Acad. Sci. USA* **114**, E697–E706 (2017). 28096349 10.1073/pnas.1620500114 PMC5293012

[43] Baretic, D. et al. Cryo-EM structure of the fork protection complex bound to CMG at a replication fork. *Mol. Cell* **78**, 926–940 e13 (2020). 32369734 10.1016/j.molcel.2020.04.012 PMC7276988

[44] Xu, Z. et al. Synergism between CMG helicase and leading strand DNA polymerase at the replication fork. *Nat. Commun.* **14**, 5849 (2023). 37730685 10.1038/s41467-023-41506-0 PMC10511561

[45] Herrera, M. C. et al. A reconstituted system reveals how activating and inhibitory interactions control DDK-dependent assembly of the eukaryotic replicative helicase. *Nucleic Acids Res.* **43**, 10238–50 (2015). 26338774 10.1093/nar/gkv881 PMC4666391

[46] Boos, D., Yekezare, M. & Diffley, J. F. Identification of a heteromeric complex that promotes DNA replication origin firing in human cells. *Science* **340**, 981–4 (2013). 23704573 10.1126/science.1237448

[47] Kohler, K. et al. The Cdk8/19-cyclin C transcription regulator functions in genome replication through metazoan Sld7. *PLoS Biol.* **17**, e2006767 (2019). 30695077 10.1371/journal.pbio.2006767 PMC6377148

[48] Klemm, R. D., Austin, R. J. & Bell, S. P. Coordinate binding of ATP and origin DNA regulates the ATPase activity of the origin recognition complex. *Cell* **88**, 493–502 (1997). 9038340 10.1016/s0092-8674(00)81889-9

[49] Speck, C., Chen, Z., Li, H. & Stillman, B. ATPase-dependent cooperative binding of ORC and Cdc6 to origin DNA. *Nat. Struct. Mol. Biol.* **12**, 965–71 (2005). 16228006 10.1038/nsmb1002 PMC2952294

[50] Burgers, P. M. Overexpression of multisubunit replication factors in yeast. *Methods* **18**, 349–55 (1999). 10454996 10.1006/meth.1999.0796

[51] Zivanov, J., Nakane, T. & Scheres, S. H. W. Estimation of high-order aberrations and anisotropic magnification from cryo-EM data sets in RELION-3.1. *IUCrJ* **7**, 253–267 (2020). 32148853 10.1107/S2052252520000081 PMC7055373

[52] Zivanov, J. et al. New tools for automated high-resolution cryo-EM structure determination in RELION-3. *Elife* **7**, e42166 (2018). 30412051 10.7554/eLife.42166 PMC6250425

[53] Rohou, A. & Grigorieff, N. CTFFIND4: Fast and accurate defocus estimation from electron micrographs. *J. Struct. Biol.* **192**, 216–21 (2015). 26278980 10.1016/j.jsb.2015.08.008 PMC6760662

[54] Punjani, A., Rubinstein, J. L., Fleet, D. J. & Brubaker, M. A. cryoSPARC: algorithms for rapid unsupervised cryo-EM structure determination. *Nat. Methods* **14**, 290–296 (2017). 28165473 10.1038/nmeth.4169

[55] Zheng, S. Q. et al. MotionCor2: anisotropic correction of beam-induced motion for improved cryo-electron microscopy. *Nat. Methods* **14**, 331–332 (2017). 28250466 10.1038/nmeth.4193 PMC5494038

[56] Kimanius, D., Dong, L., Sharov, G., Nakane, T. & Scheres, S. H. W. New tools for automated cryo-EM single-particle analysis in RELION-4.0. *Biochem J.* **478**, 4169–4185 (2021). 34783343 10.1042/BCJ20210708 PMC8786306

[57] Bepler, T., Kelley, K., Noble, A. J. & Berger, B. Topaz-Denoise: general deep denoising models for cryoEM and cryoET. *Nat. Commun.* **11**, 5208 (2020). 33060581 10.1038/s41467-020-18952-1 PMC7567117

[58] Mirdita, M. et al. ColabFold: making protein folding accessible to all. *Nat. Methods* **19**, 679–682 (2022). 35637307 10.1038/s41592-022-01488-1 PMC9184281

[59] Abramson, J. et al. Addendum: Accurate structure prediction of biomolecular interactions with AlphaFold 3. *Nature* **636**, E4 (2024). 39604737 10.1038/s41586-024-08416-7 PMC11634763

[60] Emsley, P., Lohkamp, B., Scott, W. G. & Cowtan, K. Features and development of Coot. *Acta Crystallogr. D. Biol. Crystallogr.* **66**, 486–501 (2010). 20383002 10.1107/S0907444910007493 PMC2852313

[61] Afonine, P. V. et al. Real-space refinement in PHENIX for cryo-EM and crystallography. *Acta Crystallogr D. Struct. Biol.* **74**, 531–544 (2018). 29872004 10.1107/S2059798318006551 PMC6096492

[62] Rappsilber, J., Mann, M. & Ishihama, Y. Protocol for micro-purification, enrichment, pre-fractionation and storage of peptides for proteomics using StageTips. *Nat. Protoc.* **2**, 1896–1906 (2007). 17703201 10.1038/nprot.2007.261

[63] Leitner, A., Walzheoeni, T. & Aebersold, R. Lysine-specific chemical cross-linking of protein complexes and identification of cross-linking sites using LC-MS/MS and the xQuest/xProphet software pipeline. *Nat. Protoc.* **9**, 120–137 (2013). 24356771 10.1038/nprot.2013.168

[64] Mendes, M. L. et al. An integrated workflow for crosslinking mass spectrometry. *Mol. Syst. Biol.* **15**, e8994 (2019). 31556486 10.15252/msb.20198994 PMC6753376

[65] Muller, F., Fischer, L., Chen, Z. A., Auchynnikava, T. & Rappsilber, J. On the reproducibility of label-free quantitative cross-linking/mass spectrometry. *J. Am. Soc. Mass Spectrom.* **29**, 405–412 (2018). 29256016 10.1007/s13361-017-1837-2 PMC5814520

[66] Combe, C. W., Graham, M., Kolbowski, L., Fischer, L. & Rappsilber, J. xiVIEW: visualization of crosslinking mass spectrometry data. *J. Mol. Biol.* **436**, 168656 (2024). 39237202 10.1016/j.jmb.2024.168656

[67] Gong, Z., Ye, S. X., Nie, Z. F. & Tang, C. The conformational preference of chemical cross-linkers determines the cross-linking probability of reactive protein residues. *J. Phys. Chem. B* **124**, 4446–4453 (2020). 32369371 10.1021/acs.jpcb.0c02522

[68] Sievers, F. et al. Fast, scalable generation of high-quality protein multiple sequence alignments using Clustal Omega. *Mol. Syst. Biol.* **7**, 539 (2011). 21988835 10.1038/msb.2011.75 PMC3261699

[69] Pettersen, E. F. et al. UCSF Chimera-a visualization system for exploratory research and analysis. *J. Comput Chem.* **25**, 1605–12 (2004). 15264254 10.1002/jcc.20084

[70] Pettersen, E. F. et al. UCSF ChimeraX: structure visualization for researchers, educators, and developers. *Protein Sci.* **30**, 70–82 (2021). 32881101 10.1002/pro.3943 PMC7737788

[71] Zhang, C. & Dao Duc, K. CryoSAMU: Enhancing 3D cryo-EM density maps of protein structures at intermediate resolution with structure- aware multimodal U-nets. Preprint at *arXiv* 10.48550/arXiv.2503.20291 (2025).

