# Supplementary material for: Structural insights into Sld3-Sld7-dependent Cdc45 loading during replication initiation
Source: Nat Commun. 2026 Aug 14;17:9783. doi: 10.1038/s41467-026-76309-6 (PMC13572456; doi:10.1038/s41467-026-76309-6)
Supplement: Supplementary file 1 [file 41467_2026_76309_MOESM1_ESM.pdf]

Supplementary Information

Structural insights into Sld3/7-dependent Cdc45 loading during replication initiation

Yasunori Noguchi[1,*,&], Almutasem Saleh[1,*,#], Sarah Schneider[1], Marina E. Ivanova [1,], Zhuo Angel Chen[2], Lepakshi Ranjha[1,+], Ricardo Aramayo[3], Silvia Tognetti[1], Sarah V. Faull[1], Juri Rappsilber[2,4] and Christian Speck[1,3,#]

[1] DNA Replication Group, Institute of Clinical Sciences, Faculty of Medicine, Imperial College London, London W12 0HS, UK.

[2]Technische Universität Berlin, Chair of Bioanalytics, Berlin, Germany.

[3] MRC Laboratory of Medical Sciences (LMS), W12 0HS London, United Kingdom.

[4]Si-M/'Der Simulierte Mensch', Technische Universität Berlin and Charité, Universitätsmedizin Berlin, Berlin, Germany.

* These authors contributed equally

[#] Correspondence: chris.speck@imperial.ac.uk, a.saleh@lms.mrc.ac.uk

Current address:

[&]Department of Cellular Biochemistry, Graduate School of Pharmaceutical Sciences, Kyushu University, 3-1-1 Maidashi, Higashi-ku, Fukuoka 812-8582, Japan.

[+]Institute of Molecular Biology (IMB), Mainz, Germany

This file contains: 25 Supplementary Figures and 6 Supplementary Tables

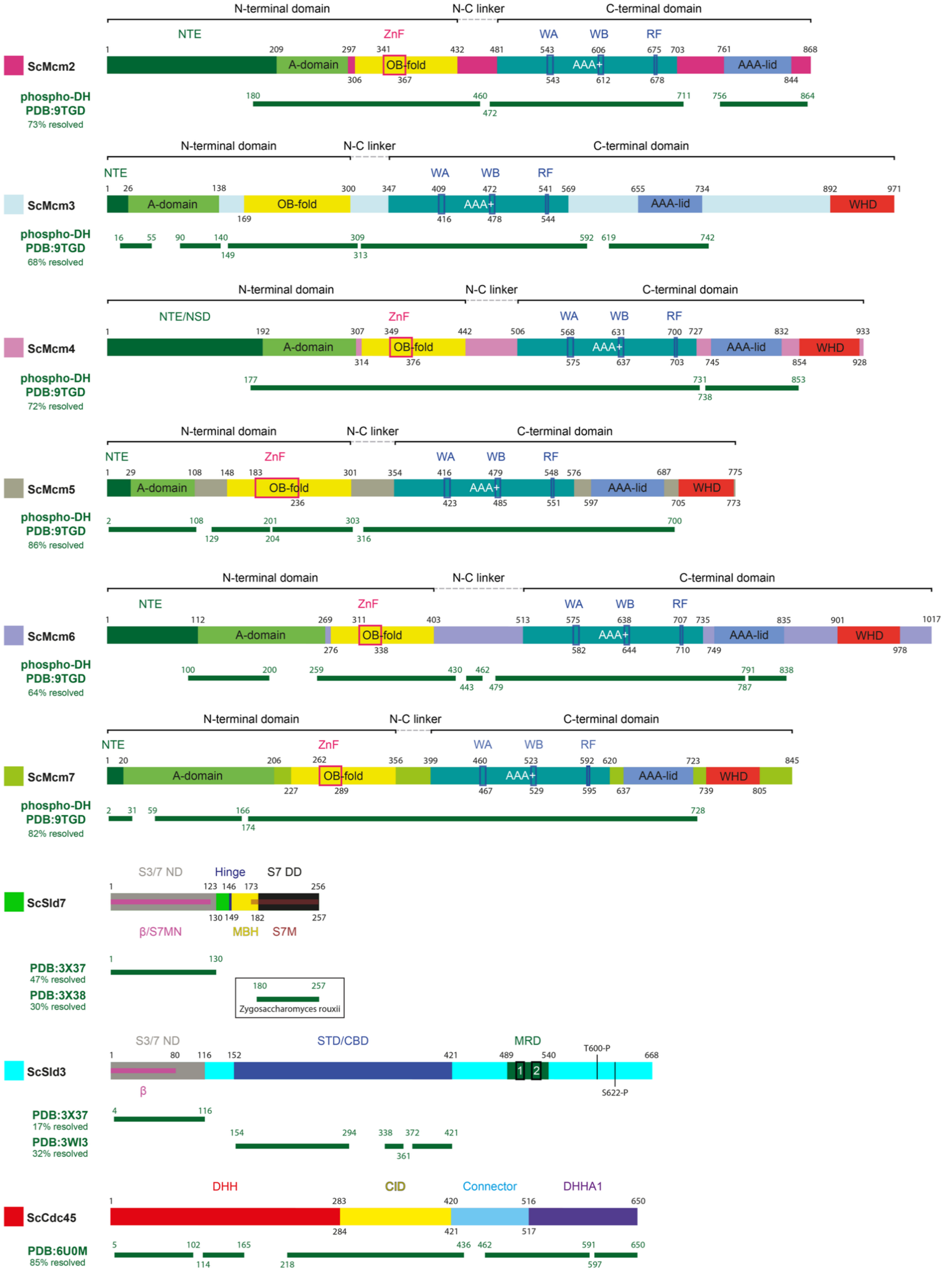

N-terminal domain
N-C linker
C-terminal domain
NTE
ZnF
WA
WB
RF
ScMcm2
1 209 297 341 432 481 543 606 675 703 761 868
A-domain
OB-fold
AAA+
AAA-lid
306 367 543 612 678 844
phospho-DH
PDB:9TGD
73% resolved
180 460 711 756 864
472
N-terminal domain
N-C linker
C-terminal domain
NTE
WA
WB
RF
ScMcm3
1 26 138 300 347 409 472 541 569 655 734 892 971
A-domain
OB-fold
AAA+
AAA-lid
WHD
169 416 478 544
phospho-DH
PDB:9TGD
68% resolved
16 55 90 140 309 592 619 742
149 313
N-terminal domain
N-C linker
C-terminal domain
NTE/NSD
ZnF
WA
WB
RF
ScMcm4
1 192 307 349 442 506 568 631 700 727 832 933
A-domain
OB-fold
AAA+
AAA-lid
WHD
314 376 575 637 703 745 854 928
phospho-DH
PDB:9TGD
72% resolved
177 731 853
738
N-terminal domain
N-C linker
C-terminal domain
NTE
ZnF
WA
WB
RF
ScMcm5
1 29 108 148 183 301 354 416 479 548 576 687 775
A-domain
OB-fold
AAA+
AAA-lid
WHD
236 423 485 551 597 705 773
phospho-DH
PDB:9TGD
86% resolved
2 108 201 303 700
129 204 316
N-terminal domain
N-C linker
C-terminal domain
NTE
ZnF
WA
WB
RF
ScMcm6
1 112 269 311 403 513 575 638 707 735 835 901 1017
A-domain
OB-fold
AAA+
AAA-lid
WHD
276 338 582 644 710 749 978
phospho-DH
PDB:9TGD
64% resolved
100 200 259 430 462 791 838
443 479 787
N-terminal domain
N-C linker
C-terminal domain
NTE
ZnF
WA
WB
RF
ScMcm7
1 20 206 262 356 399 460 523 592 620 723 845
A-domain
OB-fold
AAA+
AAA-lid
WHD
227 289 467 529 595 637 739 805
phospho-DH
PDB:9TGD
82% resolved
2 31 59 166 728
174
S3/7 ND
Hinge
S7 DD
ScSld7
1 123 146 173 256
130 149 182 257
β/S7MN
MBH
S7M
PDB:3X37
47% resolved
PDB:3X38
30% resolved
1 130
180 257
Zygosaccharomyces rouxii
S3/7 ND
STD/CBD
MRD
ScSld3
1 80 116 152 421 489 540 T600-P 668
1 2
β
S622-P
PDB:3X37
17% resolved
PDB:3WI3
32% resolved
4 116
154 294 338 372 421
361
DHH
CID
Connector
DHHA1
ScCdc45
1 283 420 516 650
284 421 517
PDB:6U0M
85% resolved
5 102 165 436 462 591 650
114 218 597

Supplementary Figure 1. 2D domain organization of MCM2-7, Sld3, Sld7 and Cdc45.

Domain organization and features of the MCM2-7-Sld3/7 (MS) and MCM2-7-Sld3/7-Cdc45 (MSC) complexes from budding yeast (Sc, Saccharomyces cerevisiae). The regions structurally resolved in this study are represented by a dark green bar below each domain schematic. MCM2-7 contain the following domains: N-terminal extension (NTE, also termed N-terminal serine/threonine-rich (NSD) domain), A-domain, oligonucleotide binding fold (OB-fold), AAA+ ATPase (AAA+) domain and winged helix domain (WHD). The MCM AAA+ domain has additional distinct motifs such as: Walker A (WA), Walker B (WB) and arginine finger (RF). The domain boundary of residues involved in the coordination of zinc ions is indicated by the zinc finger (ZF) domain. Cdc45 contains highly conserved domains among all eukaryotes including: N-terminal DHH (Aspartate-Histidine-Histidine motif) domain, CMG/C-terminal Interacting Domain (CID), connector/RecJ-like domain, C-terminal DHH Associated Domain from subfamily 1 (DHHA1) domain. Sld3 and Sld7 contain the following domains: Sld3/7 N-terminal dimerization (S3/7 ND), Cdc45-binding domain (CBD)/Sld3-Treslin domain (STD), MCM recognition domain (MRD) which includes the Mcm4 short interaction region (Sld3 Mcm4 SIR) and the MCM6 short interaction region (Sld3 Mcm6 SIR), Ku70-like β-barrel (β) domain or Sld7-MTBP N-terminal (S7MN) domain, Sld7 dimerization domain (S7 DD), Sld7 hinge domain, Sld7 Mcm6 short interaction region (Sld7 Mcm6 SIR), and the Sld7-MTBP C-terminal (S7M) domain. S-CDK phosphorylation sites on Sld3 (aa. 600 and 622) are shown.

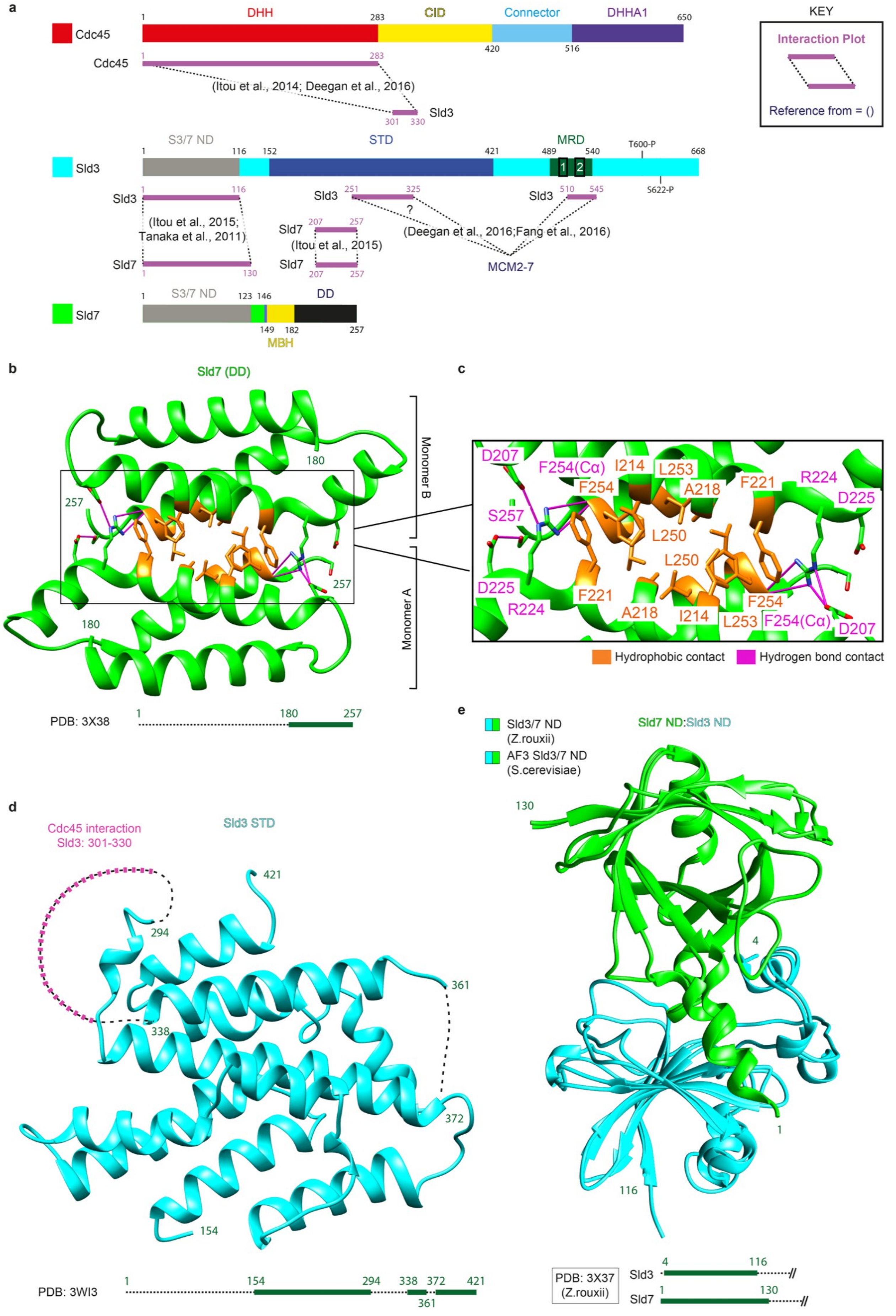
a
Cdc45
1
DHH
283
CID
Connector
DHHA1
650
420
516
Cdc45
1
283
(Itou et al., 2014; Deegan et al., 2016)
Sld3
301
330
KEY
Interaction Plot
Reference from = ()
S3/7 ND
STD
MRD
T600-P
Sld3
1
116
152
421
489
540
668
1
2
S622-P
Sld3
1
116
Sld3
251
325
Sld3
510
545
?
(Itou et al., 2015; Tanaka et al., 2011)
Sld7
207
257
(Itou et al., 2015)
(Deegan et al., 2016;Fang et al., 2016)
Sld7
1
130
Sld7
207
257
MCM2-7
S3/7 ND
DD
Sld7
1
123
146
149
182
257
MBH
b
Sld7 (DD)
Monomer B
Monomer A
180
257
257
180
PDB: 3X38
1
180
257
c
D207
F254(Cα)
I214
L253
F254
A218
F221
R224
D225
S257
L250
L250
D225
R224
F221
A218
I214
F254
L253
F254(Cα)
D207
Hydrophobic contact
Hydrogen bond contact
d
Cdc45 interaction
Sld3: 301-330
Sld3 STD
421
294
361
338
372
154
PDB: 3WI3
1
154
294
338
372
421
361
e
Sld3/7 ND (Z.rouxii)
AF3 Sld3/7 ND (S.cerevisiae)
Sld7 ND:Sld3 ND
130
4
1
116
PDB: 3X37 (Z.rouxii)
Sld3
4
116
Sld7
1
130

Supplementary Figure 2. Overview of previously reported Sld3, Sld7 and Cdc45 interactions and available structural models of Sld3/7.

a. Domain architecture of Sld3, Sld7 and Cdc45 and the interaction sites mapped in previous biochemical/structural studies (Itou et al., 2014; Itou et al., 2015; Deegan et al., 2016; Fang et al., 2016). Each protein is colored according to the keys shown. Unabbreviated domain names are detailed in Supplementary Figure 1. The DHH domain of Cdc45 has been implicated in binding the Sld3 Treslin domain (STD) of Sld3. In addition, two Sld3 segments, residues 251-325 and 510-545, have been reported to contact the MCM2-7 double hexamer, although the 251–325 region remains comparatively poorly defined. **b**. Crystal structure of a homodimer of the Sld7 dimerization domain (DD). The Sld7 dimerization interface is predominantly defined by hydrophobic contacts, residues key to these contacts are shown in orange. **c**. Zoomed-in view of the Sld7 dimer interface, residues forming hydrophobic contacts are colored in orange and hydrogen bond contacts represented as magenta sticks. **d**. Crystal structure of the STD of Sld3. The Sld3 region 301-330 encompassed within the STD, and reported to interact with Cdc45 (Itou et al., 2015), is intrinsically disordered within the crystal structure and is depicted by the dashed purple line. **e**. Crystal structure of a yeast homolog, Zygosaccharomyces rouxii, Sld3/Sld7 N-terminal DD superposed with an AlphaFold3 (AF3) model prediction of the corresponding domain from Saccharomyces cerevisiae. The two structures align closely, indicating a near-identical 3D fold. The individual structures in (**b**) and (**e**) together represent the key interaction domains that facilitate the formation of a Sld3/7 heterodimer.

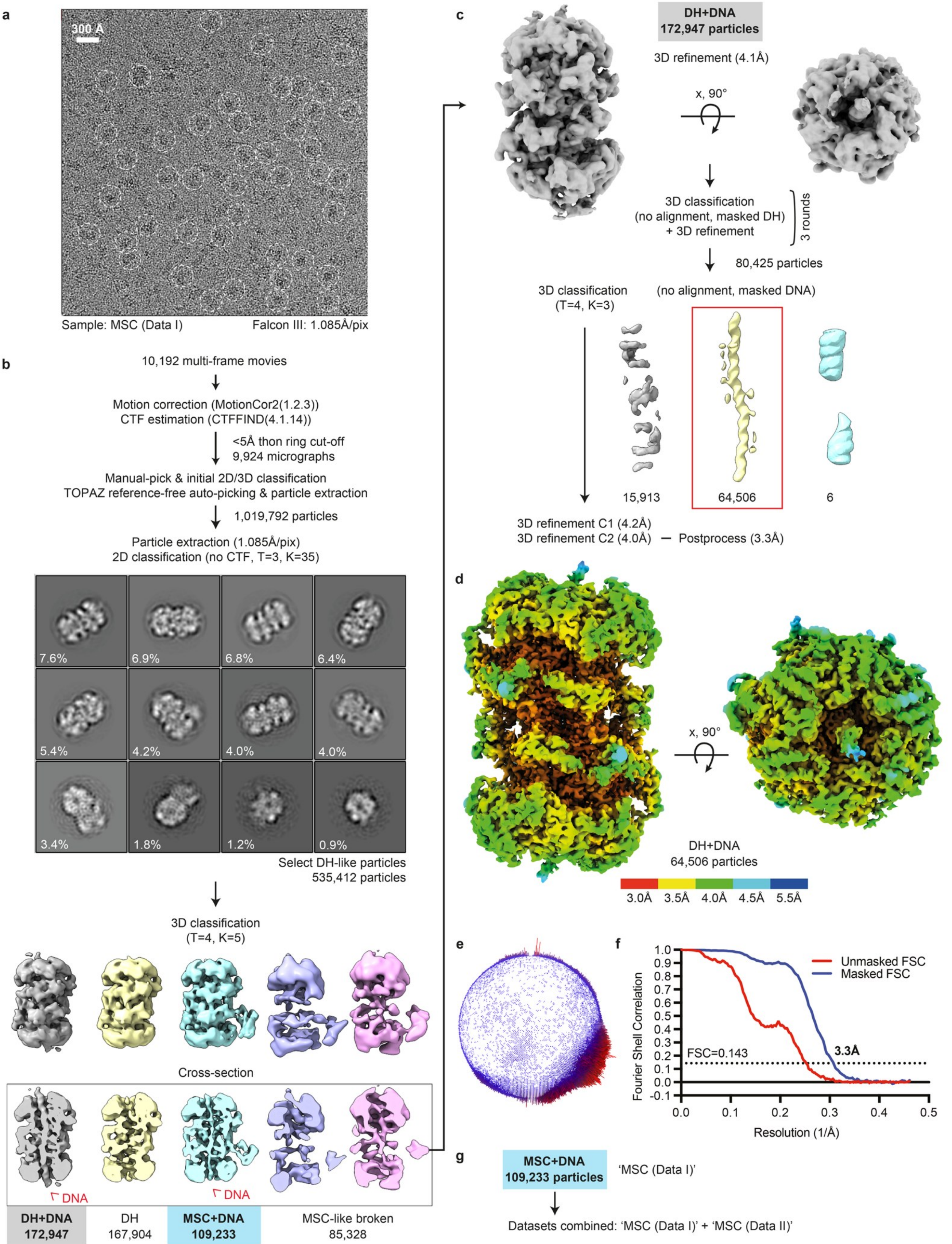
a
300 Å
Sample: MSC (Data I)
Falcon III: 1.085Å/pix
b
10,192 multi-frame movies
Motion correction (MotionCor2(1.2.3))
CTF estimation (CTFFIND(4.1.14))
<5Å thon ring cut-off
9,924 micrographs
Manual-pick & initial 2D/3D classification
TOPAZ reference-free auto-picking & particle extraction
1,019,792 particles
Particle extraction (1.085Å/pix)
2D classification (no CTF, T=3, K=35)
7.6%
6.9%
6.8%
6.4%
5.4%
4.2%
4.0%
4.0%
3.4%
1.8%
1.2%
0.9%
Select DH-like particles
535,412 particles
3D classification
(T=4, K=5)
Cross-section
DNA
DNA
DH+DNA
172,947
DH
167,904
MSC+DNA
109,233
MSC-like broken
85,328
c
DH+DNA
172,947 particles
3D refinement (4.1Å)
x, 90°
3D classification
(no alignment, masked DH)
+ 3D refinement
3 rounds
80,425 particles
3D classification
(T=4, K=3)
(no alignment, masked DNA)
15,913
64,506
6
3D refinement C1 (4.2Å)
3D refinement C2 (4.0Å)
Postprocess (3.3Å)
d
x, 90°
DH+DNA
64,506 particles
3.0Å
3.5Å
4.0Å
4.5Å
5.5Å
e
f
Unmasked FSC
Masked FSC
Fourier Shell Correlation
FSC=0.143
3.3Å
Resolution (1/Å)
g
MSC+DNA
109,233 particles
'MSC (Data I)'
Datasets combined: 'MSC (Data I)' + 'MSC (Data II)'

Supplementary Figure 3. Cryo-EM image processing workflow of DH/MSC (Data I) and 3D reconstruction of the DDK-salt stripped phosphorylated MCM2-7 DH complexed with DNA.

a. Representative cryo-EM micrograph, featuring monodispersed particles indicated by dotted white circles (n=1). **b**. Overview of image data processing workflow leading to a 3D classification, clearly separating MCM2-7 DH-like particles (not bound by Sld3/7-Cdc45 or DDK) and MSC-like particles. Movies were motion corrected and summed using MotionCor2 (ver1.2.3) and contrast transfer function (CTF) estimation was performed using CTFFIND4 (ver4.1.14). Micrographs that featured Thon rings 5 Å or better and defocus values above 3.5 μm were retained and the rest discarded. Initially, particles were manually picked to generate reference 2D classes to assess data quality and particle component occupancy, following this TOPAZ was used for reference-free auto-picking with a maximum diameter of 300 Å and then particles were extracted with a box size of 376 pixels and a pixel size of 1.085 Å/pixel. **c**. Downstream data processing workflow for the DDK-salt stripped phosphorylated MCM2-7 DH complexed with DNA map. **d**. 3D auto-refined and post-processed map of DDK-salt stripped phosphorylated MCM2-7 DH complexed with DNA at 3.3 Å mean resolution and with local resolution estimation between 3.0-4.0 Å. **e**. Euler angle particle distribution corresponding to the map shown in (**d**). The plots were generated using RELION's .bild file output (blue bars indicate the presence of particle views at specific orientations, while red bars highlight orientations with a higher relative abundance of particles). **f**. Gold-standard Fourier Shell Correlation (FSC) plot of the post-processed DDK-salt stripped phosphorylated MCM2-7 DH complexed with DNA map with and without a loose soft mask and a B-factor of -106 applied. **g**. Downstream continuation of the data processing workflow of MSC-like particles, which were combined with a second MSC data collection (MSC data II). See Supplementary Figure 15-16 for further processing. Source data are provided as a Source Data file.

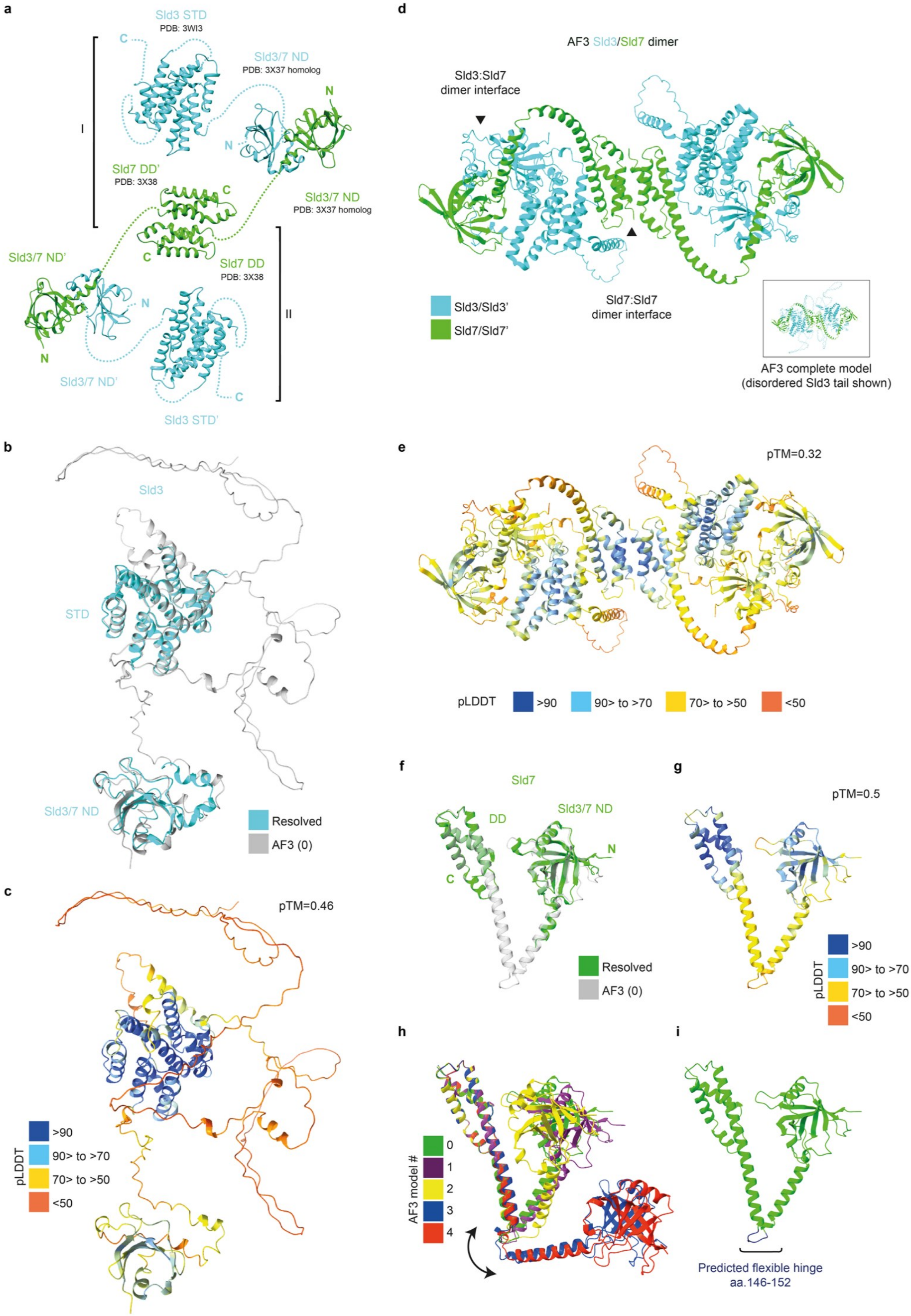
a
Sld3 STD
PDB: 3WI3
C
N
Sld3/7 ND
PDB: 3X37 homolog
N
I
Sld7 DD'
PDB: 3X38
C
C
Sld3/7 ND
PDB: 3X37 homolog
Sld3/7 ND'
Sld7 DD
PDB: 3X38
N
II
N
Sld3/7 ND'
C
Sld3 STD'
b
Sld3
STD
Sld3/7 ND
Resolved
AF3 (0)
c
pTM=0.46
pLDDT
>90
90> to >70
70> to >50
<50
d
AF3 Sld3/Sld7 dimer
Sld3:Sld7
dimer interface
Sld3/Sld3'
Sld7/Sld7'
Sld7:Sld7
dimer interface
AF3 complete model
(disordered Sld3 tail shown)
e
pTM=0.32
pLDDT
>90
90> to >70
70> to >50
<50
f
Sld7
DD
Sld3/7 ND
N
C
Resolved
AF3 (0)
g
pTM=0.5
pLDDT
>90
90> to >70
70> to >50
<50
h
AF3 model #
0
1
2
3
4
i
Predicted flexible hinge
aa.146-152

Supplementary Figure 4. AlphaFold3 (AF3) server predicted structural models of Sld7, Sld3 and Sld3/7 dimer.

a. Previously available structural data and current model of budding yeast Sld3/7 dimerization. Unabbreviated domain names are detailed in Supplementary Figure 1. **b.** AF3 prediction of full-length (FL) Sld3 (model 0, grey), superimposed on experimentally solved regions. **c**. AF3 prediction of FL Sld3 colored according to pLDDT scores. The selected model has the following metrics: 52% predicted disorder, 0.46 pTM score, 0.72 ranking score. **d**. AF3 prediction of FL Sld3/7 dimer with disordered Sld3 regions hidden from view. **e**. AF3 prediction of FL Sld3/7 dimer colored according to pLDDT scores. The selected model has the following metrics: 39% predicted disorder, 0.35 pTM score, 0.52 ranking score. **f.** AF3 prediction of FL Sld7 (model 0, grey), superimposed on experimentally solved regions. **g.** AF3 prediction of FL Sld7 colored according to pLDDT scores. The selected model has the following metrics: 10% predicted disorder, 0.50 pTM score, 0.55 ranking score. **h.** The Sld7 AF3 prediction indicated the presence of a flexible disordered hinge (aa. 146-152) that allows free movement of the Sld7 ND. Superposition of all 5 output models shows the extent of movement. **i.** AF3 prediction of FL Sld7 (model 0, green). All predicted models shown were generated with 10 recycles and total of 5 output models. The Sld3/7 dimerization mode established from experimental work was consistent with AF3 predictions and AF3 offered no reliable additional interaction to explore in this regard.

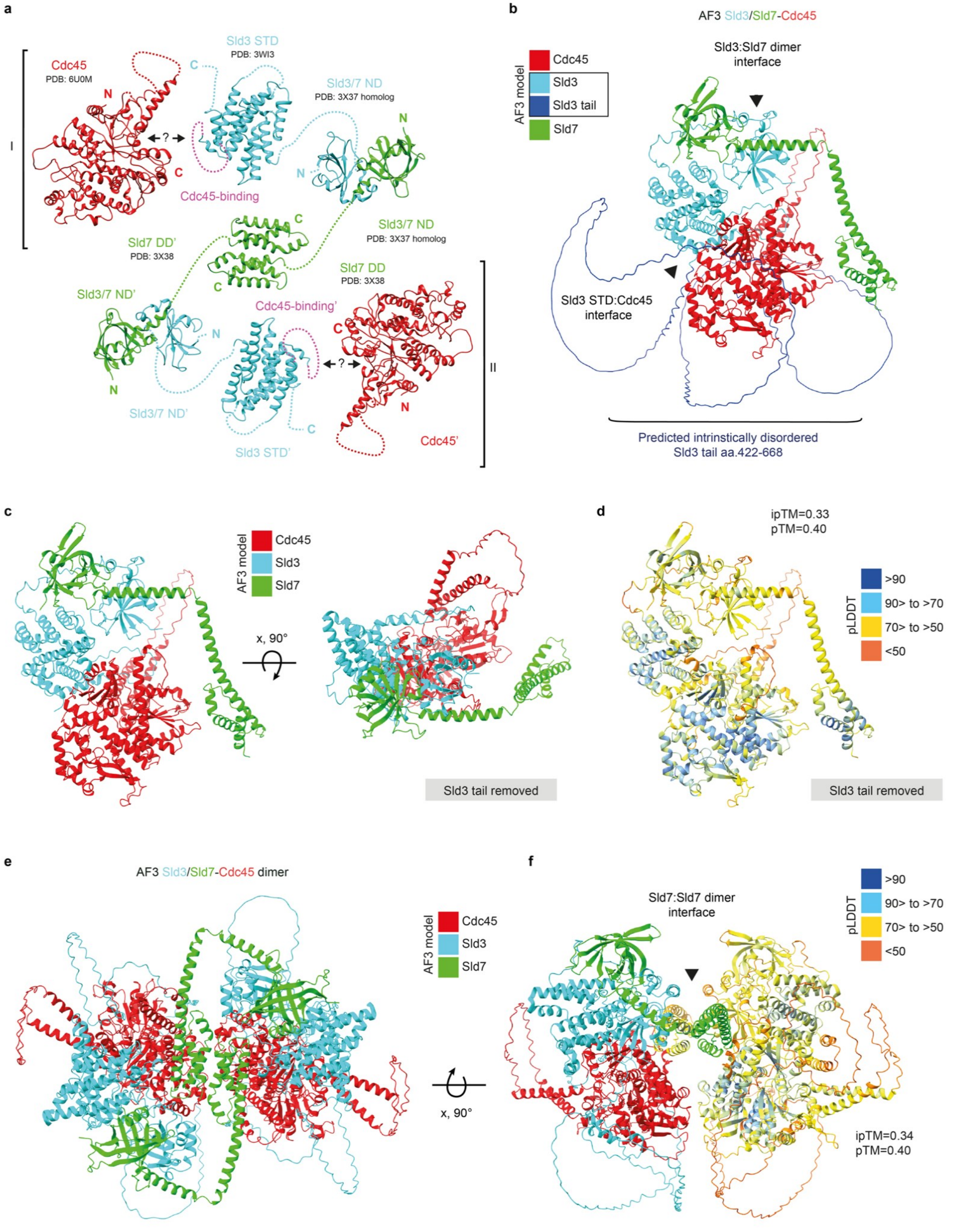

a
Cdc45
PDB: 6U0M
Sld3 STD
PDB: 3WI3
Sld3/7 ND
PDB: 3X37 homolog
Cdc45-binding
Sld7 DD'
PDB: 3X38
Sld3/7 ND
PDB: 3X37 homolog
Sld7 DD
PDB: 3X38
Sld3/7 ND'
Cdc45-binding'
Sld3/7 ND'
Sld3 STD'
Cdc45'
I
II
b
AF3 Sld3/Sld7-Cdc45
AF3 model
Cdc45
Sld3
Sld3 tail
Sld7
Sld3:Sld7 dimer interface
Sld3 STD:Cdc45 interface
Predicted intrinstically disordered
Sld3 tail aa.422-668
c
AF3 model
Cdc45
Sld3
Sld7
x, 90°
Sld3 tail removed
d
ipTM=0.33
pTM=0.40
pLDDT
>90
90> to >70
70> to >50
<50
Sld3 tail removed
e
AF3 Sld3/Sld7-Cdc45 dimer
AF3 model
Cdc45
Sld3
Sld7
x, 90°
f
Sld7:Sld7 dimer interface
pLDDT
>90
90> to >70
70> to >50
<50
ipTM=0.34
pTM=0.40

Supplementary Figure 5. AlphaFold3 (AF3) server predicted structural models of monomeric and dimeric Sld3/7-Cdc45.

a. Previously available biochemical and structural data and current model of budding yeast Sld3/7 and Cdc45. Unabbreviated domain names are detailed in Supplementary Figure 1. **b-c**. AF3 prediction of (**b**) full-length (FL) Sld3/7-Cdc45 (model 0) and (**c**) same model but with the Sld3 predicted intrinsically disordered tail hidden from view. **d**. AF3 prediction of FL Sld3/7-Cdc45 colored according to pLDDT scores. The selected model has the following metrics: 28% predicted disorder, 0.52 ipTM score, 0.56 pTM score, 0.67 ranking score. **e**. AF3 prediction of a dimer of FL Sld3/7-Cdc45 (model 0). **f**. AF3 prediction of FL Sld3 with one monomer colored according to pLDDT scores. Model metrics: 26% predicted disorder, 0.34 ipTM score, 0.40 pTM score, 0.48 ranking score. All predicted models shown were generated with 10 recycles and total of 5 output models. The Sld3/7 dimerization mode and Sld3:Cdc45 interaction region established from experimental work was consistent with AF3 predictions. The only region of Sld3/7 that is predicted to interact with, and couples, Cdc45 is the Sld3 STD. AF3 predictions shown featured a large disordered Sld3 tail (aa. 422-688), and so this region is hidden from view as indicated.

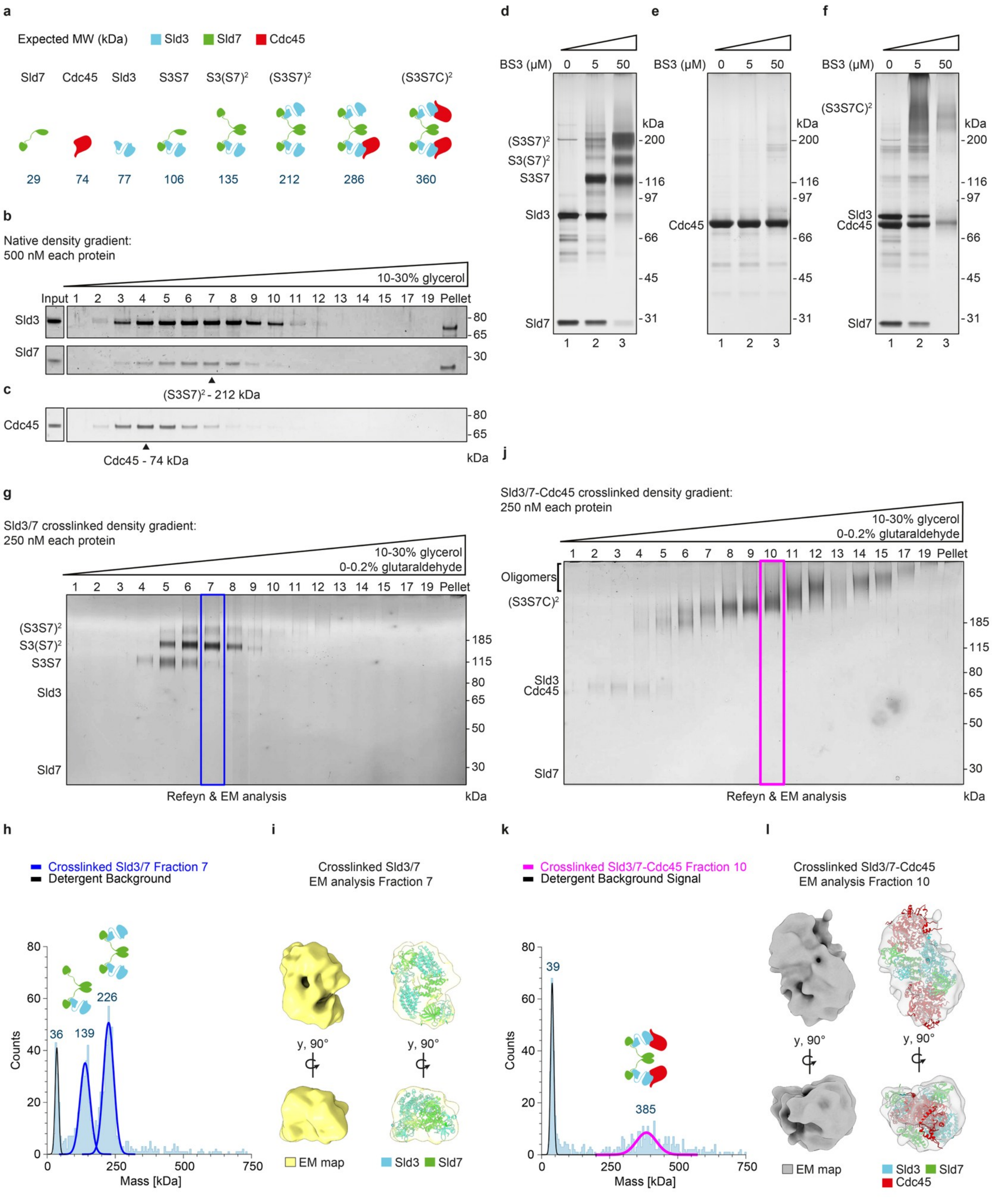

a
Expected MW (kDa)
Sld3
Sld7
Cdc45
Sld7
Cdc45
Sld3
S3S7
S3(S7)²
(S3S7)²
(S3S7C)²
29
74
77
106
135
212
286
360
b
Native density gradient:
500 nM each protein
10-30% glycerol
Input
Pellet
(S3S7)² - 212 kDa
c
Cdc45 - 74 kDa
d
e
f
BS3 (μM)
0
5
50
(S3S7)²
S3(S7)²
S3S7
Sld3
Sld7
Cdc45
(S3S7C)²
kDa
g
Sld3/7 crosslinked density gradient:
250 nM each protein
10-30% glycerol
0-0.2% glutaraldehyde
Refeyn & EM analysis
j
Sld3/7-Cdc45 crosslinked density gradient:
250 nM each protein
Oligomers
h
Crosslinked Sld3/7 Fraction 7
Detergent Background
Counts
Mass [kDa]
36
139
226
i
Crosslinked Sld3/7
EM analysis Fraction 7
y, 90°
EM map
k
Crosslinked Sld3/7-Cdc45 Fraction 10
Detergent Background Signal
39
385
l
Crosslinked Sld3/7-Cdc45
EM analysis Fraction 10

Supplementary Figure 6. Sld3/7 assembles as a heterodimer that further dimerizes into a tetramer and can bind Cdc45 independently of the MCM2-7 double hexamer.

a. Expected theoretical molecular weights of individual components and oligomeric assemblies, leading to a complex containing two copies of each subunit of Sld3/7 and Cdc45. **b-c.** Silver-stained SDS-PAGE of the sedimentation profile of density gradient-purified (**b**) Sld3/7 and (**c**) Cdc45 under native (no crosslinker) glycerol gradient conditions. The input lane represents 10 % of the sample loaded onto the gradient. Fractions with the highest protein abundance (peak fractions) are marked with black arrows. Data representative of 1 biological repeat. **d-f.** Silver-stained SDS-PAGE analysis of BS3 crosslinking of (**d**) Sld3/7, (**e**) Cdc45, and (**f**) Sld3/7-Cdc45. Sld3/7 is abbreviated as S3 or S7, and the Sld3/7-Cdc45 complex as S3S7C. Data representative of 3 biological repeats for 0 and 50 uM, 5 uM represents 1 illustrative biological repeat. **g.** Silver-stained SDS-PAGE of the sedimentation profile of Sld3/7 from a glycerol gradient containing a 0-0.2% glutaraldehyde crosslinker. Data represents one qualitative experiment. **h.** Mass photometry (data representative of 2 technical repeats) and **i.** negative-stain EM of the corresponding crosslinked Sld3/7 peak fraction (part (**g**) fraction 7). **j.** Silver-stained SDS-PAGE of the sedimentation profile of Sld3/7-Cdc45 from a glycerol gradient containing a 0-0.2% glutaraldehyde crosslinker. Data represents one qualitative experiment. **k.** Mass photometry (data representative of 1 biological repeat) and **l.** negative-stain EM of the corresponding crosslinked Sld3/7-Cdc45 peak fraction (part (**j**) fraction 10). The mass photometry background signal arises from detergent in the buffer and is shown as a black curve. The EM maps were rigid-body fitted with two copies of Sld3/7, guided using the crystal structures of Sld3 STD (PDB: 3WI3), Sld3/7 ND homolog (PDB: 3X37), and Sld7 DD (PDB: 3X38). For the larger Sld3/7-Cdc45 EM map, fitting was performed using the Sld3 STD crystal structure and Cdc45 (PDB: 5U8S) aligned to an AF3 prediction of Cdc45-Sld3 STD. Both EM maps could accommodate the expected densities and exhibited features consistent with approximate C2 symmetry, although the symmetry was not exact. Source data are provided as a Source Data file.

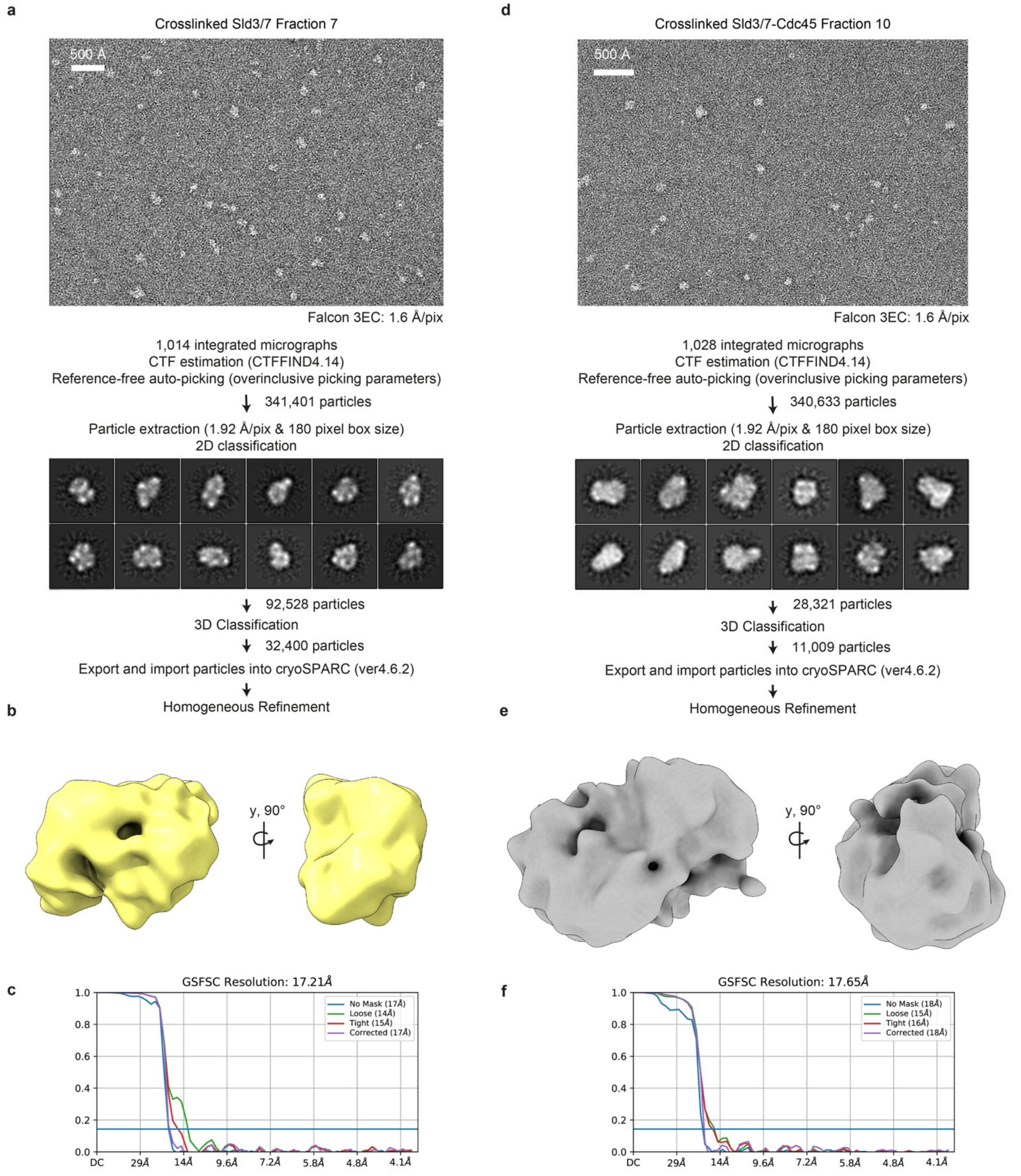

Supplementary Figure 7. Negative stain-EM analysis of Sld3/7 and Sld3/7-Cdc45 complex assembly.
a. Example micrograph of monodispersed particles of Sld3/7 complexes (Supplementary Figure 6g, fraction 7) and corresponding NS-EM data processing workflow (n=1) **b.** 3D homogeneous refined map of the crosslinked Sld3/7 sample. **c.** Gold-standard Fourier Shell Correlation (GSFSC) plot of the crosslinked Sld3/7 map at 17 Å resolution using the FSC cutoff at 0.143. **d.** Example micrograph of monodispersed particles of Sld3/7-Cdc45 complexes (Supplementary Figure 6j, fraction 10) and corresponding NS-EM data processing workflow (n=1). **e.** 3D homogeneous refined map of the crosslinked Sld3/7-Cdc45 sample. **f.** GSFSC plot of the crosslinked Sld3/7-Cdc45 map at 18 Å resolution using the FSC cutoff at 0.143. Source data are provided as a Source Data file.

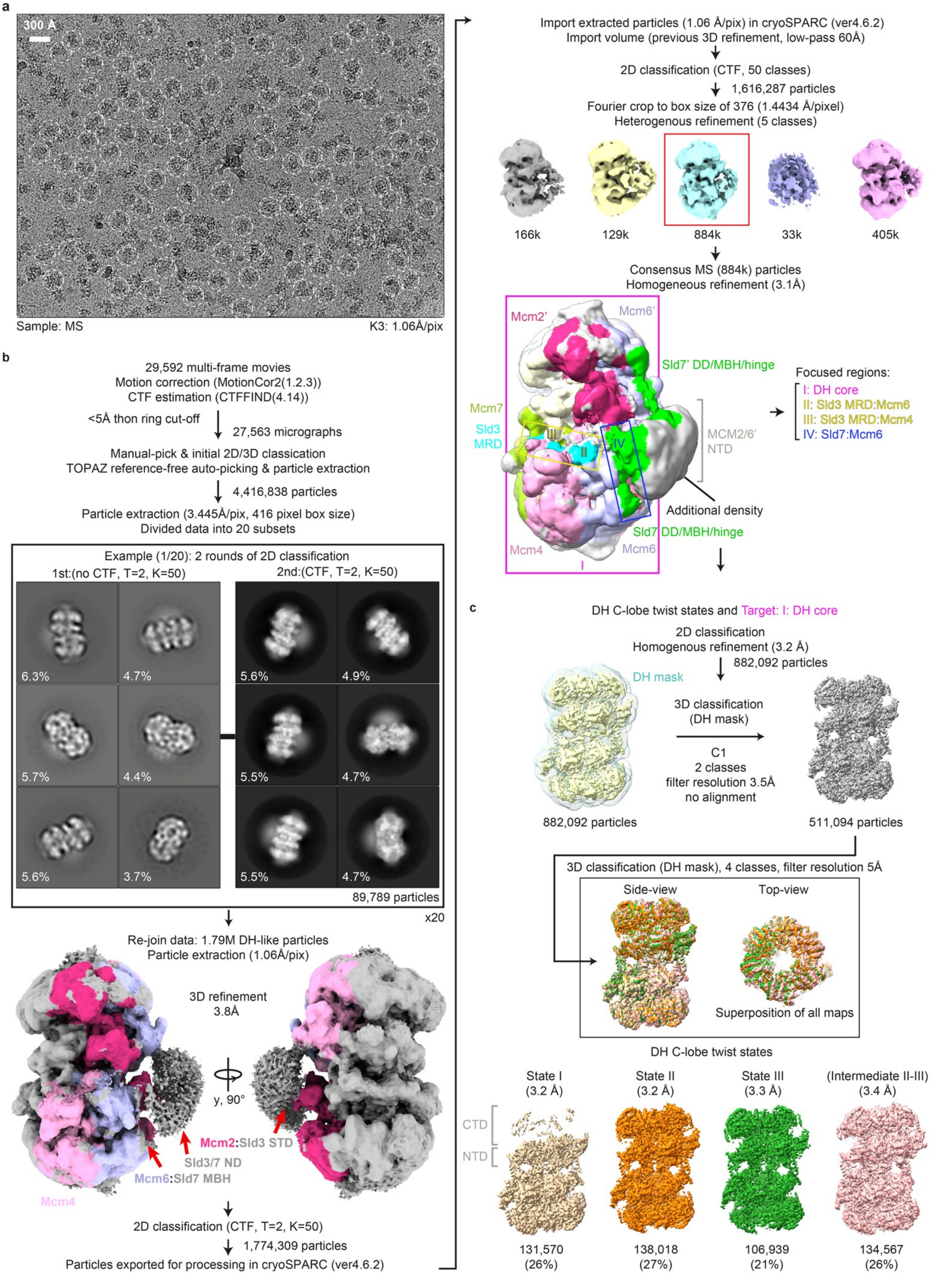
a
300 Å
Sample: MS
K3: 1.06Å/pix
b
29,592 multi-frame movies
Motion correction (MotionCor2(1.2.3))
CTF estimation (CTFFIND(4.14))
<5Å thon ring cut-off
27,563 micrographs
Manual-pick & initial 2D/3D classication
TOPAZ reference-free auto-picking & particle extraction
4,416,838 particles
Particle extraction (3.445Å/pix, 416 pixel box size)
Divided data into 20 subsets
Example (1/20): 2 rounds of 2D classification
1st:(no CTF, T=2, K=50)
2nd:(CTF, T=2, K=50)
6.3%
4.7%
5.7%
4.4%
5.6%
3.7%
5.6%
4.9%
5.5%
4.7%
5.5%
4.7%
89,789 particles
x20
Re-join data: 1.79M DH-like particles
Particle extraction (1.06Å/pix)
3D refinement
3.8Å
y, 90°
Mcm2:Sld3 STD
Sld3/7 ND
Mcm6:Sld7 MBH
Mcm4
2D classification (CTF, T=2, K=50)
1,774,309 particles
Particles exported for processing in cryoSPARC (ver4.6.2)
Import extracted particles (1.06 Å/pix) in cryoSPARC (ver4.6.2)
Import volume (previous 3D refinement, low-pass 60Å)
2D classification (CTF, 50 classes)
1,616,287 particles
Fourier crop to box size of 376 (1.4434 Å/pixel)
Heterogenous refinement (5 classes)
166k
129k
884k
33k
405k
Consensus MS (884k) particles
Homogeneous refinement (3.1Å)
Mcm2'
Mcm6'
Sld7' DD/MBH/hinge
Mcm7
Sld3
MRD
III
II
IV
MCM2/6'
NTD
Additional density
Sld7 DD/MBH/hinge
Mcm4
Mcm6
I
Focused regions:
I: DH core
II: Sld3 MRD:Mcm6
III: Sld3 MRD:Mcm4
IV: Sld7:Mcm6
c
DH C-lobe twist states and Target: I: DH core
2D classification
Homogenous refinement (3.2 Å)
882,092 particles
DH mask
3D classification
(DH mask)
C1
2 classes
filter resolution 3.5Å
no alignment
882,092 particles
511,094 particles
3D classification (DH mask), 4 classes, filter resolution 5Å
Side-view
Top-view
Superposition of all maps
DH C-lobe twist states
State I
(3.2 Å)
State II
(3.2 Å)
State III
(3.3 Å)
(Intermediate II-III)
(3.4 Å)
CTD
NTD
131,570
(26%)
138,018
(27%)
106,939
(21%)
134,567
(26%)

Supplementary Figure 8. Part 1 of 2: Cryo-EM image processing workflow for 3D reconstruction of the MCM2-7 DH-Sld3/7 (MS) complex.

a. Representative cryo-EM micrograph showing monodisperse particles, indicated by dotted white circles (n=1). **b.** Overview of the image processing workflow in RELION (ver4.0) leading to a DH-like particle stack for export to cryoSPARC (ver4.6.2). A total of 29,592 movies were motion corrected and summed in MotionCor2 (ver1.2.3) and CTF estimation was performed. Micrographs with visible Thon rings extending to at least 5 Å were retained. After initial manual picking and 2D/3D classification, particles were auto-picked in TOPAZ, yielding 4,416,838 particles. The dataset was divided into 20 equal subsets for manual curation by two rounds of reference-free 2D classification per subset. Classes displaying high-resolution features and the expected double hexamer views were retained, resulting in 1.79 million DH-like particles that were re-centered and re-extracted at 1.06 Å/pixel and subjected to 3D refinement (3.8 Å). A further round of 2D classification yielded 1,774,309 cleaned particles that were exported to cryoSPARC. The particles were subjected to 2D classification and Fourier-cropped, then used for heterogeneous refinement into five classes. One class, comprising 882,092 particles, displayed the strongest and best-resolved density at the DH center for Sld3/7 and was selected for homogeneous refinement (C1, 3.1 Å). This subset defines the consensus MS particle set from which all focused processing strategies were derived. Four regions were defined for focused analysis: I) DH core; II) Sld3 MRD:Mcm6 interface; III) Sld3 MRD:Mcm4 interface; and IV) Sld7:Mcm6 interface. **c.** The processing strategy used to resolve distinct DH C-lobe twist states and region I) DH core. The DH core particles (882,092 particles) were homogeneously refined (C1, 3.2 Å) and then classified into two classes with a DH mask, yielding a better-resolved class of 511,094 particles. To account for the residual unexpected heterogeneity within the C-terminal lobe, this subset was further classified with the same DH mask into four classes (filter resolution 5 Å), separating three predominant C-lobe conformations (states I-III) and one intermediate between states II and III, with map resolutions of 3.2-3.4 Å and particle numbers as indicated.

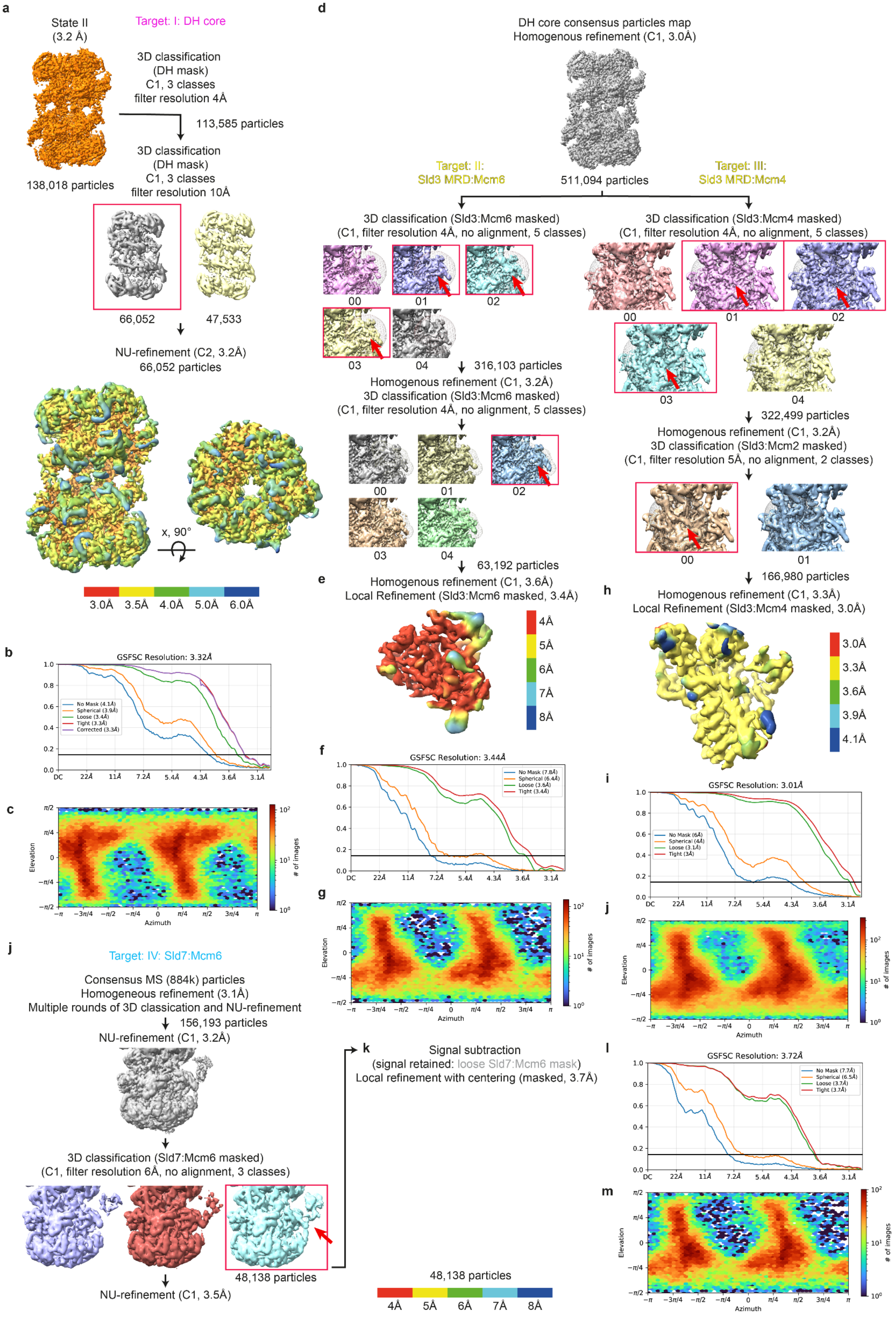
a
State II
(3.2 Å)
Target: I: DH core
3D classification
(DH mask)
C1, 3 classes
filter resolution 4Å
113,585 particles
3D classification
(DH mask)
C1, 3 classes
filter resolution 10Å
138,018 particles
66,052
47,533
NU-refinement (C2, 3.2Å)
66,052 particles
x, 90°
3.0Å
3.5Å
4.0Å
5.0Å
6.0Å
b
GSFSC Resolution: 3.32Å
No Mask (4.1Å)
Spherical (3.9Å)
Loose (3.4Å)
Tight (3.3Å)
Corrected (3.3Å)
c
Elevation
Azimuth
# of images
d
DH core consensus particles map
Homogenous refinement (C1, 3.0Å)
Target: II:
Sld3 MRD:Mcm6
511,094 particles
Target: III:
Sld3 MRD:Mcm4
3D classification (Sld3:Mcm6 masked)
(C1, filter resolution 4Å, no alignment, 5 classes)
00
01
02
03
04
316,103 particles
Homogenous refinement (C1, 3.2Å)
3D classification (Sld3:Mcm6 masked)
(C1, filter resolution 4Å, no alignment, 5 classes)
00
01
02
03
04
63,192 particles
3D classification (Sld3:Mcm4 masked)
(C1, filter resolution 4Å, no alignment, 5 classes)
00
01
02
03
04
322,499 particles
Homogenous refinement (C1, 3.2Å)
3D classification (Sld3:Mcm2 masked)
(C1, filter resolution 5Å, no alignment, 2 classes)
00
01
166,980 particles
e
Homogenous refinement (C1, 3.6Å)
Local Refinement (Sld3:Mcm6 masked, 3.4Å)
4Å
5Å
6Å
7Å
8Å
f
GSFSC Resolution: 3.44Å
No Mask (7.8Å)
Spherical (6.4Å)
Loose (3.6Å)
Tight (3.4Å)
g
Elevation
Azimuth
# of images
h
Homogenous refinement (C1, 3.3Å)
Local Refinement (Sld3:Mcm4 masked, 3.0Å)
3.0Å
3.3Å
3.6Å
3.9Å
4.1Å
i
GSFSC Resolution: 3.01Å
No Mask (6Å)
Spherical (4Å)
Loose (3.1Å)
Tight (3Å)
j
Elevation
Azimuth
# of images
j
Target: IV: Sld7:Mcm6
Consensus MS (884k) particles
Homogeneous refinement (3.1Å)
Multiple rounds of 3D classication and NU-refinement
156,193 particles
NU-refinement (C1, 3.2Å)
3D classification (Sld7:Mcm6 masked)
(C1, filter resolution 6Å, no alignment, 3 classes)
48,138 particles
NU-refinement (C1, 3.5Å)
k
Signal subtraction
(signal retained: loose Sld7:Mcm6 mask)
Local refinement with centering (masked, 3.7Å)
48,138 particles
4Å
5Å
6Å
7Å
8Å
l
GSFSC Resolution: 3.72Å
No Mask (7.7Å)
Spherical (6.5Å)
Loose (3.7Å)
Tight (3.7Å)
m
Elevation
Azimuth
# of images

Supplementary Figure 9. Part 2 of 2: Cryo-EM image processing workflow for 3D reconstruction of the MCM2-7 DH-Sld3/7 complex.

a-m. Data processing workflow for MS, outlining the focused processing of the DH core, Sld3 MRD:Mcm6, Sld3 MRD:Mcm4 and Sld7:Mcm6 interfaces, using cryoSPARC. **a.** Focused processing of the DH core was subjected to two rounds of 3D classification with a DH-mask. Non-uniform refinement with C2 symmetry applied produced a DH core map at 3.2 Å mean resolution. **b.** Gold-standard Fourier shell correlation (GSFSC) curves for the DH core non-uniform refinement shown in (**a**), with a mean resolution of 3.3 Å. **c.** Viewing direction distribution plot corresponding to the DH core reconstruction in (**a**). **d.** Focused processing of Sld3 MRD interfaces on Mcm6/Mcm4. The DH core consensus particle set was homogeneously refined and used for masked 3D classification around the Sld3 MRD:Mcm6 interface (left branch) or Sld3 MRD:Mcm4 interface (right branch). For **II**) Sld3 MRD:Mcm6: iterative cycles of homogeneous refinement and masked 3D classification yielded ordered MRD density. For **III**) Sld3 MRD:Mcm4: an analogous strategy enriched for ordered MRD density. **e.** Local refinement of the Sld3 MRD:Mcm6 interface, colored by local resolution. **f.** GSFSC curves for the locally refined Sld3 MRD:Mcm6 map in (**e**), reporting a mean resolution of 3.4 Å. **g.** Viewing direction distribution plot corresponding to the Sld3 MRD:Mcm6 local refinement in (**e**). **h.** GSFSC curves for the locally refined Sld3 MRD:Mcm4 map in (**d**). **i.** Viewing direction distribution plot corresponding to the Sld3 MRD:Mcm4 local refinement in (**h**). **j.** Focused processing of the **IV**) Sld7:Mcm6 interface. **k.** Signal subtraction retaining only the Sld7:Mcm6 region and neighboring density, followed by local refinement. **l.** GSFSC-curves for the Sld7:Mcm6 local refinement shown in (k), with a mean resolution of 3.7 Å. **m.** Viewing direction distribution plot corresponding to the Sld7:Mcm6 reconstruction in (**k**). The four focused maps (regions **I**-**IV**) were combined using phenix.combine_focused_maps to generate a composite MS map in which the DH core, Sld3 MRD:Mcm6, Sld3 MRD:Mcm4 and Sld7:Mcm6 interfaces are all represented at their locally optimal resolutions and supported atomic-model building. Source data are provided as a Source Data file.

a

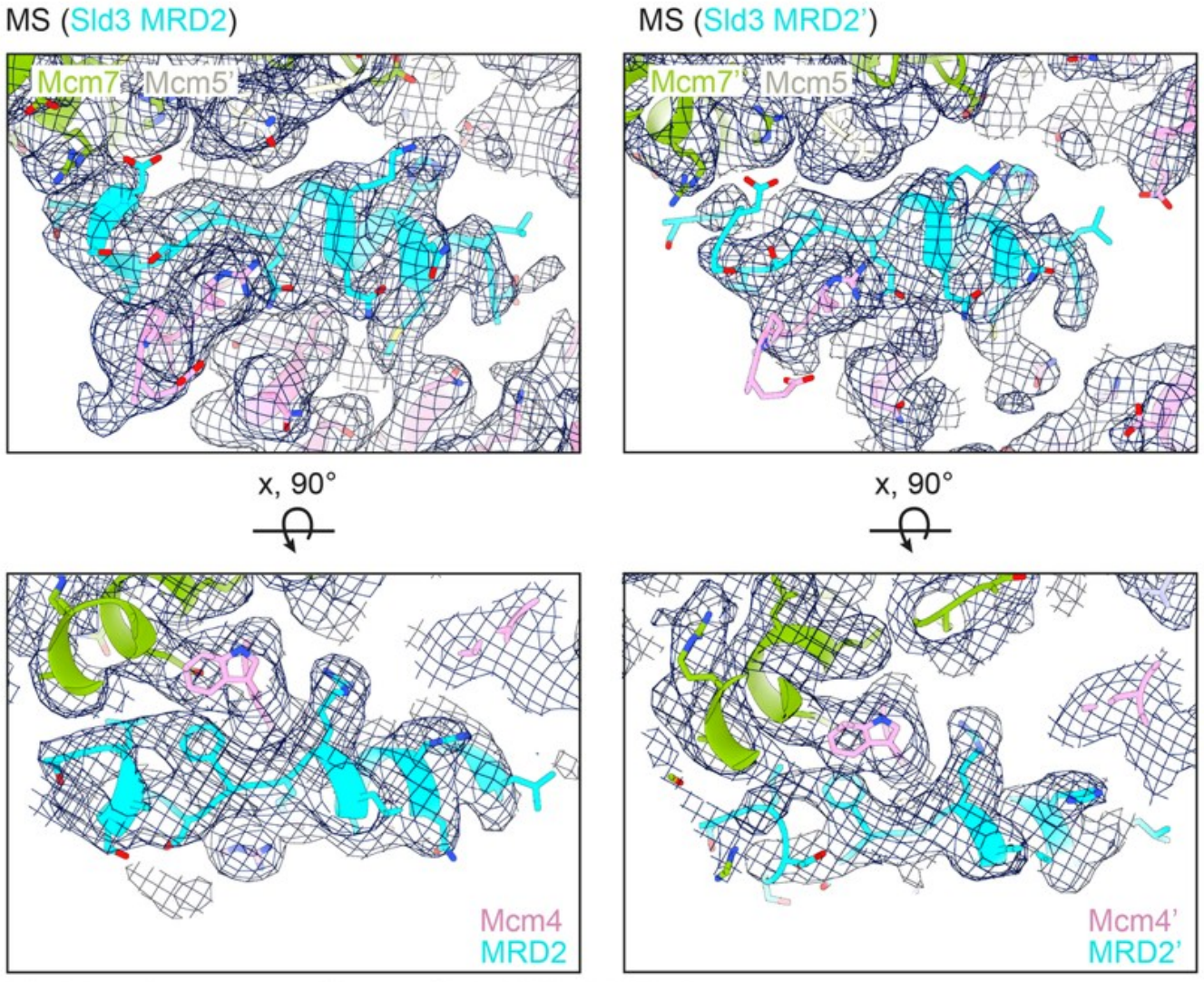

Model assignment confidence: Sld3 MRD2/MRD2'
De novo modelling + mutational analysis (Deegan et al., 2016)

b

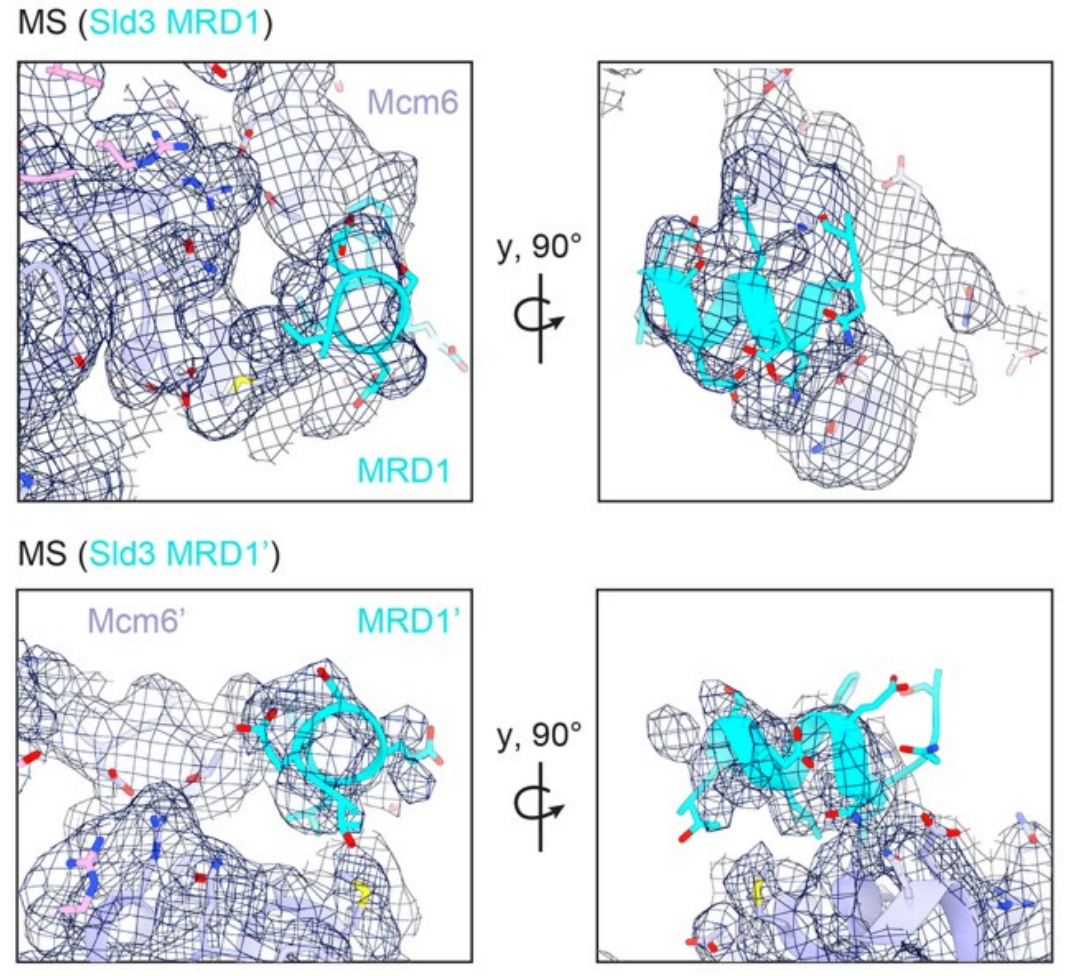

Model assignment confidence: Sld3 MRD1/MRD1'
De novo modelling + AF3 predicition

c

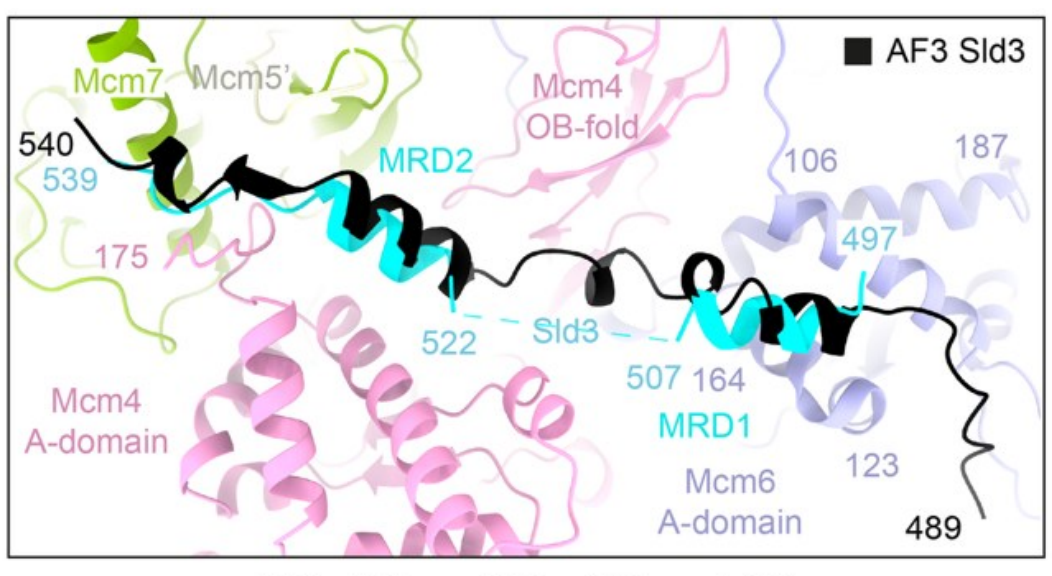

MS + AF3 predicition (MS model A)

d

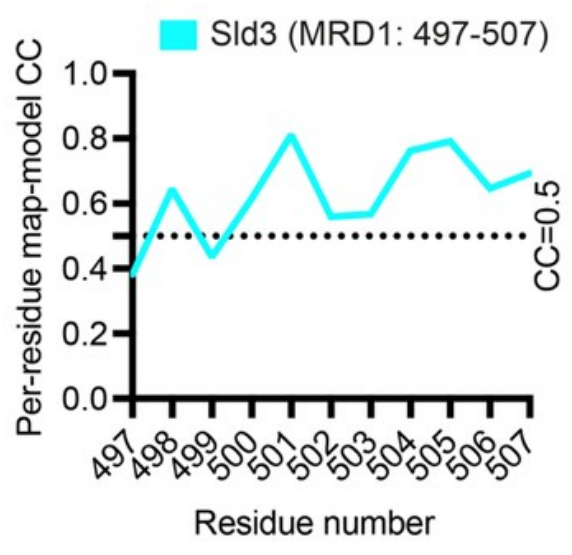

e

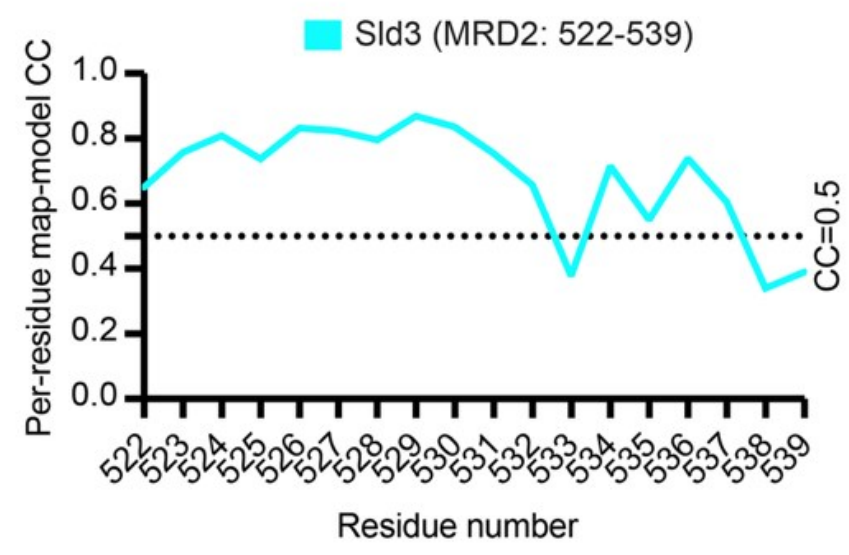

f

Sld3 Region:	MRD1	MRD2
CC_mask	0.544	0.669
Chain CC	0.513	0.501
Main-chain CC	0.658	0.568
Side-chain CC	0.475	0.531
Mean residue CC	0.628	0.682
Median residue CC	0.645	0.735

g

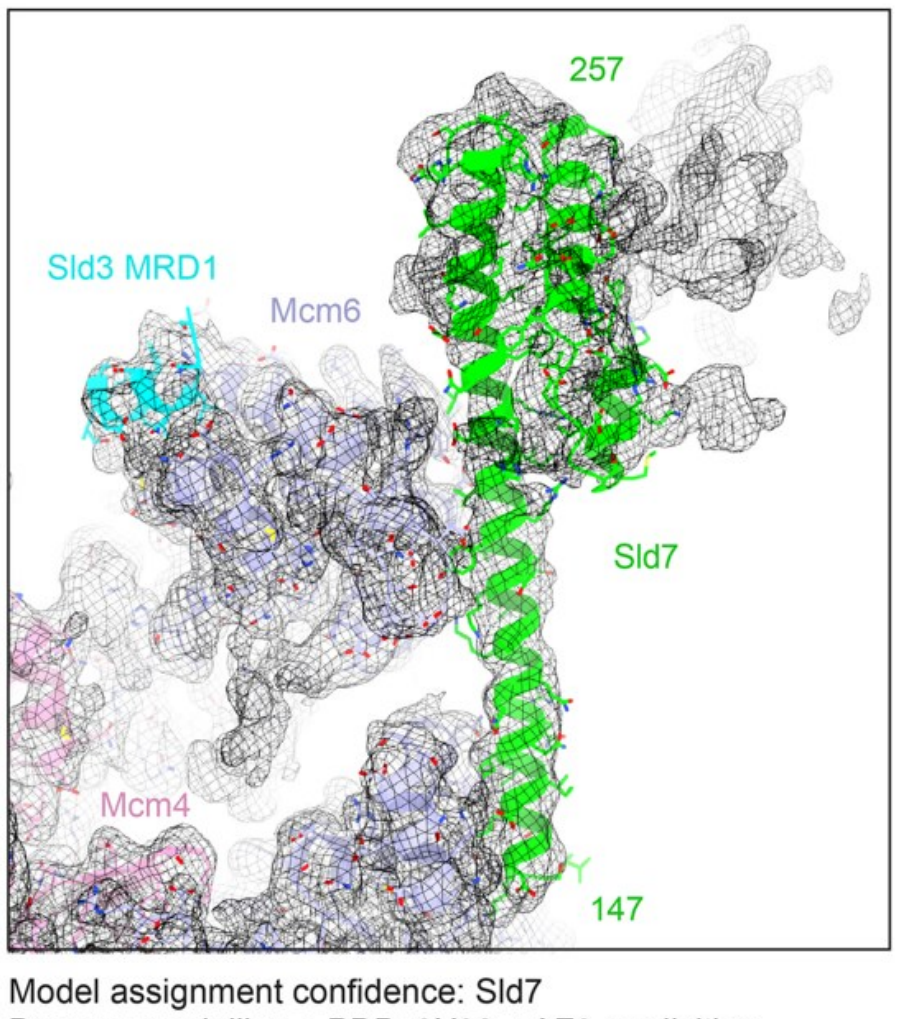

Model assignment confidence: Sld7
De novo modelling + PDB: 3X38 + AF3 predicition

h

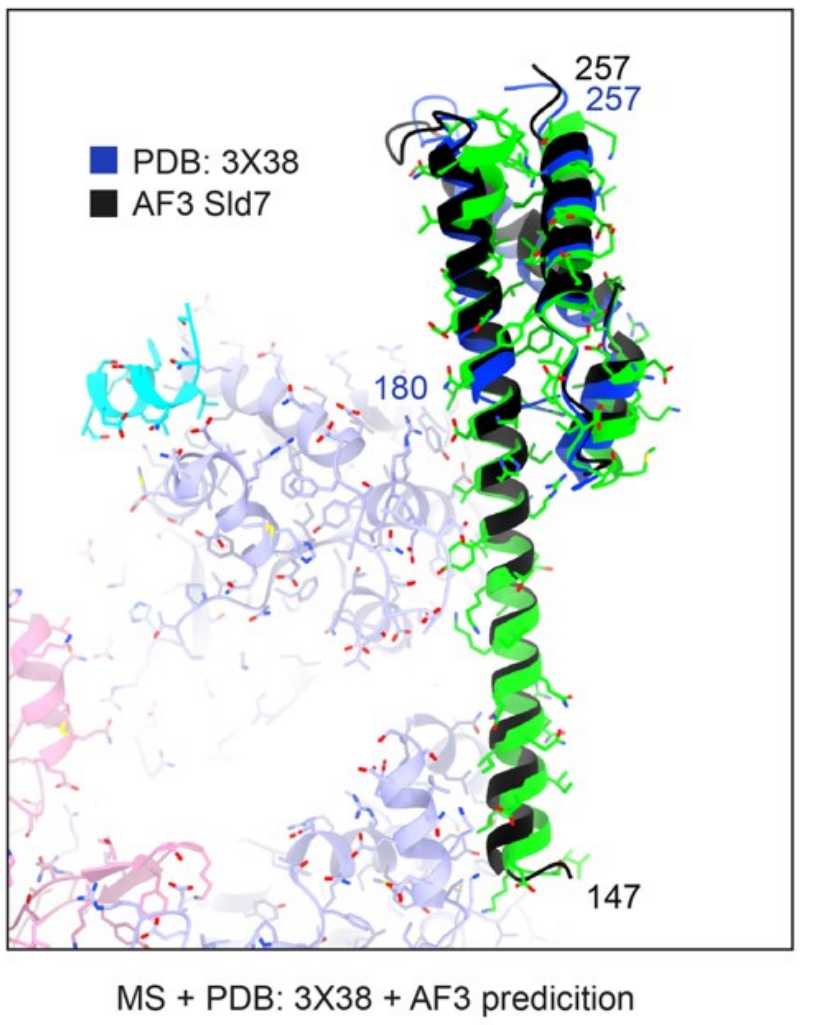

MS + PDB: 3X38 + AF3 predicition

i

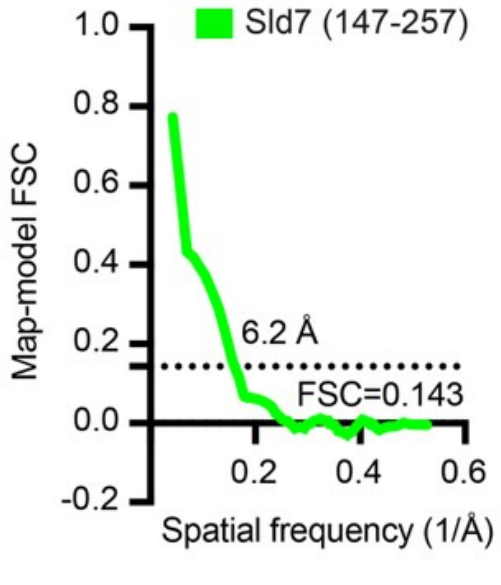

j

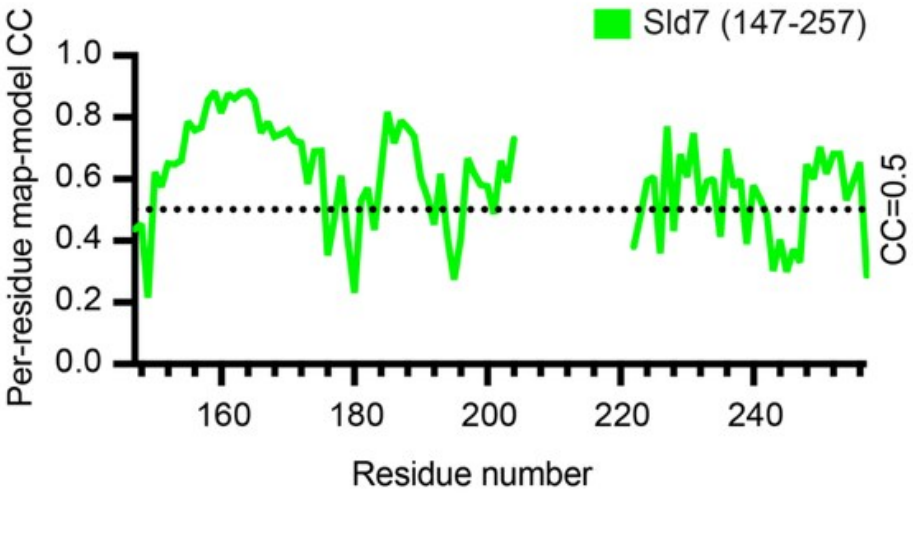

k

Sld7	
CC_mask	0.522
Chain CC	0.517
Main-chain CC	0.557
Side-chain CC	0.514
Mean residue CC	0.599
Median residue CC	0.607

Supplementary Figure 10. Overview of atomic model building and local validation of peripheral Sld3 MRD1/MRD2 and Sld7 assignments in the MS complex.

a-b. Representative density views of (**a**) Sld3 MRD2/MRD2' and (**b**) Sld3 MRD1/MRD1'. Sld3 is shown in cyan with neighbouring MCM subunits coloured as indicated. The placement of MRD2/MRD2' is supported by de novo model building and prior mutational analysis, whereas MRD1/MRD1' is supported by de novo model building and AF3 guided placement. **c**. Zoomed view showing the positions of Sld3 MRD1 and MRD2 in the MS structure, overlaid with the AlphaFold3 prediction. The AF3 prediction model places MRD2 at the Mcm4 interface and MRD1 at the Mcm6 interface. **d-e**. Per-residue map-model correlation coefficients for (**d**) Sld3 MRD1 residues 497-507 and (**e**) Sld3 MRD2 residues 522-539. The dotted line indicates CC = 0.5 as a visual reference. **f**. Summary of map-model statistics for Sld3 MRD1/2. **g**. Representative density view of Sld7 (DD/MBH/hinge). Sld7 is shown in green with neighbouring MCM subunits coloured as indicated. **h**. Comparison of modelled Sld7 with PDB: 3X38 (Sld7 DD) and the AlphaFold3 prediction, supporting the overall assignment of the Sld7 fold within the peripheral density. **i**. Boxed map-model FSC for Sld7 residues 147-257. The curve crosses the FSC = 0.143 threshold at approximately 6.2 Å. **j**. Per-residue map-model correlation coefficients for Sld7 residues 147-257. The discontinuity corresponds to the missing/unmodeled segment. The dotted line indicates CC = 0.5 as a visual reference. **k**. Summary of map-model statistics for Sld7. All local map-model correlation coefficient values were calculated from boxed maps, derived from the composite refinement map, and corresponding boxed models using phenix.map_correlations with an 8 Å box cushion and a 3.6 Å resolution cutoff corresponding to the FSC(model)=0.5 estimate from the Phenix validation of the entire composite map. EM density maps shown were enhanced using CryoSAMU. Together, these analyses support only the approximate placement of Sld3 MRD1/MRD2 and Sld7 in the MS map. For the Sld3 MRDs in particular, the density is best interpreted at backbone/placement level, without implying side-chain or sequence-register certainty. Source data are provided as a Source Data file.

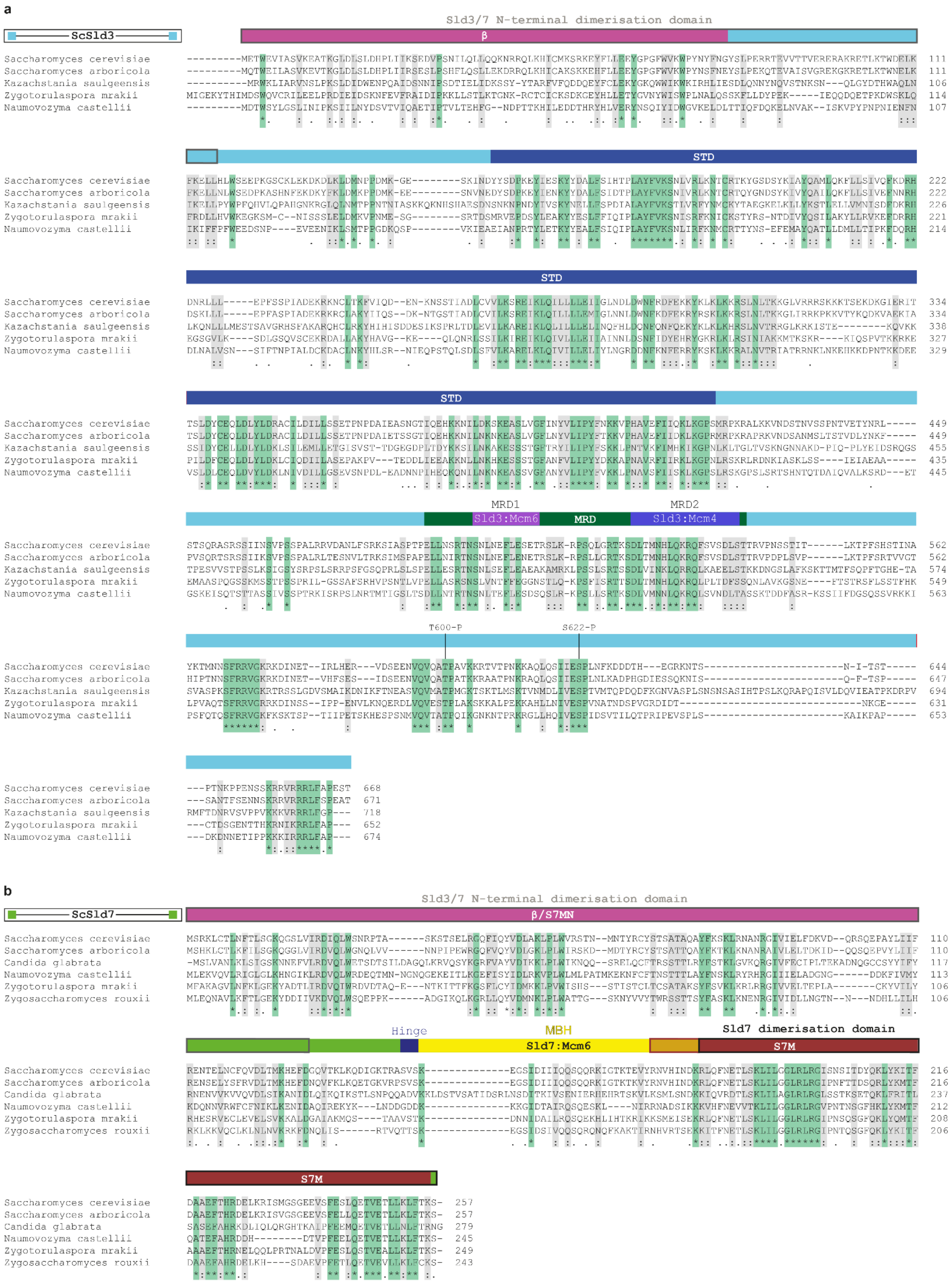
a
Sld3/7 N-terminal dimerisation domain
ScSld3
β
STD
MRD1
MRD2
Sld3:Mcm6
MRD
Sld3:Mcm4
T600-P
S622-P
Saccharomyces cerevisiae
Saccharomyces arboricola
Kazachstania saulgeensis
Zygotorulaspora mrakii
Naumovozyma castellii
b
Sld3/7 N-terminal dimerisation domain
ScSld7
β/S7MN
Hinge
MBH
Sld7:Mcm6
Sld7 dimerisation domain
S7M
Saccharomyces cerevisiae
Saccharomyces arboricola
Candida glabrata
Naumovozyma castellii
Zygotorulaspora mrakii
Zygosaccharomyces rouxii

Supplementary Figure 11. Multiple sequence alignment of Sld3/7 homologs.

a. Sld3 and **b.** Sld7 homologs from a range of species aligned using Clustal Omega. ScSld3/7 domain boundaries are indicated above the respective sequence. Highly conserved residues are colored with a vertical green line and indicated by the symbol (*), highly similar residues are colored with a vertical grey line and indicated by the symbol (:), and residues with similar properties are indicated by the symbol (.). Sld3 and Sld7 contain the following domains; Sld3/7 N-terminal dimerization (S3/7 ND) domain, Sld3-Treslin domain (STD), MCM recognition domain (MRD) which includes the Mcm4 short interaction region (Sld3 Mcm4 SIR) and the MCM6 short interaction region (Sld3 Mcm6 SIR), Ku70-like β-barrel (β) domain or Sld7-MTBP N-terminal (S7MN) domain, Sld7 dimerization domain (S7 DD), Sld7 hinge domain, Sld7 Mcm6 short interaction region (Sld7 Mcm6 SIR), and the Sld7-MTBP C-terminal (S7M) domain. S-CDK phosphorylation sites on Sld3 (aa. 600 and 622) are shown in (**a**).

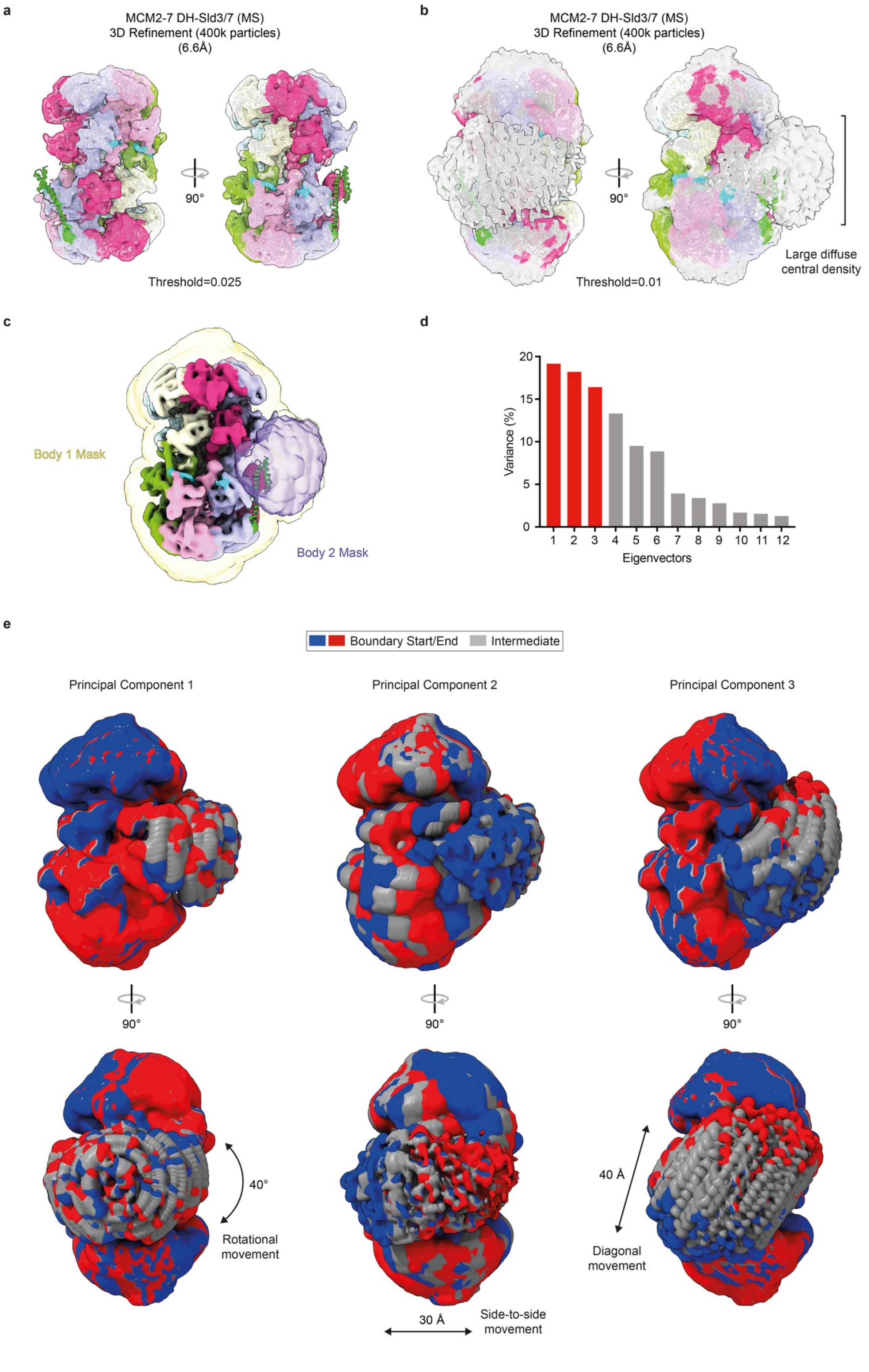
a
MCM2-7 DH-Sld3/7 (MS)
3D Refinement (400k particles)
(6.6Å)
90°
Threshold=0.025
b
MCM2-7 DH-Sld3/7 (MS)
3D Refinement (400k particles)
(6.6Å)
90°
Threshold=0.01
Large diffuse
central density
c
Body 1 Mask
Body 2 Mask
d
Variance (%)
Eigenvectors
e
Boundary Start/End
Intermediate
Principal Component 1
Principal Component 2
Principal Component 3
90°
40°
Rotational
movement
30 Å
Side-to-side
movement
40 Å
Diagonal
movement

Supplementary Figure 12. Multi-body refinement and flexible analysis of MS conformational heterogeneity.

a-b. 3D auto-refined map of the MCM2-7 DH-Sld3/7 (MS) complex, used as input for subsequent multi-body refinement, shown at two contour levels. The lower threshold map in (**b**) reveals a large diffuse central density at the N-lobe of the double hexamer (DH) near the Mcm2/6 interface. The MS atomic model is fitted into the map to indicate which regions reached higher resolution. **c.** Segmented loose soft masks used in RELION to define Body 1 (DH core and Sld3/7, yellow) and Body 2 (Sld3/7 and associated density, purple) for multi-body refinement and flexible analysis. **d.** Histogram plot of the eigenvectors from the multi-body analysis. The first three principal components (red bars) explain 19.2%, 18.2% and 16.4% of the variance, respectively (53.8% in total). **e.** Conformational changes described by principal components (PC) 1-3. For each component, boundary start and end states are shown in blue and red, with intermediate conformations in grey. Rigid-body fitting of the Sld3/7 body between boundary states indicates that PC1 corresponds to an ~40° rotational pivot with minimal translation, PC2 to a predominantly side-to-side translation of ~30 Å, and PC3 to a diagonal translation of ~40 Å with minimal rotation. Source data are provided as a Source Data file.

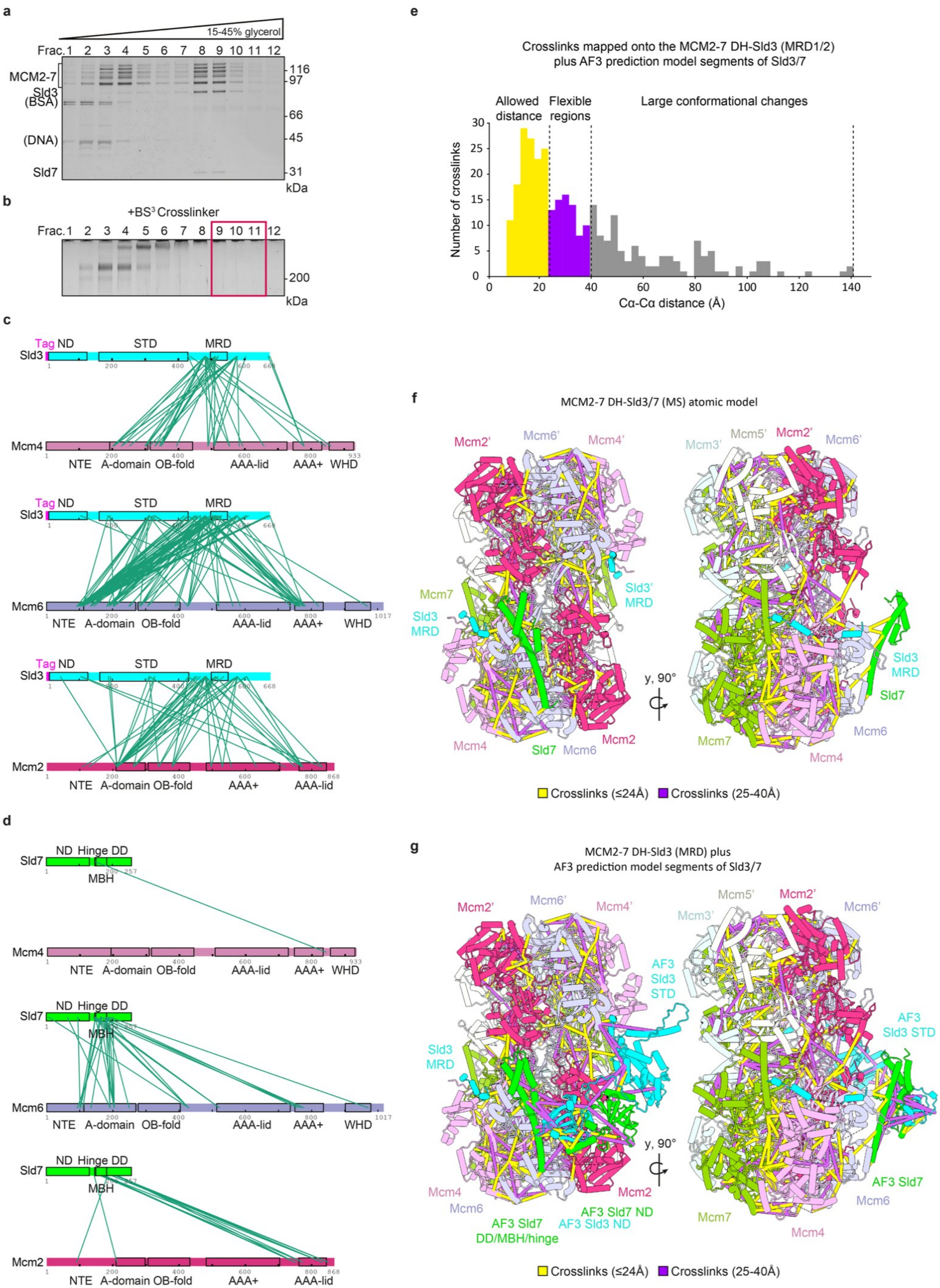
a
15-45% glycerol
Frac. 1 2 3 4 5 6 7 8 9 10 11 12
MCM2-7
Sld3
(BSA)
(DNA)
Sld7
116
97
66
45
31
kDa
b
+BS3 Crosslinker
Frac. 1 2 3 4 5 6 7 8 9 10 11 12
200
kDa
c
Tag ND
STD
MRD
Sld3
Mcm4
NTE A-domain OB-fold AAA-lid AAA+ WHD
Mcm6
NTE A-domain OB-fold AAA-lid AAA+ WHD
Mcm2
NTE A-domain OB-fold AAA+ AAA-lid
d
ND Hinge DD
Sld7
MBH
Mcm4
Mcm6
Mcm2
e
Crosslinks mapped onto the MCM2-7 DH-Sld3 (MRD1/2)
plus AF3 prediction model segments of Sld3/7
Allowed distance
Flexible regions
Large conformational changes
Number of crosslinks
Cα-Cα distance (Å)
f
MCM2-7 DH-Sld3/7 (MS) atomic model
Mcm2'
Mcm6'
Mcm4'
Mcm3'
Mcm5'
Mcm7
Sld3 MRD
Sld3' MRD
Mcm4
Sld7
Mcm6
Mcm2
y, 90°
Crosslinks (≤24Å)
Crosslinks (25-40Å)
g
MCM2-7 DH-Sld3 (MRD) plus
AF3 prediction model segments of Sld3/7
AF3 Sld3 STD
AF3 Sld7
AF3 Sld7 ND
AF3 Sld3 ND
AF3 Sld7 DD/MBH/hinge

Supplementary Figure 13. Chemical crosslinking coupled with mass spectrometry (XL-MS) analysis of the MCM2-7-Sld3/7 complex and structural mapping of crosslinks.

a-b. SDS-PAGE analysis of the MCM2-7 DH-Sld3/7 (MS) complex purified by density gradient ultracentrifugation under (**a**) native conditions (representative of 3 biological repeats) and (**b**) BS^3 crosslinking treatment (representative of one qualitative experiment). The crosslinked fractions 9-11 (indicated by red box) were pooled for XL-MS. **c-d**. 2D domain mapping of inter-crosslinks between (**c**) Sld3 and Mcm2/4/6 and (**d**) Sld7 and Mcm2/4/6. **e**. Histogram of Cα-Cα distances of observed crosslinks measured on the composite MS atomic model shown in (**f**). Most of the crosslinks lie within the allowed distance range (≤24 Å), with outliers attributable to the presence of flexible regions or large conformational changes in the complex. **f**. Crosslinks mapped onto the MS atomic model (derived in this study). Only crosslinks <40 Å are mapped for simplicity. **g**. Crosslinks (<40 Å) mapped onto a composite model built using the MS atomic model (MCM2-7 DH and Sld3 MRD regions) supplemented with AF3 predictions for flexible/missing large Sld3/7 domain regions, rigid-body fitted into a low-resolution EM map (Main Figure 5a). XL-MS data is representative of one biological repeat. Source data are provided as a Source Data file.

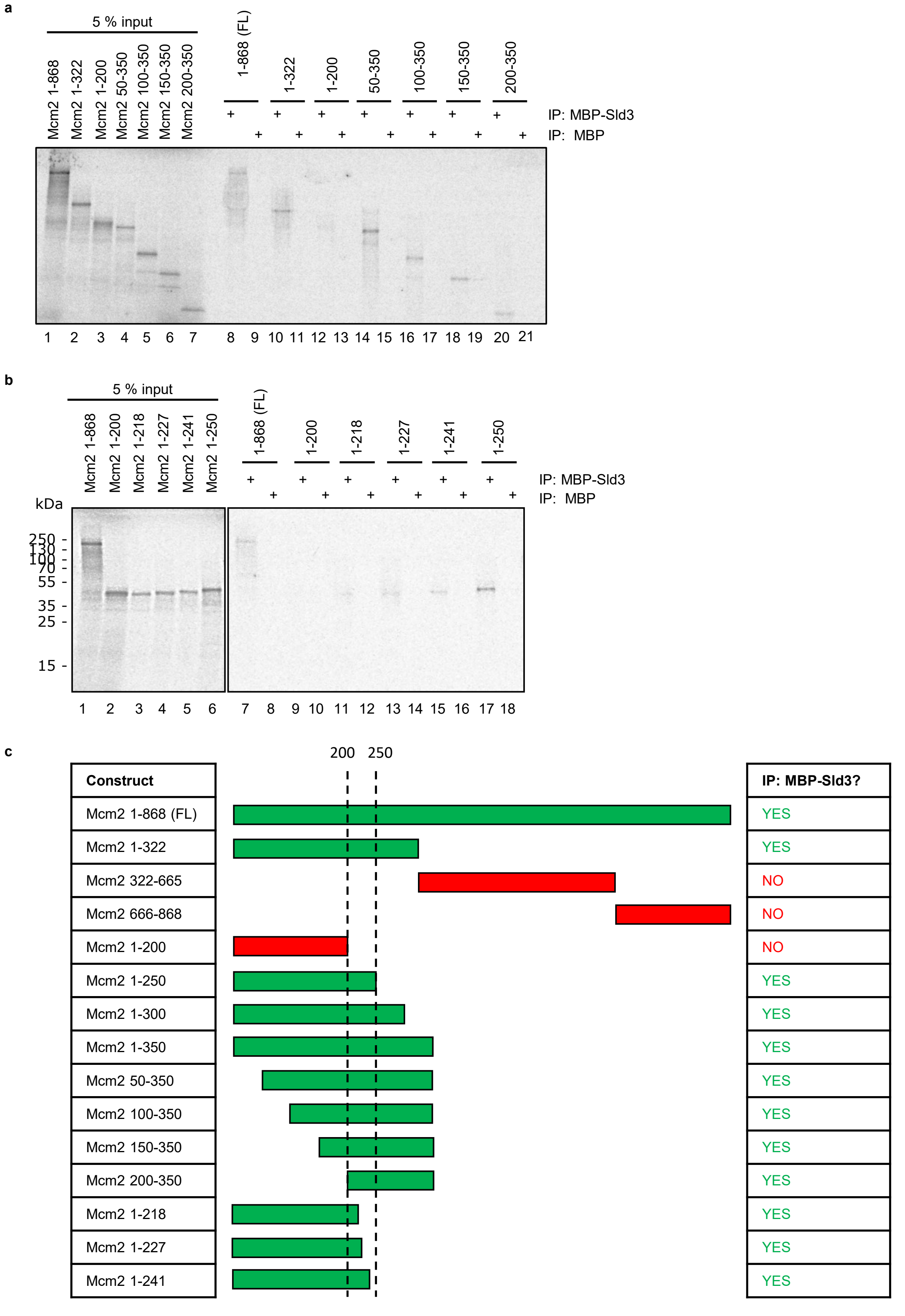

Supplementary Figure 14. Sld3 interacts with the Mcm2 N-terminus.
a. Immunoprecipitation (IP) assay. MBP or MBP-Sld3 were immobilized on anti-MBP magnetic beads and used as bait for ^{35}S labelled *in vitro* transcribed and translated truncations of Mcm2, washed, separated by SDS-PAGE and analyzed by autoradiography. **b.** IP analysis from part (**a**) and Main Figures 5g-h map the Mcm2 binding region of Sld3 to be within residue 200-250 (dashed lines). **c**. Illustration of Mcm2 fragments used in IP assay. The table on the right indicates whether the region can bind Sld3 in the pulldown. Data are representative of three biological repeats. Source data are provided as a Source Data file.

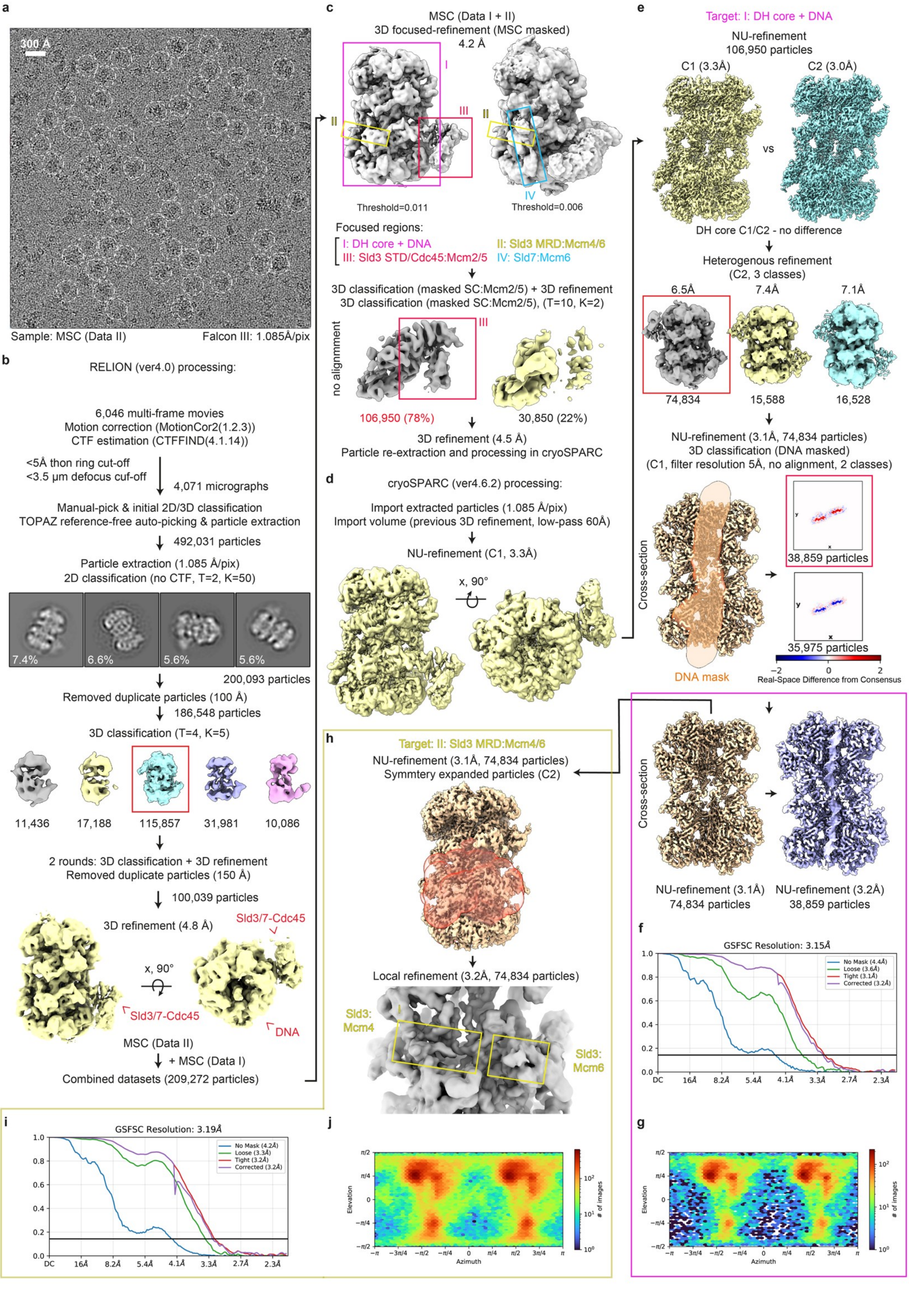
a
300 Å
Sample: MSC (Data II)
Falcon III: 1.085Å/pix
b
RELION (ver4.0) processing:
6,046 multi-frame movies
Motion correction (MotionCor2(1.2.3))
CTF estimation (CTFFIND(4.1.14))
<5Å thon ring cut-off
<3.5 μm defocus cuf-off
4,071 micrographs
Manual-pick & initial 2D/3D classification
TOPAZ reference-free auto-picking & particle extraction
492,031 particles
Particle extraction (1.085 Å/pix)
2D classification (no CTF, T=2, K=50)
7.4%
6.6%
5.6%
5.6%
200,093 particles
Removed duplicate particles (100 Å)
186,548 particles
3D classification (T=4, K=5)
11,436
17,188
115,857
31,981
10,086
2 rounds: 3D classification + 3D refinement
Removed duplicate particles (150 Å)
100,039 particles
3D refinement (4.8 Å)
Sld3/7-Cdc45
x, 90°
Sld3/7-Cdc45
DNA
MSC (Data II)
+ MSC (Data I)
Combined datasets (209,272 particles)
c
MSC (Data I + II)
3D focused-refinement (MSC masked)
4.2 Å
I
II
III
IV
Threshold=0.011
Threshold=0.006
Focused regions:
I: DH core + DNA
II: Sld3 MRD:Mcm4/6
III: Sld3 STD/Cdc45:Mcm2/5
IV: Sld7:Mcm6
3D classification (masked SC:Mcm2/5) + 3D refinement
3D classification (masked SC:Mcm2/5), (T=10, K=2)
no alignment
106,950 (78%)
30,850 (22%)
3D refinement (4.5 Å)
Particle re-extraction and processing in cryoSPARC
d
cryoSPARC (ver4.6.2) processing:
Import extracted particles (1.085 Å/pix)
Import volume (previous 3D refinement, low-pass 60Å)
NU-refinement (C1, 3.3Å)
x, 90°
e
Target: I: DH core + DNA
NU-refinement
106,950 particles
C1 (3.3Å)
C2 (3.0Å)
vs
DH core C1/C2 - no difference
Heterogenous refinement
(C2, 3 classes)
6.5Å
7.4Å
7.1Å
74,834
15,588
16,528
NU-refinement (3.1Å, 74,834 particles)
3D classification (DNA masked)
(C1, filter resolution 5Å, no alignment, 2 classes)
Cross-section
DNA mask
38,859 particles
35,975 particles
Real-Space Difference from Consensus
Cross-section
NU-refinement (3.1Å)
74,834 particles
NU-refinement (3.2Å)
38,859 particles
f
GSFSC Resolution: 3.15Å
No Mask (4.4Å)
Loose (3.6Å)
Tight (3.1Å)
Corrected (3.2Å)
DC 16Å 8.2Å 5.4Å 4.1Å 3.3Å 2.7Å 2.3Å
g
Elevation
Azimuth
# of images
h
Target: II: Sld3 MRD:Mcm4/6
NU-refinement (3.1Å, 74,834 particles)
Symmtery expanded particles (C2)
Local refinement (3.2Å, 74,834 particles)
Sld3: Mcm4
Sld3: Mcm6
i
GSFSC Resolution: 3.19Å
No Mask (4.2Å)
Loose (3.3Å)
Tight (3.2Å)
Corrected (3.2Å)
DC 16Å 8.2Å 5.4Å 4.1Å 3.3Å 2.7Å 2.3Å
j
Elevation
Azimuth
# of images

Supplementary Figure 15. Part 1 of 2: Cryo-EM image processing workflow for 3D reconstruction of the MCM2-7 DH-Sld3/7-Cdc45 (MSC) complex bound to DNA.

a. Representative cryo-EM micrograph showing monodisperse particles, indicated by dotted white circles. (n=1). **b.** Overview of the image processing workflow for 3D classification, separation of distinct MCM2-7 DH-like particle populations, including a dominant C2 symmetric MSC-like class. The particles within an MSC-like class separated by 3D classification (MSC data **II**) were combined with another data set (MSC data **I**) to increase the number of MSC particles. **c.** 3D refinement of MSC particles from data **I** and **II** using a tight mask encompassing the entire complex (focused refinement) yielded an MSC map at 4.2 Å resolution. Because of particle flexibility and the limited particle number, subsequent processing focused on individual regions of interest to improve local density and assemble a more detailed picture of the interactions. The focused processing strategy was divided into four regions (**I**-**IV**): **I**) core DH and central channel DNA; **II**) Sld3 MRD:Mcm4/6 interface; **III**) Sld3 STD/Cdc45:Mcm2/5 interface; and **IV**) Sld7:Mcm6 interface. The consensus set of particles, from which subsequent focused maps were derived, featured at least one molecule of Sld3/7-Cdc45. **d.** Downstream MSC data processing workflow performed in cryoSPARC (ver4.6.2). **e.** Processing strategy used to improve the resolution of the core DH and central DNA channel. Maps before/after focused processing are shown for comparison. **f.** Gold-standard Fourier shell correlation (GSFSC) curves for the post-processed MSC core DH and DNA map shown in (**e**), calculated with and without a loose soft mask and with a B-factor of -116 applied. **g.** Viewing direction distribution plot corresponding to MSC core DH and DNA map (purple map shown in (**e**)). **h.** Processing strategy used to improve the Sld3 MRD:Mcm4/6 interface. **i.** GSFSC curves for the post-processed MSC Sld3 MRD:Mcm4/6 focused map shown in (h), calculated with/without a loose soft mask and with a B-factor of -123 applied. **j.** Viewing direction distribution plot corresponding to the MSC Sld3 MRD:Mcm4/6 focused map (grey map shown in (**h**)). Source data are provided as a Source Data file.

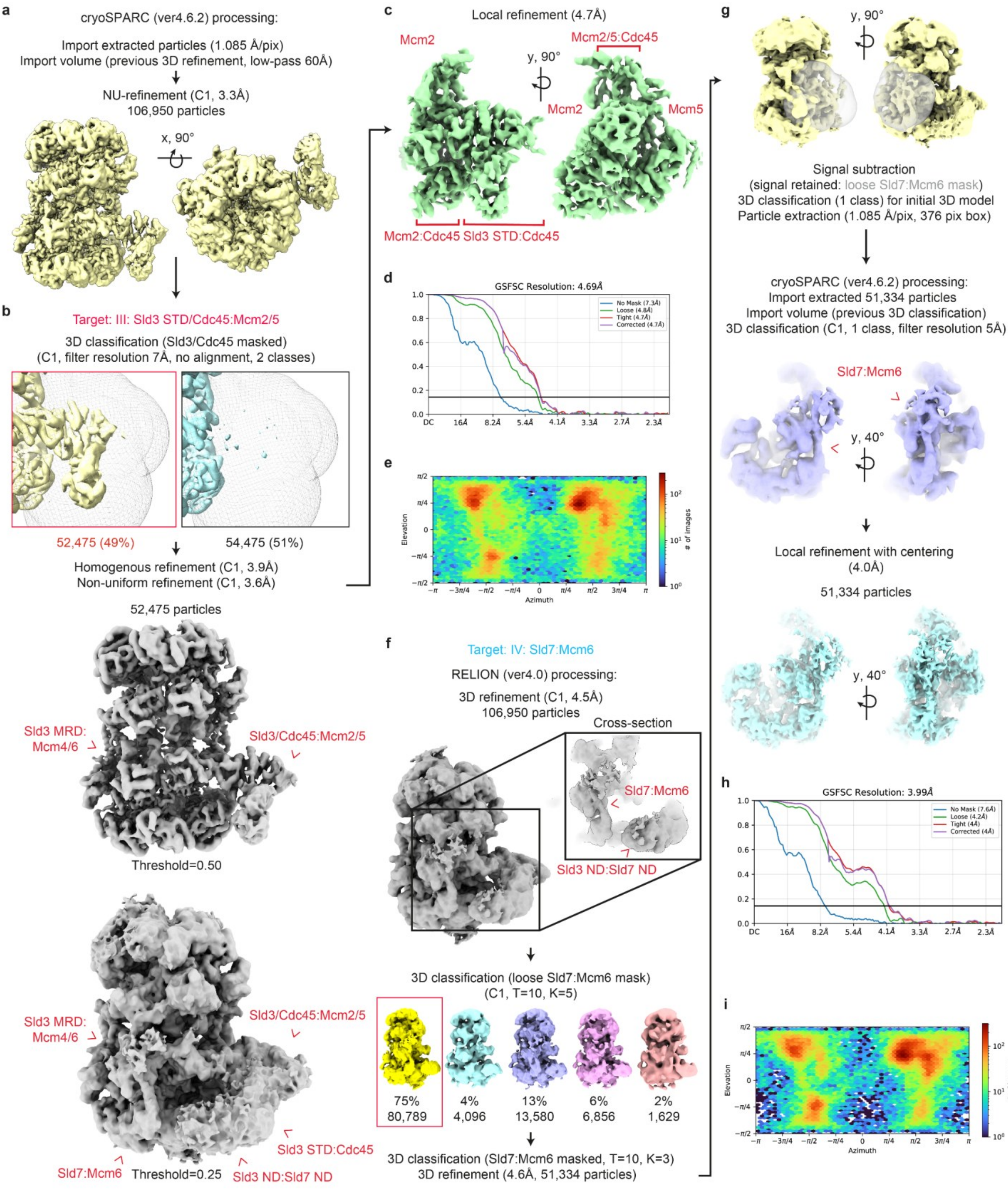
a
cryoSPARC (ver4.6.2) processing:
Import extracted particles (1.085 Å/pix)
Import volume (previous 3D refinement, low-pass 60Å)
NU-refinement (C1, 3.3Å)
106,950 particles
x, 90°
b
Target: III: Sld3 STD/Cdc45:Mcm2/5
3D classification (Sld3/Cdc45 masked)
(C1, filter resolution 7Å, no alignment, 2 classes)
52,475 (49%)
54,475 (51%)
Homogenous refinement (C1, 3.9Å)
Non-uniform refinement (C1, 3.6Å)
52,475 particles
Sld3 MRD: Mcm4/6
Sld3/Cdc45:Mcm2/5
Threshold=0.50
Sld3 MRD: Mcm4/6
Sld3/Cdc45:Mcm2/5
Sld3 STD:Cdc45
Sld7:Mcm6
Threshold=0.25
Sld3 ND:Sld7 ND
c
Local refinement (4.7Å)
Mcm2
Mcm2/5:Cdc45
y, 90°
Mcm2
Mcm5
Mcm2:Cdc45
Sld3 STD:Cdc45
d
GSFSC Resolution: 4.69Å
No Mask (7.3Å)
Loose (4.8Å)
Tight (4.7Å)
Corrected (4.7Å)
e
Elevation
Azimuth
# of images
f
Target: IV: Sld7:Mcm6
RELION (ver4.0) processing:
3D refinement (C1, 4.5Å)
106,950 particles
Cross-section
Sld7:Mcm6
Sld3 ND:Sld7 ND
3D classification (loose Sld7:Mcm6 mask)
(C1, T=10, K=5)
75%
80,789
4%
4,096
13%
13,580
6%
6,856
2%
1,629
3D classification (Sld7:Mcm6 masked, T=10, K=3)
3D refinement (4.6Å, 51,334 particles)
g
y, 90°
Signal subtraction
(signal retained: loose Sld7:Mcm6 mask)
3D classification (1 class) for initial 3D model
Particle extraction (1.085 Å/pix, 376 pix box)
cryoSPARC (ver4.6.2) processing:
Import extracted 51,334 particles
Import volume (previous 3D classification)
3D classification (C1, 1 class, filter resolution 5Å)
Sld7:Mcm6
y, 40°
Local refinement with centering
(4.0Å)
51,334 particles
y, 40°
h
GSFSC Resolution: 3.99Å
No Mask (7.6Å)
Loose (4.2Å)
Tight (4Å)
Corrected (4Å)
i
Elevation
Azimuth
# of images

Supplementary Figure 16. Part 2 of 2: Cryo-EM image processing workflow for 3D reconstruction of the MCM2-7 DH-Sld3/7-Cdc45 complex bound to DNA.

a. Downstream data-processing workflow for MSC. The MSC consensus particle dataset of 106,950 particles and non-uniform refinement (C1, 3.3 Å,) was used as the starting reference for focused classification. **b.** Focused 3D classification of the Sld3 STD/Cdc45:Mcm2/5 region. Particles were classified in C1 into two classes using a soft/loose mask around Sld3/Cdc45 and Mcm2/5. The class with stronger Sld3 STD/Cdc45 (52,475 particles) was subjected to homogeneous and non-uniform refinement. Representative views at different thresholds illustrate the segregation of density for Sld3 MRD:Mcm4/6, Sld3 STD/Cdc45:Mcm2/5 and Sld7:Mcm6 and Sld3/7 N-terminal dimerization (ND) domains. **c.** Local refinement of the Sld3 STD/Cdc45:Mcm2/5 interface (4.7Å) was performed using a focused, loose mask around the subunits, resolving the protein interfaces. A manually generated loose mask, around the Sld3 STD/Cdc45:Mcm2/5 segment in RELION yielded a post-processed map with a resolution of 5.2Å. **d.** Gold-standard Fourier shell correlation curves for the locally refined Sld3 STD/Cdc45:Mcm2/5 map shown in (**c**) with a mean resolution of 4.69 Å and with a B-factor of -210 applied. **e.** Viewing direction distribution for the Sld3 STD/Cdc45:Mcm2/5 local refinement shown in (**c**). **f.** Focused processing of the Sld7:Mcm6 interface in RELION. The consensus MSC reconstruction (C1, 4.5 Å, 106,950 particles) was subjected to 3D classification with a loose mask around Sld7 and Mcm6 (T=10, K=5), yielding one class with well-defined Sld7 density. This subset (51,334 particles) was refined (C1, 4.6 Å) and then re-classified (T=10, K=3) with an Sld7:Mcm6 mask to enrich for particles with Sld7 bound. **g.** Signal-subtraction retaining the Sld7:Mcm6 region and neighboring density, followed by import of the subtracted particles into cryoSPARC for 3D classification and local refinement with centering. The final Sld7:Mcm6 map (C1, 4.0 Å, 51,334 particles) reveals Sld7 docking on the Mcm6 N-terminal and C-terminal domains. **h.** GSFSC curves for the Sld7:Mcm6 local refinement in (**g**) reporting a mean resolution of 4 Å and with a B-factor of -50 applied. **i.** Viewing direction distribution plot for the locally refined Sld7:Mcm6 reconstruction shown in (**g**). Source data are provided as a Source Data file.

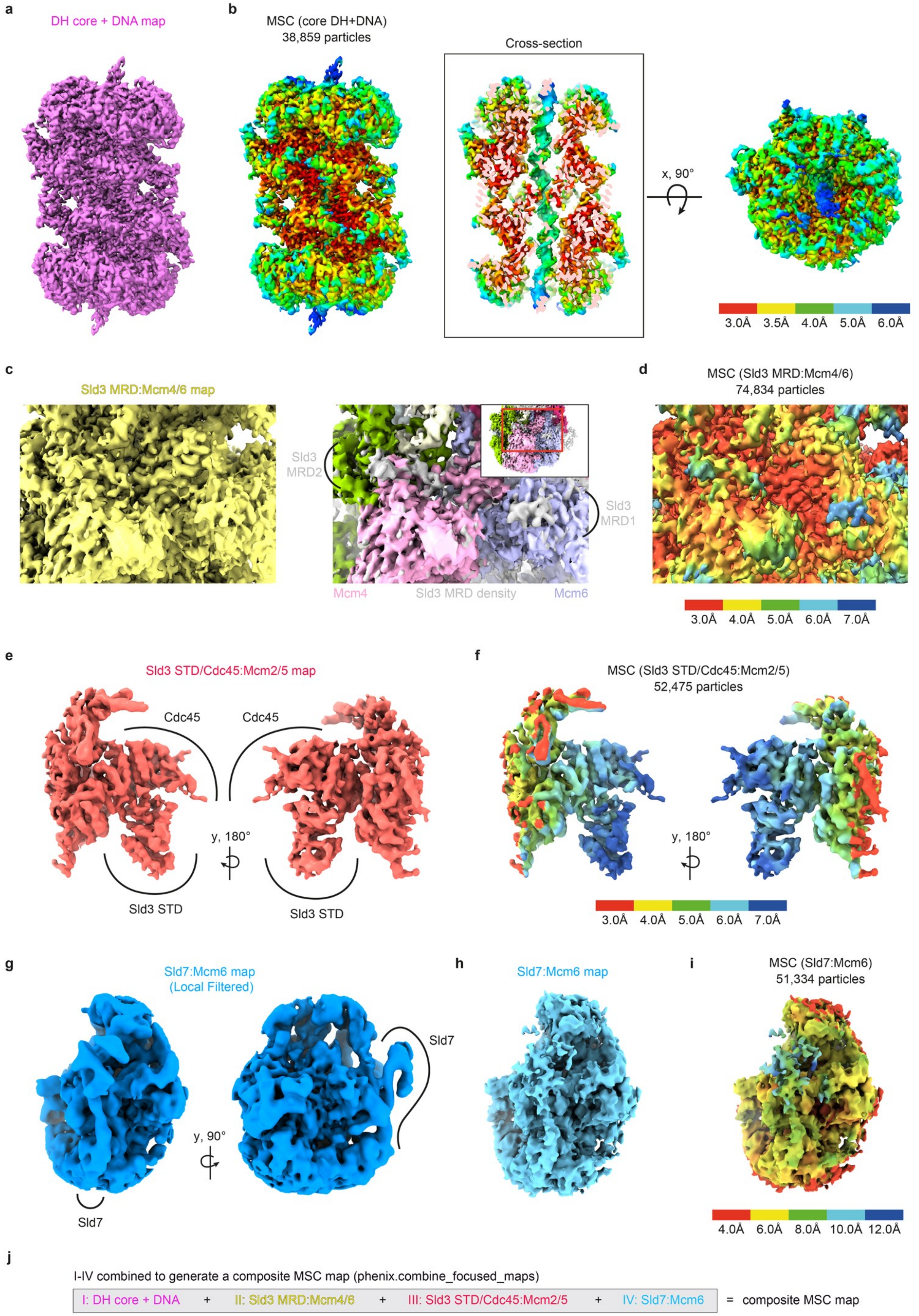
a
DH core + DNA map
b
MSC (core DH+DNA)
38,859 particles
Cross-section
x, 90°
3.0Å 3.5Å 4.0Å 5.0Å 6.0Å
c
Sld3 MRD:Mcm4/6 map
Sld3 MRD2
Sld3 MRD1
Mcm4
Sld3 MRD density
Mcm6
d
MSC (Sld3 MRD:Mcm4/6)
74,834 particles
3.0Å 4.0Å 5.0Å 6.0Å 7.0Å
e
Sld3 STD/Cdc45:Mcm2/5 map
Cdc45
Cdc45
y, 180°
Sld3 STD
Sld3 STD
f
MSC (Sld3 STD/Cdc45:Mcm2/5)
52,475 particles
y, 180°
3.0Å 4.0Å 5.0Å 6.0Å 7.0Å
g
Sld7:Mcm6 map
(Local Filtered)
Sld7
y, 90°
Sld7
h
Sld7:Mcm6 map
i
MSC (Sld7:Mcm6)
51,334 particles
4.0Å 6.0Å 8.0Å 10.0Å 12.0Å
j
I-IV combined to generate a composite MSC map (phenix.combine_focused_maps)
I: DH core + DNA + II: Sld3 MRD:Mcm4/6 + III: Sld3 STD/Cdc45:Mcm2/5 + IV: Sld7:Mcm6 = composite MSC map

Supplementary Figure 17. Focused maps (regions I-IV) used to generate the composite MSC map.
a-b. Focused map for region I, showing the DH core with the central DNA duplex (3.2 Å, 38,859 particles) and (**b**) a locally filtered map colored by local resolution and shown in cross-section, highlighting the continuous density for the double-stranded DNA within the central channel. **c-d.** Focused map for region II, the Sld3 MRD:Mcm4/6 interface (3.2 Å, 74,834 particles) and (**d**) a locally filtered map colored by local resolution, illustrating how Sld3 MRD engages both Mcm4 and Mcm6. **e.** Focused map for region III, the Sld3 STD/Cdc45:Mcm2/5 interface (4.7 Å, 52,475 particles). In comparison, a manually generated loose mask, around the Sld3 STD/Cdc45:Mcm2/5 segment in RELION yielded a post-processed map with a resolution of 5.2Å. **f** a locally filtered map of (e) colored by local resolution, revealing the architecture of Cdc45 at Mcm2/5 and how this interaction is tethered by the Sld3 STD. **g-i.** Focused maps for region IV, the Sld7:Mcm6 interface (4.0 Å, 51,334 particles), showing (**g**) the locally filtered Sld7:Mcm6 map, (**h**) the corresponding focused local refinement map and (**i**) the same map as in (**h**) colored by local resolution, highlighting Sld7 bound along the N- and C- terminal domains of Mcm6. **j.** Schematic illustrating the combination of the four focused maps (regions I-IV) using phenix.combine_focused_maps to generate a composite MSC map in which the DH core, DNA, Sld3 MRD:Mcm4/6, Sld3 STD/Cdc45:Mcm2/5 and Sld7:Mcm6 interfaces are all represented at their locally optimal resolutions and used for atomic model building.

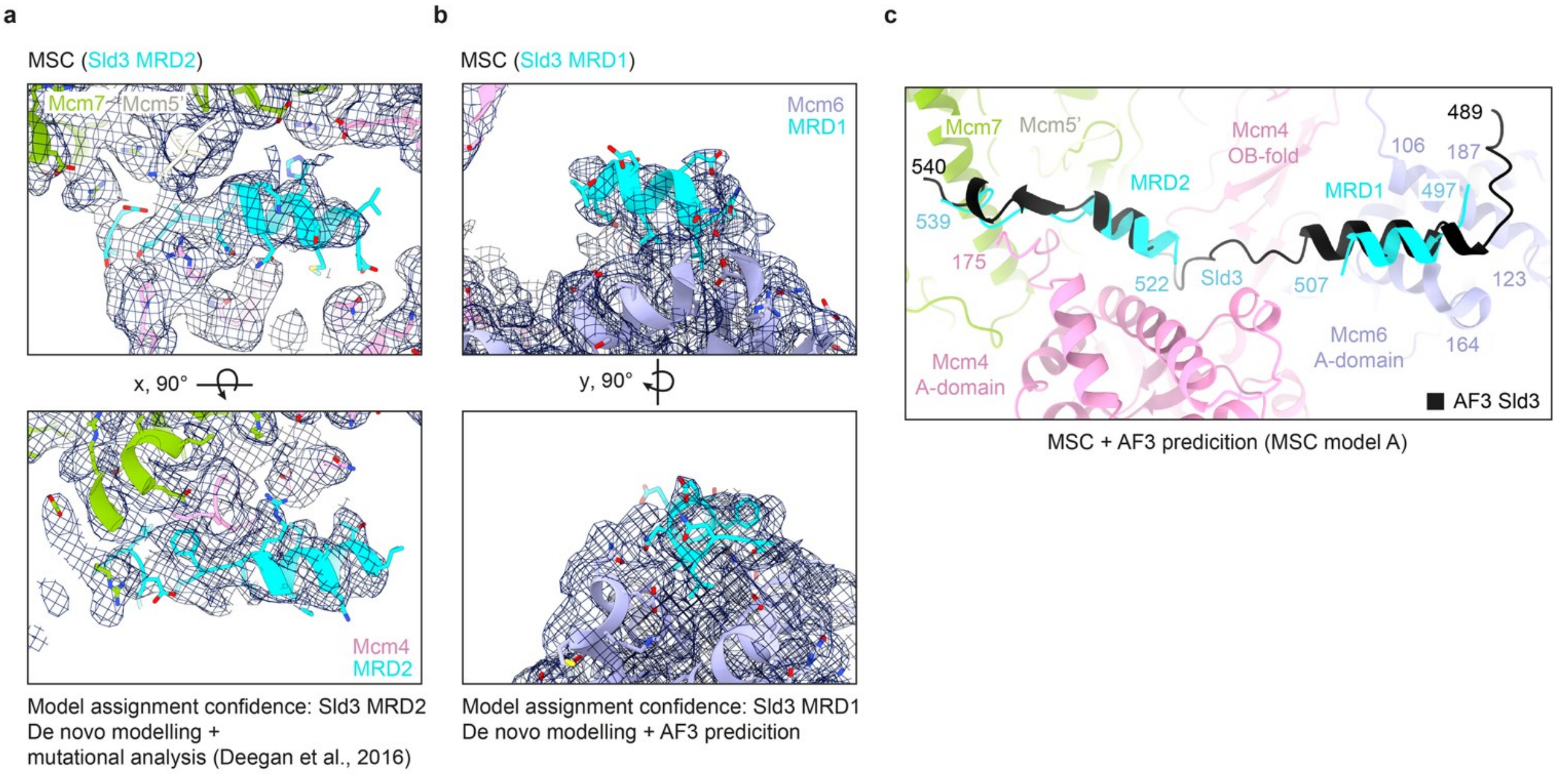

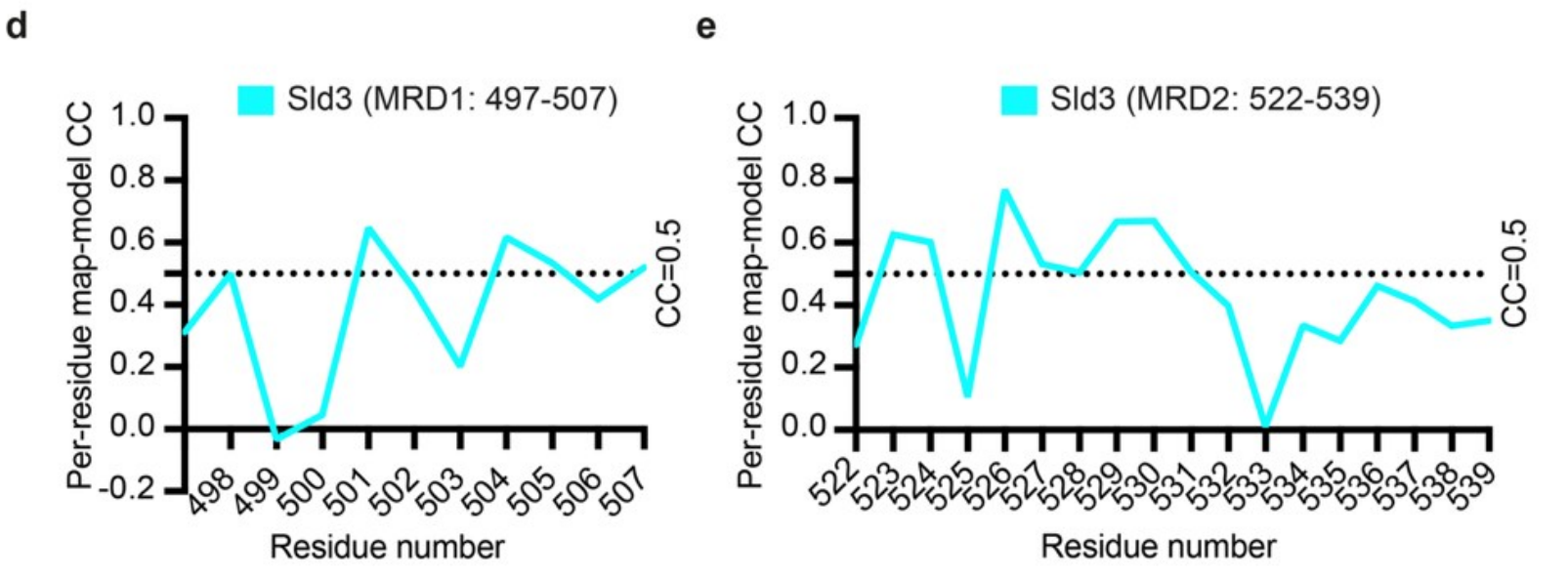

f

Sld3 Region:	MRD1	MRD2
CC_mask	0.414	0.507
Chain CC	0.211	0.256
Main-chain CC	0.411	0.317
Side-chain CC	0.182	0.292
Mean residue CC	0.383	0.436
Median residue CC	0.448	0.437

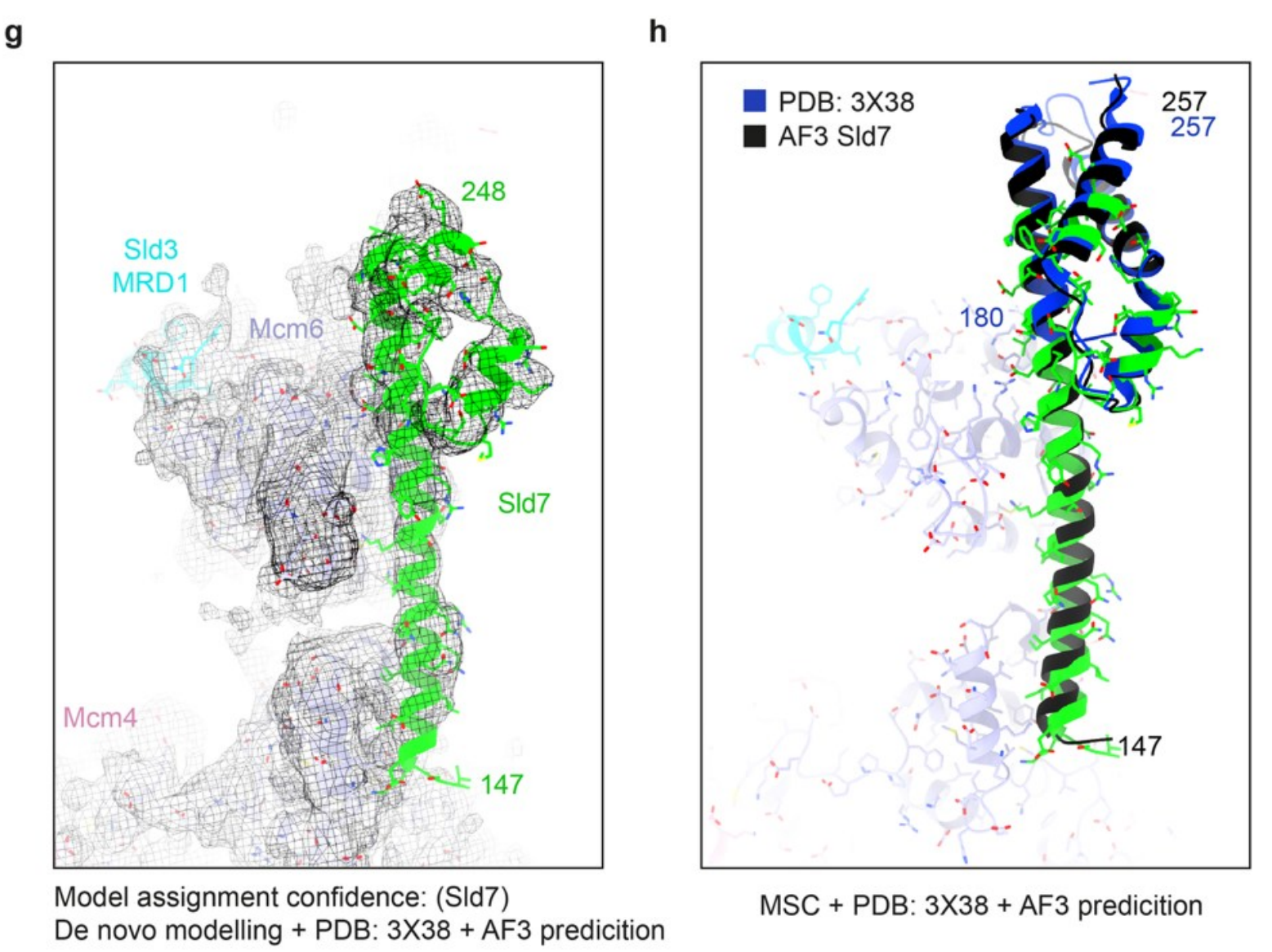

k

Sld7	
CC_mask	0.539
Chain CC	0.547
Main-chain CC	0.574
Side-chain CC	0.531
Mean residue CC	0.607
Median residue CC	0.649

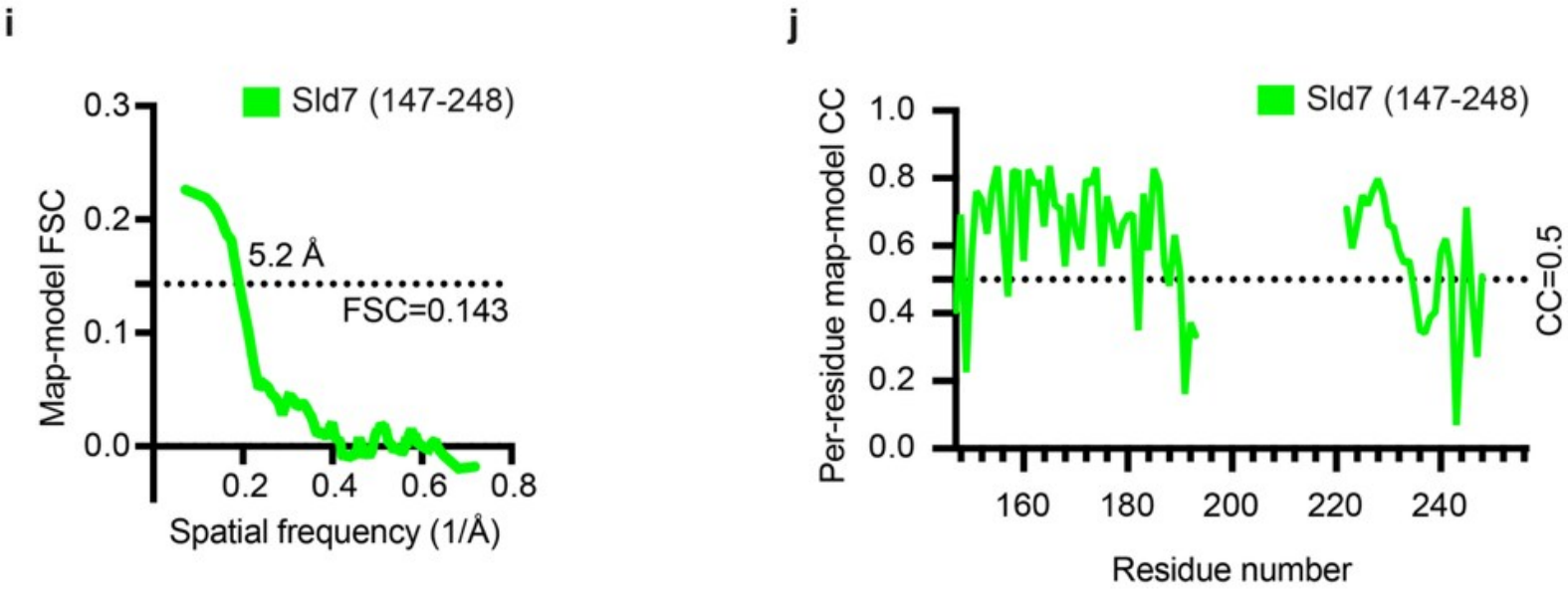

Supplementary Figure 18. Overview of atomic model building and local validation of peripheral Sld3 MRD1/MRD2 and Sld7 assignments in the MSC complex.

a-b. Representative density views of (**a**) Sld3 MRD2 and (**b**) Sld3 MRD1. The placement of MRD2 is supported by de novo model building and prior mutational analysis, whereas MRD1 is supported by de novo model building and AF3 guided placement. **c**. Zoomed view showing the positions of Sld3 MRD1 and MRD2 in the MSC structure, overlaid with the AlphaFold3 prediction. Modelled Sld3 regions are shown in cyan and the AlphaFold3 prediction in black. The AF3 prediction model places MRD2 at the Mcm4 interface and MRD1 at the Mcm6 interface. **d-e**. Per-residue map-model correlation coefficients for (**d**) Sld3 MRD1 residues 497-507 and (**e**) Sld3 MRD2 residues 522-539. The dotted line indicates CC = 0.5 as a visual reference. **f**. Summary of map-model statistics for Sld3 MRD1/2. **g**. Representative density view of Sld7 (DD/MBH/hinge). Sld7 is shown in green with neighbouring MCM subunits coloured as indicated. **h**. Comparison of modelled Sld7 with PDB: 3X38 (Sld7 DD) and the AlphaFold3 prediction, supporting the overall assignment of the Sld7 fold within the peripheral density. **i**. Boxed map-model FSC for Sld7 residues 147-248. The curve crosses the FSC = 0.143 threshold at approximately 5.2 Å. **j**. Per-residue map-model correlation coefficients for Sld7 residues 147-248. The discontinuity corresponds to the missing/unmodeled segment. The dotted line indicates CC = 0.5 as a visual reference. **k**. Summary of map-model statistics for Sld7. All local map-model correlation coefficient values were calculated from boxed maps, derived from the composite refinement map, and corresponding boxed models using phenix.map_correlations with an 8 Å box cushion and a 3.6 Å resolution cutoff corresponding to the FSC(model)=0.5 estimate from the Phenix validation of the entire composite map. EM density maps shown were enhanced using CryoSAMU for visualization. Together, these analyses support only the approximate placement of Sld3 MRD1/MRD2 and Sld7 in the MSC map. For the Sld3 MRDs, the density is best interpreted at backbone/placement level, without implying side-chain or sequence-register certainty. Source data are provided as a Source Data file.

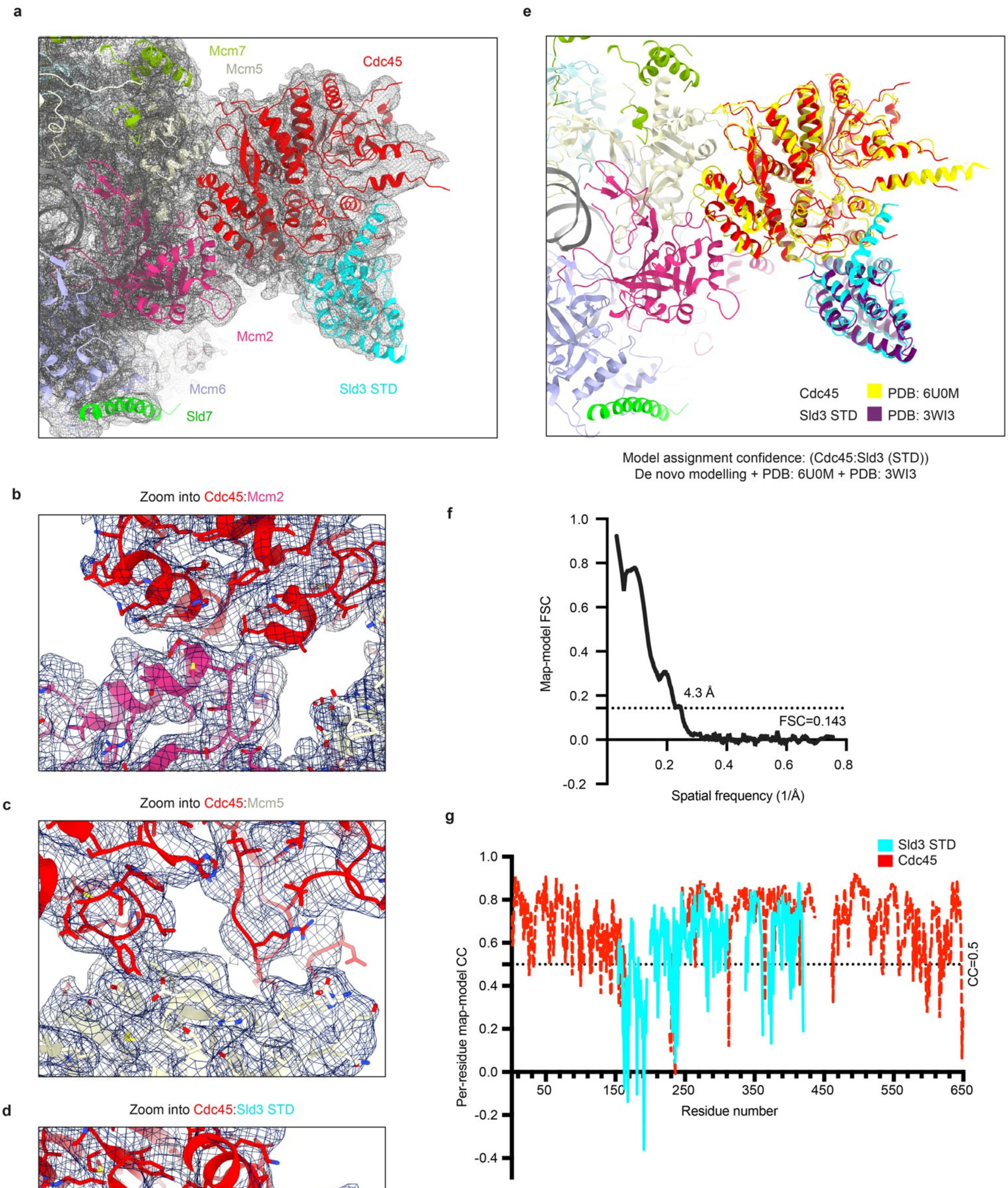

h

Cdc45-Sld3 STD	
CC_mask	0.653
Chain CC	Sld3 STD: 0.602<br>Cdc45: 0.685
Main-chain CC	0.674
Side-chain CC	0.656
Mean residue CC	Sld3 STD: 0.576<br>Cdc45: 0.701
Median residue CC	Sld3 STD: 0.631<br>Cdc45: 0.745

Supplementary Figure 19. Overview of atomic model building and local validation of Cdc45:Sld3 STD assignment in the MSC complex.

a. Representative density view of the Cdc45:Sld3 STD region. Cdc45 is shown in red, Sld3 STD in cyan, Sld7 in green, and neighbouring MCM subunits as indicated. The surrounding cryo-EM density is shown as mesh. **b**. Zoomed density view of the Cdc45:Mcm2 interaction interface. **c**. Zoomed density view of the Cdc45:Mcm5 interaction interface. **d**. Zoomed density view of the Cdc45:Sld3 STD interaction interface. **e**. Structural comparison of the modelled Cdc45:Sld3 STD region with reference structures. Cdc45 is compared with PDB 6U0M and Sld3 STD with PDB 3WI3, supporting assignment of the overall fold and interface geometry. **f**. Boxed map-model FSC for the Cdc45:Sld3 boxed region. The curve crosses the FSC = 0.143 threshold at approximately 4.3 Å. **g**. Per-residue map-model correlation coefficients for the Cdc45:Sld3 boxed region. The discontinuity corresponds to the missing/unmodeled segments. The dotted line indicates CC = 0.5 as a visual reference. **h**. Summary of map-model statistics for Cdc45:Sld3 STD. All local map-model correlation coefficient values were calculated from boxed maps, derived from the composite refinement map, and corresponding boxed models using phenix.map_correlations with an 8 Å box cushion and a 3.6 Å resolution cutoff corresponding to the FSC(model)=0.5 estimate from the Phenix validation of the entire composite map. EM density maps shown were enhanced using CryoSAMU to improve interpretability and generate smoothed maps for visualization. Source data are provided as a Source Data file.

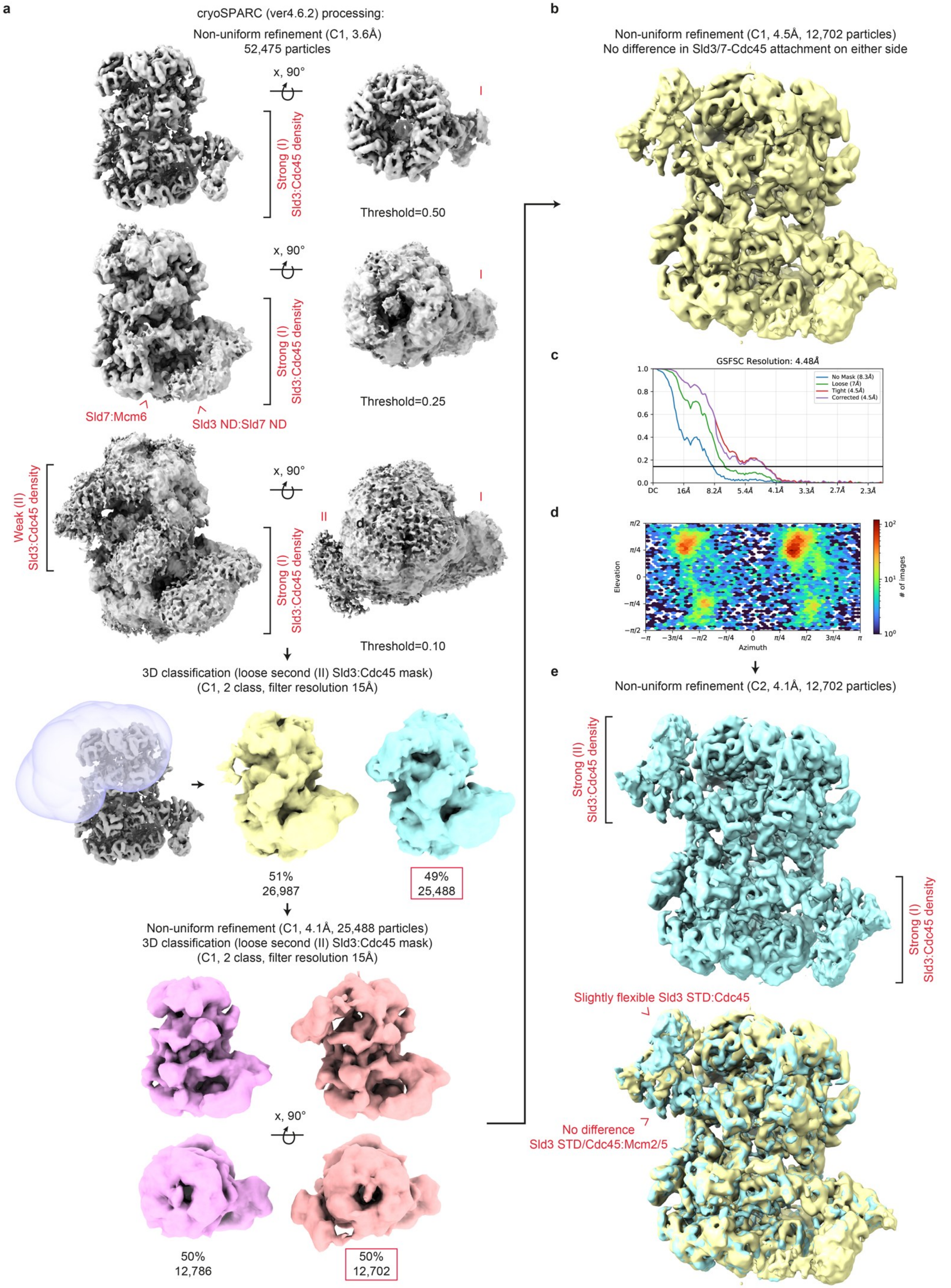

a
cryoSPARC (ver4.6.2) processing:
Non-uniform refinement (C1, 3.6Å)
52,475 particles
x, 90°
Strong (I) Sld3:Cdc45 density
I
Threshold=0.50
x, 90°
Strong (I) Sld3:Cdc45 density
I
Threshold=0.25
Sld7:Mcm6
Sld3 ND:Sld7 ND
Weak (II) Sld3:Cdc45 density
x, 90°
Strong (I) Sld3:Cdc45 density
II
I
Threshold=0.10
3D classification (loose second (II) Sld3:Cdc45 mask)
(C1, 2 class, filter resolution 15Å)
51%
26,987
49%
25,488
Non-uniform refinement (C1, 4.1Å, 25,488 particles)
3D classification (loose second (II) Sld3:Cdc45 mask)
(C1, 2 class, filter resolution 15Å)
x, 90°
50%
12,786
50%
12,702
b
Non-uniform refinement (C1, 4.5Å, 12,702 particles)
No difference in Sld3/7-Cdc45 attachment on either side
c
GSFSC Resolution: 4.48Å
No Mask (8.3Å)
Loose (7Å)
Tight (4.5Å)
Corrected (4.5Å)
d
Elevation
Azimuth
# of images
e
Non-uniform refinement (C2, 4.1Å, 12,702 particles)
Strong (II) Sld3:Cdc45 density
Strong (I) Sld3:Cdc45 density
Slightly flexible Sld3 STD:Cdc45
No difference
Sld3 STD/Cdc45:Mcm2/5

Supplementary Figure 20. Cryo-EM image processing workflow for 3D reconstruction of an MSC complex with two Sld3/7-Cdc45 molecules bound to the MCM2-7 DH.

a. Non-uniform refinement in cryoSPARC (C1, 3.6 Å, 52,475 particles) of the Sld3 STD/Cdc45 enriched subset from Supplementary Figure 16 revealing strong Sld3/Cdc45 density on one side of the DH and variable density on the opposite side at different map thresholds. Focused 3D classification without alignment using a loose mask around the second Sld3/Cdc45 region (C1, 2 classes, 15 Å filter) separated particles with strong and weak density for this second Sld3/Cdc45 (26,987 and 25,488 particles, respectively). The class with stronger density (25,488 particles) was selected for further refinement. **b.** Non-uniform refinement of the selected class, followed by a second round of focused 3D classification with the same mask (C1, 2 classes, 15 Å filter), yielded two nearly equal classes, one of which retained strong density for Sld3/Cdc45 on both sides of the DH (12,702 particles). This subset refined to a 4.5 Å map (C1, 12,702 particles). **c.** GSFSC curves for the refined reconstruction in (**b**) with a mean resolution of 4.8 Å and with a B-factor of 0 applied. **d.** Viewing direction distribution plot of the map shown in (**b**). **e.** Non-uniform refinement with C2 symmetry applied to the final particle subset (4.5 Å, 12,702 particles), revealing that the two Sld3 STD/Cdc45 subunits adopt essentially identical configurations on opposite sides of the DH with only modest residual flexibility. This analysis supports the conclusion that the MSC complex can accommodate two Sld3/Cdc45 assemblies arranged symmetrically around the MCM2-7 DH. Source data are provided as a Source Data file.

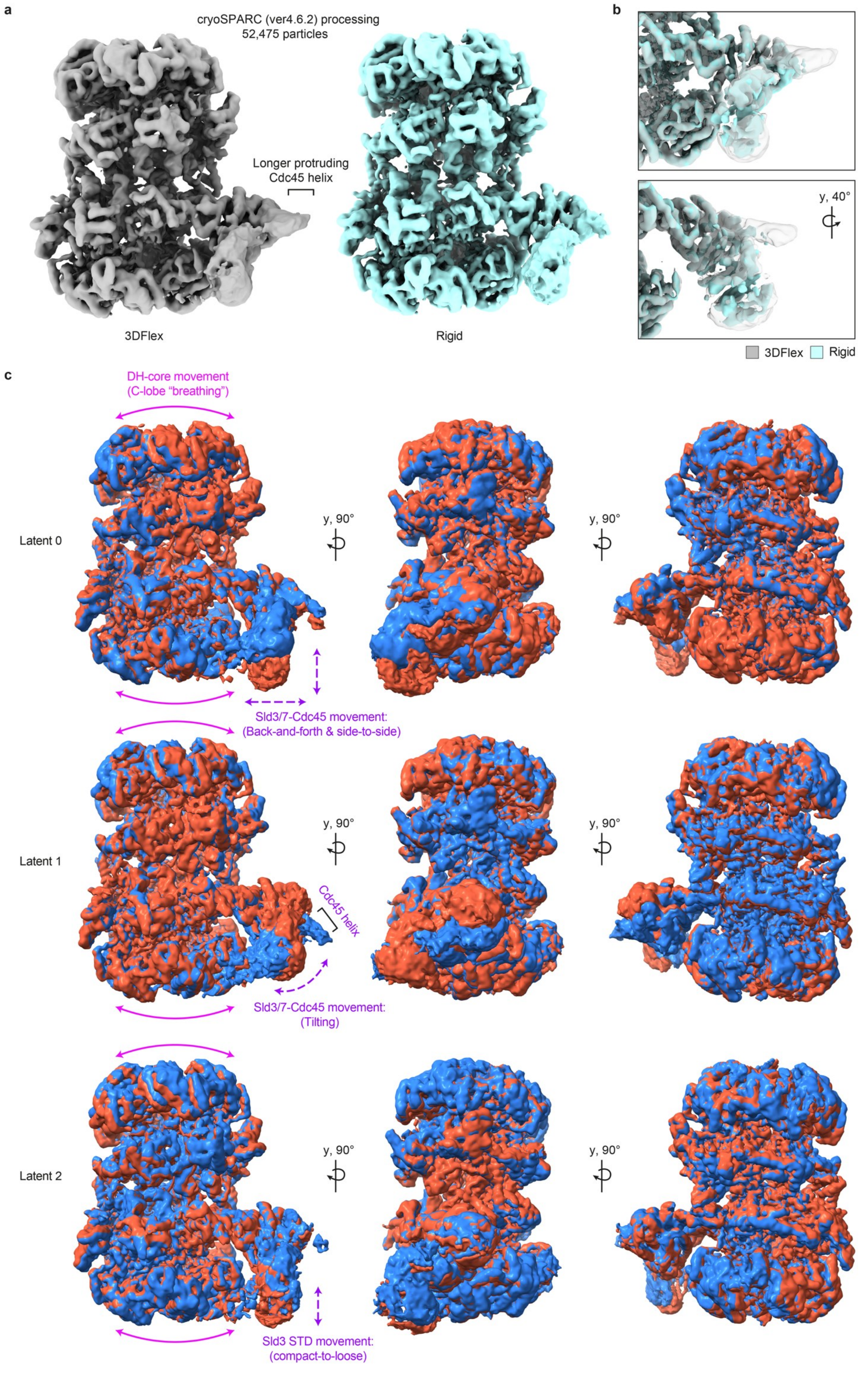
a
cryoSPARC (ver4.6.2) processing
52,475 particles
Longer protruding
Cdc45 helix
3DFlex
Rigid
b
y, 40°
3DFlex
Rigid
c
DH-core movement
(C-lobe "breathing")
Latent 0
y, 90°
y, 90°
Sld3/7-Cdc45 movement:
(Back-and-forth & side-to-side)
Latent 1
y, 90°
y, 90°
Cdc45 helix
Sld3/7-Cdc45 movement:
(Tilting)
Latent 2
y, 90°
y, 90°
Sld3 STD movement:
(compact-to-loose)

Supplementary Figure 21. 3DFlex analysis of MSC conformational heterogeneity.
a. 3D Flexible Refinement (3DFlex) and rigid refinement of the MSC complex, using cryoSPARC. The refinements were performed with a focused subset of 52,475 particles selected for exhibiting density within masks encompassing the Sld3 STD-Cdc45 region (see Supplementary Figure 16). The 3DFlex model resolves additional features, including a striking continuous extension of a protruding long helix that projects outward of Cdc45, consistent with previous yeast CMG (Cdc45 helical segment, aa. 218-240; Yuan et al., 2016; Abid Ali et al., 2016) and human Cdc45 structures (Li et al., 2016)**.** The 3DFlex approach accounts for the broader conformational heterogeneity at the Sld3 STD-Cdc45:Mcm2/5 region, however at a cost of locally reduced resolution for Sld3 STD-Cdc45 compared with the corresponding rigid reconstruction. **b**. Overlay of the 3DFlex and rigid reconstructions highlighting features recovered by 3DFlex motion modelling that are absent from the rigid map. **c**. Latent coordinate trajectories obtained from 3DFlex modelling illustrate coordinated motions across Sld3/7-Cdc45 and the DH core. Three representative latent states (Latent 0-2) are shown. Density maps corresponding to the latent start and end points are colored red and blue, respectively. Latent 0-2 all capture a lateral ‘‘breathing’’ motion for the DH core. Latent 0 captures a back-and-forth and side-to-side displacement of Sld3/7-Cdc45. Latent 1 captures tilting of Sld3/7-Cdc45 and bending of the Cdc45 protruding helix. Latent 2 captures a compact-to-loose transition within the Sld3 STD domain coupled to subtle rearrangements around the Cdc45 interface. Arrows denote the principal directions of motion for each latent trajectory.

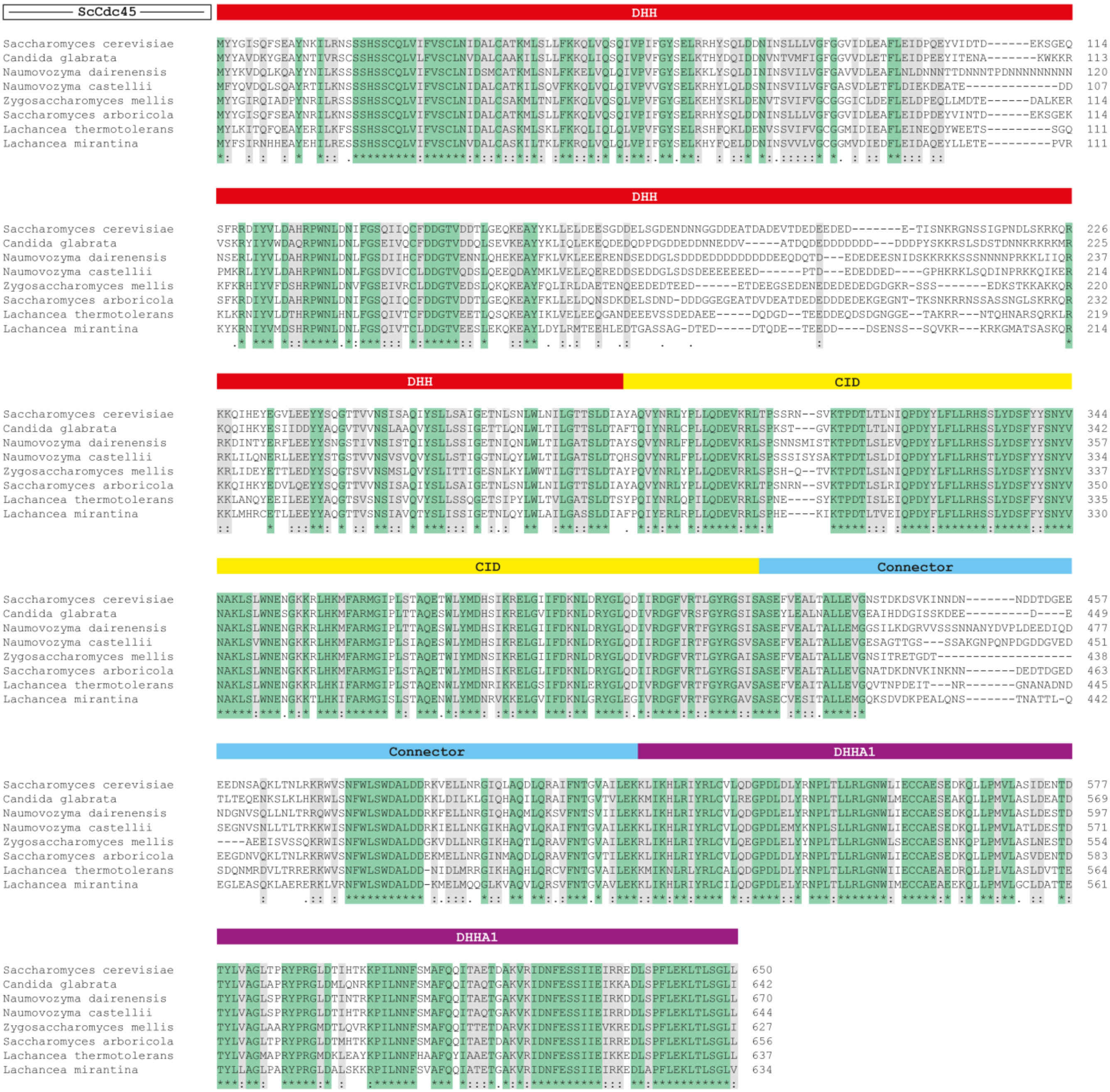

Supplementary Figure 22. Multiple sequence alignment of Cdc45 homologs.
Cdc45 homologs from a range of different species aligned using Clustal Omega. Saccharomyces cerevisiae (Sc)Cdc45 domain boundaries are indicated above the respective sequence. Highly conserved residues are colored with a vertical green line and indicated by the symbol (*), highly similar residues are colored with a vertical grey line and indicated by the symbol (:), and residues with similar properties are indicated by the symbol (.). Cdc45 contains the following domains: N-terminal DHH (Aspartate-Histidine-Histidine motif) domain, CMG/C-terminal Interacting Domain (CID), connector/RecJ-like domain, C-terminal DHH Associated Domain from subfamily 1 (DHHA1) domain.

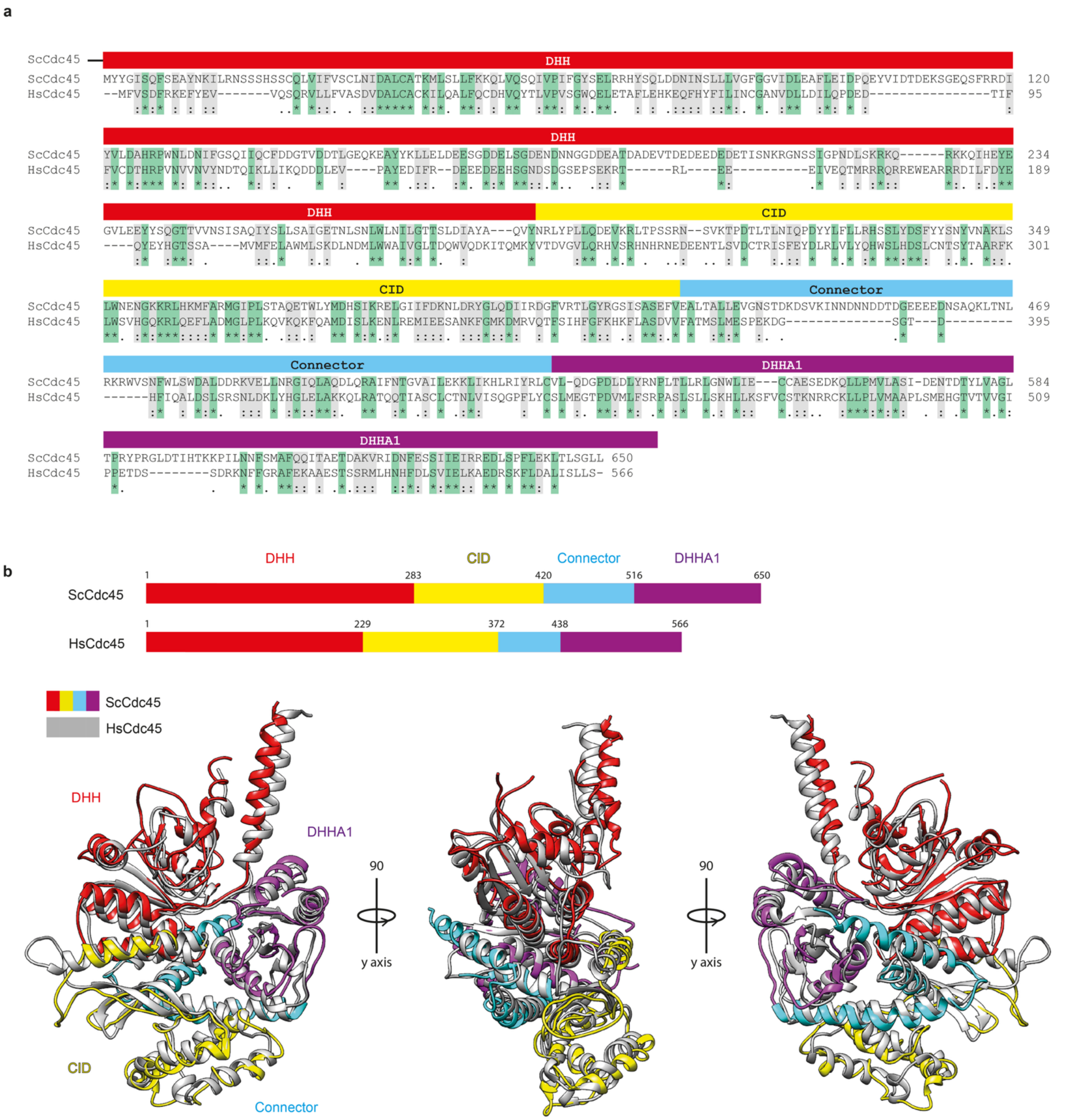

Supplementary Figure 23. Comparison of budding yeast and human Cdc45.
a. Sequence alignment of budding yeast (Sc, Saccharomyces cerevisiae) and human (Hs, Homo sapiens) Cdc45 performed using Clustal Omega. ScCdc45 domain boundaries are indicated above the respective sequence. Highly conserved residues are colored with a vertical green line and indicated by the symbol (*), highly similar residues are colored with a vertical grey line and indicated by the symbol (:), and residues with similar properties are indicated by the symbol (.). **b**. Superposition of the 3D structures of budding yeast (PDB: 6U0M) and human Cdc45 (PDB: 5DGO) reveals a highly similar architecture. Cdc45 contains the following domains: N-terminal DHH (Aspartate-Histidine-Histidine motif) domain, CMG/C-terminal Interacting Domain (CID), connector/RecJ-like domain, C-terminal DHH Associated Domain from subfamily 1 (DHHA1) domain.

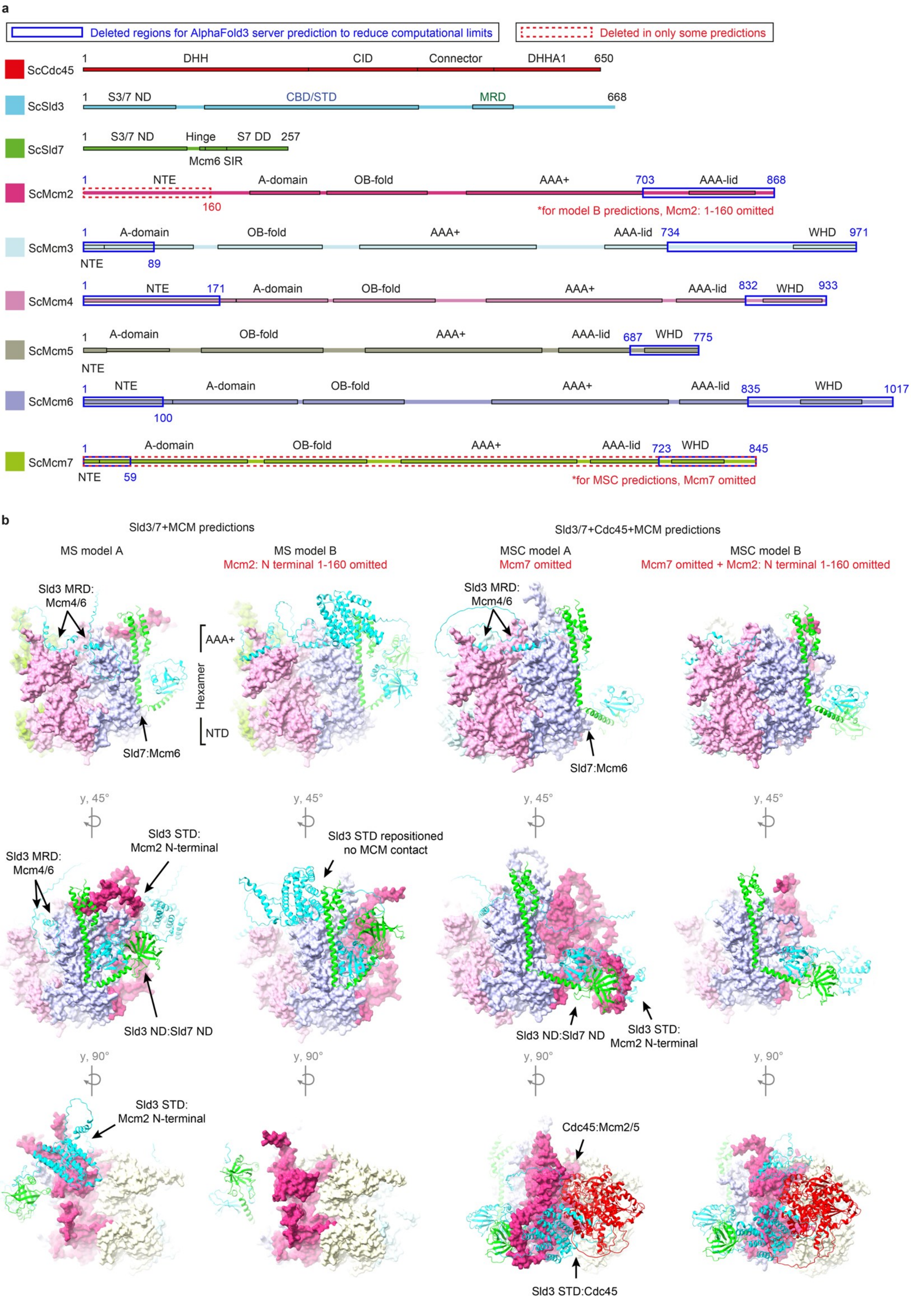
a
Deleted regions for AlphaFold3 server prediction to reduce computational limits
Deleted in only some predictions
ScCdc45
1 DHH CID Connector DHHA1 650
ScSld3
1 S3/7 ND CBD/STD MRD 668
ScSld7
1 S3/7 ND Hinge S7 DD 257
Mcm6 SIR
ScMcm2
1 NTE A-domain OB-fold AAA+ 703 AAA-lid 868
160
*for model B predictions, Mcm2: 1-160 omitted
ScMcm3
1 A-domain OB-fold AAA+ AAA-lid 734 WHD 971
NTE 89
ScMcm4
1 NTE 171 A-domain OB-fold AAA+ AAA-lid 832 WHD 933
ScMcm5
1 A-domain OB-fold AAA+ AAA-lid 687 WHD 775
NTE
ScMcm6
1 NTE A-domain OB-fold AAA+ AAA-lid 835 WHD 1017
100
ScMcm7
1 A-domain OB-fold AAA+ AAA-lid 723 WHD 845
NTE 59
*for MSC predictions, Mcm7 omitted
b
Sld3/7+MCM predictions
Sld3/7+Cdc45+MCM predictions
MS model A
MS model B
Mcm2: N terminal 1-160 omitted
MSC model A
Mcm7 omitted
MSC model B
Mcm7 omitted + Mcm2: N terminal 1-160 omitted
Sld3 MRD: Mcm4/6
AAA+
Hexamer
NTD
Sld7:Mcm6
y, 45°
Sld3 STD: Mcm2 N-terminal
Sld3 STD repositioned no MCM contact
Sld3 ND:Sld7 ND
y, 90°
Cdc45:Mcm2/5
Sld3 STD:Cdc45

Supplementary Figure 24. AlphaFold3 server predictions of MS and MSC complexes.
a. Domain organization of protein components used and the sequence regions included and omitted for protein structure prediction using AF3. All predictions shown used full-length (FL) Sld3, Sld7 or Cdc45, only MCM regions were omitted to overcome computational limitations. The dotted blue rectangles represent the variations in sequence used for models predicted in (**b**). **b**. Variations (model A/B) of AF3 models created for MS and MSC. Notably, the predictions show that in MS, the Sld3 STD density is localized at the N-terminal lobe/NTD of the hexamer, whereas in MSC it is repositioned towards the C-terminal AAA+ lobe to coordinate Cdc45 binding at the Mcm2/5 interface.

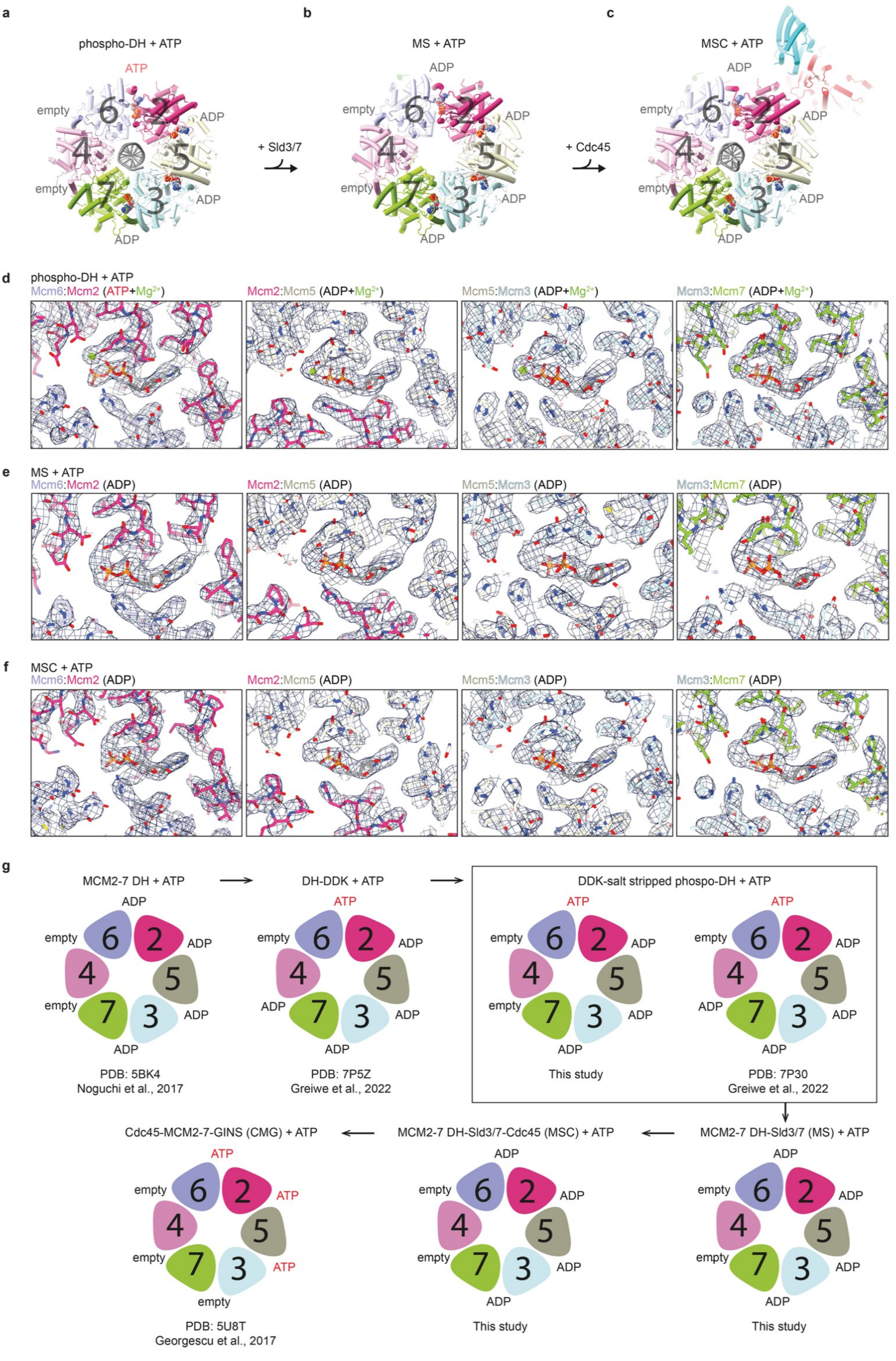

a
phospho-DH + ATP
ATP
empty
ADP
empty
ADP
ADP
+ Sld3/7
b
MS + ATP
ADP
empty
ADP
empty
ADP
ADP
+ Cdc45
c
MSC + ATP
ADP
empty
ADP
empty
ADP
ADP
d
phospho-DH + ATP
Mcm6:Mcm2 (ATP+Mg2+)
Mcm2:Mcm5 (ADP+Mg2+)
Mcm5:Mcm3 (ADP+Mg2+)
Mcm3:Mcm7 (ADP+Mg2+)
e
MS + ATP
Mcm6:Mcm2 (ADP)
Mcm2:Mcm5 (ADP)
Mcm5:Mcm3 (ADP)
Mcm3:Mcm7 (ADP)
f
MSC + ATP
Mcm6:Mcm2 (ADP)
Mcm2:Mcm5 (ADP)
Mcm5:Mcm3 (ADP)
Mcm3:Mcm7 (ADP)
g
MCM2-7 DH + ATP
PDB: 5BK4
Noguchi et al., 2017
DH-DDK + ATP
PDB: 7P5Z
Greiwe et al., 2022
DDK-salt stripped phospo-DH + ATP
This study
PDB: 7P30
Greiwe et al., 2022
Cdc45-MCM2-7-GINS (CMG) + ATP
PDB: 5U8T
Georgescu et al., 2017
MCM2-7 DH-Sld3/7-Cdc45 (MSC) + ATP
This study
MCM2-7 DH-Sld3/7 (MS) + ATP
This study

Supplementary Figure 25. Nucleotide occupancy of MCM2-7 during stepwise activation of the DNA bound helicase.

a-c. Cryo-EM derived atomic models showing the MCM2-7 ATPase ring nucleotide states in representative initiation intermediates. **a**. DNA-bound phosphorylated MCM2-7 double hexamer complex (phospho-DH + ATP; this study) displays mixed nucleotide occupancy. **b**. In the MCM2-7 DH-Sld3/7 complex (MS + ATP; this study), the overall nucleotide pattern resembles the phospho-DH + ATP state, but the Mcm6:Mcm2 site now contains ADP rather than ATP. This change suggests that engagement of Sld3/7 is accompanied by nucleotide turnover at this site, either through ATP hydrolysis or by promoting nucleotide exchange. **c**. In the MCM2-7 DH-Sld3/7-Cdc45 complex (MSC + ATP; this study) the same asymmetric nucleotide pattern as MS is retained. **d-f**. Close-up views of the six ATPase interfaces for the complexes in **a-c**, respectively. EM map density is shown for the nucleotide, associated Mg^{2+} ion and coordinating residues, illustrating that the map quality is sufficient to distinguish ATP+Mg^{2+} from ADP+Mg^{2+} and from nucleotide free sites. In contrast to phospho-DH, Mg^{2+} ions were omitted from MS and MSC because weaker density at the nucleotide-binding sites made Mg^{2+} assignment uncertain. Interfaces are labelled according to the two interacting MCM subunits. EM density maps shown in **d-f** were enhanced using CryoSAMU to improve interpretability and generate smoothed maps for visualization. **g**. Schematic comparison of nucleotide states across published and current structures (in the presence of ATP) of the initiation pathway. The DNA bound DH (MCM2-7 DH + ATP; PDB 5BK4; Noguchi et al., 2017) contains predominantly ADP and several empty sites and phosphorylation and DDK binding (phospho-DH-DDK + ATP; PDB 7P5Z; Greiwe et al., 2022) introduces a single ATP-bound site at Mcm6:Mcm2. The DDK-salt-stripped phospho-DH (phospho-DH + ATP; this study and equivalent reported by Greiwe et al., 2022; PDB 7P30) preserves this asymmetric pattern with ATP at Mcm6:Mcm2. Subsequent recruitment of Sld3/7 (MS) and Sld3/7-Cdc45 (MSC) features an ADP at Mcm6:Mcm2 with the remaining sites occupied by ADP or empty, whereas the later CMG structure (PDB 5U8T; Georgescu et al., 2017) shows ATP at multiple sites consistent with an activated helicase.

Supplementary Table 1. Summary of cryo-EM data collection and microscope parameters.

Sample	MS	DH/MSC (Data I)	MSC (Data II)
**Microscope**	Titan Krios	Titan Krios	Titan Krios
**Magnification**	81,000X	81,000X	81,000X
**Voltage (kV)**	300	300	300
**Camera**	Gatan K3	Falcon 3EC	Falcon 3EC
**Imaging mode**	Super-resolution	Linear	Linear
**Shots per hole**	3	3	3
**Total electron exposure (e^-/Å^2)**	50.4	58.4	62.1
**Total number of frames**	50	27	27
**Defocus range (μm)**	-1.0 to -3.2	-1.0 to -3.5	-1.5 to -3.0
**Automated data collection software**	EPU	EPU	EPU
**Pixel size (Å/pixel)**	0.530	1.085	1.085

Supplementary Table 2. Summary of cryo-EM data processing

Sample	phospho-DH (Data I)	MS composite	MSC composite (Data I)	MSC composite (Data II)
**Data collection and processing**				
**PDB ID**<br>**EMDB ID**	PDB:9TGD<br>EMD-55897	PDB:9TGM<br>EMD-55904	PDB:9TH8<br>EMD-55918	
**Number of multi-frame movies**	10,192	29,592	10,192	6,046
**Symmetry imposed**	C2	C1	C1	C1
**Initial auto-picked particles**	1,019,792	4,416,838	1,019,792	492,031
**Initial particle images**	172,947	1,774,309	109,233	186,548
**Final particle images**	64,506	344,362	292,336	
**Final map pixel size (Å)**	1.085	1.4434	1.085	1.085
**Map resolution (Å)**				
**FSC threshold (0.143) unmasked**	4.0	3.6<br>(mtriage estimation)	2.9<br>(mtriage estimation)	
**FSC threshold (0.143) masked**	3.3	N/A	N/A	
**Local resolution range (Å)**	2.5-4.0	2.5-8.0	2.5-8.0	
**Applied B-factor ($Å^2$)**	-176	N/A	N/A	N/A
**Final refinement software**	RELION	cryoSPARC	cryoSPARC	cryoSPARC

Supplementary Table 3. Summary of refinement and structure validation statistics.

Sample	phospho-DH (Data I)	MS composite	MSC composite (Data I/II)
Initial model used (PDB ID)	7PT6/7P30/5BK4	7PT6/7P30/5BK4/3WI3/ 3X38/AF3 MS model A	7PT6/7P30/5BK4/5U8S/A F3 MSC model A
**Model**			
Composition (#)			
Atoms	65010 (Hydrogens: 0)	63086 (Hydrogens: 0)	72267 (Hydrogens: 0)
Residues	Protein: 7900<br>Nucleotide: 120	Protein: 7958<br>Nucleotide: 0	Protein: 8793<br>Nucleotide: 120
Water	0	0	0
Ligands	ZN: 10<br>MG: 8<br>ATP: 2<br>ADP: 6	ZN: 10<br>ADP: 8	ZN: 10<br>ADP: 8
Bonds (RMSD)			
Length (Å) (# > 4σ)	0.004 (0)	0.004 (0)	0.003 (0)
Angles (°) (# > 4σ)	0.629 (0)	0.694 (1)	0.724 (3)
MolProbity score	1.68	1.67	1.82
Clash score	8.84	11.02	12.14
Ramachandran plot (%)			
Outliers	0.00	0.00	0.02
Allowed	3.27	2.56	3.41
Favored	96.73	97.44	96.56
Rama-Z (Ramachandran plot Z-score, RMSD)			
whole (N = 9150)	-0.82 (0.09)	0.17 (0.09)	-0.09 (0.09)
helix (N = 3584)	-0.22 (0.09)	0.79 (0.09)	0.53 (0.09)
sheet (N = 1592)	-1.13 (0.14)	-0.44 (0.13)	-0.59 (0.13)
loop (N = 3974)	-0.37 (0.10)	-0.12 (0.11)	-0.24 (0.11)
Rotamer outliers (%)	0.09	0.09	0.05
Cβ outliers (%)	NA	NA	NA
Peptide plane (%)			
Cis proline/general	0.0/0.0	0.0/0.0	0.0/0.0
Twisted proline/general	0.0/0.0	0.0/0.0	0.0/0.0
CaBLAM outliers (%)	1.61	0.78	1.51
ADP (B-factors)			
Iso/Aniso (#)	65010/0	63086/0	72267/0
Protein	22.72/184.31/81.34	-0.00/471.97/53.63	5.63/397.03/67.72
Nucleotide	10.34/184.27/75.53	NA	34.99/150.70/70.34
Ligand	50.00/183.25/80.67	24.30/243.78/50.81	41.37/151.91/53.26
Occupancy			
Mean	1.00	1.00	1.00
occ = 1 (%)	100.00	100.00	100.00
0 < occ < 1 (%)	0.00	0.00	0.00
occ > 1 (%)	0.00	0.00	0.00
**Model vs. Data**			
CC (mask)	0.86	0.79	0.80
CC (box)	0.79	0.75	0.78
CC (peaks)	0.75	0.69	0.70
CC (volume)	0.85	0.78	0.79
Mean CC for ligands	0.86	0.68	0.77

Supplementary Table 4. Summary of AlphaFold3 structural models deposited to ModelArchive and corresponding accession codes

Model / Data Name (Project Title)	Accession code
AlphaFold3 prediction of yeast MCM2-7-Sld3-Sld7-Cdc45 complex, referred to as MSC model B (excludes Mcm2 1-160)	ma-3dk7p
AlphaFold3 prediction of yeast MCM2-7-Sld3-Sld7-Cdc45 complex, referred to as MSC model A (includes Mcm2 1-160)	ma-1zsqn
AlphaFold3 prediction of yeast MCM2-7-Sld3-Sld7 complex, referred to as MS model B (excludes Mcm2 1-160)	ma-3pbi9
AlphaFold3 prediction of yeast MCM2-7-Sld3-Sld7 complex, referred to as MS model A (includes Mcm2 1-160)	ma-x5w4s
AlphaFold3 prediction of yeast Sld3 STD-Cdc45	ma-7fp39
AlphaFold3 prediction of yeast Sld3-Sld7-Cdc45 dimer / $(Sld3\text{-}Sld7\text{-}Cdc45)_2$ complex	ma-38qbs
AlphaFold3 prediction of yeast Sld3-Sld7-Cdc45	ma-r8lak
AlphaFold3 prediction of yeast Sld3-Sld7 heterotetramer / $(Sld3\text{-}Sld7)_2$ complex	ma-ykqf6
AlphaFold3 prediction of human TRESLIN STD-Cdc45-Mcm2/Mcm5	ma-t59e5
AlphaFold3 prediction of yeast Sld7	ma-ymqbw
AlphaFold3 prediction of yeast Sld3	ma-algfi

Supplementary Table 5. Oligonucleotides used in this study

Oligo	Name	Sequence
oCS1670	Mcm2_Fwd	*ACTGATCTAATACGACTCACTATAGGGCATCCACCATG*TCTGATAATAGAAGACGTAGAC
oCS1799	Mcm2_50_Fwd	*ACTGATCTAATACGACTCACTATAGGGCATCCACCATG*GACGATAATGAAGTTGACGATG
oCS1801	Mcm2_101_Fwd	*ACTGATCTAATACGACTCACTATAGGGCATCCACCATG*CAACAAGAACTATCTTTAAGCGAAC
oCS1805	Mcm2_200_Fwd	ACTGATCTAATACGACTCACTATAGGGCATCCACCATGCAACCTAATGTCTCAAGAACTATTG
oCS1673	Mcm2_322_Fwd	ACTGATCTAATACGACTCACTATAGGGCATCCACCATGGGGGTGGTGACAAGAAGAAC
oCS1784	Mcm2_666_Fwd	*ACTGATCTAATACGACTCACTATAGGGCATCCACCATG*AATGTTAGTTTGACCGAGCCTATT
oCS1800	Mcm2_200_Rev+1Met	AAAAAAAAAAAAAAAAAAAAAAAAAAAAAATTACATTTGTGTTATCCATTCCGAGTAAC
oCS1804	Mcm2_300_Rev+2Met	AAAAAAAAAAAAAAAAAAAAAAAAAAAAAATTACAT CATAAAATCAGAGATTCTTACGTGAATTTC
oCS1672	Mcm2_332_Rev	AAAAAAAAAAAAAAAAAAAAAAAAAAAAAATTACCCAGTGACGCGTACTAG
oCS1806	Mcm2_350_Rev+3Met	AAAAAAAAAAAAAAAAAAAAAAAAAAAAAATTACATCATCATTGGGCCCAAAATGGAGCCA
oCS1783	Mcm2_665_Rev	AAAAAAAAAAAAAAAAAAAAAAAAAAAAAATTACTGAGCTAAAGGCAAGGTTGAA
oCS1785	Mcm2_Rev_end	AAAAAAAAAAAAAAAAAAAAAAAAAAAAAATTAGTGACCCAAGGTATAAATTGC

Supplementary Table 6. Summary of deposited atomic coordinates and EM maps with corresponding PDB and EMDB accession codes.

EM map name		Final particle images	Map resolution (Å)	Applied B-factor ($Å^2$)	Accession ID (PDB/EMDB)
**Negative-stain EM envelope of crosslinked Sld3/7**		32,400	17	0	EMD-56011
**Negative-stain EM envelope of crosslinked Sld3/7-Cdc45**		11,009	18	0	EMD-56012
**DDK-salt stripped phosphorylated-DH (Data I)**		38,859	3.3	-176	PDB:9TGD EMD-55897
**MS composite**		344,362	3.6 (mtriage estimation, unmasked)	N/A	PDB:9TGM EMD-55904
MS consensus map and associated low-resolution MS map		882,092	3.2	0	EMD-55972
Associated focused maps:	C2 DH core	66,052	3.4	-60	EMD-55973
	Sld3 MRD1:Mcm6	63,192	3.4	-32	EMD-55974
	Sld3 MRD2:Mcm4	166,980	2.9	-19	EMD-55975
	Sld7:Mcm6	48,138	3.7	-10	EMD-55976
MS DH core C-lobe twist consensus map		511,094	3.1	0	EMD-55980
C-lobe twist states (I-III):	State I	131,570	3.2	-86	EMD-55981
	State II	138,018	3.2	-91	EMD-55982
	State III	106,939	3.3	-89	EMD-55983
**MSC composite (Data I+II)**		292,336	2.9 (mtriage estimation, unmasked)	N/A	PDB:9TH8 EMD-55918
MSC consensus map		106,950	3.4	-114	EMD-55985
Associated focused maps:	DH core	38,859	3.5	-80	EMD-55986
	Sld3 MRD1/2:Mcm4/6	149,668	3.2	-10	EMD-55987
	Sld7:Mcm6	51,334	4.1	-50	EMD-55988
	Sld3 STD/Cdc45:Mcm2/5	52,475	5.2	-210	EMD-55989
**3DFlex MSC (Data I+II)**		52,475	3.9	0	EMD-55990
**C1 2x Sld3/7-Cdc45 bound MSC (Data I+II)**		12,702	4.5	0	EMD-56009
**C2 2x Sld3/7-Cdc45 bound MSC (Data I+II)**		12,702	4.0	0	EMD-56010
