# Supplementary material for: Structural insights into Sld3-Sld7-dependent Cdc45 loading during replication initiation
Source: Nat Commun. 2026 Aug 14;17:9783. doi: 10.1038/s41467-026-76309-6 (PMC13572456; doi:10.1038/s41467-026-76309-6)
Supplement: Supplementary file 2 [file 41467_2026_76309_MOESM2_ESM.pdf]

Description of Additional Supplementary Files

File name: Supplementary Movie 1

Description: Cryo-EM structures of MCM2-7 DH-Sld3/7 (MS) and MCM2-7 DH-Sld3/7 (MSC).

File name: Supplementary Movie 2

Description: Multi-body analysis of MCM2-7 DH-Sld3/7 (MS).

File name: Supplementary Movie 3

Description: Morph between MCM2-7 DH-Sld3/7 (MS) and MCM2-7 DH-Sld3/7 (MSC) with AlphaFold3 predicted regions fitted.

File name: Supplementary Movie 4

Description: 3DFlex analysis of MCM2-7 DH-Sld3/7-Cdc45 (MSC).
