# Supplementary material for: Structural insights into Sld3-Sld7-dependent Cdc45 loading during replication initiation
Source: Nat Commun. 2026 Aug 14;17:9783. doi: 10.1038/s41467-026-76309-6 (PMC13572456; doi:10.1038/s41467-026-76309-6)
Supplement: Supplementary file 3 [file 41467_2026_76309_MOESM7_ESM.pdf]

nature portfolio

Corresponding author(s): Christian Speck, Almutasem Saleh

Last updated by author(s): 26-0162026

Reporting Summary

Nature Portfolio wishes to improve the reproducibility of the work that we publish. This form provides structure for consistency and transparency in reporting. For further information on Nature Portfolio policies, see our Editorial Policies and the Editorial Policy Checklist.

Statistics

For all statistical analyses, confirm that the following items are present in the figure legend, table legend, main text, or Methods section.

n/a	Confirmed	
☐	☒	The exact sample size (*n*) for each experimental group/condition, given as a discrete number and unit of measurement
☐	☒	A statement on whether measurements were taken from distinct samples or whether the same sample was measured repeatedly
☐	☒	The statistical test(s) used AND whether they are one- or two-sided<br>*Only common tests should be described solely by name; describe more complex techniques in the Methods section.*
☐	☒	A description of all covariates tested
☐	☒	A description of any assumptions or corrections, such as tests of normality and adjustment for multiple comparisons
☒	☐	A full description of the statistical parameters including central tendency (e.g. means) or other basic estimates (e.g. regression coefficient) AND variation (e.g. standard deviation) or associated estimates of uncertainty (e.g. confidence intervals)
☒	☐	For null hypothesis testing, the test statistic (e.g. *F*, *t*, *r*) with confidence intervals, effect sizes, degrees of freedom and *P* value noted<br>*Give P values as exact values whenever suitable.*
☒	☐	For Bayesian analysis, information on the choice of priors and Markov chain Monte Carlo settings
☒	☐	For hierarchical and complex designs, identification of the appropriate level for tests and full reporting of outcomes
☒	☐	Estimates of effect sizes (e.g. Cohen's *d*, Pearson's *r*), indicating how they were calculated

Our web collection on statistics for biologists contains articles on many of the points above.

Software and code

Policy information about availability of computer code

Data collection	ThermoFischer EPU (Talos/Krios). AcquireMP software (v. 2023R2, Refeyn Ltd).
Data analysis	cryoSPARC v4.6.2, RELION v3.1/v4.0, COOT v0.9.8, Phenix v1.21.2, UCSF Chimera v1.19, ChimeraX-1.9, ProteoWizard 3.0.11729, xiSEARCH 1.7.6.4, xiFDR 2.1.5.6, DiscoverMP software (v2023R2, Refeyn Ltd), MotionCor2 v1.2.3, CTFFIND4 v4.1.14

For manuscripts utilizing custom algorithms or software that are central to the research but not yet described in published literature, software must be made available to editors and reviewers. We strongly encourage code deposition in a community repository (e.g. GitHub). See the Nature Portfolio guidelines for submitting code & software for further information.

Data

Policy information about availability of data

All manuscripts must include a data availability statement. This statement should provide the following information, where applicable:

- Accession codes, unique identifiers, or web links for publicly available datasets
- A description of any restrictions on data availability
- For clinical datasets or third party data, please ensure that the statement adheres to our policy

The cryo-EM data in this study have been deposited (summarized in Supplementary Table 6) in the Electron Microscopy Data Bank (EMDB) under accession code EMD-55897 [https://www.ebi.ac.uk/emdb/EMD-55897] (DDK-salt stripped phosphorylated DH), EMD-55904 [https://www.ebi.ac.uk/emdb/EMD-55904] MCM2-7 DH-Sld3/7 (MS), EMD-55918 [https://www.ebi.ac.uk/emdb/EMD-55918] MCM2-7 DH-Sld3/7-Cdc45 (MSC), EMD-56011 [https://www.ebi.ac.uk/emdb/EMD-56011]

negative-stain EM envelope of crosslinked Sld3/7, EMD-56012 [https://www.ebi.ac.uk/emdb/EMD-56012] negative-stain EM envelope of crosslinked Sld3/7-Cdc45, EMD-55972 [https://www.ebi.ac.uk/emdb/EMD-55972] MS consensus map and associated low resolution MS map with associated maps: (EMD-55973 [https://www.ebi.ac.uk/emdb/EMD-55973] C2 DH core, EMD-55974 [https://www.ebi.ac.uk/emdb/EMD-55974] Sld3 MRD1:Mcm6, EMD-55975 [https://www.ebi.ac.uk/emdb/EMD-55975] Sld3 MRD2:Mcm4, EMD-55976 [https://www.ebi.ac.uk/emdb/EMD-55976] Sld7:Mcm6), EMD-55980 [https://www.ebi.ac.uk/emdb/EMD-55980] MS DH core C-lobe twist consensus map with associated maps: (EMD-55981 [https://www.ebi.ac.uk/emdb/EMD-55981] C-lobe twist State I, EMD-55982 [https://www.ebi.ac.uk/emdb/EMD-55982] C-lobe twist State II, EMD-55983 [https://www.ebi.ac.uk/emdb/EMD-55983] C-lobe twist State III), EMD-55985 [https://www.ebi.ac.uk/emdb/EMD-55985] MSC consensus map with associated focused maps: (EMD-55986 [https://www.ebi.ac.uk/emdb/EMD-55986] DH core, EMD-55987 [https://www.ebi.ac.uk/emdb/EMD-55987] Sld3 MRD1/2:Mcm4/6, EMD-55988 [https://www.ebi.ac.uk/emdb/EMD-55988] Sld7:Mcm6, EMD-55989 [https://www.ebi.ac.uk/emdb/EMD-55989] Sld3 STD/Cdc45:Mcm2/5), MD-55990 [https://www.ebi.ac.uk/emdb/EMD-55990] 3DFlex MSC, EMD-56009 [https://www.ebi.ac.uk/emdb/EMD-56009] C1 2x Sld3/7-Cdc45 bound MSC, EMD-56010 [https://www.ebi.ac.uk/emdb/EMD-56010] C2 2x Sld3/7-Cdc45 bound MSC. The atomic coordinates have been deposited in the RCSB Protein Data Bank under accession code PDB:9TGD [https://www.rcsb.org/structure/unreleased/9TGD] (DDK-salt stripped phosphorylated DH), PDB:9TGM [https://www.rcsb.org/structure/unreleased/9TGM] MCM2-7 DH-Sld3/7 (MS) and PDB:9TH8 [https://www.rcsb.org/structure/unreleased/9TH8] MCM2-7 DH-Sld3/7-Cdc45 (MSC). The crosslinking mass spectrometry data have been deposited in the ProteomeXchange Consortium via the PRIDE partner repository under the accession code PXD070193 [https://www.ebi.ac.uk/pride/archive/projects/PXD070193]. Previously published structural data used for comparison, rigid-body fitting and model building are available from the EMDB and RCSB Protein Data Bank under accession codes EMD-31684 [https://www.ebi.ac.uk/emdb/EMD-31684] (unphosphorylated MCM2-7 DH), PDB:3WI3 [https://www.rcsb.org/structure/3WI3] (Sld3 STD), PDB:3X37 [https://www.rcsb.org/structure/3X37] (Sld3/7 ND homolog), PDB:3X38 [https://www.rcsb.org/structure/3X38] (Sld7 DD), PDB:5U8S [https://www.rcsb.org/structure/5U8S] (Cdc45), PDB:6U0M [https://www.rcsb.org/structure/6U0M] (budding yeast Cdc45), PDB:5DGO [https://www.rcsb.org/structure/5DGO] (human Cdc45), PDB:5BK4 [https://www.rcsb.org/structure/5BK4] (DNA-bound MCM2-7 DH), PDB:7P5Z [https://www.rcsb.org/structure/7P5Z] (phospho-DH-DDK), PDB:7P30 [https://www.rcsb.org/structure/7P30] (DDK-salt-stripped phospho-DH), PDB:7PT6 [https://www.rcsb.org/structure/7PT6] (DH-DDK complex), PDB:5U8T [https://www.rcsb.org/structure/5U8T] (CMG), PDB:6SKL [https://www.rcsb.org/structure/6SKL] (CMG-Ctf4-fork protection complex), and PDB:8KG6 [https://www.rcsb.org/structure/8KG6] (replisome). The AlphaFold3 models generated in this study have been deposited (summarized in Supplementary Table 4) in ModelArchive under accession codes ma-3dk7p [https://modelarchive.org/doi/10.5452/ma-3dk7p] (yeast MCM2–7–Sld3–Sld7–Cdc45 complex, MSC model B, excluding Mcm2 residues 1–160), ma-1zsqn [https://modelarchive.org/doi/10.5452/ma-1zsqn] (yeast MCM2–7–Sld3–Sld7–Cdc45 complex, MSC model A, including Mcm2 residues 1–160), ma-3pbi9 [https://modelarchive.org/doi/10.5452/ma-3pbi9] (yeast MCM2–7–Sld3–Sld7 complex, MS model B, excluding Mcm2 residues 1–160), ma-x5w4s [https://modelarchive.org/doi/10.5452/ma-x5w4s] (yeast MCM2–7–Sld3–Sld7 complex, MS model A, including Mcm2 residues 1–160), ma-7fp39 [https://modelarchive.org/doi/10.5452/ma-7fp39] (yeast Sld3 STD–Cdc45 complex), ma-38qbs [https://modelarchive.org/doi/10.5452/ma-38qbs] (yeast Sld3–Sld7–Cdc45 dimer, or $(Sld3–Sld7–Cdc45)_2$ complex), ma-r8lak [https://modelarchive.org/doi/10.5452/ma-r8lak] (yeast Sld3–Sld7–Cdc45 complex), ma-ykqf6 [https://modelarchive.org/doi/10.5452/ma-ykqf6] (yeast Sld3–Sld7 heterotetramer, or $(Sld3–Sld7)_2$ complex), ma-t59e5 [https://modelarchive.org/doi/10.5452/ma-t59e5] (human TRESLIN STD–Cdc45–Mcm2/Mcm5 complex), ma-ymqbw [https://modelarchive.org/doi/10.5452/ma-ymqbw] (yeast Sld7), and ma-algfi [https://modelarchive.org/doi/10.5452/ma-algfi] (yeast Sld3). The models' requests for resources and reagents should be directed to the corresponding authors and will be fulfilled by them. Source data are provided with this paper.

Research involving human participants, their data, or biological material

Policy information about studies with human participants or human data. See also policy information about sex, gender (identity/presentation), and sexual orientation and race, ethnicity and racism.

Reporting on sex and gender	N/A
Reporting on race, ethnicity, or other socially relevant groupings	N/A
Population characteristics	N/A
Recruitment	N/A
Ethics oversight	N/A

Note that full information on the approval of the study protocol must also be provided in the manuscript.

Field-specific reporting

Please select the one below that is the best fit for your research. If you are not sure, read the appropriate sections before making your selection.

☒ Life sciences ☐ Behavioural & social sciences ☐ Ecological, evolutionary & environmental sciences

For a reference copy of the document with all sections, see nature.com/documents/nr-reporting-summary-flat.pdf

Life sciences study design

All studies must disclose on these points even when the disclosure is negative.

Sample size	No statistical methods were used to predetermine sample size, as much data as possible was collected during allocated instrument session time.
Data exclusions	Data was excluded at different stages of image processing. Particles that did not meet the image resolution quality criteria or could not be averaged were excluded from the final structures, based on 2D averages and 3D map classification results. The method of data exclusion is common practice in the cryo-EM field to allow for the generation of interpretable 3D models.

Replication	Biochemical experiments were repeated two or three times as biological replicates, and replication was successful. Very large experiments involving cross-linking mass spectrometry or electron microscopy structural analysis were repeated only once and labelled accordingly, consistently with standard procedure in the field.
Randomization	Experiments were not randomized due to the nature of the data not involving patients or animals. The experiments were not grouped and needed to be preformed in a particular order.
Blinding	Blinding is not applicable to this research, which does not entail randomization and does not involve patients or animals. The experiments required essential visual inspection of the data quality during data screening and data collection and so no blinding could be performed.

Reporting for specific materials, systems and methods

We require information from authors about some types of materials, experimental systems and methods used in many studies. Here, indicate whether each material, system or method listed is relevant to your study. If you are not sure if a list item applies to your research, read the appropriate section before selecting a response.

Materials & experimental systems

n/a	Involved in the study	
☐	☒	Antibodies
☒	☐	Eukaryotic cell lines
☒	☐	Palaeontology and archaeology
☒	☐	Animals and other organisms
☒	☐	Clinical data
☒	☐	Dual use research of concern
☒	☐	Plants

Methods

n/a	Involved in the study	
☒	☐	ChIP-seq
☒	☐	Flow cytometry
☒	☐	MRI-based neuroimaging

Antibodies

Antibodies used	Antibody: anti-MBP (mouse), Catalogue number: NEB E8032L
Validation	Validated by NEB

Plants

Seed stocks	*Report on the source of all seed stocks or other plant material used. If applicable, state the seed stock centre and catalogue number. If plant specimens were collected from the field, describe the collection location, date and sampling procedures.*
Novel plant genotypes	*Describe the methods by which all novel plant genotypes were produced. This includes those generated by transgenic approaches, gene editing, chemical/radiation-based mutagenesis and hybridization. For transgenic lines, describe the transformation method, the number of independent lines analyzed and the generation upon which experiments were performed. For gene-edited lines, describe the editor used, the endogenous sequence targeted for editing, the targeting guide RNA sequence (if applicable) and how the editor was applied.*
Authentication	*Describe any authentication procedures for each seed stock used or novel genotype generated. Describe any experiments used to assess the effect of a mutation and, where applicable, how potential secondary effects (e.g. second site T-DNA insertions, mosiacism, off-target gene editing) were examined.*
