# Supplementary material for: Structural insights into Sld3-Sld7-dependent Cdc45 loading during replication initiation
Source: Nat Commun. 2026 Aug 14;17:9783. doi: 10.1038/s41467-026-76309-6 (PMC13572456; doi:10.1038/s41467-026-76309-6)
Supplement: Supplementary file 4 [file 41467_2026_76309_MOESM8_ESM.pdf]

nature portfolio

Peer Review File

Structural insights into Sld3-Sld7-dependent Cdc45 loading during replication initiation

Corresponding Author: Professor Christian Speck

This file contains all reviewer reports in order by version, followed by all author rebuttals in order by version.

Version 0:

Reviewer comments:

Reviewer #1

(Remarks to the Author)
This paper describes a single particle cryo-EM based investigation of the structural basis of the regulation MCM2-7 replicative helicase by DDK phosphorylation, the Cdc45 protein and the and the Sld3-Sld7 protein complex. To this end the structures of various complexes formed from the MCM2-7 double hexamer are compared to form the basis of a mechanistic structural model of part of the replication initiation pathway. This is an important and relevant research area and the structural analysis of these MCM2-7 complexes is a promising approach. However, the it is important in cryo-EM based structural analysis of this nature to demonstrate that the conclusions are fully justified. In my view this is not fully achieved in the current version of this paper and I recommend that this is addressed in a revised version.

Detailed points:-

1) DDK releases an autoinhibitory loop that binds to Mcm4 and Mcm6.
In this section the authors compare the structures they obtained of DDK phosphorylated MCM2-7 double hexamer before with existing data from unphosphorylated MCM2-7 double hexamer obtained by Cheng et al, 2022 (confusingly referred to in the text as Zhai et al).
They authors state that they 'reanalysed' the unphosphorylated map. They should explain what this means. For example, did they calculate an improved map from raw data, and/or postprocess the map or do they simply mean that they extended the existing interpretation of the deposited map by building atomic coordinates into extra regions?
At the bottom of page 4 the authors state :- "Our re-analysis of the unphosphorylated MCM2-7 DH map shows an additional density adjacent to Mcm6, which is absent in our DDK-phosphorylated MCM2-7 map (Fig. 1C), and is in agreement with all other available DDK-phosphorylated MCM2-7 maps27-29. Importantly, Dbf4 contacts the same regions of Mcm4 and Mcm6 (Fig. 1D)27-29."
My understanding here is that the additional density identified in the authors' reanalysis is the region adjacent to MCM6 (coloured red in Fig 1C), while the densities adjacent to MCM4 (also coloured red) had previously been reported in the earlier work and already assigned to the N-terminal serine/threonine rich domain of MCM. The final sentence states that DFB4 contacts the same regions of MCM4 and MCM6 (presumably the regions coloured red in Fig 1c), but appears to refer back to a single region which just contacts MCM6. This needs to be clarified. Additionally it is suggested in the the legend for Fig1 that the newly identified density corresponds to the "upstream region of the MCM4 N-terminal tail". This comment should be brought into the main body of the text and properly justified. It would also help if the authors clarified what they mean by the upstream region of the MCM4 N-terminal tail. Do they actually mean the N-terminus itself? It appears that the first 130 residues of MCM4 remain unaccounted for.
The colour coding information in Fig 1 should include blue for CDC7.

2) Sld3-Sld7 recognize the DDK-phosphorylated MCM2-7 DH
The authors present their cryo-EM structure of the complex formed between the DDK-phosphorylated MCM2-7 double hexamer and Sld3-Sld7 at an estimated resolution of between 2.5 and 8 Angstroms.
In paragraph 4 page 6, they state that DNA can be lost in the glycerol gradient they use to purify the complex. It is not clear what the relevance of this observation is.
In paragraphs 3 & 4, page 7, they assign regions of their cryo-EM map to Sld3 and Sld7. They need to explain the reasons for their assignments and to present supporting evidence such as agreement between fitted coordinates and experimental density. In particular, the assigned regions of Sld3 appear to consist of two short fragments (11 and 18 amino acids). In my

view it would be quite challenging to make unique structural assignments of such fragments based solely on agreement with cryo-EM density at the resolution. These assignments need to be backed up by detailed comparison between the fitted coordinates and density and any other supporting evidence available. Furthermore in paragraph 4, page7, they state that "Sld3 makes multiple contacts with MCM2-7 DH (Fig 3E)" We resolved two of these at high resolution". As far as I can see Fig 3E only shows the "high resolution" contact regions.

3) MCM2-7 ATP status and structural change
The authors use 3D classification to identify conformers of the MCM2-7 Sld3-Sld7 complex differentiated by a twisting of the C-lobes. The authors relate this twisting to proposed differences in nucleotide binding between the phosphorylated MCM2-7 double hexamer and the MCM2-7 Sld3-Sld7 complex. Firstly, they need to explain why they are making this comparison. Is there a conformational change between the C-lobes of these two complexes equivalent to the twisting they describe in the classes of the MCM2-7 Sld3-Sld7 complex? Secondly, when they introduce the concept of differential nucleotide binding they need to present the evidence that this occurs. Strangely, in this section they just refer to Figure 5h which is a schematic illustration. Later in the section titled "Structural changes upon MSC to CMG transition" on page 13, nucleotide binding is revisited and Extended data Figure 17 is referred to. Here, they show maps and fitted coordinates for the nucleotide binding regions of phosphorylated MCM2-7 and the MCM2-7 Sld3-Sld7 complex. Although the experimental data, at the resolution obtained it is not obvious to me that it is possible to reliably distinguish between ATP and ADP. At the chosen contour level, the terminal phosphate appears to be tightly constrained within the density contour in the structures identified as ATP, while in those designated as ADP there appears to be quite a lot of free space. Moreover, the placement of the coordinated Mg ions appear somewhat arbitrary and it is not obvious that the geometry of coordination is correct.

References
Something has gone wrong with references 54 & 56 which are marked as INVALID CITATION.

Reviewer #2

(Remarks to the Author)
In this manuscript, using the yeast system, the authors present an extensive cryo-EM and biochemical analysis of early eukaryotic replication initiation intermediates, focusing on how the DDK-phosphorylated MCM2–7 double hexamer recruits Sld3, Sld7, and Cdc45. Using a combination of high-resolution cryo-EM of the MCM2–7 DH, focused refinement strategies, biochemical reconstitution, mass photometry, and integrative modeling, the study defines structural snapshots of the MCM2–7–Sld3–Sld7 (MS) and MCM2–7–Sld3–Sld7–Cdc45 (MSC) complexes.

The work provides a coherent structural framework that consolidates prior genetic and biochemical observations: (i) DDK phosphorylation relieves Mcm4-mediated autoinhibition, (ii) Sld3/Sld7 recognize the activated MCM surface, and (iii) Sld3 recruits Cdc45 to initiate CMG assembly. A particularly strong aspect of the study is the identification and functional validation of a specific Sld3–Cdc45 interface within the Sld3 STD, supported by a designed mutant that selectively disrupts Cdc45 recruitment while preserving MCM binding. This represents a meaningful advance in understanding how Cdc45 is delivered to the helicase.

Overall, the manuscript offers a comprehensive and technically sophisticated structural synthesis of early helicase activation, and it will be of interest to the replication field. At the same time, several conclusions rely on model-supported interpretation and would benefit from more cautious framing.

In addition, the manuscript would benefit from clearer and more focused presentation. The current organization, with eight multi-panel figures, makes it difficult to identify the central advances. Streamlining the text and reducing the number of figures to emphasize the key novelties would substantially improve readability and impact.

Major points

1) Resolution and certainty of Sld3/Sld7 interface assignments
While the global MCM maps reach high resolution, the Sld3 and Sld7 densities are peripheral, lower-occupancy, and locally lower resolution. The placement of Sld3 MRD1/MRD2 and Sld7 MBH/DD motifs relies heavily on focused refinement, prior biochemical data, and AlphaFold modeling. The manuscript at times implies residue-level or "high-resolution" definition of these interfaces, which is not fully supported by the density. The authors should include map-to-model correlation plots (or provide map-to-model FSCs) for the critical newly modelled regions of the proteins.

2) Asymmetry of Sld3/Sld7 binding to the MCM2–7 double hexamer
The reconstructions show Sld3/Sld7 density on only one hexamer of the MCM double hexamer. While this is consistent with the asymmetric nature of early initiation intermediates, the manuscript does not clearly distinguish whether this asymmetry reflects biology, limited occupancy, or focused classification bias.

3) Interpretation of Sld7 dimer "splitting" upon DH binding
The observation that the Sld7 C-terminal dimerization domains are separated when bound to the DH is well supported geometrically. However, the manuscript frames this rearrangement as a functional mechanism that enhances recruitment efficiency. Given the bivalent nature of the DH, dissociation of the Sld7 dimer upon engagement of two Mcm6 sites appears to be an expected geometric consequence rather than a demonstrated regulatory feature.

4) Physiological relevance of oligomeric Sld3/Sld7/Cdc45 assemblies in solution

The mass photometry, crosslinking, and negative-stain EM data indicate that higher-order Sld3–Sld7 and Sld3–Sld7–Cdc45 assemblies can form in vitro. However, these experiments rely on crosslinking and low-resolution methods and lack mutational or functional validation of the inferred oligomerization interfaces.
Please temper conclusions regarding the biological relevance of these oligomeric assemblies and emphasize that they are observed under in vitro, crosslinked conditions.

5) Validation of the Sld3–Cdc45 interface
The manuscript identifies a specific Sld3–Cdc45 interaction interface within the Sld3 STD and supports its functional relevance through mutational analysis. While this is one of the strongest aspects of the study, the structural interpretation of the interface would benefit from more explicit validation against the cryo-EM density. In particular, no map–model Fourier shell correlation (FSC) curves or local map–model correlation coefficients (CCs) are provided for the Sld3–Cdc45 interface region.

6) Explicit acknowledgment of model dependence
AlphaFold models are used extensively to guide interpretation of densities that are not fully resolved. While this is reasonable, the dependence on modeling is not always made explicit. Please clearly state where AlphaFold predictions are used to support structural interpretation, both in the Results and in the relevant figure legends.

Minor points
1. The manuscript states that “the Cdc45 structure has been identified only in the context of the CMG complex.” This is incorrect, as a structure of human Cdc45 in isolation was reported previously (Simon et al., 2016, Nature Communications). Please revise accordingly.
2. The description of the 3D-FLEX analysis overstates the level of modeling performed. While conformational variability is visualized, atomic models are not fitted to the 3D-FLEX maps. Please rephrase to reflect this more accurately.
3. The reference list contains several invalid or incorrect citations. Please revise the references and in-text citations for accuracy and completeness.

Reviewer #3

(Remarks to the Author)
There has been an explosion of molecular insights into the eukaryotic DNA replication machinery over the last decade. This is a very active area of research and of broad interest to multiple fields interested in molecular mechanisms relating to genome biology. Cryo-EM of replisome complexes reconstituted from purified proteins has been crucial in this area. Here, Speck and colleagues use this approach to study the conversion of MCM double hexamers into pairs of replisomes during DNA replication initiation. These initiation steps have been extensively studied, but there are still very few published structures of intermediates in the CMG assembly pathway, which represents an important gap in our understanding.

The authors solve structures of multiple complexes containing different combinations of the ‘firing factors’ Sld3-7 and Cdc45 bound to Mcm2-7 double hexamers. The structures provide interesting and novel insights into how Sld3, Sld7 and Cdc45 bind to Mcm2-7 double hexamers, and how these interactions are regulation by the Dbf4-dependent kinase. The structures presented in this manuscript represent a step forward in our molecular understanding of replication initiation. I detail below the concerns that I think should be addressed before publication.

Major points
• The manuscript as a whole is unnecessarily long, with too many figures and very dense text. This has the overall effect of burying the main messages (which are themselves novel and interesting) in a sea of data. I recommend significantly reorganising the manuscript. For example, some of the data in Figure 2 and 4 could be omitted or at least shifted to the supplementary material. There is also some redundancy in other figures (e.g. 8g vs 8h, 7a-c, 6b-c, 1f vs. 4h) which could be addressed. This would make the manuscript much more accessible to the reader.

• The Sld3-Mcm2-7 interactions delineated by the authors’ structures are of great interest, and provide a key molecular insight into how DDK phosphorylation of Mcm2-7 facilitates Sld3 binding. Although based on lower confidence portions of the EM density maps (see PDB report), these surfaces on Mcm4/6 and Sld3 have been previously subjected to mutational / functional analysis by the Stillman, Bell and Diffley labs, in support of the authors’ conclusions. The same cannot be said, however, for the completely novel Sld7-Mcm interactions described in the paper. Although Sld7 is not essential, its deletion does confer defective S-phase progression and a slow growth phenotype in budding yeast (PMID: 21487389). The authors are now in a position to assess the relative contributions of e.g. Mcm binding vs. Sld7 dimerisation to Sld7 function in vivo. This is relatively straightforward yeast genetics and would significantly improve the manuscript.

Minor points
• How confident are the authors of their assignment of the ‘extra’ density on the surface on Mcm6 to the Mcm4 N-terminal tail? I appreciate that this density is absent in the DDK phosphorylated structure, but this could be a product of some other phosphorylation-induced conformational change. It would be quite straight forward to solve a structure of a double hexamer using mutant Mcm2-7 in which this tail was deleted.

• It is not obvious why the mass photometry in Fig. 2 was performed with crosslinked complexes. Has this been done with native Sld3-7 as well? This should at least be commented upon.

• According to PMID: 26912723, the Sld3-Cdc45 interaction in Fig. 7 has been functionally assessed (like the Sld3-Mcm interaction that the authors discuss extensively) – this could be commented upon.

• The XL-MS data presented in Fig. 5 is very hard to follow. This is partly because of how busy the structural representations of the cross links are, but also because the data is buried in a figure containing 12 panels covering everything from Sld7 dimerisation to nucleotide binding by Mcm2-7 and mapping DDK-independent interactions in the MS complex. This is a general theme throughout the paper (see major point above) and is not helpful for the reader.

Version 1:

Reviewer comments:

Reviewer #1

(Remarks to the Author)
The paper has been substantially revised taking into account the reviewers' comments and is greatly improved. Overall, I consider the paper ready for publication.

On rereading, however, I did run in difficulties understanding the schematic diagram in figure 1c left panel, illustrating the autoinhibitory binding of MCM4 N-terminal regions to activation factor binding sites on MCM4 and MCM6. Here in the lower hexamer, MCM4 (pink) is shown on the right of MCM6 (lilac). This seems to contradict the arrangement in the density maps in figure 1 and elsewhere which show MCM4 to the left of MCM6 in the lower hexamer. Is this intentional? The arrangement is repeated in related schematic diagrams in figure 1, figure 3 and figure 8.

Reviewer #2

(Remarks to the Author)
The authors have responded thoroughly to my comments, and the manuscript is substantially improved. The text is clearer and better focused, the integrative basis for the peripheral Sld3/Sld7 assignments is now stated transparently, and the additional map-to-model validation is provided. The Sld3–Cdc45 interface (the central advance) is now convincingly supported both structurally and functionally.
I have one remaining request, which concerns wording rather than data, and I recommend publication once it is addressed. The local validation for the MSC complex (Supplementary Fig. 18) is considerably weaker than for the MS complex: for MRD1, the chain and side-chain correlations (≈0.21 and ≈0.18) are essentially at the noise level, and MRD2 is only marginally better. This is at odds both with the statement that the MSC MRD regions were "resolved at a similar local resolution to those in the MS map" and with the description elsewhere of the Sld3 MRD as "resolved to high resolution." I ask that the text describe these MRD motifs consistently as backbone/placement-level assignments, without implying high-resolution or sequence-register certainty, particularly for the MSC complex.
All other points from my original review have been satisfactorily addressed.

Reviewer #3

(Remarks to the Author)
I am satisfied that all comments have been addressed and support publication of this manuscript.

Open Access This Peer Review File is licensed under a Creative Commons Attribution 4.0 International License, which permits use, sharing, adaptation, distribution and reproduction in any medium or format, as long as you give appropriate credit to the original author(s) and the source, provide a link to the Creative Commons license, and indicate if changes were made.
In cases where reviewers are anonymous, credit should be given to 'Anonymous Referee' and the source.
The images or other third party material in this Peer Review File are included in the article's Creative Commons license, unless indicated otherwise in a credit line to the material. If material is not included in the article's Creative Commons license and your intended use is not permitted by statutory regulation or exceeds the permitted use, you will need to obtain permission directly from the copyright holder.
To view a copy of this license, visit https://creativecommons.org/licenses/by/4.0/

We sincerely thank the reviewers for their time and for their thoughtful, constructive, and encouraging feedback. Their suggestions have been very helpful in improving the clarity, rigor, and overall presentation of the manuscript. We have carefully addressed each comment in the revised manuscript and provide a detailed point-by-point response below.

Reviewer #1 (Remarks to the Author):

This paper describes a single particle cryo-EM based investigation of the structural basis of the regulation MCM2-7 replicative helicase by DDK phosphorylation, the Cdc45 protein and the and the Sld3-Sld7 protein complex. To this end the structures of various complexes formed from the MCM2-7 double hexamer are compared to form the basis of a mechanistic structural model of part of the replication initiation pathway. This is an important and relevant research area and the structural analysis of these MCM2-7 complexes is a promising approach. However, the it is important in cryo-EM based structural analysis of this nature to demonstrate that the conclusions are fully justified. In my view this is not fully achieved in the current version of this paper and I recommend that this is addressed in a revised version.

We thank the reviewer for their constructive comments and detailed points. We have revised the manuscript and incorporated their suggestions, which have strengthened our manuscript.

Detailed points:1) DDK releases an autoinhibitory loop that binds to Mcm4 and Mcm6.

In this section the authors compare the structures they obtained of DDK phosphorylated MCM2-7 double hexamer before with existing data from unphosphorylated MCM2-7 double hexamer obtained by Cheng et al, 2022 (confusingly referred to in the text as Zhai et al).

Thank you for noticing this oversight. We have corrected the reference to Cheng *et al*., 2022.

They authors state that they 'reanalysed' the unphosphorylated map. They should explain what this means. For example, did they calculate an improved map from raw data, and/or postprocess the map or do they simply mean that they extended the existing interpretation of the deposited map by building atomic coordinates into extra regions?

We have replaced the term "reanalysis" with "reinterpretation" throughout to clarify that we reinterpreted, using new binding interface information from our MS and MSC structures, the previously deposited cryo-EM map and associated atomic model of the unphosphorylated MCM2-7 double hexamer reported by Cheng et al. (2022, EMD-31684/PDB: 7V3U). Indeed,

we did not reprocess the raw cryo-EM data or remodel the deposited atomic model, and accordingly, we have clarified the manuscript.

At the bottom of page 4 the authors state :- “Our re-analysis of the unphosphorylated MCM2-7 DH map shows an additional density adjacent to Mcm6, which is absent in our DDK-phosphorylated MCM2-7 map (Fig. 1C), and is in agreement with all other available DDK-phosphorylated MCM2-7 maps27-29. Importantly, Dbf4 contacts the same regions of Mcm4 and Mcm6 (Fig. 1D)27-29.”
My understanding here is that the additional density identified in the authors’ reanalysis is the region adjacent to MCM6 (coloured red in Fig 1C), while the densities adjacent to MCM4 (also coloured red) had previously been reported in the earlier work and already assigned to the N-terminal serine/threonine rich domain of MCM. The final sentence states that DFB4 contacts the same regions of MCM4 and MCM6 (presumably the regions coloured red in Fig 1c), but appears to refer back to a single region which just contacts MCM6. This needs to be clarified.

Additionally it is suggested in the legend for Fig1 that the newly identified density corresponds to the “upstream region of the MCM4 N-terminal tail”. This comment should be brought into the main body of the text and properly justified. It would also help if the authors clarified what they mean by the upstream region of the MCM4 N-terminal tail. Do they actually mean the N-terminus itself? It appears that the first 130 residues of MCM4 remain unaccounted for.

We thank the reviewer for these helpful comments and agree that the description of our interpretation of the unphosphorylated DH map required clarification. We have revised the text to explain what was done and how the additional Mcm4 density was assigned.

Our reinterpretation was based on comparative inspection of the deposited unphosphorylated DH map by Cheng et al with: (i) published DDK-bound DH structures, including Saleh et al. (2022), Greiwe et al. (2022), and Cheng et al. (2022), (ii) our new phosphorylated DH structure and (iii) our new MS/MSC structures. These comparisons allowed us to reassess density features in the unphosphorylated DH that were previously difficult to assign.

The unphosphorylated DH structure reported by Cheng et al. (2022) included density for the Mcm4 N-terminal serine/threonine-rich domain (NSD, regions 132-153) and is modelled contacting the rigid Mcm4 N-terminal surface, but visible additional unassigned density extending toward Mcm6 was neither modelled nor discussed. Importantly, Cheng et al. (2022) have already reported, in the same study, EM maps of Mcm4 NSD truncation mutants lacking the first 140 or 174 residues of Mcm4 (DHΔN140 and DHΔN174). In these mutants, the additional density observed in the full-length unphosphorylated DH near the Mcm6 surface is

no longer present, demonstrating that it originates from the Mcm4 N-terminal tail. Since residues 132-152 were already modelled at the Mcm4 surface, the density near Mcm6 most likely corresponds to residues N-terminal to this segment (within the deleted 1-140 region). Together, these observations support a model in which the Mcm4 NSD contacts both Mcm4 and Mcm6 in the unphosphorylated DH, forming a larger extended autoinhibitory interaction surface than previously thought, and is displaced upon phosphorylation. We have revised the text accordingly, added a more detailed explanation, and made the requested changes.

The colour coding information in Fig 1 should include blue for CDC7

We have updated Fig. 1 to include colour coding for both Cdc7 and Dbf4.

2) Sld3-Sld7 recognize the DDK-phosphorylated MCM2-7 DH.

The authors present their cryo-EM structure of the complex formed between the DDK-phosphorylated MCM2-7 double hexamer and Sld3-Sld7 at an estimated resolution of between 2.5 and 8 Angstroms.

In paragraph 4 page 6, they state that DNA can be lost in the glycerol gradient they use to purify the complex. It is not clear what the relevance of this observation is.

We clarified this in the text, stating: "The glycerol gradient ultracentrifugation purification method is ideal for purifying a more homogeneous complex for structural studies, but a disadvantage for this particular complex is that the DNA can slide out of the MCM2-7 DH, as previously observed with the MCM2-7 DH purified from yeast[1,5]. Despite this, the loss of DNA from the purified MCM2-7 DH does not substantially alter the overall conformation of the double hexamer, as DNA-bound and DNA-free structures are essentially identical[3,5], providing confidence in the MS complex structure." Nevertheless, we think this is an important point for the reader to note, because once the MCM2-7 DH is loaded and the ring is closed around dsDNA in vivo, the DNA is expected to remain topologically engaged with the complex.

In paragraphs 3 & 4, page 7, they assign regions of their cryo-EM map to Sld3 and Sld7. They need to explain the reasons for their assignments and to present supporting evidence such as agreement between fitted coordinates and experimental density. In particular, the assigned regions of Sld3 appear to consist of two short fragments (11 and 18 amino acids). In my view it would be quite challenging to make unique structural assignments of such fragments based solely on agreement with cryo-EM density at the resolution. These assignments need to be backed up by detailed comparison between the fitted coordinates and density and any other supporting evidence available.

Thank you for raising this point. We agree that the short Sld3 MRD1 and MRD2 fragments cannot be assigned from cryo-EM density alone with the same confidence as larger domains. We have now revised the manuscript to clarify that the assignments were integrative and based on

1.) Structural components: We generated map to model comparisons (Supplementary Fig. 10,18 and 19), which show bulky-residue density matching with the sequence and also provide local map-model correlation plots. Moreover, AlphaFold3 structure prediction data agree with the structure in large.

2.) Biochemical components:

The MRD motifs are consistent with mutagenesis and functional data, specifically the Sld3 MRD region (6E mutant) interaction analysis described by Deegan et al. (2016).

We revised the text to state that the local resolution permits backbone tracing of the Sld3 MRD1/MRD2 motifs and visible bulky side-chain features at several positions, supporting the sequence register. However, side-chain confidence is not uniform across all residues, and we are avoiding claims of complete residue-level certainty.

Furthermore, in paragraph 4, page7, they state that "Sld3 makes multiple contacts with MCM2-7 DH (Fig 3E)" We resolved two of these at high resolution". As far as I can see Fig 3E only shows the "high resolution" contact regions.

Thank you for pointing this out. Fig. 2e, previously Fig. 3e, emphasizes the better-resolved Sld3 MRD1/MRD2:MCM2-7 and Sld7 DD/MBH/hinge:MCM2-7 contact regions. The additional lower-resolution Sld3/Sld7 densities, which account for the remaining expected Sld3/Sld7 density in the MS complex, are introduced later in the manuscript in Fig. 5. We have revised the text to make this distinction clear and to indicate that the full lower-resolution MS map is described in the later section.

3) MCM2-7 ATP status and structural change.

The authors use 3D classification to identify conformers of the MCM2-7 Sld3-Sld7 complex differentiated by a twisting of the C-lobes. The authors relate this twisting to proposed differences in nucleotide binding between the phosphorylated MCM2-7 double hexamer and the MCM2-7 Sld3-Sld7 complex. Firstly, they need to explain why they are making this comparison. Is there a conformational change between the C-lobes of these two complexes equivalent to the twisting they describe in the classes of the MCM2-7 Sld3-Sld7 complex? Secondly, when they introduce the concept of differential nucleotide binding they need to

present the evidence that this occurs. Strangely, in this section they just refer to Figure 5h which is a schematic illustration. Later in the section titled "Structural changes upon MSC to CMG transition" on page 13, nucleotide binding is revisited and Extended data Figure 17 is referred to. Here, they show maps and fitted coordinates for the nucleotide binding regions of phosphorylated MCM2-7 and the MCM2-7 Sld3-Sld7 complex. Although the experimental data, at the resolution obtained it is not obvious to me that it is possible to reliably distinguish between ATP and ADP. At the chosen contour level, the terminal phosphate appears to be tightly constrained within the density contour in the structures identified as ATP, while in those designated as ADP there appears to be quite a lot of free space. Moreover, the placement of the coordinated Mg ions appear somewhat arbitrary and it is not obvious that the geometry of coordination is correct.

Many thanks for the detailed comments. We have revised the text accordingly and updated the figures. We note that, only in the MS complex intermediate, we observe a continuous twist in the C-lobe of the DH core, and from distinct structural snapshots (state II/III), we observe that the largest changes in twist occur at the Mcm2/6 interface, which is also happens to be bound by Sld3-Sld7 and exhibits a change in nucleotide occupancy compared to the phosphorylated-DH intermediate complex stage. We have provided a new figure to illustrate the observed structural change at the Mcm2/6 interface (Fig. 4d) and provide evidence for the assigned nucleotide occupancy (Fig. 4e and 4f). We have now revised the atomic structures and made sure the Mg^{2+} ions are correctly coordinated (Supplementary Fig. 25d-f). We agree that the local map resolution does not allow for unambiguous Mg^{2+} coordination assignment at all nucleotide binding sites. Accordingly, the Mg^{2+} ions were assigned for the phosphorylated-DH structure only at canonical nucleotide-associated metal sites supported by local density and refined with bond and angle restraints to expected contacts with the neighbouring Walker A serine and nucleotide phosphate oxygens. In contrast, Mg^{2+} ions are now omitted from MS and MSC structures, because weaker density made it difficult to unambiguously assign Mg^{2+} ion positions at all nucleotide-bound sites. We have revised the text to clarify how the ion positions were assigned.

Also, we provide the reviewer with a comparison: Here, we fitted either ATP or ADP to the nucleotide density at the Mcm2/6 interface in the phospho-DH map. The fit for ATP appears consistent with the map.

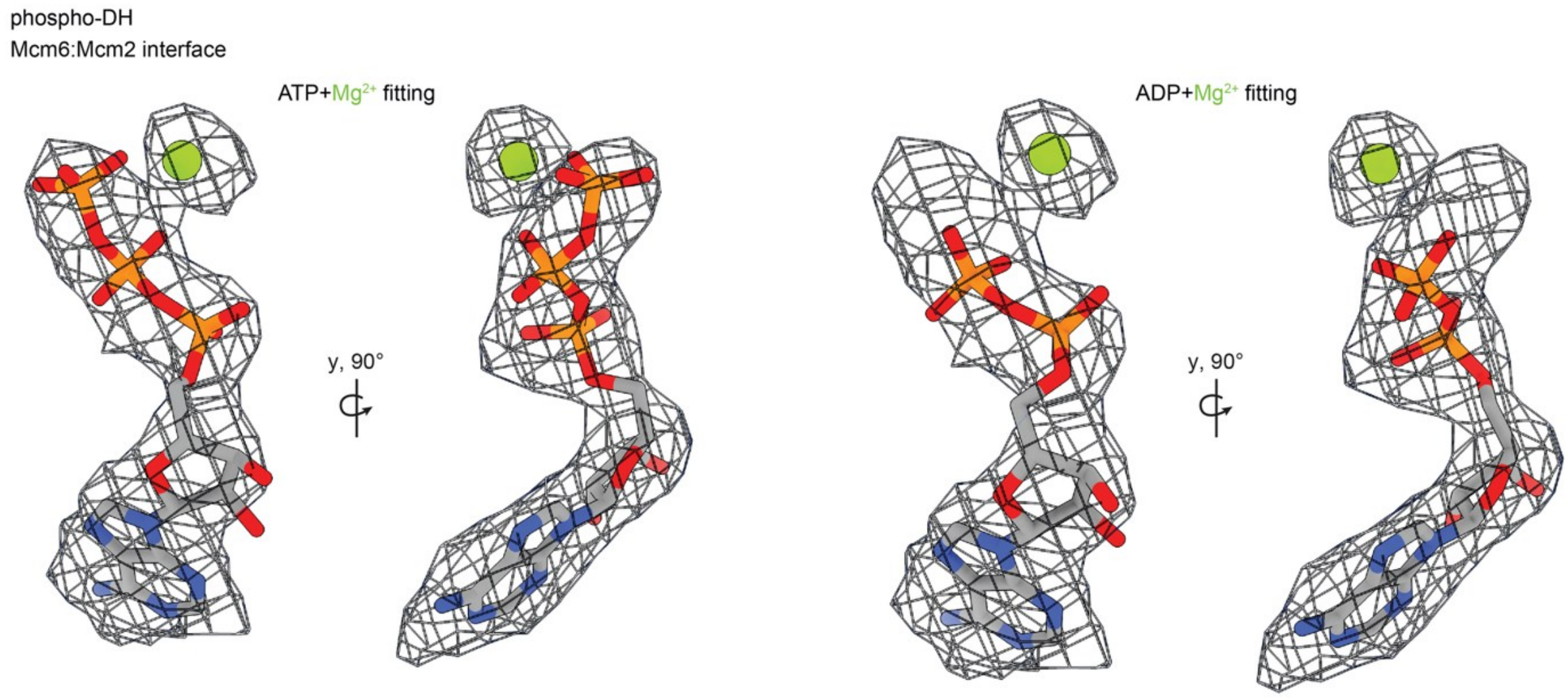

EM density supports ATP-Mg^{2+} occupancy at the Mcm6:Mcm2 interface of the phosphorylated-DH.

Zoomed view of the EM density at the Mcm6:Mcm2 nucleotide-binding interface of the phosphorylated-DH, with either ADP-Mg^{2+} or ATP-Mg^{2+} fitted into the map. The density shows continuous signal corresponding to three phosphate groups. ATP-Mg^{2+} fitting accounts for all density features, whereas ADP-Mg^{2+} fitting leaves unmodeled density at the gamma-phosphate position. This supports assignment of the Mcm6:Mcm2 site as ATP-bound.

References
Something has gone wrong with references 54 & 56 which are marked as INVALID CITATION.

Thank you for noticing this. We have updated the bibliography.

Reviewer #2 (Remarks to the Author)

In this manuscript, using the yeast system, the authors present an extensive cryo-EM and biochemical analysis of early eukaryotic replication initiation intermediates, focusing on how the DDK-phosphorylated MCM2–7 double hexamer recruits Sld3, Sld7, and Cdc45. Using a combination of high-resolution cryo-EM of the MCM2–7 DH, focused refinement strategies, biochemical reconstitution, mass photometry, and integrative modeling, the study defines structural snapshots of the MCM2–7–Sld3–Sld7 (MS) and MCM2–7–Sld3–Sld7–Cdc45 (MSC) complexes.

The work provides a coherent structural framework that consolidates prior genetic and biochemical observations: (i) DDK phosphorylation relieves Mcm4-mediated autoinhibition, (ii) Sld3/Sld7 recognize the activated MCM surface, and (iii) Sld3 recruits Cdc45 to initiate CMG assembly. A particularly strong aspect of the study is the identification and functional validation of a specific Sld3–Cdc45 interface within the Sld3 STD, supported by a designed mutant that selectively disrupts Cdc45 recruitment while preserving MCM binding. This represents a meaningful advance in understanding how Cdc45 is delivered to the helicase.

Overall, the manuscript offers a comprehensive and technically sophisticated structural synthesis of early helicase activation, and it will be of interest to the replication field. At the same time, several conclusions rely on model-supported interpretation and would benefit from more cautious framing.

In addition, the manuscript would benefit from clearer and more focused presentation. The current organization, with eight multi-panel figures, makes it difficult to identify the central advances. Streamlining the text and reducing the number of figures to emphasize the key novelties would substantially improve readability and impact.

Thank you for this suggestion. We have extensively revised the manuscript text for clarity and streamlined the main figures by reducing the number of panels and moving figures to the supplementary, thereby improving focus. In particular, the previous main Fig. 2 has been moved to the supplementary (now Supplementary Fig. 6), allowing the main figures to better emphasise key focused structural findings, namely Sld3-Sld7/Cdc45 in the context of the MCM2-7 DH.

Major points:

1) Resolution and certainty of Sld3/Sld7 interface assignments
While the global MCM maps reach high resolution, the Sld3 and Sld7 densities are peripheral, lower-occupancy, and locally lower resolution. The placement of Sld3 MRD1/MRD2 and Sld7 MBH/DD motifs relies heavily on focused refinement, prior biochemical data, and AlphaFold modeling. The manuscript at times implies residue-level or "high-resolution" definition of these interfaces, which is not fully supported by the density. The authors should include map-to-model correlation plots (or provide map-to-model FSCs) for the critical newly modelled regions of the proteins.

Thank you for raising this point. We agree that the short Sld3 MRD1 and MRD2 fragments cannot be assigned from cryo-EM density alone with the same confidence as larger domains. We have now revised the manuscript to clarify that the assignments were integrative and based on:

1.) Structural information:

We generated map to model comparisons (Supplementary Fig. 10,18 and 19), which show bulky-residue density matching with the sequence and also provide local map-model correlation plots. Moreover, AlphaFold3 structure prediction data agree with the structure in large.

Since the Sld3 MRD1/MRD2 motifs are short, boxed map-model FSC curves are not informative and instead, per-residue map-model CC values and density views were generated as the primary local validation metrics. For the larger Sld7 and Sld3/Cdc45 regions, we also compare the model to our fitted coordinates and provide map-to-model FSCs (Supplementary Fig.s 10, 18, and 19).

2.) Biochemical information:

The MRD motifs are consistent with mutagenesis and functional data, specifically the Sld3 MRD region (6E mutant) interaction analysis described by Deegan et al. (2016).

We revised the text to state that the local resolution permits backbone tracing of the Sld3 MRD1/MRD2 motifs and visible bulky side-chain features at several positions, supporting the sequence register. However, side-chain confidence is not uniform across all residues, and we are avoiding claims of complete residue-level certainty.

2) Asymmetry of Sld3/Sld7 binding to the MCM2–7 double hexamer
The reconstructions show Sld3/Sld7 density on only one hexamer of the MCM double hexamer. While this is consistent with the asymmetric nature of early initiation intermediates, the manuscript does not clearly distinguish whether this asymmetry reflects biology, limited occupancy, or focused classification bias.

Many thanks for the comment. We have addressed this now and added the following section to the manuscript to clarify this: "Here, we observed that both MCM2-7 hexamers are occupied by Sld3 MRD1/2, but the remaining Sld3 and Sld7 densities were best resolved on only one MCM2-7 hexamer (Fig. 2e). This apparent asymmetry may arise from several factors, including heterogeneous Sld3-Sld7 occupancy during purification, the focused classification strategy used to improve local density or conformational flexibility and therefore is unlikely to be associated with biological function."

3) Interpretation of Sld7 dimer "splitting" upon DH binding.

The observation that the Sld7 C-terminal dimerization domains are separated when bound to the DH is well supported geometrically. However, the manuscript frames this rearrangement as a functional mechanism that enhances recruitment efficiency. Given the bivalent nature of the DH, dissociation of the Sld7 dimer upon engagement of two Mcm6 sites appears to be an expected geometric consequence rather than a demonstrated regulatory feature.

Thank you for this comment. We agree that the separation of the Sld7 dimerization domains upon DH engagement is a geometric consequence of the C2 architecture of the DH rather than a demonstrated regulatory mechanism. Accordingly, we have revised the text and removed the remark regarding in vivo relevance and efficiency and now state that the functional significance of Sld7 dimerization in this context remains unknown.

4) Physiological relevance of oligomeric Sld3/Sld7/Cdc45 assemblies in solution
The mass photometry, crosslinking, and negative-stain EM data indicate that higher-order Sld3–Sld7 and Sld3–Sld7–Cdc45 assemblies can form in vitro. However, these experiments rely on crosslinking and low-resolution methods and lack mutational or functional validation of the inferred oligomerization interfaces.
Please temper conclusions regarding the biological relevance of these oligomeric assemblies and emphasize that they are observed under in vitro, crosslinked conditions.

Many thanks for your comment. For the Sld3-Sld7 complex, we performed native glycerol gradients without crosslinker in parallel with gradients with crosslinker (GraFix). In both conditions, Sld3 and Sld7 co-migrated in the same fractions, indicating that the observed complex is not an artifact of crosslinking and that crosslinking did not detectably alter complex

formation. This analysis is now shown in Supplementary Fig. 6, which was moved from the main figures to better focus the manuscript on the Sld3-7 in the context of the DH, rather than in-solution. We have also revised the text to state clearly where crosslinked samples were used and have tempered the biological interpretation of these results accordingly.

5) Validation of the Sld3–Cdc45 interface

The manuscript identifies a specific Sld3–Cdc45 interaction interface within the Sld3 STD and supports its functional relevance through mutational analysis. While this is one of the strongest aspects of the study, the structural interpretation of the interface would benefit from more explicit validation against the cryo-EM density. In particular, no map–model Fourier shell correlation (FSC) curves or local map–model correlation coefficients (CCs) are provided for the Sld3–Cdc45 interface region.

We have added all the requested items (FSC, CC), and it is shown in Supplementary Fig 19.

6) Explicit acknowledgment of model dependence

AlphaFold models are used extensively to guide interpretation of densities that are not fully resolved. While this is reasonable, the dependence on modeling is not always made explicit. Please clearly state where AlphaFold predictions are used to support structural interpretation, both in the Results and in the relevant figure legends.

In the context of MS and MSC, we have now clarified where the experimentally resolved initial templates, or prediction models, were used during model building, as shown in the text and figures (Supplementary Figs 10, 18, and 19). Also, we used AlphaFold as an independent comparison for flexible regions in the low-resolution map, including the Sld3/Sld7 N-terminal dimerization domains and Sld3 STD in MS (Figs. 5a and 5b) (additionally supported using XL-MS and interaction assays in context of MS – Fig. 5c-5h) and the Sld3/Sld7 N-terminal dimerization domains in MSC (Fig. 6g-6i).

In the context of the in-solution Sld3-Cdc45 analysis, we used AlphaFold3 predictions to (i) predict arrangement for low-resolution negative-stain EM envelope fitting (Supplementary Fig. 6).

We have ensured that where AlphaFold3 prediction models were used or contributed to interpretation, this is clearly acknowledged in the results, methods and relevant figure legends throughout the revised manuscript.

Minor points.

1. The manuscript states that “the Cdc45 structure has been identified only in the context of the CMG complex.” This is incorrect, as a structure of human Cdc45 in isolation was reported previously (Simon et al., 2016, Nature Communications). Please revise accordingly.

We have clarified that the yeast Cdc45 structure has been identified only as part of the CMG and added a citation for the isolated human CDC45 crystal structure.

2. The description of the 3D-FLEX analysis overstates the level of modeling performed. While conformational variability is visualized, atomic models are not fitted to the 3D-FLEX maps. Please rephrase to reflect this more accurately.

We have changed our wording from model to “visualize” to better reflect the data.

3. The reference list contains several invalid or incorrect citations. Please revise the references and in-text citations for accuracy and completeness.

Thank you for noticing this. We have now corrected it.

Reviewer #3 (Remarks to the Author)

There has been an explosion of molecular insights into the eukaryotic DNA replication machinery over the last decade. This is a very active area of research and of broad interest to multiple fields interested in molecular mechanisms relating to genome biology. Cryo-EM of replisome complexes reconstituted from purified proteins has been crucial in this area. Here, Speck and colleagues use this approach to study the conversion of MCM double hexamers into pairs of replisomes during DNA replication initiation. These initiation steps have been extensively studied, but there are still very few published structures of intermediates in the CMG assembly pathway, which represents an important gap in our understanding.

The authors solve structures of multiple complexes containing different combinations of the 'firing factors' Sld3-7 and Cdc45 bound to Mcm2-7 double hexamers. The structures provide interesting and novel insights into how Sld3, Sld7 and Cdc45 bind to Mcm2-7 double hexamers, and how these interactions are regulation by the Dbf4-dependent kinase. The structures presented in this manuscript represent a step forward in our molecular understanding of replication initiation. I detail below the concerns that I think should be addressed before publication.

Major points:

The manuscript as a whole is unnecessarily long, with too many figures and very dense text. This has the overall effect of burying the main messages (which are themselves novel and interesting) in a sea of data. I recommend significantly reorganising the manuscript. For example, some of the data in Figure 2 and 4 could be omitted or at least shifted to the supplementary material. There is also some redundancy in other figures (e.g. 8g vs 8h, 7a-c, 6b-c, 1f vs. 4h) which could be addressed. This would make the manuscript much more accessible to the reader.

Thank you for your suggestions. We have reviewed the manuscript and made extensive changes to the text and main figures to improve clarity and readability. We have moved Fig. 2 to the Supplementary Information, now Supplementary Fig. 6, streamlined Fig. 4, and removed redundant panels from other figures where appropriate. We have retained Fig. 8h, but have clarified that it differs from Fig. 8g because it represents the proposed in vivo pathway and highlights noteworthy differences from the in vitro structural pathway. We hope these changes make the central findings clearer and improve the overall flow of the manuscript.

The Sld3-Mcm2-7 interactions delineated by the authors' structures are of great interest, and provide a key molecular insight into how DDK phosphorylation of Mcm2-7 facilitates Sld3 binding. Although based on lower confidence portions of the EM density maps (see PDB report), these surfaces on Mcm4/6 and Sld3 have been previously subjected to mutational / functional analysis by the Stillman, Bell and Diffley labs, in support of the authors' conclusions. The same cannot be said, however, for the completely novel Sld7-Mcm interactions described in the paper. Although Sld7 is not essential, its deletion does confer defective S-phase progression and a slow growth phenotype in budding yeast (PMID: 21487389). The authors are now in a position to assess the relative contributions of e.g. Mcm binding vs. Sld7 dimerisation to Sld7 function in vivo. This is relatively straightforward yeast genetics and would significantly improve the manuscript.

Thank you for this suggestion. We have assessed the phenotype in the referenced paper and note that complete deletion of Sld7 results in only a very modest growth defect. We therefore believe that mutational analysis of a single interface in Sld7 may not provide a measurable effect, rather at best produce a subtle or intermediate phenotype that would be difficult to interpret unambiguously, particularly given that Sld7 also contributes to Sld3 binding/stability and dimerization. We therefore believe that such an experiment would not provide mechanistic insight beyond what can be inferred from the complete deletion of Sld7.

Minor points:

How confident are the authors of their assignment of the 'extra' density on the surface on Mcm6 to the Mcm4 N-terminal tail? I appreciate that this density is absent in the DDK phosphorylated structure, but this could be a product of some other phosphorylation-induced conformational change. It would be quite straight forward to solve a structure of a double hexamer using mutant Mcm2-7 in which this tail was deleted.

We thank the reviewer for this comment. The basis of our confidence in the assignment comes from the suggested experiment that has already been previously performed.

Our reinterpretation of the unphosphorylated DH map was based on comparative inspection of the deposited unphosphorylated DH map by Cheng et al with: (i) published DDK-bound DH structures, including Saleh et al. (2022), Greiwe et al. (2022), and Cheng et al. (2022), (ii) our new phosphorylated DH structure and (iii) our new MS/MSC structures. These comparisons allowed us to reassess density features in the unphosphorylated DH that were previously difficult to assign.

The unphosphorylated DH structure reported by Cheng et al. (2022) included density for the Mcm4 N-terminal serine/threonine-rich domain (NSD, regions 132-153) and is modelled contacting the rigid Mcm4 N-terminal surface, but visible additional unassigned density

extending toward Mcm6 was neither modelled nor discussed. Importantly, Cheng et al. (2022) have already reported, in the same study, EM maps with Mcm4 NSD truncation mutants lacking the first 140 or 174 residues of Mcm4 (DHΔN140 and DHΔN174). In these mutants, the additional density observed in the full-length unphosphorylated DH near the Mcm6 surface is no longer present, demonstrating that it originates from the Mcm4 N-terminal tail. Since residues 132-152 were already modelled at the Mcm4 surface, the density near Mcm6 most likely corresponds to residues N-terminal to this segment (within the deleted 1-140 region).

It is not obvious why the mass photometry in Fig. 2 was performed with crosslinked complexes. Has this been done with native Sld3-7 as well? This should at least be commented upon.

Thank you for raising this point. Mass photometry required dilution of the Sld3-Sld7 complex, which can promote complex dissociation; crosslinking was therefore used to stabilize the complex during measurement. Crosslinking was also used for negative-stain EM grid preparation because the low pH of the stain can destabilize protein complexes.

To address whether the observed Sld3-Sld7 complex was crosslinking-dependent, we performed native glycerol gradients without crosslinker in parallel with GraFix gradients. In both conditions, Sld3 and Sld7 co-migrated in the same fractions, indicating that the observed complex is not an artifact of crosslinking and that crosslinking did not detectably alter complex formation. This analysis is now shown in Supplementary Fig. 6, which was moved from the main figures to better focus the manuscript on Sld3-Sld7 in the context of the DH rather than in solution. We have also revised the text to state clearly where crosslinked samples were used and have tempered the biological interpretation of these results accordingly.

According to PMID: 26912723, the Sld3-Cdc45 interaction in Fig. 7 has been functionally assessed (like the Sld3-Mcm interaction that the authors discuss extensively) – this could be commented upon.

Many thanks for this helpful comment. We show a plot in Supplementary Fig 2a, detailing the interaction interface determined from the reference mentioned, and other relevant studies. We have now discussed this previously identified interaction region between Sld3:Cdc45 in more detail in the relevant section, mentioning several residue substitution mutants and their impact on the Sld3-Cdc45 interaction and DNA synthesis.

The XL-MS data presented in Fig. 5 is very hard to follow. This is partly because of how busy the structural representations of the cross links are, but also because the data is buried in a figure containing 12 panels covering everything from Sld7 dimerisation to nucleotide binding

by Mcm2-7 and mapping DDK-independent interactions in the MS complex. This is a general theme throughout the paper (see major point above) and is not helpful for the reader.

Thank you for this comment. We have completely reorganised Fig. 5 and the associated Supplementary Fig. 13. The figure now focuses specifically on the low-resolution MS map, fitting and interpretation of AF3 prediction models and their validation by the XL-MS data. We simplified the crosslinking structural representations by showing only crosslinks with mapped distances ≤40 Å, enlarged the structural views, and reduced the number of panels where possible. Data relating to nucleotide binding and the Sld7 dimerization domain have been moved to figures where they fit more naturally within the narrative, avoiding the overcrowding that made the original figure difficult to follow.

We sincerely thank the reviewers for their time and the feedback. The final suggestions are well spotted and will significantly further improve the presentation of the manuscript and remove errors. We have now addressed the remaining comments in the revised manuscript and provide a detailed point-by-point response below.

Reviewer #1 (Remarks to the Author):

The paper has been substantially revised taking into account the reviewers' comments and is greatly improved. Overall, I consider the paper ready for publication.

On rereading, however, I did run in difficulties understanding the schematic diagram in figure 1c left panel, illustrating the autoinhibitory binding of MCM4 N-terminal regions to activation factor binding sites on MCM4 and MCM6. Here in the lower hexamer, MCM4 (pink) is shown on the right of MCM6 (lilac). This seems to contradict the arrangement in the density maps in figure 1 and elsewhere which show MCM4 to the left of MCM6 in the lower hexamer. Is this intentional? The arrangement is repeated in related schematic diagrams in figure 1, figure 3 and figure 8.

We thank the reviewer for identifying this inconsistency in the schematic diagrams. The schematics were intended as simplified representations of the MCM2-7 double hexamer, but we agree that the relative placement of Mcm4 and Mcm6 in the lower hexamer was confusing because it did not match the orientation shown in the corresponding density maps.

We have corrected the schematics in Figure 1, Figure 3 and Figure 8 so that the relative positions of Mcm4 and Mcm6 are now consistent with the structural views throughout.

Reviewer #2 (Remarks to the Author):

The authors have responded thoroughly to my comments, and the manuscript is substantially improved. The text is clearer and better focused, the integrative basis for the peripheral Sld3/Sld7 assignments is now stated transparently, and the additional map-to-model validation is provided. The Sld3–Cdc45 interface (the central advance) is now convincingly supported both structurally and functionally.
I have one remaining request, which concerns wording rather than data, and I recommend publication once it is addressed. The local validation for the MSC complex (Supplementary Fig. 18) is considerably weaker than for the MS complex: for MRD1, the chain and side-chain correlations (≈0.21 and ≈0.18) are essentially at the noise level, and MRD2 is only marginally better. This is at odds both with the statement that the MSC MRD regions were "resolved at a similar local resolution to those in the MS map" and with the description elsewhere of the Sld3 MRD as "resolved to high resolution." I ask that the text describe these MRD motifs consistently as backbone/placement-level assignments, without implying high-resolution or sequence-register certainty, particularly for the MSC complex.
All other points from my original review have been satisfactorily addressed.

We thank the reviewer for their positive assessment of the revised manuscript and for this final, constructive comment. We agree that our previous wording overstated the confidence of the peripheral Sld3 MRD/Sld7 assignments, particularly for the MSC complex, and we have revised the text throughout to consistently describe these motifs as backbone-level assignments, without implying high-resolution or sequence-register certainty.

Specifically:

In the Results section describing the MS complex, we have softened the description of the Sld3 MRD1/MRD2 and Sld7 contact regions to remove the implication of sequence register certainty:

"For the peripheral Sld3 MRD1/MRD2 motifs and the Sld7 MBH/C-terminal helices, the local density supports their overall placement on the MCM2-7 DH and permits tracing of the main backbone features. Because map quality varies across these regions, we interpret them conservatively as backbone level assignments and do not infer uniform side-chain or sequence register certainty."

We have removed the phrase "resolved to high resolution" in reference to the Sld3 MRD regions. This sentence now reads:

"Previously, the Sld3 MRD1/MRD2 regions, which we have modeled at backbone level confidence (Fig. 2h), were shown to be essential for Sld3 interaction with MCM2-7."

In the Results section describing the MSC complex, we have removed the statement that these regions were "resolved at a similar local resolution to those in the MS map," since this is not supported by the correlation coefficients in Supplementary Fig. 18. This now reads:

"In the MSC map, the peripheral Sld3 MRD and Sld7 contact densities were interpreted conservatively as backbone level assignments, guided by local density, AlphaFold3 predictions and prior biochemical constraints. These densities support the overall placement of the motifs, but do not justify high-resolution or sequence-register certainty, particularly for the Sld3 MRDs."

We thank the reviewer again for their careful reading, which has helped us state our confidence in these peripheral regions more precisely and consistently throughout the manuscript.

We have also added clarifying statements in the legends of Supplementary Figures 10 and 18.

Reviewer #3 (Remarks to the Author):

I am satisfied that all comments have been addressed and support publication of this manuscript.

We thank the reviewer for their positive assessment of the revised manuscript .2nd Revision Response
